# Exploring the differential localization of protein kinase A isoforms in *Candida albicans*

**DOI:** 10.1128/msphere.01037-24

**Published:** 2025-02-25

**Authors:** Saif Hossain, Zhongle Liu, Nicole Robbins, Leah E. Cowen

**Affiliations:** 1 Department of Molecular Genetics, University of Toronto 204248 , Toronto, Ontario, Canada; University of Georgia, Athens, Georgia, USA

**Keywords:** protein kinase A, filamentation, *Candida albicans*, fungal pathogen, protein localization

## Abstract

The cAMP-dependent protein kinase A (PKA) plays important roles in a wide range of biological processes in eukaryotic organisms. In the fungal pathogen *Candida albicans*, PKA is a critical regulator of morphological transitions, which are a key virulence trait. PKA is composed of two catalytic isoforms, Tpk1 and Tpk2, which are often thought to act together in a complex with the regulatory subunit Bcy1. Although Tpk1 and Tpk2 have some redundant functions, they also have distinct cellular functions for which the mechanistic underpinnings remain largely elusive. Here, we constructed functional GFP-tagged fusion proteins for Tpk1, Tpk2, and Bcy1 to explore the localization of PKA isoforms. We observed that the PKA holoenzyme is mainly found in the cytoplasm, as Bcy1 is always excluded from the nucleus. Under glucose-replete conditions, both Tpk1 and Tpk2 translocate into the nucleus from the cytosol. In the presence of glycerol, Tpk1 resides in the cytosol, whereas Tpk2 and Bcy1 become enriched on the vacuolar membrane. As the C-terminal domains of Tpk are highly homologous, we investigated the localization and function of hybrid Tpk proteins with exchanged N-terminal domains. We found the catalytic C-terminus of Tpk1 is required for morphogenesis in solid medium, whereas the C-terminus of Tpk2 is critical for filamentation in liquid. Interestingly, the N-terminus of Tpk2 drives its localization to the vacuolar membrane. Our work highlights environmentally contingent localization patterns for the PKA subunits and suggests that the nuclear localization of Tpk is not sufficient to induce the filamentation program in a leading fungal pathogen of humans.

**IMPORTANCE:**

Fungal pathogens have a devastating impact on human health worldwide. They infect billions of people and kill more than 2.5 million per year. *Candida albicans* is a leading human fungal pathogen responsible for causing life-threatening systemic disease in immunocompromised individuals. A key virulence trait in *C. albicans* is the ability to switch between yeast and filamentous forms. The conserved protein kinase A (PKA) regulates diverse functions in the cell, including growth and filamentation. Although PKA has been studied in *C. albicans* for decades, the subcellular localization of PKA has not been thoroughly investigated. Here, we constructed functional GFP-tagged PKA subunits to explore their localization. We identified differential localization patterns for the PKA subunits that are carbon-source dependent and report that these proteins localize into foci in response to diverse environmental stresses. These findings further our understanding of a critical regulator of growth and virulence in *C. albicans*.

## INTRODUCTION

*Candida albicans* is a commensal fungus frequently found as a benign member of the human mucosal and skin microbiota. Studies have estimated that as many as 70% of healthy individuals are colonized with *C. albicans* at any given time (1). However, *C. albicans* is also an opportunistic pathogen that can cause superficial infections in immunocompetent individuals, as well as life-threatening systemic disease in immunocompromised patients (2). *C. albican*s causes ~1.6 million cases of bloodstream infections per year with mortality rates estimated at 64% (3). This is in part enabled by a suite of virulence factors the organism employs to thrive within a human host, including the expression of adhesins that mediate attachment to epithelial cells, the secretion of hydrolytic enzymes that induce host cell damage, the production of biofilms that are resistant to antifungal drugs, and the ability to transition between yeast and filamentous growth states through a process called morphogenesis (4). Understanding the complex signaling pathways that govern these virulence traits is imperative for our understanding of how *C. albicans* thrives in a human host.

The cAMP-Protein Kinase A (PKA) pathway plays important roles in many facets of *C. albicans* biology, including growth, virulence, stress response, metabolism, and morphogenesis (5). Morphogenetic plasticity, including the ability to transition between yeast and filamentous forms, is a key virulence trait of *C. albicans* and the cAMP-PKA pathway is a critical regulator of this developmental transition (4). This signaling cascade becomes activated when the GTPase Ras1 is stimulated, leading to the release of the adenylyl cyclase Cyr1, and the induction of cAMP (6, 7). cAMP-PKA signaling can be stimulated by cues such as peptidoglycan (8), physiological CO_2_ (9), serum (10), and *N*-acetylglucosamine (11). Elevated cAMP levels are important for activating the protein kinase A (PKA) complex, which is a heterotetramer of two catalytic subunits (Tpk1 and/or Tpk2) and two copies of a regulatory subunit (Bcy1). cAMP binds to Bcy1, leading to the release and activation of Tpk1 and Tpk2 (12). The catalytic subunits play distinct roles in filamentation, where Tpk1 is more important for filamentation in solid medium, and Tpk2 is more important for filamentation in liquid (13). PKA is thought to phosphorylate downstream transcription factors, such as Efg1, responsible for activating the transcriptional program governing filamentous growth (14). As such, deletion of both *TPK1* and *TPK2* abrogates filamentation in all *in vitro* conditions (15).

PKA localization varies widely across different species. In mammalian cells, the regulatory subunit targets the PKA holoenzyme to defined subcellular compartments through interaction with AKAPs (A kinase anchoring proteins) (16, 17). Additionally, the catalytic subunits are targeted to the cytoplasm, nucleus, and cell membrane by C-KAPs (catalytic kinase anchoring proteins) (18). However, anchoring proteins have not been identified in yeast and different subcellular localizations have been observed. In the fission yeast *Schizosaccharomyces pombe,* the PKA holoenzyme is localized to the cytoplasm, and when cells are cultured to log phase under glucose-rich conditions, both regulatory and catalytic subunits of PKA become concentrated in the nucleus and diffusely distributed in the cytoplasm (19). Interestingly, the budding yeast *Saccharomyces cerevisiae* has three catalytic subunits of PKA: Tpk1, Tpk2, and Tpk3. In cells growing in glucose, Bcy1 and Tpk2 mainly localize to the nucleus, whereas Tpk1 and Tpk3 show nuclear-cytoplasmic distribution (20). When cells are grown in glycerol, all PKA subunits display mostly cytoplasmic localization in both *S. pombe* and *S. cerevisiae* (19, 20). Given the wide variation in PKA subunit distribution across species and growth conditions, inferring protein localization from model organisms can be misleading, and thus, it is important to explore PKA localization in the relevant species of interest.

In *C. albicans*, Efg1 is a substrate of Tpk2 and a key transcriptional regulator of filamentation that has been exclusively detected in the nucleus (21, 22). As such, the current paradigm is that Tpk2 likely interacts with and phosphorylates Efg1 in the nucleus. However, a comprehensive investigation of the subcellular localization of PKA subunits in *C. albicans* remains incomplete. Here, we explored the localization of PKA isoforms in *C. albicans*. We generated and characterized functional GFP-tagged fusion proteins of Tpk1, Tpk2, and Bcy1. Our work suggests the PKA holoenzyme mainly exists in the cytosol, as Bcy1 is excluded from the nucleus. Dynamic translocation of Tpk1 and Tpk2 to the nucleus can be observed in cells growing in glucose. In cells growing in glycerol, a less favorable carbon source, Tpk1 and Tpk2 fail to show a similar translocation, and instead, Tpk2 and Bcy1 localize to the vacuolar membrane. We also observed that in glycerol conditions, an additional isoform of Tpk2 is translated from an alternative start codon 93 nucleotides upstream of the annotated start site. Overall, this work defines the subcellar localization of PKA subunits in response to distinct environmental conditions and sheds new insights into the expression and localization of this important regulator of growth, virulence, morphogenesis, and stress response in *C. albicans*.

## RESULTS

### Generation of functional GFP-tagged subunits of PKA

**Fig 1:**
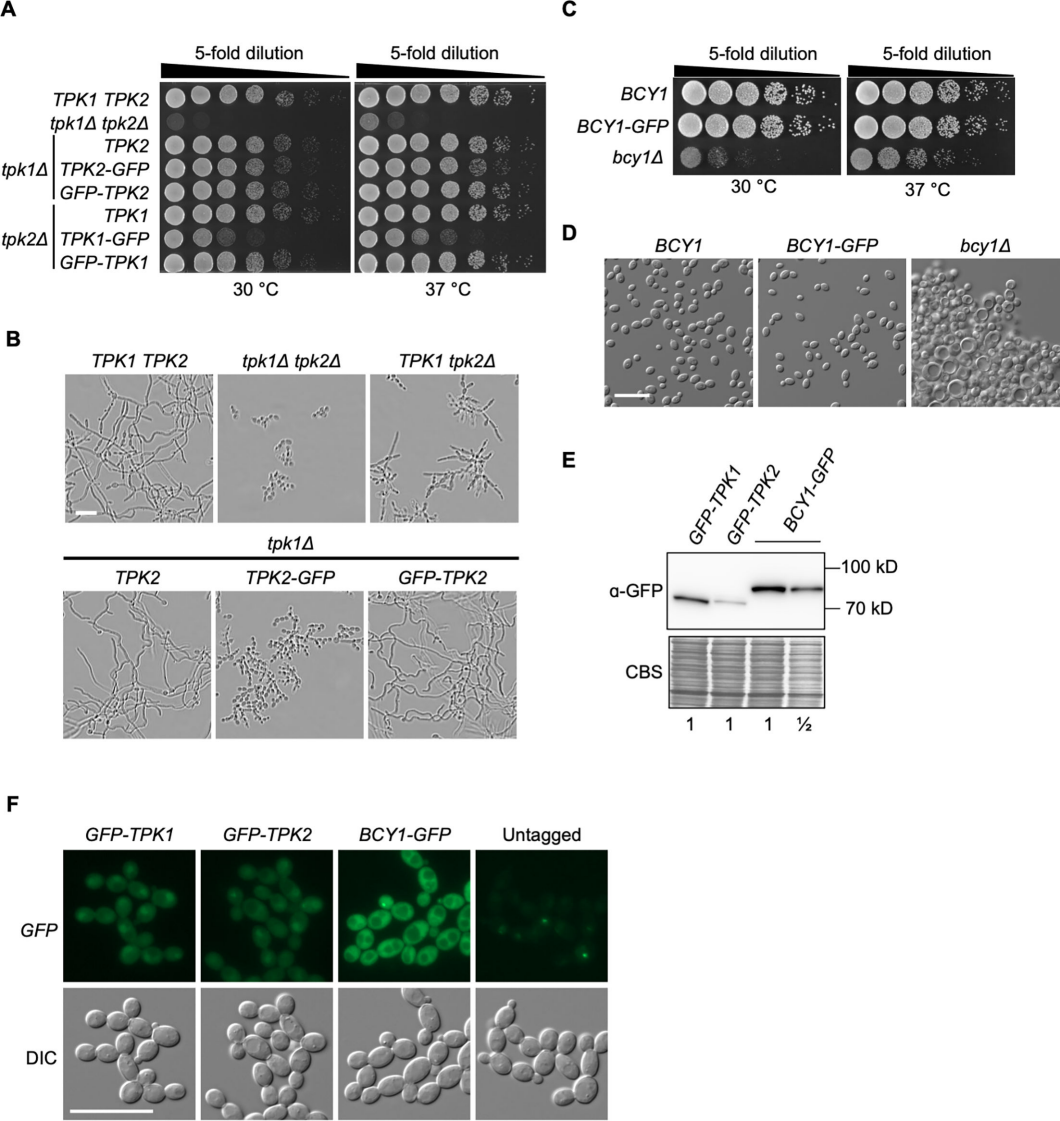
Generation of functional GFP-tagged subunits of PKA. (**A**) N-terminal GFP tagged Tpk1 better supports growth than the C-terminal tagged version. Cells from overnight cultures were 5-fold serially diluted and spotted on YPD medium. Agar plates were incubated at 30°C or 37°C and imaged after 24 h. (**B**) N-terminal GFP-tagged Tpk2 better supports filamentation than the C-terminal tagged version. Strains were grown under static conditions in the presence of 5 mM *N*-acetylglucosamine at 37°C for 4 h prior to imaging. (**C**) C-terminal GFP-tagged Bcy1 is functional on solid medium. Cells were grown under the same conditions as for panel **A**. (**D**) C-terminal GFP-tagged Bcy1 is functional in liquid medium. Overnight cultures were grown under shaking in YPD medium prior to imaging. (**E**) PKA protein levels in exponentially growing cells in glucose. Strains were grown in YPD with shaking for 5 h before protein extraction and western blotting using an anti-GFP antibody. Coomassie blue staining (CBS) of the blot was used to confirm equal loading. For comparison, loading of the Bcy1-GFP sample was titrated. (**F**) GFP-tagged PKA subunits can be detected by fluorescence microscopy. Images were taken from strains growing exponentially in YPD medium for 3 h. All genotypes are homozygous. Scale bar, 20 µm. GFP, green fluorescent protein.

Previously, PKA localization was investigated through immunofluorescence using strains harboring C-terminal triple hemagglutinin (HA) tags on the various PKA subunits, suggesting Tpk1, Tpk2, and Bcy1 can tolerate epitope tags on the C-terminus of the proteins (23). Additionally, Tpk1 localization was assessed through a C-terminal GFP tag; however, the functionality of the allele was not confirmed (24). In this work, to study the subcellular localization of PKA catalytic subunits, we first fused GFP to the C-terminus of Tpk1 and Tpk2. The modification was performed on both alleles while maintaining expression under the control of the native promoters, and phenotypes were compared with a deletion mutant. Deletion of both *TPK* genes caused a severe growth defect (Fig. 1A), as seen previously (15). When Tpk1-GFP was expressed as the sole source of Tpk, we saw a substantial growth defect compared with the wild-type strain at both 30°C and 37°C, suggesting C-terminal GFP-tagged Tpk1 was hypomorphic (Fig. 1A). Interestingly, when GFP was fused to the N-terminus of Tpk1 and expressed as the only source of Tpk, GFP-Tpk1 grew similar to wild-type cells at both temperatures (Fig. 1A). When N- or C-terminal GFP-tagged Tpk2 were expressed as the sole source of Tpk, no growth defect was observed on solid agar at both 30°C and 37°C relative to the wild type. Unlike Tpk1, Tpk2 is not required for normal growth at either temperature on a solid medium. Therefore, we assessed the functionality of tagged-Tpk2 alleles under liquid filament-inducing conditions and found that Tpk2-GFP was unable to restore filamentation when expressed as the sole source of Tpk, whereas GFP-Tpk2 was able to maintain filamentous growth similar to wild-type cells (Fig. 1B). Thus, C-terminal GFP-tagged Tpk alleles appear hypomorphic, whereas N-terminal GFP-tagged Tpk alleles are fully functional.

Next, we fused GFP to the C-terminus of the regulatory subunit of PKA, Bcy1. Bcy1-GFP was able to maintain growth comparable with cells expressing wild-type Bcy1 and did not cause any growth defect at 30°C or 37°C, as was seen upon deletion of *BCY1* (Fig. 1C). Additionally, a *bcy1* homozygous deletion mutant flocculated and displayed abnormal cellular morphology with enlarged vacuoles, whereas cells expressing Bcy1-GFP displayed normal cellular morphology, suggesting Bcy1-GFP was functional (Fig. 1D).

Finally, we monitored the expression of the functional GFP-tagged PKA subunits using immunoblotting and fluorescence microscopy. When grown in YPD medium, Tpk1 was expressed to a greater degree compared with Tpk2, and Bcy1 was expressed at even higher levels than either catalytic isoform (Fig. 1E). In addition, all three PKA subunits were detected in logarithmic growing cells by fluorescence microscopy (Fig. 1F). Thus, based on this characterization, we report the successful generation of functional GFP-tagged subunits of PKA in *C. albicans*.

### C-terminus of Tpk1 and Tpk2 are important for filamentation

**Fig 2:**
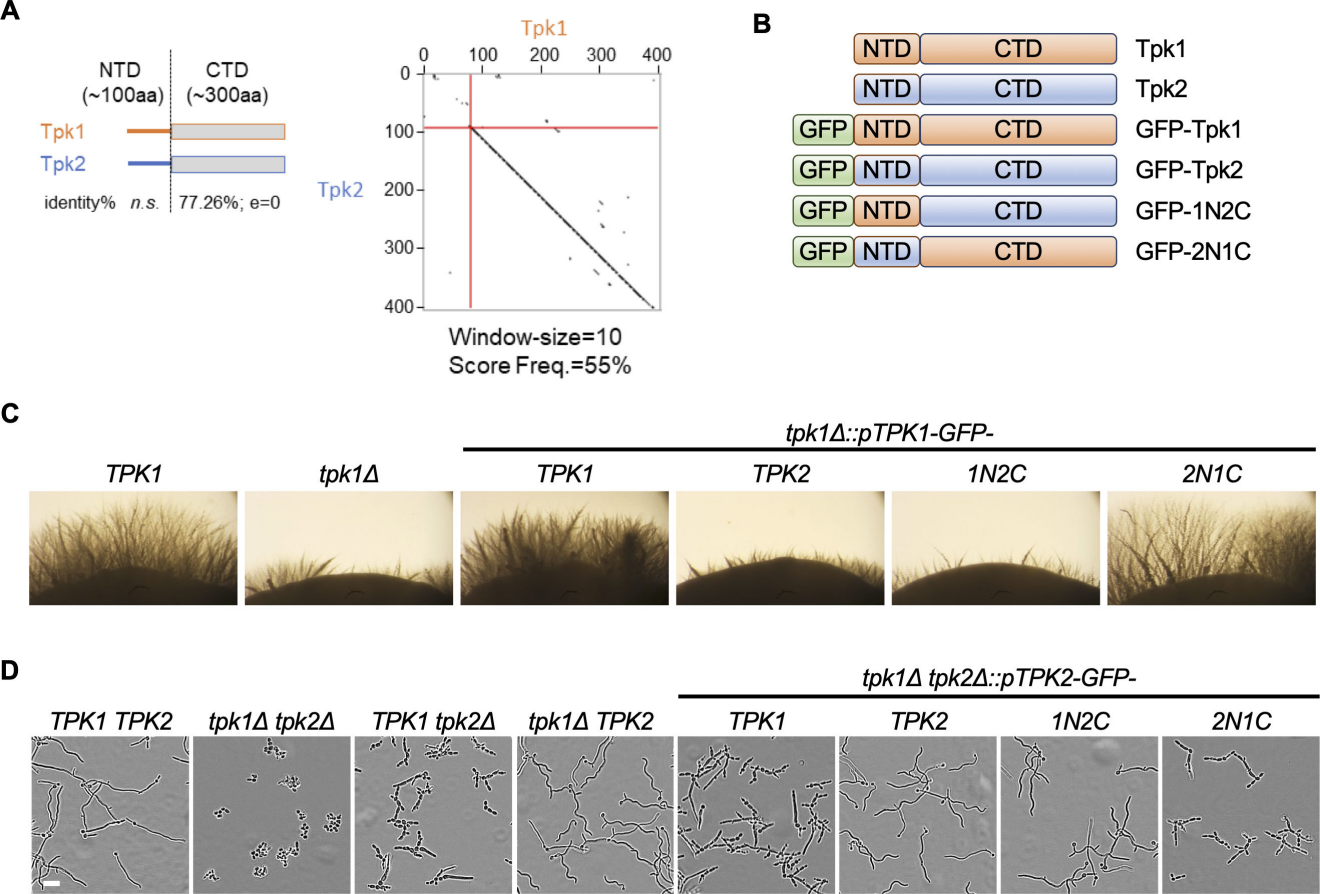
C-terminal kinase domain determines the condition-dependent role of Tpk1 and Tpk2 in filamentation. (**A**) Dot plot showing high similarity between Tpk1 and Tpk2 C-terminal domain (CTD) and divergence of the N-terminal domain (NTD) (window-size = 10; threshold = 55%). (**B**) Schematic of chimeric proteins. (**C**) The C-terminus of Tpk1 is important for filamentous growth on solid medium. Cells from overnight cultures were spotted on YPD medium and incubated at 30°C and imaged after 7 days under a dissection microscope. Images show the edges of colonies on agar plates. (**D**) The C-terminus of Tpk2 is important for filamentous growth in liquid medium. Strains were grown under static conditions in the presence of 5 mM *N*-acetylglucosamine at 37°C for 4 h prior to imaging. All genotypes are homozygous. Scale bar, 20 µm.

In *S. cerevisiae,* the PKA catalytic subunits share high similarity throughout the C-terminus, whereas the N-terminal region of these proteins does not share any similarity and is heterogeneous in length (25). Similarly, in *C. albicans,* the C-terminus of Tpk subunits share ~77% identity, whereas the N-terminus of the proteins is not conserved (Fig. 2A). To dissect whether the N-terminus or the C-terminus of the Tpk isoforms was important for regulating filamentous growth in liquid or solid media, we constructed chimeric proteins encoding either the N-terminal domain of Tpk1 fused to the C-terminal domain of Tpk2 (1N2C) or the N-terminal domain of Tpk2 fused to the C-terminal domain of Tpk1 (2N1C) (Fig. 2B). Given that the catalytic subunits of PKA play distinct roles in filamentation, where Tpk1 is more important for filamentation on solid medium and Tpk2 is more important for filamentation in liquid (13), the fusion proteins were tested for their ability to undergo filamentous growth in the different media states. Deletion of *TPK1* severely impaired filamentous growth on solid medium. This morphogenetic block was alleviated upon reintroduction of wild-type GFP-Tpk1 or the GFP-2N1C chimera. In contrast, neither wild-type GFP-Tpk2 nor the GFP-1N2C hybrid restored filamentous growth on solid (Fig. 2C). This indicates the catalytic C-terminal domain of Tpk1 is necessary for filamentation on solid-inducing medium. Similarly, deletion of *TPK2* impaired filamentation in liquid medium, forming short filaments and peudohyphae (Fig. 2D). This phenotype was reversed by the introduction of wild-type GFP-Tpk2 or the GFP-1N2C chimera, but not by GFP-Tpk1 or GFP-2N1C. Thus, the catalytic portion of Tpk2 is responsible for regulating filamentous growth in liquid-inducing medium (Fig. 2D). Overall, our findings are consistent with previous observations that the C-terminus of Tpk1 and Tpk2 are important for filamentous growth on solid- and liquid-inducing conditions, respectively (13), and introduction of an N-terminal GFP epitope does not impact the function of such chimeric proteins.

### Tpk1 and Tpk2 dynamically translocate into the nucleus in a carbon source-dependent manner

**Fig 3:**
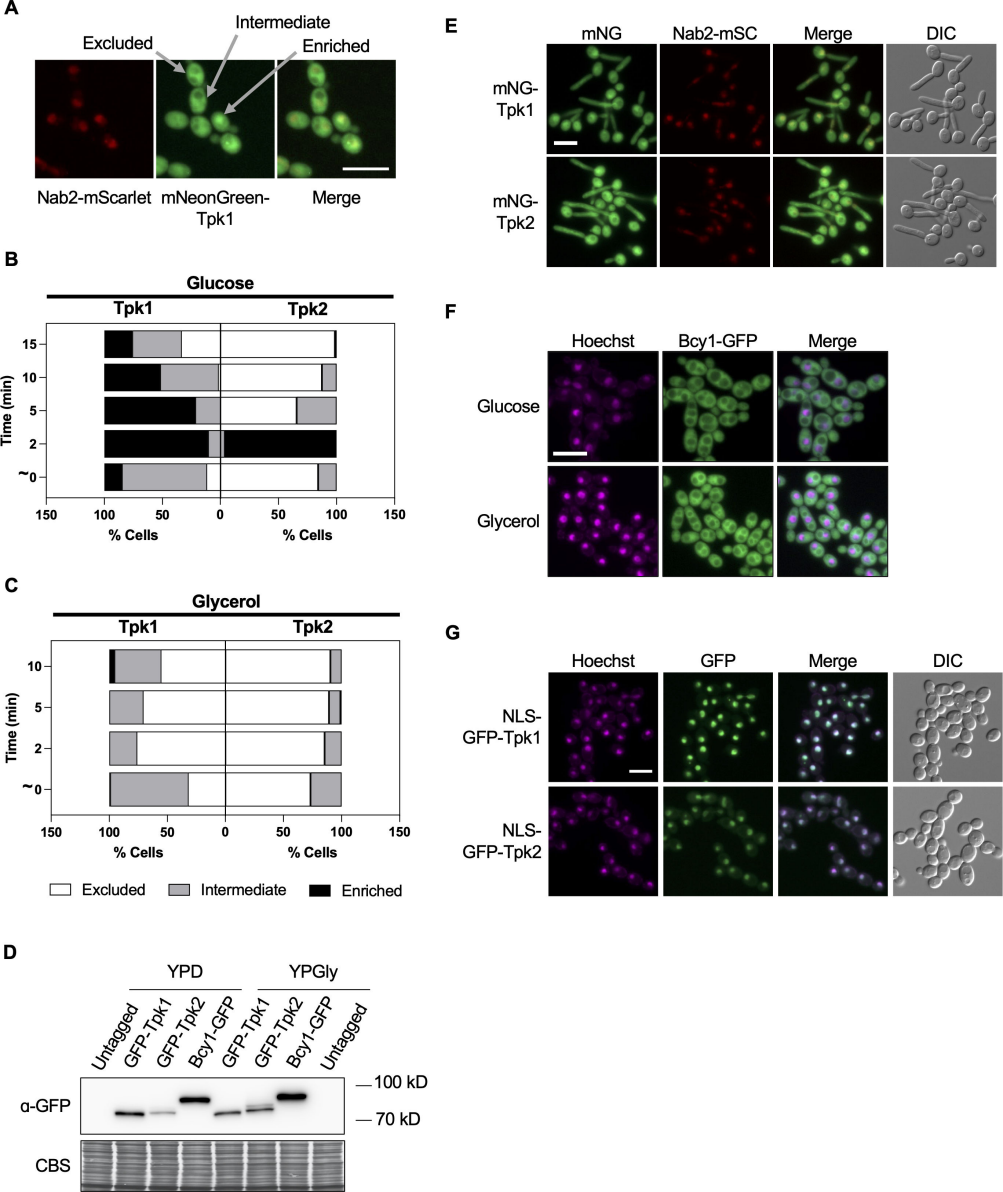
Tpk1 and Tpk2 dynamically translocate into the nucleus in a carbon source-dependent manner. (**A**) Subcellular localization of Tpk1 in rapidly growing cells in glucose. (**B**) Tpk1 and Tpk2 transiently localize to the nucleus upon contact with slides under glucose-replete conditions with subsequent varying kinetics of nuclear export. Strains were grown in YPD at 30°C with shaking for 3 h before imaging. Cells were imaged at periodic intervals starting from immediately after adding to the glass slides up to 15 min post-contact. For localization distribution, approximately 100–150 cells were counted per time point. Three biological experiments were performed for panels **B and C**, and similar trends and kinetics were observed for the localization of Tpk. One biological replicate is shown. (**C**) Tpk1 and Tpk2 do not localize to the nucleus when *C. albicans* utilize glycerol as the carbon source for growth. Strains were grown in YPG medium at 30°C with shaking for 3 h before imaging as described above. (**D**) PKA protein levels in cells grown in glucose or glycerol. Strains were grown in YPD or YPGly with shaking for 5 h before protein extraction and western blotting using an anti-GFP antibody. Coomassie blue staining (CBS) of the blot was used to confirm equal loading. (**E**) Subcellular localization of Tpk subunits in filaments. Strains were grown in the presence of 5 mM *N*-acetylglucosamine at 37°C for 1 h prior to imaging. (**F**) Bcy1 is excluded from the nucleus independent of the carbon source. Strains were grown in YPD or YPGly at 30°C with shaking for 3 h and subsequently stained with 5 µg/mL Hoechst 33342 before imaging. (**G**) Nuclear deposition of Tpk does not induce filamentation. Cells were grown under the same conditions as for panel **B**. All genotypes are homozygous, except for Nab2, where only one allele is tagged. Scale bar, 10 µm. NLS, nuclear localization signal; mNG, mNeonGreen; GFP, green fluorescent protein.

As the Tpk subunits tolerated N-terminal GFP tags, we subsequently fused next-gen fluorescent reporter mNeonGreen (mNG) to the N-terminus of Tpk subunits, as these epitopes have higher intensity compared with GFP, which generated fully functional fusion proteins (Fig. S1) (26). This was conducted in a strain co-expressing an mScarlet (mSc)-tagged nuclear protein Nab2. When *C. albicans* was grown in YPD medium containing glucose, we observed three major distribution patterns of the Tpk isoforms: (i) Tpk was excluded from the nucleus, (ii) Tpk was enriched in the nucleus, and (iii) comparable nuclear and cytosolic distribution (Fig. 3A). To interrogate the localization patterns of each isoform more precisely, we quantified how the localization of Tpk changed over time. Both Tpk1 and Tpk2 rapidly translocated into the nucleus within 2 min of contact with glass slides under glucose-replete conditions (Fig. 3B). Although Tpk2 was quickly translocated back into the cytosol, Tpk1 translocated back into the cytoplasm with slower kinetics (Fig. 3B), highlighting that the localization pattern of each isoform is largely independent of one another. In *S. cerevisiae*, PKA subunit localization changes when grown in fermentable versus non-fermentable carbon sources (27). Therefore, we assessed the localization and abundance of *C. albicans* Tpk in the non-fermentable carbon source, glycerol. Tpk1 and Tpk2 remained mostly cytosolic and excluded from the nucleus when utilizing glycerol as the carbon source for growth throughout the entire time frame that was examined (Fig. 3C). We compared how the expression of PKA subunits changed when utilizing fermentable or non-fermentable carbon. Tpk1 expression was lower in glycerol compared with growth in glucose. On the contrary, Tpk2 and Bcy1 expressions were higher in glycerol relative to growth in glucose (Fig. 3D). Finally, given the importance of PKA in regulating filamentation, we examined Tpk localization in filament-inducing conditions. Although the three-dimensional layering of filaments made it difficult to accurately quantify Tpk distribution in various cellular compartments, we did observe that in developing hyphae, both Tpk isoforms showed similar heterogeneous distribution patterns as they did in yeast cells. This indicates that persistent Tpk2 nuclear localization is not required for morphogenesis in liquid medium (Fig. 3E).

Next, we monitored the localization of the regulatory subunit, Bcy1. Unlike Tpk subunits, Bcy1 remained exclusively in the cytosol independent of the carbon source (Fig. 3F). This led us to hypothesize that Bcy1 is actively sequestered in the cytosol, and therefore, the Tpk in the nucleus is likely the active kinase. To this end, we hypothesized if Tpk was constitutively localized to the nucleus, it would phosphorylate its downstream targets and trigger the filamentation signaling pathway in the absence of an inducing cue. To test this model, we fused a nuclear localization signal (NLS) to GFP-tagged Tpk subunits as well as Tpk subunits without the additional epitope. Although both Tpk1 and Tpk2 were constitutively localized to the nucleus, this was not sufficient to induce filamentation with any of the strains tested, highlighting there are added layers of regulation on Tpk required to induce filamentation (Fig. 3G; Fig. S2). Collectively, these results highlight unique localization patterns for each of the PKA subunits, unveil differential localization of the Tpk isoforms that is carbon source dependent, and suggest nuclear localization of Tpk is not sufficient to activate the filamentation program.

### PKA subunits localize into foci in response to environmental stresses

**Fig 4:**
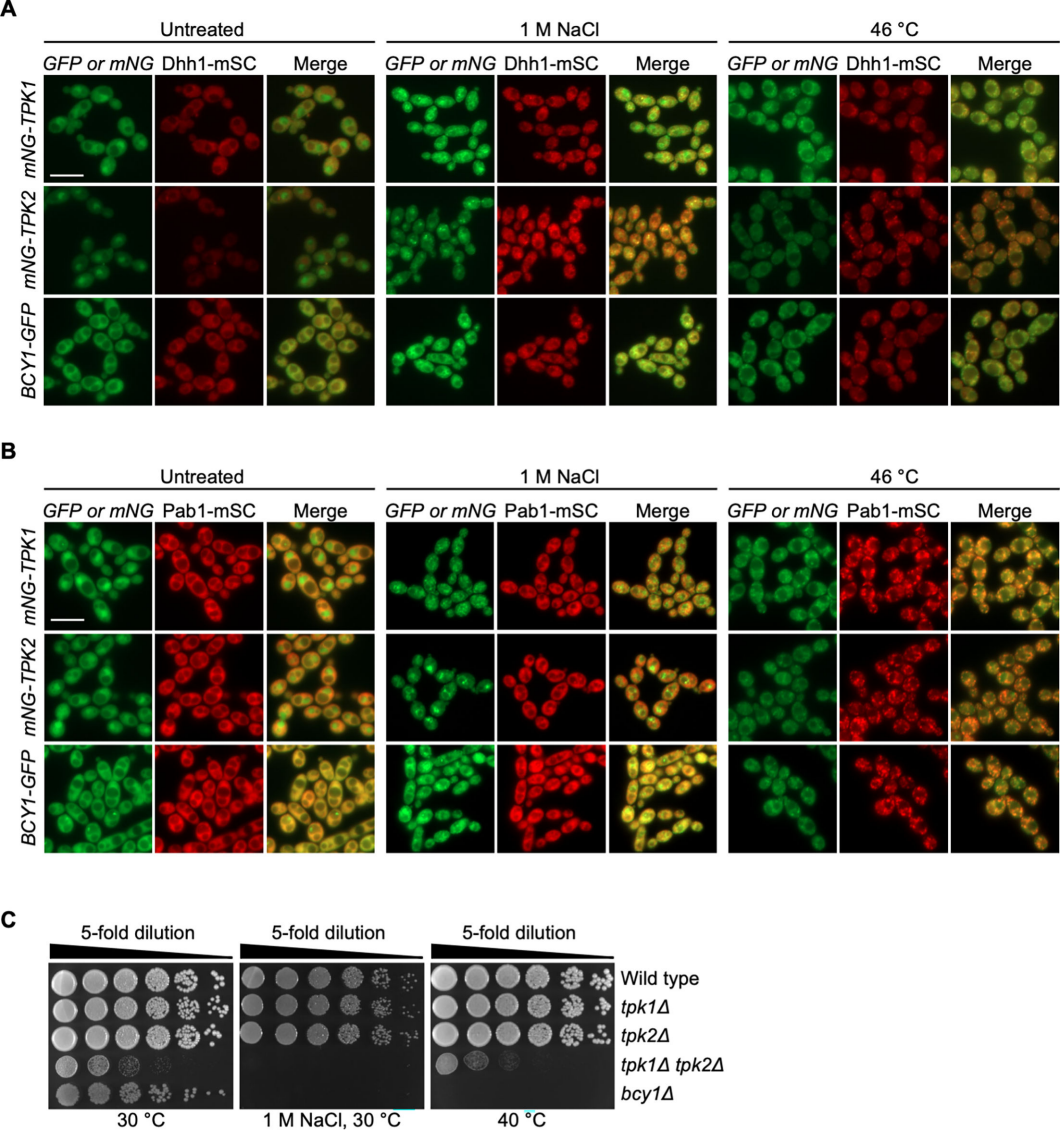
PKA subunits localize to foci in response to thermal insult or salt stress. PKA subunits are associated with the P-body marker Dhh1 (**A**) and the stress granule marker Pab1 (**B**) in response to stress at elevated temperatures. Strains were grown in YPD at 30°C with shaking for 3 h and subsequently exposed to a 10-min incubation at 46°C or with 1 M NaCl at 30°C before imaging. (**C**) PKA activity is important for stress response. Cells from overnight cultures were 5-fold serially diluted and spotted on YPD medium with or without 1 M NaCl. Agar plates were incubated at 30°C or 40°C and imaged after 48 h. Scale bar, 10 µm. GFP, green fluorescent protein; mNG, mNeonGreen; mSC, mScarlet.

Considering PKA has a wide variety of downstream targets (15), it seemed plausible that subcellular localization of PKA would change in response to environmental stress to confer spatiotemporal control of cellular signaling. In support of this idea, in *S. cerevisiae* PKA subunits have been found to be associated with P-bodies and stress granules in stationary phase, glucose deprivation, and hyperosmotic stress (20). We exposed *C. albicans* to hyperosmotic stress, with 1 M salt, or thermal insult at 46°C, and observed dotted distribution of all three PKA subunits (Fig. 4A and B). To examine if the dotted structures were associated with P-bodies, Dhh1-mSc (as a marker for P-bodies) was co-expressed with mNG-Tpk1, mNG-Tpk2, or Bcy1-GFP. P-bodies are aggregates of translationally silenced messenger ribonucleoproteins (mRNPs) and mRNA degradation machinery (28). Elevated temperatures induce the formation of P-bodies as evident from Dhh1-mSc foci. Strong association was seen between Dhh1-mSc and mNG-Tpk1, mNG-Tpk2, and Bcy1-GFP, suggesting that PKA subunits are associated with P-bodies in response to thermal insults (Fig. 4A). Stress granules are another group of RNP bodies that form under stress-induced conditions when translation is inhibited, and specific translation machinery are sequestered into these assemblies (28). To investigate if PKA also localizes to these cytoplasmic mRNP compartments, we visualized mNG-Tpk1, mNG-Tpk2, or Bcy1-GFP with Pab1-mSc as a marker for stress granules. All three PKA subunits were associated with Pab1-mSc in response to elevated temperature (Fig. 4B). Interestingly, hyperosmotic stress induced the formation of PKA foci but did not induce the formation of P-bodies or stress granules (Fig. 4A and B). This suggests that PKA can also localize to distinct cytosolic compartments during salt stress that are different from stress granules or P-bodies. Collectively, our findings highlight that PKA subunits are localized to P-bodies, stress granules, and other foci in response to external environmental stresses.

Given that PKA subunits formed distinct foci in response to environmental stress, we assessed if the specific subcellular localization was important for mediating stress tolerance. We tested homozygous deletion mutants of PKA subunits for their ability to tolerate hyperosmotic or temperature stress. Deletion of both catalytic subunits resulted in slower growth relative to wild type and hypersensitivity to both stressors, suggesting that PKA activity is important for growth and stress tolerance (Fig. 4C; Fig. S3). Surprisingly, neither of the single *tpk* mutants showed hypersensitivity to either stress, highlighting Tpk1 and Tpk2 have overlapping functions (Fig. 4C). Interestingly, the *bcy1* mutant was hypersensitive to high salt and elevated temperature, suggesting that hyperactivation of PKA is detrimental during these environmental stresses (Fig. 4C). Overall, these observations suggest that upon exposure to external stress, PKA subunits are sequestered into foci, and regulated PKA activity is important for stress response.

### Tpk2 localizes to the vacuolar membrane when utilizing an alternative carbon source

**Fig 5:**
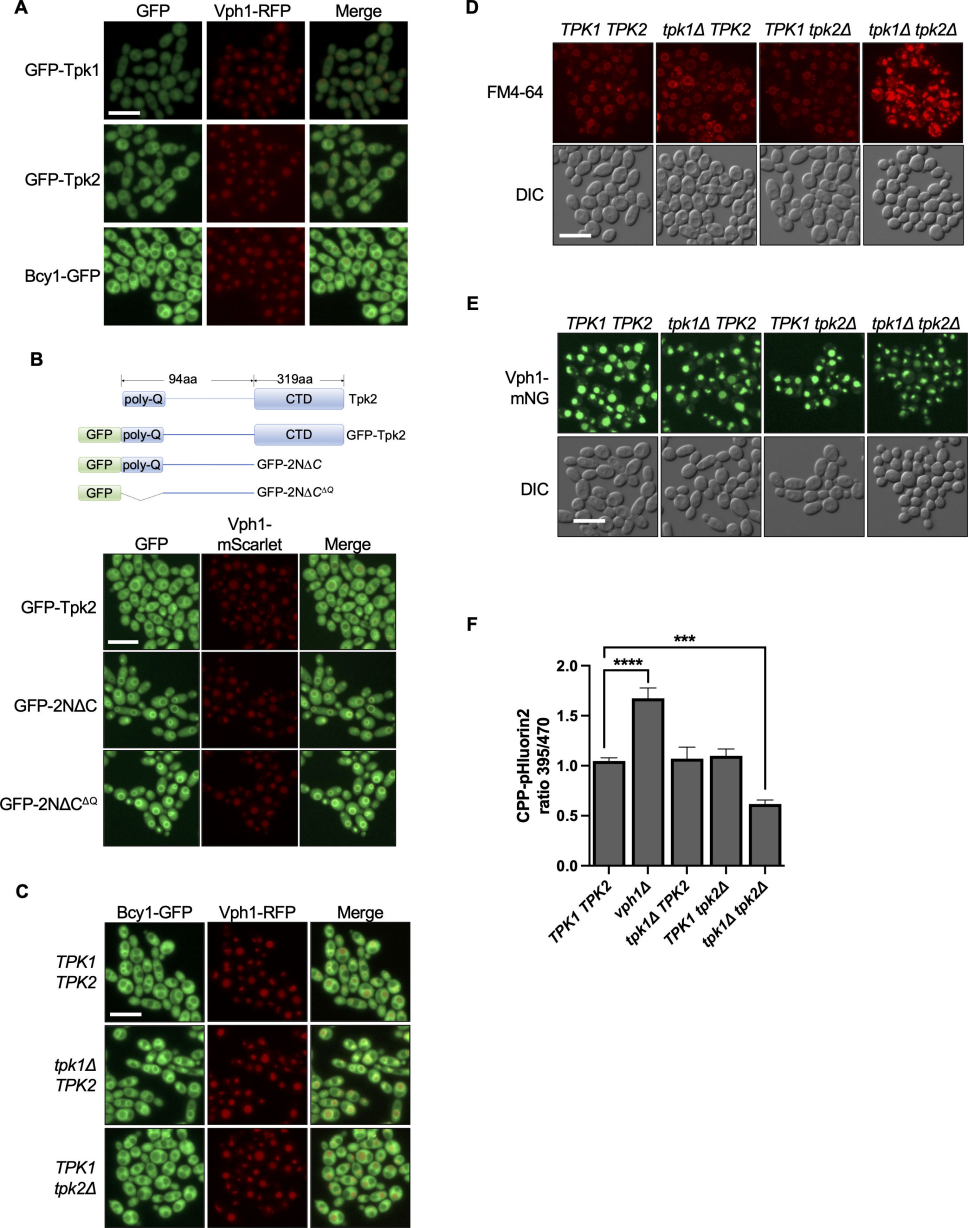
Tpk2 localizes to the vacuolar membrane in cells utilizing an alternative carbon source. (**A**) Tpk2 and Bcy1 localize to the vacuolar membrane when *C. albicans* utilize an alternative carbon source such as glycerol. Strains were grown in YPGly at 30°C with shaking for 3 h before imaging. (**B**) N-terminus of Tpk2 drives its localization to the vacuolar membrane. Schematic of Tpk2 fusion proteins (top). Strains were grown in YPGly at 30°C with shaking for 3 h before imaging. (**C**) Tpk2 is necessary for the localization of Bcy1 on the vacuolar membrane. Strains were grown in YPGly at 30°C with shaking for 3 h before imaging. (**D and E**) Double deletion of *TPK1* and *TPK2* results in fragmented vacuoles. Vacuolar morphology was assessed using membrane dye FM4-64 (**D**) or in a Vph1-mNG-expressing strain (**E**). Strains were grown in YPGly at 30°C with shaking for 3 h prior to staining or direct imaging. (**F**) Double deletion of *TPK1* and *TPK2* reduces vacuolar pH. CPP-pHluorin2-expressing strains were grown in YPGly at 30°C with shaking for 3 h. Cells were washed and resuspended in synthetic complete media with glycerol. Fluorescence intensity was then measured at a fixed wavelength of 509 nm, following excitation at 395 nm and 470 nm, by using a monochromator-based plate reader. The 395/470 ratio was calculated and plotted. One-way ANOVA; *** *P*-value < 0.005, ****P-value < 0.0001. All genotypes are homozygous, except for *VPH1*-mNG, where only one allele is tagged. Scale bar, 10 µm. GFP, green fluorescent protein; RFP, red fluorescent protein; mNG, mNeonGreen; mSC, mScarlet.

When *C. albicans* was grown in the presence of the non-fermentative carbon source glycerol, the most striking observation we initially noted was that all PKA subunits were excluded from the nucleus. Upon closer examination, we also found in these conditions Tpk2 and Bcy1, but not Tpk1, were enriched around the vacuolar membrane (Fig. 5A), as visualized using strains expressing RFP-tagged Vph1, a subunit of the vacuolar ATPase. This was particularly exciting as, to our knowledge, PKA has not been reported to localize to the vacuole periphery in *S. cerevisiae* or *C. albicans*. To explore this observation further, we constructed GFP-tagged Tpk2 fragments that included either full-length Tpk2, a truncation of the Tpk2 C-terminal domain (GFP-2NΔC), or a Tpk2 fragment lacking its N-terminal polyglutamine (PolyQ) tract (GFP-2NΔC^ΔQ^) and assessed where they localize when growing in glycerol. As with the full-length protein, Tpk2 lacking the C-terminal domain (GFP-2NΔC) or lacking the N-terminal poly PolyQ tract (GFP-2NΔC^ΔQ^) localized to the vacuolar membrane, suggesting the N-terminus of Tpk2 independent of the PolyQ tract is required for vacuolar membrane localization (Fig. 5B).

To investigate the interdependency between Tpk2 and Bcy1 for vacuolar localization, we deleted *TPK2* in the strain expressing Bcy1-GFP. Deletion of *TPK2* abolished vacuolar membrane localization of Bcy1-GFP, suggesting that Tpk2 tethers Bcy1 to the vacuolar membrane when *C. albicans* utilizes glycerol for growth (Fig. 5C). Bcy1-GFP vacuolar localization was not affected by deletion of *TPK1* (Fig. 5C).

Next, we sought to determine if the localization of Tpk2 on the vacuolar membrane, when cells were grown in non-fermentable carbon, is important for vacuolar function. We first investigated how vacuolar morphology was affected in the absence of Tpk using the lipophilic dye, FM4-64. Deletion of either *TPK* did not affect vacuolar morphology when cells were grown in glycerol; however, the absence of both catalytic subunits resulted in highly fragmented and smaller vacuoles, indicative of vacuolar dysfunction (Fig. 5D). As an independent approach, we visualized vacuolar morphology using strains expressing Vph1-mNG and confirmed that deletion of both *TPK1* and *TPK2* results in smaller and abnormal vacuoles compared with the wild type (Fig. 5E). To investigate if Tpk2 plays a role in maintaining vacuolar pH when grown in glycerol, we used strains expressing a pH-sensitive variant of GFP fused to the C-terminus of *C. albicans* carboxypeptidase Y that targets the protein to the vacuole (CPP-pHlourin2) (29). The ratio of fluorescence emission at 509 nm following excitation at 395 nm followed by 470 nm can be used as a proxy for vacuolar pH, where a higher ratio indicates more alkaline pH. As expected, deletion of the vacuolar V-ATPase-encoding gene *VPH1* increased vacuolar pH and higher absorbance ratio (Fig. 5F). Although deletion of either *TPK* alone did not alter vacuolar acidity, deletion of both *TPK1* and *TPK2* caused a lower absorbance ratio indicating hyper-acidic vacuoles. Thus, although PKA catalytic activity is important for maintaining vacuolar homeostasis and function, the relative contribution of the individual subunits is difficult to ascertain due to the functional redundancy and overlapping function of Tpk1 and Tpk2, despite the fact that only Tpk2 localizes to the vacuolar membrane.

### Alternative translation of Tpk2 is dispensable for filamentation and vacuolar localization

**Fig 6:**
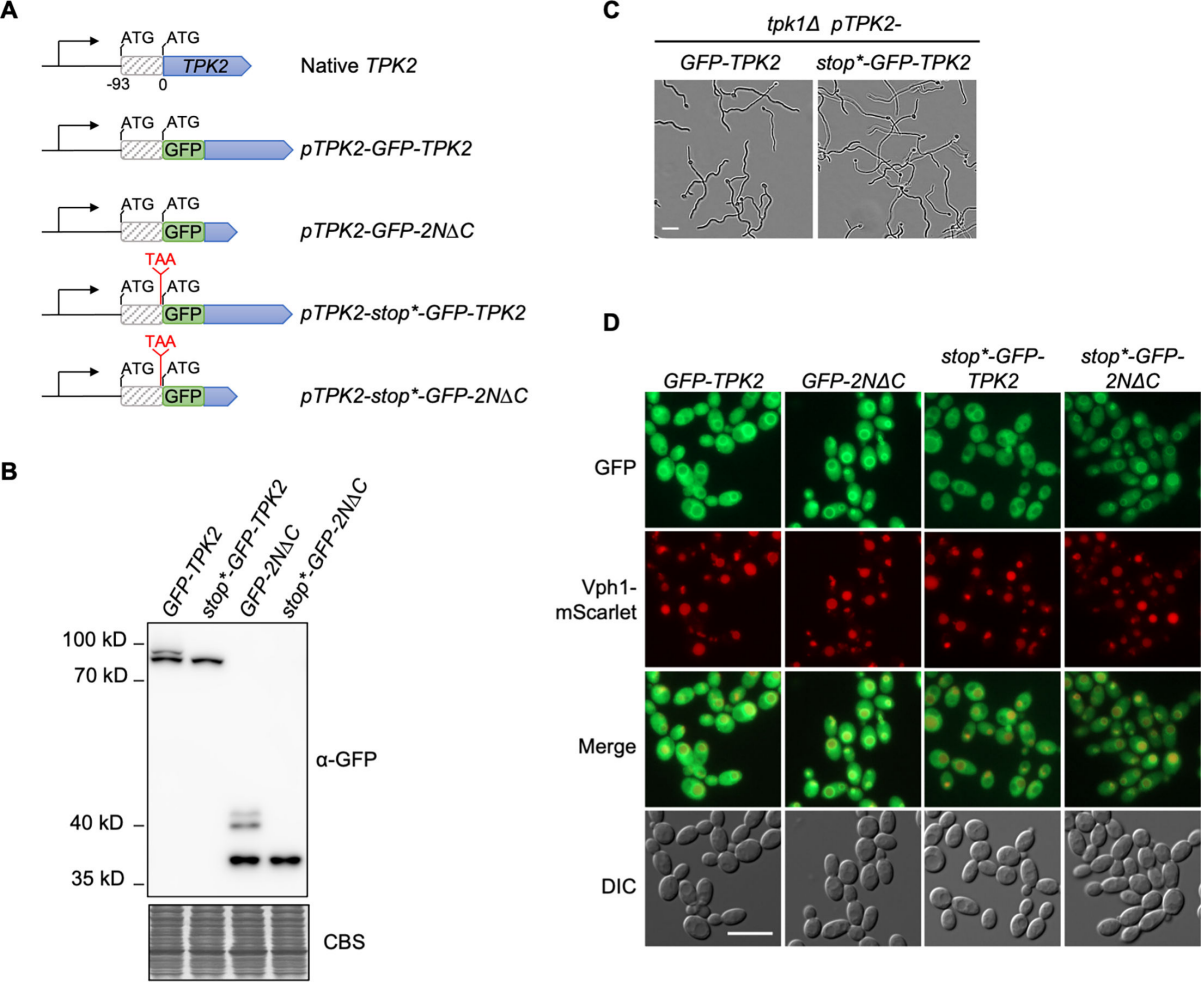
Detection of alternative translation product of Tpk2. (**A**) Schematic for *TPK2* locus and generated mutant strains. (**B**) Introducing a stop codon upstream of the canonical start codon abolishes alternatively translated Tpk2. Strains were grown for 5 h in YPGly at 30°C with shaking. Protein extracts were prepared and analyzed by western blotting. Immunoblots with an anti-GFP antibody and CBS as a loading control are shown. (**C**) Alternative translation of Tpk2 is dispensable for filamentation. Strains were grown under static conditions in the presence of 5 mM *N*-acetylglucosamine at 37°C for 4 h prior to imaging. Scale bar: 20 µm (**D**) Alternative translation of Tpk2 is dispensable for vacuolar localization. Strains were grown in YPGly medium at 30°C with shaking for 3 h before imaging. Scale bar: 10 µm. All genotypes are homozygous, except for *VPH1-*Scarlet, where only one allele is tagged. GFP, green fluorescent protein; CBS, Coomassie blue staining.

In addition to the expression and subcellular localization patterns of Tpk2 being distinct in glycerol compared with glucose (Fig. 3 and 4), we noted an unexpected high molecular weight band when visualizing GFP-Tpk2 expression from cells grown in glycerol (Fig. 3D). This finding was in agreement with previous immunoblots by Min et al. using a rabbit polyclonal antibody raised against *C. albicans* Tpk2 (30). To identify the potential alternative isoform, we analyzed the promoter region of *TPK2* and identified a cryptic start codon 93 nucleotides upstream of the annotated canonical start site (Fig. 6A). To investigate if this upstream start site was the source for the alternative isoform of Tpk2, we introduced a stop codon just upstream of the canonical start site to prevent the larger isoform from being translated (stop*-GFP-TPK2) (Fig. 6A). As expected, when the stop codon was introduced, only a single band of Tpk2 was visualized by western blot (Fig. 6B). The alternative isoform was dispensable for filamentation as the stop*-GFP-TPK2 strain was able to filament comparable with the GFP-Tpk2 strain (Fig. 6C). To investigate if the alternative isoform was required for vacuolar localization, we monitored Tpk2 localization of protein fragments with or without the alternative isoform. These protein fragments included full-length Tpk2 (stop*-GFP-TPK2), as well as the GFP-tagged N-terminus of Tpk2 (stop*-GFP-2N∆C) (Fig. 6A). In all strains examined, GFP localized to the vacuolar membrane (Fig. 6D). Overall, this shows *C. albicans* encodes and expresses an alternative isoform of Tpk2 that is dispensable for vacuolar localization and filamentation.

## DISCUSSION

PKA signaling plays a major role in controlling growth and stress responses in yeast. The catalytic subunits of PKA have highly homologous C-terminal kinase domains and yet have both distinct and redundant functions in *C. albicans* (13). Localization of these kinases to specific subcellular compartments could be important to mediate substrate specificity for each of the Tpk proteins. In this work, we generated functional GFP-tagged fusion proteins for Tpk1, Tpk2, and Bcy1 and explored the localization of all PKA subunits under distinct environmental conditions. Our findings show subcellular localization of PKA subunits is differentially regulated by carbon source. During growth in glucose, a favorable carbon source prone to activate PKA, Tpk1, and Tpk2 transiently translocate into the nucleus from the cytosol and are subsequently exported back to the cytoplasm with varying kinetics. During growth on glycerol, a less favorable carbon source, Tpk remains mostly cytosolic and excluded from the nucleus. Bcy1 is largely excluded from the nucleus under both conditions. Moreover, Tpk2 and Bcy1 localize to the vacuolar periphery when cells are grown in glycerol, suggesting that although the PKA holoenzyme is mainly cytosolic, a sub-population of Tpk2-containing holoenzyme can be targeted to specific organelles. Furthermore, we showed PKA subunits localize to P-bodies, stress granules, and other foci in response to environmental stresses. Overall, our work provides the first detailed analysis of the subcellular localization of all PKA subunits in *C. albicans* and provides extensive resources to study *C. albicans* PKA for the research community.

Contact sensing plays important roles in fungi for diverse processes from cellular differentiation to virulence. In *C. albicans,* hyphae exhibit thigmotropism, the ability to change the direction of growth when encountering irregular topographies (31, 32). Mechanosensitive (MS) ion channels are involved in governing thigmotropism (32). Contact of the hyphal tip with ridges results in membrane stretch and activation of MS channels, culminating in the reorientation of the growth axis (33). This ability may help the fungus grow toward the points of entry in the epithelia and endothelia. Additionally, invasive hyphal growth and biofilm formation are also contact-dependent processes. The mitogen-activated protein kinase, Mkc1, signals to induce invasive hyphal growth and biofilm formation in response to niche-specific contact (34). Moreover, mechanical compression can also alter cell morphology during the invasive growth of *C. albicans* (35). The role of PKA in sensing or responding to contact has not been previously explored in *C. albicans*. In this work, we show that Tpk1 and Tpk2 rapidly translocate into the nucleus from the cytoplasm upon contact with glass slides when grown in the presence of glucose (Fig. 3). This phenotype was dependent on growth conditions, as Tpk was not imported in the nucleus during growth in glycerol. It is possible that contact-sensing induces a surge in cAMP production in a glucose-dependent manner, causing the release of Tpk from the holoenzyme in the cytoplasm and subsequent nuclear import. Interestingly, there is precedence of PKA playing roles in contact-sensing in mammalian systems. PIEZO2 is a MS channel that enables cells to detect and respond to mechanical stimuli (36). PKA activity modulates PIEZO2, such that cAMP agonists induce large PIEZO2-mediated currents and PKA inhibitors dampen mechanotransduction (37, 38).

PKA subunits show nucleo-cytoplasmic distribution in *S. pombe* and *S. cerevisiae*. Similarly, in *C. albicans*, we observed that PKA subunits mostly displayed nucleo-cytoplasmic localization. However, in this work, we show Tpk2 and Bcy1 localized to the vacuolar periphery when cells utilized non-fermentable carbon for growth (Fig. 5). To our knowledge, this novel localization had never been observed in any fungal species. In *S. pombe,* the sole catalytic subunit of PKA, Pka1, localizes inside vacuoles under glucose-starved conditions. It was postulated that Pka1 was targeted to the vacuole for degradation, since PKA signaling is less required during starvation (39). This did not appear to be the case in *C. albicans*, as the holoenzyme containing Tpk2 and Bcy1 was enriched around the vacuolar membrane and not inside the vacuole. We propose that Tpk2 containing inactive holoenzyme is concentrated around the vacuolar membrane to keep it poised for activation when cAMP-PKA signaling is locally required to phosphorylate substrates around the vacuole. This idea is supported by observations in *S. cerevisiae* where the cAMP-PKA pathway plays important roles in glucose-dependent V-ATPase assembly (40). Additionally, V-ATPase assembly is regulated by cytosolic pH in yeast, and functional V-ATPase is required for PKA activation in glucose (41). Also in kidney cells, PKA regulates plasma membrane V-ATPase subcellular localization and activity (42, 43). Phosphoproteomic studies in *C. albicans* have also implicated vacuolar transporters, Zrt3 and Avt4, as substrates of PKA (15). These observations suggest potential functional connections between PKA and the vacuole in *C. albicans,* despite the fact that we were unable to detect differences in vacuolar integrity or vacuolar pH when only *TPK2* was deleted. Due to the functional redundancy of Tpk1 and Tpk2, Tpk1 and/or other kinases could potentially compensate for the lack of Tpk2 in maintaining vacuolar homeostasis. Additionally, it remains to be determined if Tpk2 regulates other pathways that require vacuolar function. Interestingly, we found deletion of both PKA catalytic subunits decreased vacuolar pH, further establishing connections between PKA and the vacuole (Fig. 5F). Vacuolar acidification is important for diverse cellular processes including protein degradation, autophagy, calcium and metal homeostasis, storage of basic amino acids and polyphosphates, cargo sorting at the Golgi, and also drug resistance (44–46). Thus, although the role of PKA in regulating vacuolar pH remains elusive, our work paves the road for further exploration into the connection between *C. albicans* PKA and vacuolar functions. Also, in mammalian cells, PKA subunits are targeted to subcellar compartments through interaction with specific anchor proteins (16–18). However, similar anchor proteins have not been identified in any fungal species to date. Our findings suggest for the first time the possibility of the existence of an anchoring protein for Tpk2 on the vacuolar periphery in *C. albicans*.

cAMP-PKA signaling is critical for growth and metabolism in response to diverse environmental conditions. In *S. cerevisiae*, PKA activity is stimulated in response to the addition of glucose to cells grown in a non-fermentable carbon source or to stationary phase cells, and PKA signaling is dampened in stationary phase and nutrient starvation (47, 48). Downregulation of PKA signaling is important for the adaptation to stress and nutrient starvation (49, 50). P-bodies are required for the long-term survival of quiescent cells, and inactivation of PKA signaling drives the formation of P-bodies (51). Here, we showed PKA subunits localize into foci, including P-bodies and stress granules, in response to both hyperosmotic stress and heat stress (Fig. 4). Additionally, we show that hyperactivation of PKA was deleterious when responding to external stresses (Fig. 4). This leads us to suggest that PKA subunits are localized into foci to downregulate PKA activity and finetune PKA signaling to adapt to these different environmental stressors. Additionally, although PKA signaling is indispensable for filamentous growth *in vitro*, mutants lacking both catalytic subunits of PKA can still undergo filamentation in mouse models of infection, suggesting that cAMP-PKA signaling is not essential for filamentous growth *in vivo* (52). It remains to be explored where PKA subunits localize in filaments *in vivo* and if that contributes to the tuning of PKA signaling. Until now, studies exploring PKA subcellular localization have been very limited in *C. albicans*. However, the reagents constructed in this study will undoubtedly be key resources for the field to explore the localization and role of PKA in different facets of *C. albicans* growth and virulence.

## MATERIALS AND METHODS

### Culture conditions

Strains were grown in YP (1% yeast extract, 2% peptone) medium supplemented with 2% glucose (YPD) or 3% glycerol (YPGly) at 30°C. For solid medium, 2% agar was added. All strains were stored in 25% glycerol in YPD medium at −80°C.

### Strain and plasmid construction

All strains used in this study including genotypes are listed in Table S1. All plasmids used in this study are listed in Table S2. All oligonucleotides used in this study are listed in Table S3. Full sequence of plasmids carrying the GFP-Tpk variants is provided in Text S1. Strain construction details are also provided in Text S1.

### Western blot

Crude cell lysates were prepared using a TCA-precipitation-based method as described previously (53). Protein contents were assessed by analyzing lysates using homemade 10% stain-free gels (regular SDS gel with 0.5% (vol/vol) 2,2,2-tricholoroethanol added to the resolving layer). After calibration, comparable amounts of protein from each sample were resolved on a 10% Novex Tris-glycine gel (Invitrogen), transferred to the polyvinylidene difluoride (PVDF) membrane, and probed by a mouse anti-GFP antibody (Roche; 11814460001). After ECL development, membranes were stained briefly with Coomassie blue to confirm comparable loading.

### Microscopy

Overnight cultures grown in YP medium were diluted to an OD_600_ of 0.2 in fresh YP medium and grown at 30°C for 3 h with shaking at 200 rpm. After 3 h, the cells were washed once with YNB medium (0.171% YNB-folic acid-riboflavin powder [Sunrise Science, #1535-250], 0.192% Dropout mix without uracil [Bioshop, #DOM007], 0.008% uridine, 0.1% monosodium glutamate, 2% glucose or 3% glycerol) and resuspended in the same medium before imaging. For nuclear staining, 5 µg/mL Hoechst 33342 was added to the outgrowth cultures for 10 min with shaking at 200 rpm in the dark prior to washing with YNB medium and imaging. Cells were imaged using a Zeiss Axio Observer.M1 upright microscope at 40× magnification. Fluorescence was imaged using an X-Cite series 120 light source and ET green fluorescent protein (GFP), ET HQ tetramethylrhodamine isothiocyanate (TRITC)/DsRED, and 4′,6-diamidino-2-phenylindole (DAPI) filter sets.

For visualizing stress granules and P-bodies, strains were grown in YPD and subsequently washed and resuspended in YNB-glucose medium as described above. Strains were challenged with heat shock at 46°C or 1 M NaCl at 30°C for 10 min prior to imaging. Cells were imaged using a Zeiss Axio Observer.Z1 inverted microscope at 40× magnification. Fluorescence was imaged using an X-Cite series 120 light source and ET green fluorescent protein (GFP) and ET HQ tetramethylrhodamine isothiocyanate (TRITC)/DsRED filter sets.

### FM4-64 staining

Overnight cultures grown in YPG medium were diluted to an OD_600_ of 0.2–0.5 in fresh medium and grown at 30°C for 3 h with shaking at 200 rpm. Cells were stained with 8 µM FM4-64 (Thermo Fisher Scientific, # T3166) in the dark for 30 min at 30°C with shaking. After 30 min, the cells were resuspended in 1 mL YPG medium to remove free FM4-64 and added to 4 mL of fresh YPG medium. Cells were incubated in FM4-64 free medium for 90 min at 30°C, spun down, and resuspended in 1× PBS. Cells were imaged using a Zeiss Axio Observer.M1 at 40× magnification. FM4-64 staining was observed using an X-Cite series 120 light source and an ET HQ tetramethylrhodamine isothiocyanate (TRITC)/DsRED filter set.

### IncuCyte imaging

To monitor *C. albicans* filamentation, overnight cultures were diluted to an OD_600_ of ~0.002 into a specific filament-inducing medium (0.67% yeast nitrogen base with ammonium sulfate and without amino acids, 2% casamino acids, 0.2% glucose, and 5 mM N-acetylglucosamine) and incubated under static growth conditions at 37°C for 4–5 h. Images were captured using the IncuCyte S3 Live-Cell Analysis System (Sartorius) using 20× magnification.

### Spot dilution assay

Overnight cultures were washed once with sterile water and diluted to an OD_600_ of 0.2. Cells were then serially diluted 5-fold in water. Five microliters of the dilutions was spotted on agar plates. Plates were incubated at 30°C or 37°C for 1–2 days and imaged using ChemiDoc.

### Vacuolar acidity assay

Overnight cultures grown in YPG medium were diluted to an OD_600_ of 0.4–0.8 in fresh medium and grown at 30°C for 3 h with shaking at 200 rpm. Cells were washed twice in YNB-glycerol medium and subsequently resuspended in the same medium to an OD_600_ of 0.5. Cell suspension (100 µL) was transferred to a black 96-well plate (Grenier bio #655076). Relative fluoresce units (RFU) were measured using emission at 509 nm (20 nm bandwidth) following excitation wavelengths of 395 nm and 470 nm (9 nm bandwidth for both) using an Infinite 200 PRO reader (Tecan). Wild-type cells were used as controls for background fluorescence. Background fluorescence at 395 nm and 470 nm was subtracted from the RFU obtained from cells expressing pHluorin2, and the ratio of absorbance was calculated. Statistical significance was determined using one-way ANOVA using GraphPad Prism 8.

## SUPPLEMENTAL MATERIAL

Supplemental material msphere.01037-24-s0001.pdf Supplemental figures, tables, methods, and references.

ASM does not own the copyrights to Supplemental Material that may be linked to, or accessed through, an article. The authors have granted ASM a non-exclusive, world-wide license to publish the Supplemental Material files. Please contact the corresponding author directly for reuse.
