# Supplementary material for: Exploring the differential localization of protein kinase A isoforms in Candida albicans
Source: mSphere. 2025 Feb 25;10(3):e01037-24. doi: 10.1128/msphere.01037-24 (PMC11934313; doi:10.1128/msphere.01037-24)
Supplement: Supplemental material — Supplemental figures, tables, methods, and references. [file msphere.01037-24-s0001.pdf]

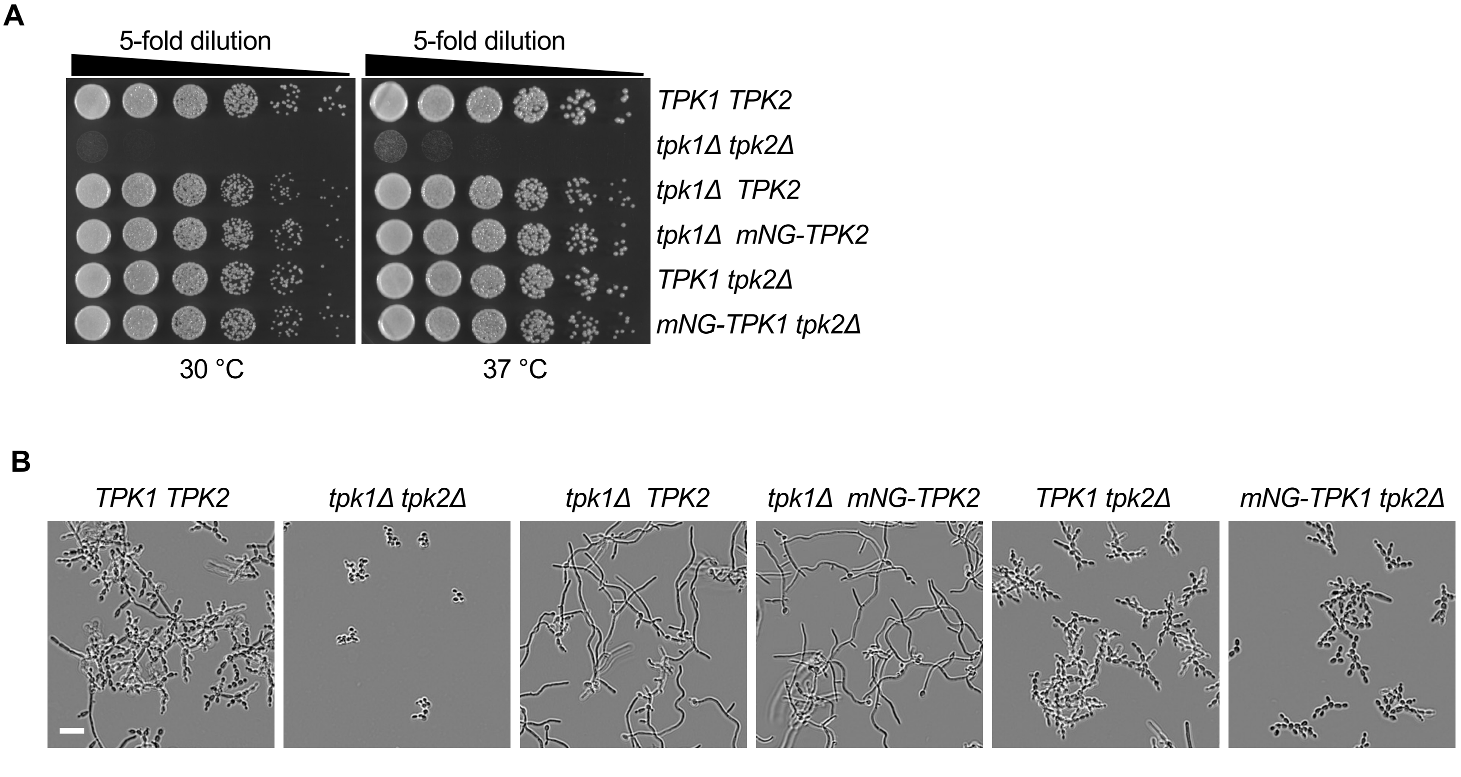


**Figure S1. Generation of functional mNeonGreen-tagged catalytic subunits of PKA. (A)** N-terminal mNeonGreen tagged Tpk1 supports growth comparable to wild-type Tpk1 on solid medium. Cells from overnight cultures were 5-fold serially diluted and spotted on YPD medium. Agar plates were incubated at 30 °C (left) or 37 °C (right) and imaged after 24 hours. (**B**) N-terminal mNeonGreen tagged Tpk2 supports filamentation in liquid medium. Strains were grown under static conditions in the presence of 5 mM *N*-acetylglucosamine at 37 °C for 5 hours prior to imaging. All genotypes are homozygous. Scale bar, 20 µm; mNG, mNeonGreen.

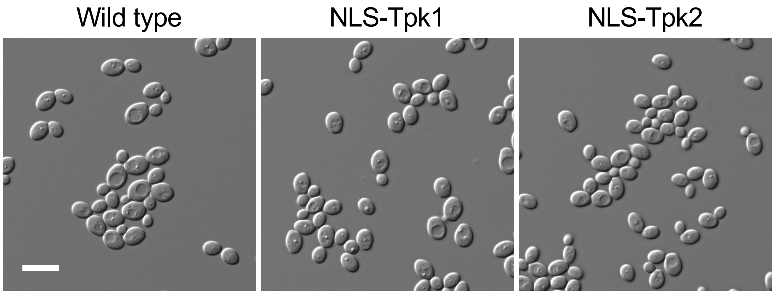


**Figure S2. Nuclear deposition of Tpk does not induce filamentation.** Strains expressing NLS-Tpk lacking any GFP tag were grown overnight under shaking conditions in YPD medium at 30 °C prior to imaging**.** Scale bar, 10 μm.

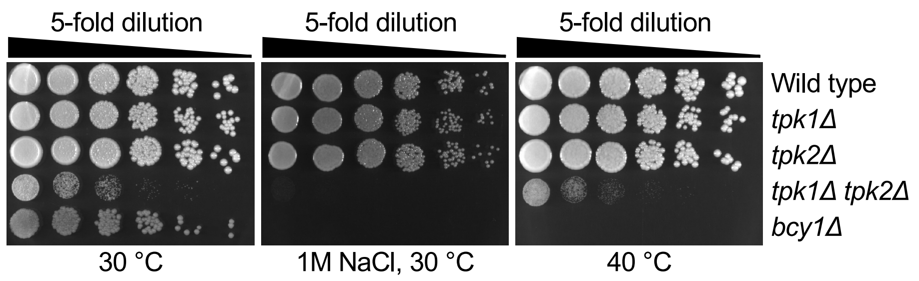


**Figure S3. PKA activity is important for stress response.** Cells from overnight cultures were 5-fold serially diluted and spotted on YPD medium with or without 1M NaCl. Agar plates were incubated at 30 °C or 40 °C and imaged after 48 hours. This is an independent biological replicate of the experiment shown in Figure 4C.

**Table S1: Strains used in this study**

| Strain Name | Alias | Genotype | Source |
|---|---|---|---|
| CaLC155 | SC5314 | Clinical isolate | (1) |
| CaLC239 | SN95 Wild type | *arg/arg4 his1/his1 URA3/ura3::imm$^{434}$ IRO1/iro1::imm$^{434}$* | (2) |
| CaLC6118 | SN95 *CPP-Phluorin* | As CaLC239 *neut5l::CPP-PHluorin2-SAT1/NEUT5L* | This Study |
| CaLC6489 | SN95 *bcy1Δ* | As CaLC239 *bcy1::HygB/bcy1::HygB* | This Study |
| CaLC6699 | SN95 *tpk2Δ* | As CaLC239 *tpk1::ARG4/tpk1::ARG4* | |
| CaLC6702 | SN95 *tpk2Δ* | As CaLC239 *tpk2::ARG4/tpk2::ARG4* | This Study |
| CaLC6818 | SN95 *GFP-TPK1* | As CaLC239 *tpk1::pTPK1-GFP-TPK1-HIS1/tpk1::pTPK1-GFP-TPK1-SAT1* | This Study |
| CaLC6826 | SN95 *GFP-TPK2* | As CaLC239 *tpk2::pTPK2-GFP-TPK2-HIS1/tpk2::pTPK2-GFP-TPK2-SAT1* | This Study |
| CaLC6836 | SN95 *tpk1Δ tpk2Δ* | As CaLC239 *tpk1::HygB/tpk1::HygB tpk2::HygB/tpk2::HygB* | This Study |
| CaLC6852 | SN95 *tpk2Δ GFP-TPK1* | As CaLC239 *tpk1::pTPK1-GFP-TPK1-HIS1/tpk1::pTPK1-GFP-TPK1-SAT1 tpk2::ARG4/tpk2::ARG4* | This Study |
| CaLC6860 | SN95 *tpk1Δ GFP-TPK2* | As CaLC239 *tpk2::pTPK2-GFP-TPK2-HIS1/tpk2::pTPK2-GFP-TPK2-SAT1 tpk1::ARG4/tpk1::ARG4* | This Study |
| CaLC6978 | SN95 *BCY1-GFP* | As CaLC239 *BCY1-GFP-HIS1/BCY1-GFP-HIS1* | This Study |
| CaLC6985 | SN95 *GFP-TPK1 VPH1-RFP* | As CaLC239 *tpk1::pTPK1-GFP-TPK1-HIS1/tpk1::pTPK1-GFP-TPK1-SAT1 VPH1/VPH1-RFP::HygB* | This Study |
| CaLC6987 | SN95 *GFP-TPK2 VPH1-RFP* | As CaLC239 *tpk2::pTPK2-GFP-TPK2-HIS1/tpk2::pTPK2-GFP-TPK2-SAT1 VPH1/VPH1-RFP::HygB* | This Study |
| CaLC6993 | SN95 *BCY1-GFP VPH1-RFP* | As CaLC239 *BCY1-GFP-HIS1/BCY1-GFP-HIS1 VPH1/VPH1-RFP::HygB* | This Study |
| CaLC7700 | SN95 *BCY1-GFP VPH1-RFP tpk1Δ* | As CaLC239 *BCY1-GFP-HIS1/BCY1-GFP-HIS1 VPH1/VPH1-RFP::HygB tpk1::SAT1/tpk1::SAT1* | This Study |
| CaLC7702 | SN95 *BCY1-GFP VPH1-RFP tpk2Δ* | As CaLC239 *BCY1-GFP-HIS1/BCY1-GFP-HIS1 VPH1/VPH1-RFP::HygB tpk2::SAT1/tpk2::SAT1* | This Study |
| CaLC7878 | SN95 *NLS-GFP-TPK1* | As CaLC239 *tpk1::pTPK1-NLS-GFP-TPK1-HIS1/tpk1::pTPK1-NLS-GFP-TPK1-SAT1* | This Study |
| CaLC7884 | SN95 *NLS-GFP-TPK2* | As CaLC239 *tpk2::pTPK2-NLS-GFP-TPK2-HIS1/tpk2::pTPK2-NLS-GFP-TPK2-SAT1* | This Study |
| CaLC8597 | SC5314 *tpk1Δ* | As SC5314 *tpk1::SAT1/tpk1::SAT1* | This Study |
| CaLC8599 | SC5314 *tpk2Δ* | As SC5314 *tpk2::HygB/tpk2::HygB* | This Study |
| CaLC8601 | SC5314 *tpk1Δ tpk2Δ* | As SC5314 *tpk1::SAT1/tpk1::SAT1 tpk2::HygB/tpk2::HygB* | This Study |

| | | | |
|---|---|---|---|
| CaLC8867 | SN95 *tpk1Δ pTPK1-GFP-2N1C* | As CaLC239 *tpk1::pTPK1-GFP-(TPK2N+TPK1C)-HIS1/tpk1::pTPK1-GFP-(TPK2N+TPK1C)-SAT1* | This Study |
| CaLC8869 | SN95 *tpk1Δ pTPK1-GFP-TPK2* | As CaLC239 *tpk1::pTPK1-GFP-TPK2-HIS1/tpk1::pTPK1-GFP-TPK2-SAT1* | This Study |
| CaLC8873 | SN95 *tpk1Δ pTPK1-GFP-1N2C* | As CaLC239 *tpk1::pTPK1-GFP-(TPK1N+TPK2C)-HIS1/tpk1::pTPK1-GFP-(TPK1N+TPK2C)-SAT1* | This Study |
| CaLC8946 | SN95 *VPH1-mNG* | As CaLC239 *VPH1/VPH1-mNG::HygB* | This Study |
| CaLC8948 | SN95 *VPH1-mNG tpk1Δ* | As CaLC239 *VPH1/VPH1-mNG::HygB tpk1::ARG4/tpk1::ARG4* | This Study |
| CaLC8950 | SN95 *VPH1-mNG tpk2Δ* | As CaLC239 *VPH1/VPH1-mNG::HygB tpk2::ARG4/tpk2::ARG4* | This Study |
| CaLC8966 | SN95 *tpk1Δ pTPK2-GFP-1N2C* | As CaLC239 *tpk2::pTPK2-GFP-(TPK1N+TPK2C)-HIS1/tpk2::pTPK2-GFP-(TPK1N+TPK2C)-SAT1 tpk1::HygB/tpk1::HygB* | This Study |
| CaLC8968 | SN95 *tpk1Δ pTPK2-GFP-TPK1* | As CaLC239 *tpk2::pTPK2-GFP-TPK1-HIS1/tpk2::pTPK2-GFP-TPK1-SAT1 tpk1::HygB/tpk1::HygB* | This Study |
| CaLC8972 | SN95 *tpk1Δ pTPK2-GFP-2N1C* | As CaLC239 *tpk2::pTPK2-GFP-(TPK2N+TPK1C)-HIS1/tpk2::pTPK2-GFP-(TPK2N+TPK1C)-SAT1 tpk1::HygB/tpk1::HygB* | This Study |
| CaLC8995 | SN95 *tpk1Δ TPK2-GFP* | As CaLC239 *tpk1::ARG4/tpk1::ARG4 pTPK2-TPK2-GFP-HygB/pTPK2-TPK2-GFP-HygB* | This Study |
| CaLC8997 | SN95 *tpk2Δ TPK1-GFP* | As CaLC239 *tpk2::ARG/tpk2::ARG pTPK1-TPK1-GFP-HygB/pTPK1-TPK1-GFP-HygB* | This Study |
| CaLC9013 | SN95 *mNG-TPK1* | As CaLC239 *tpk1::pTPK1-mNG-TPK1-HIS1/tpk1::pTPK1-mNG-TPK1-SAT1* | This Study |
| CaLC9015 | SN95 *mNG-TPK2* | As CaLC239 *tpk2::pTPK2-mNG-TPK2-HIS1/tpk1::pTPK2-mNG-TPK2-SAT1* | This Study |
| CaLC9049 | SN95 p*TPK2-GFP-TPK2 VPH1-mSC* | As CaLC239 *tpk2::pTPK2-GFP-TPK2-HIS1/tpk2::pTPK2-GFP-TPK2-HIS1 VPH1-mSC-HygB/VPH1* | This Study |
| CaLC9051 | SN95 *pTPK2-GFP-TPK2NΔC VPH1-mSC* | As CaLC239 *tpk2::pTPK2-GFP-TPK2NΔC-HIS1/tpk2::pTPK2-GFP-TPK2NΔC-HIS1 VPH1-mSC-HygB/VPH1* | This Study |
| CaLC9053 | SN95 *pTPK2-STOP-GFP-TPK2 VPH1-mSC* | As CaLC239 *tpk2::pTPK2-STOP-GFP-TPK2-HIS1/tpk2::pTPK2-STOP-GFP-TPK2-HIS1 VPH1-mSC-HygB/VPH1* | This Study |
| CaLC9055 | SN95 *pTPK2-STOP-GFP-TPK2NΔC VPH1-mSC* | As CaLC239 *tpk2::pTPK2-STOP-GFP-TPK2NΔC-HIS1/tpk2::pTPK2-STOP-GFP-TPK2NΔC-HIS1 VPH1-mSC-HygB/VPH1* | This Study |

| | | | |
|---|---|---|---|
| CaLC9057 | SN95 *GFP-2NΔC$^{ΔQ}$ VPH1-mSC* | As CaLC239 *tpk2::pTPK2-GFP-TPK2NΔC$^{ΔQ}$ -HIS1/tpk2::pTPK2-GFP-TPK2NΔC$^{ΔQ}$ -HIS1 VPH1/VPH1-mSC::HygB* | This Study |
| CaLC9063 | SN95 *pTPK2-STOP-GFP-TPK2 VPH1-mSC tpk1Δ* | As CaLC239 *tpk2::pTPK2-STOP-GFP-TPK2-HIS1/tpk2::pTPK2-STOP-GFP-TPK2-HIS1 VPH1-mSC-HygB/VPH1 tpk1::NAT/tpk1::NAT* | This Study |
| CaLC9103 | SN95 *CPP-PHluorin vph1Δ* | As CaLC239 *neut5l::CPP-PHluorin2-SAT1/NEUT5L vph1::HygB/vph1::HygB* | This Study |
| CaLC9154 | SN95 *CPP-PHluorin tpk1Δ* | As CaLC239 *neut5l::CPP-PHluorin2-SAT1/NEUT5L tpk1::HygB/tpk1::HygB* | This Study |
| CaLC9156 | SN95 *CPP-PHluorin tpk2Δ* | As CaLC239 *neut5l::CPP-PHluorin2-SAT1/NEUT5L tpk2::HygB/tpk2::HygB* | This Study |
| CaLC9158 | SN95 *CPP-PHluorin tpk1Δ tpk2Δ* | As CaLC239 *neut5l::CPP-PHluorin2-SAT1/NEUT5L tpk1::HygB/tpk1::HygB tpk2::HygB/tpk2::HygB* | This Study |
| CaLC9458 | SN95 *mNG-TPK1 NAB2-mSC* | As CaLC239 *tpk1::pTPK1-mNG-TPK1-HIS1/tpk1::pTPK1-mNG-TPK1-SAT1 NAB2/NAB2-mSc-HygB* | This Study |
| CaLC9460 | SN95 *mNG-TPK2 NAB2-mSC* | As CaLC239 *tpk2::pTPK2-mNG-TPK2-HIS1/tpk1::pTPK2-mNG-TPK2-SAT1 NAB2/NAB2-mSc-HygB* | This Study |
| CaLC9478 | SN95 *VPH1-mNG tpk1Δ tpk2Δ* | As CaLC239 *VPH1/VPH1-mNG::HygB tpk1::SAT1/tpk1::SAT1 tpk2::SAT1/tpk2::SAT1* | This Study |
| CaLC10330 | SN95 *mNG-TPK1 PAB1-mSC* | As CaLC239 *tpk1::mNG-TPK1-HIS1/tpk1::mNG-TPK1-SAT1 PAB1/PAB1-mSC::ARG4* | This Study |
| CaLC10332 | SN95 *mNG-TPK2 PAB1-mSC* | As CaLC239 *tpk2::mNG-TPK2-HIS1/tpk1::mNG-TPK2-SAT1 PAB1/PAB1-mSC::ARG4* | This Study |
| CaLC10334 | SN95 *BCY1-GFP PAB1-mSC* | As CaLC239 *BCY1-GFP-HIS1/BCY1-GFP-HIS1 PAB1/PAB1-mSC::ARG4* | This Study |
| CaLC10371 | SN95 *mNG-TPK1 DHH1-mSC* | As CaLC239 *tpk1::mNG-TPK1-HIS1/tpk1::mNG-TPK1-SAT1 DHH1-mSC::ARG4/DHH1-mSC::HygB* | This Study |
| CaLC10373 | SN95 *mNG-TPK2 DHH1-mSC* | As CaLC239 *tpk2::mNG-TPK2-HIS1/tpk1::mNG-TPK2-SAT1 DHH1-mSC::ARG4/DHH1-mSC::HygB* | This Study |
| CaLC10375 | SN95 *BCY1-GFP DHH1-mSC* | As CaLC239 *BCY1-GFP-HIS1/BCY1-GFP-HIS1 DHH1-mSC::ARG4/DHH1-mSC::HygB* | This Study |
| CaLC10445 | SN95 *mNG-TPK1 tpk2Δ* | As CaLC239 *tpk1::pTPK1-mNG-TPK1-HIS1/tpk1::pTPK1-mNG-TPK1-SAT1 tpk2::HygB/tpk2::HygB* | This Study |

| | | | |
|---|---|---|---|
| CaLC10447 | SN95 *mNG-TPK2 tpk1Δ* | As CaLC239 *tpk2::pTPK2-mNG-TPK2-HIS1/tpk1::pTPK2-mNG-TPK2-SAT1 tpk1::HygB/tpk1::HygB* | This Study |
| CaLC10572 | SN95 *NLS-TPK1* | As CaLC239 *tpk1::pTPK1-NLS-TPK1-HIS1/tpk1::pTPK1-NLS-TPK1-HIS1* | This Study |
| CaLC10575 | SN95 *NLS-TPK2* | As CaLC239 *tpk2::pTPK2-NLS-TPK2-HIS1/tpk2::pTPK2-NLS-TPK2-HIS1* | This Study |

**Table S2: Plasmids used in this study**

| Plasmid ID | Description | Source |
|---|---|---|
| pLC963 | pV1393-1 (CaCas9/sgRNA entry expression vector, contains NatR gene, targeting *NEUT5L*) | (3) |
| pLC1081 | CAS9 vector pV1093 | (4, 5) |
| pLC1082 | pNAT | (4) |
| pLC1087 | pFA-3HA-SAT1 | (6) |
| pLC1100 | pFA-3HA-ARG4 | (6) |
| pLC1181 | pDUP3-CPP-PHL2 | (7). |
| pLC1206 | pFA-GFP-HIS1 | This study |
| pLC1211 | pFA-3HA-HygB | This study |
| pLC1214 | pFA-GFP-HygB | This study |
| pLC1215 | pFA-RFP-HygB | This study |
| pLC1372 | pFA-GFP-TPK1-HIS1-TPK1 3' | This study |
| pLC1375 | pFA-GFP-TPK2-HIS1-TPK2 3' | This study |
| pLC1402 | pFA-GFP-TPK1-SAT1-TPK1 3' | This study |
| pLC1406 | pFA-GFP-TPK2-SAT1-TPK1 3' | This study |
| pLC1527 | pFA-NLS-GFP-TPK1-HIS1 | This study |
| pLC1528 | pFA-NLS-GFP-TPK2-HIS1 | This study |
| pLC1529 | pFA-NLS-GFP-TPK1-SAT1 | This study |
| pLC1530 | pFA-NLS-GFP-TPK2-SAT1 | This study |
| pLC1668 | pFA-pTpk1-GFP-2N1C-HIS1 | This study |
| pLC1669 | pFA-pTpk1-GFP-2FL-HIS1 | This study |
| pLC1671 | pFA-pTpk2-GFP-1N2C-HIS1 | This study |
| pLC1672 | pFA-pTpk2-GFP-1FL-HIS1 | This study |
| pLC1673 | pFA-pTpk2-GFP-2N-HIS11 | This study |
| pLC1676 | pFA-pTpk1-GFP-1N2C-HIS1 | This study |
| pLC1677 | pFA-pTpk2-GFP-2N1C-HIS | This study |
| pLC1682 | pFA-pTpk1-GFP-2N1C-SAT1 | This study |
| pLC1683 | pFA-pTpk1-GFP-2FL-SAT1 | This study |
| pLC1685 | pFA-pTpk1-GFP-1N2C-SAT1 | This study |
| pLC1686 | pFA-pTpk2-GFP-1N2C-SAT1 | This study |
| pLC1687 | pFA-pTpk2-GFP-1FL-SAT1 | This study |
| pLC1689 | pFA-pTpk2-GFP-2N1C-SAT1 | This study |
| pLC1693 | pFA-mScarlet-ARG4 | This study |
| pLC1696 | pFA-NeonGreen-HygB | This study |
| pLC1697 | pFA-mScarlet-HygB | This study |
| pLC1699 | pFA-mNeonGreen-TPK1-HIS1 | This study |
| pLC1700 | pFA-mNeonGreen-TPK2-HIS1 | This study |
| pLC1708 | pFA-mNeonGreen-TPK1-SAT1 | This study |
| pLC1709 | pFA-mNeonGreen-TPK2-SAT1 | This study |
| pLC1717 | pFA-p2-GFP-stop2FL-HIS1 | This study |
| pLC1718 | pFA-p2-GFP-stop2N-HIS1 | This study |
| pLC1719 | pFA-p2-GFP-Qless2N-HIS1 | This study |
| pLC2041 | pFA- NLS-TPK1-HIS1 | This study |
| pLC2042 | pFA- NLS-TPK2-HIS1 | This study |

**Table S3: Oligonucleotides used in this study**

| Oligo ID | Name | Sequence (5'>3') |
|---|---|---|
| oLC747 | CaTPK2+1132_AB-F | GGACCAGCAGATATCAGAAATC |
| oLC814 | TPK1-984-F | CCTATATTTGTAGTTCTTATCC |
| oLC1077 | CaTPK2-586+Apa1-F | TTGCGGGCCCGCTGAATTTCAAGTTCCATTC |
| oLC1824 | CaTpk1-R | TGCACATCTCAGTCAAATTT |
| oLC4434 | CaPab1+1577-F | AGGTGGATACTATCCAAACC |
| oLC4435 | CaPab1+2688-R | CTCAGATATTATCCTTACTC |
| oLC5367 | CaTPK1AB+102-F | AGGATCACCTACACTAGAAG |
| oLC5368 | CaTPK1AB+309-R | GAACCTGTTCCTAATGTTCG |
| oLC5370 | CaTPK1AB+789-R | TTTGTTGCCACCACTTCAGG |
| oLC5371 | CaTPK2AB+148-F | GTGTCGAGCTCCAACATTAC |
| oLC5372 | CaTPK2AB+278-R | CAGTAGAACGTTCAGGCAAC |
| oLC5374 | CaTPK2AB+784-R | GGTACCACATAACGTCCATG |
| oLC5377 | CaHygrB+864-F | GCTCAAGGTAGATGTGATGC |
| oLC5698 | CaTPK2-278-AB | CAATACTCGGATTGCAGGTC |
| oLC5951 | HygB+341-R | TTATTGCGTACACGACCTGG |
| oLC6526 | CaDHH1+1224-F | GCTGTTCCAAGACCATTTAG |
| oLC6527 | CaDHH1+2291-R | ACTTTGAGAACACACGTTTC |
| oLC6916 | pLC1869 F (Kpp050) | TTTAAAGTCAATAGGCATTCTCG |
| oLC6924 | CaCas9/for | ATCTCATTAGATTTGGAACTTGTGGGTT |
| oLC6925 | CaCas9/rev | TTCGAGCGTCCCAAAACCTTCT |
| oLC6926 | SNR52/F | AAGAAAGAAAGAAAACCAGGAGTGAA |
| oLC6927 | sgRNA/R | ACAAATATTTAAACTCGGGACCTGG |
| oLC6928 | SNR52/N | GCGGCCGCAAGTGATTAGACT |
| oLC6929 | sgRNA/N | GCAGCTCAGTGATTAAGAGTAAAGATGG |
| oLC6942 | LM21 | CTAATTAACGTGTGTGTATGGATC |
| oLC6943 | Kpp063 | CACAGGATGACGCCTAAC |
| oLC6970 | Kpp065 | TGCTCTTGGTGGTACTGC |
| oLC6971 | Kpp068 | CATTCTGTTTATTTGAGACTACTCTTG |
| oLC7318 | CaTPK2+209-R | CGAAAAACAAAGCAGACAAGG |
| oLC7958 | HygR check R | CAACAGAAGTAGCAGTCAATTCTGG |
| oLC7959 | HygR check F | GAAGACCATCTACTAGACCAAGAGC |
| oLC8032 | NAT1-3118 | CCCAGATGCGAAGTTAAGTGCGCAG |
| oLC8033 | pDUP3-4969 | GGATTTAGTTCCATTATGG |
| oLC8428 | SNR52/R_bcy1del | CCAACCAAGGAAGTAGACCCCAAATTAAAAATAGTTTACGC AAGTC |
| oLC8429 | sgRNA/F_bcy1del | GGGTCTACTTCCTTGGTTGGGTTTTAGAGCTAGAAATAGCA AGTTAAA |

| | | |
|---|---|---|
| oLC8430 | del bcy1 F | TCACTATGTATCTATGTGATTACCTTATCAAATTGGACAATACGGATTTTTAATTGACCACGATAATTACAAACTTCCCCGTAACGGTCGACGGATCCCC |
| oLC8431 | del bcy1 R | GGGAAGCTCGTTCATGCTGCCATCTCGTATATACCTGACAACAAGAAATGTAGTTGAACTTTTGCACCAACCATCCATTATCTCGATGAATTCGAGCTCG |
| oLC8562 | SNR52/R_tpk1Tag | CCCAAACATTGTTGCTAGACCAAATTAAAAATAGTTTACGCAAGTC |
| oLC8563 | sgRNA/F_tpk1Tag | GTCTAGCAACAATGTTTGGGGTTTTAGAGCTAGAAATAGCAAGTTAAA |
| oLC8564 | tpk1 c tag sense | ATTTGATCGATACCCAGAAGATAAAGATTTGGATTATGGTATAAGTGGAGTTGAAGACCCATATCGTGATCAATTCCAGGACTTTGGTCGACGGATCCCC |
| oLC8565 | tpk1 c tag anti | ATTCTCAATGTGGTACTCAGATAAAAACCAATTCTCGCTATTATGTTTGATTCCAATATTGAACTATTGTGTGTTACACTCCTCGATGAATTCGAGCTCG |
| oLC8566 | oLC8566_tpk1 tag checkF | GTGATATAGAAACTCCATATGAACCACC |
| oLC8567 | tpk1 tag checkR | ATGGCAACTTAATAACATCCAATACC |
| oLC8568 | SNR52/R_tpk2Tag | ATACGAGTTTCATCTTTGACCAAATTAAAAATAGTTTACGCAAGTC |
| oLC8569 | sgRNA/F_tpk2Tag | GTCAAAGATGAAACTCGTATGTTTTAGAGCTAGAAATAGCAAGTTAAA |
| oLC8570 | tpk2 c tag sense | GTCATTGTTTGACCATTATCCAGAAGAACAATTAGACTACGGAAGCCAAGGAGAAGATCCTTATGCTCTGTATTTCCTTGACTTTGGTCGACGGATCCCC |
| oLC8571 | tpk2 c tag anti | TACCGAAAAACAAAGCAGACAAGGAAGAGTCAACATGTTAAGTCATTCGTATAATAACAAAACGAAAATATTAATGTGCATTTCGATGAATTCGAGCTCG |
| oLC8572 | tpk2 tag checkR | CCAGGCGTAAGATATTCTGGGTG |
| oLC8573 | SNR52/R_bcy1Tag | TCTCGTATATACCTGACAACCAAATTAAAAATAGTTTACGCAAGTC |
| oLC8574 | sgRNA/F_bcy1Tag | GTTGTCAGGTATATACGAGAGTTTTAGAGCTAGAAATAGCAAGTTAAA |
| oLC8575 | bcy1 c tag sense | CGGGTTCCAAAGATTATTGGGTCCTGTTGTGGAGGTATTGAAAGAACAAGACCCTACAAAGAGTCAAGACCCAACTGCTGGTCATGGTCGACGGATCCCC |
| oLC8576 | bcy1 c tag anti | TAGAAGCCAAGTTTGCAATATATTAGTTACCATGTTTCGCAAAAGGCACGGCCGGATAACTTCAATAATGTTCAACCCATGGTCGATGAATTCGAGCTCG |
| oLC8577 | bcy1 tag check F | GACAAGCTACTGTGGAAGCATTGG |
| oLC8578 | bcy1 tag check R | CTGTGAGAGGGATTCTCGGGAATTCTC |
| oLC8596 | tpk1 del sense | CAACCAAAACCAGGATGACATCCATGGAACCAGCAGACACAAGCATCAGGTCATTAAACGACATCAACTTACAAGAACTTGCCAAGGTCGACGGATCCCC |
| oLC8597 | tpk2 del sense | CTTCACATCACCAAGCTGCATCAACTGAATCAATCCAATTCGGACAGTAATTCCTTAAACTCAAACACATCAATGGACAATCATCGGTCGACGGATCCCC |
| oLC8604 | tpk1 com check anti | ACTCAACTATTGTCTGAATGTGTG |
| oLC8823 | SNR52/R_CaNATMarker | TCTTCACCAGCATCGGATTCCAAATTAAAAATAGTTTACGCAAGTC |
| oLC8824 | sgRNA/F_CaNATMarker | GAATCCGATGCTGGTGAAGAGTTTTAGAGCTAGAAATAGCAAGTTAAA |

| | | |
|---|---|---|
| oLC9038 | Vph1 C tag F | GGTTGAAAGTATGTCGAAATATTTTGAAGGTGGTGGTTCTG CTTTTGAACCATTTACTTTTAAAGGTTTATTAGACAGTGTTTT AGGTCGACGGATCCCC |
| oLC9039 | Vph1 C tag R | CTTAATTCTTTTTTATAAATTAATTGATGCTTTAATGAATGAA TCTAAACAAGTTAAAACCCCCAACCCTATGGTTCTAGGCTC GATGAATTCGAGCTCG |
| oLC9040 | Vph1 C tag check F | CTATGACTATTGGTAATGCATTTGGTCC |
| oLC9041 | Vph1 C tag check R | GAAGAAGGAGTACCTTTAATCTCTTCC |
| oLC9085 | nab2 c tag F | CAGGGATGGTAGTGGCAATAATAACAGTACCTCAAATCGAT CATTTGCTGTTTCTGAAGATCAGATTATGGAACAAGTTGCT CAAGGTCGACGGATCCCC |
| oLC9086 | nab2 c tag R | GGATATACACCTCTTCTACTACTATAACCTCTTCATTCCAGT TATTCAGATTATTTCAATATTGTTCTCTTGTGTTATTTTATCG ATGAATTCGAGCTCG |
| oLC9087 | nab2 check F | GTGTATGTACCAACATCCTGAAGGAAG |
| oLC10760 | GFP Q F | TGAAGGTGAAGGTGATGCTAC |
| oLC10761 | GFP Q R | CTAAGGTTGGCCATGGAACT |
| oLC11300 | mSc R Asc1 | GACTGGCGCGCCCTAGAGCTAATATAATTCATCCATACCAC C |
| oLC11381 | deltpk1 pNAT_F | AGGATGACATCCATGGAACCAGCAGACACAAGCATCAGGT CATTAAACGACATCAACTTACAAGAACTTGCCAATAATGATT TTTCCCAGTCACGACGTT |
| oLC11382 | deltpk1 pNAT_R | TTCTCAATGTGGTACTCAGATAAAAACCAATTCTCGCTATTA TGTTTGATTCCAATATTGAACTATTGTGTGTTACACTCCGTG GAATTGTGAGCGGATA |
| oLC11383 | deltpk2 pNAT_F | CAAGCTGCATCAACTGAATCAATCCAATTCGGACAGTAATT CCTTAAACTCAAACACATCAATGGACAATCATCTAATGATTT TTCCCAGTCACGACGTT |
| oLC11384 | deltpk2 pNAT_R | ACCGAAAAACAAAGCAGACAAGGAAGAGTCAACATGTTAA GTCATTCGTATAATAACAAAACGAAAATATTAATGTGCATTG TGGAATTGTGAGCGGATA |
| oLC11403 | del_vph1_F | CTCTCTCTTATCCCTTATCAATACATTTCAAGAAAAAAAGGT ATGCCGGAGAAGGAAGAAGCAGTATTCCGTTCAGCGGATA TGTGGTCGACGGATCCCC |
| oLC11404 | del_vph1_R | CAACCCTATGGTTCTAGGCTTATAAAACACTGTCTAATAAA CCTTTAAAAGTAAATGGTTCAAAAGCAGAACCACCACCTTC TCGATGAATTCGAGCTCG |
| oLC11405 | SNR52R_delvph1 | CCAACTTATACTCGTACCAACAAATTAAAAATAGTTTACGCA AGTC |
| oLC11406 | sgRNAF_delvph1 | TTGGTACGAGTATAAGTTGGGTTTTAGAGCTAGAAATAGCA AGTTAAA |
| oLC11407 | del vph1 check F | GGTCAGTCGATTGGTTTGCCACAGGGAG |
| oLC11408 | del vph1 check R | CCAAATGCATTACCAATAGTCATAGACC |
| oLC12593 | CaDHH1_NEON F | ATTTGCTGGCTACCCAGGTCAACCTCCACAACTCCCACAA GGTCAACAACAACATGCTCAAGCACAAAATCCTGCTCAACA ATATGGTCGACGGATCCCC |
| oLC12941 | Pab1-mSC F | GTTCAATAACCATTTCGAAGATGCTTTGACTGCTTTTGAAGA GTACAAGAAGTCTGAAGCTGCCGGTAATGCTGAAGAGCAA GCTGGTCGACGGATCCCC |
| oLC12942 | Pab1-mSC R | TAATCTAGATTAATTCTTGGACATCTCCCACTTTCATAAATT CCGTTACAACATCTCCTCTCTGCAATAACAATCAAGTCTTTC GATGAATTCGAGCTCG |

| | | |
|---|---|---|
| oLC12943 | Dhh1-mSC R | GCTTTAAACTATGGTACAATAAAATACAAGAACAAATGAGGAATTGGCTTTTCAACAACAGCAAAATGGAGGGTGCATAATCTCGATGAATTCGAGCTCG |

**Text S1:** Supplementary methods including strain construction details and plasmid sequences.

**Strain construction:**

**CaLC6118:** To construct the CPP-PHluorin2 strain, the repair template was digested from pLC1181 (pDUP3-CPP-PHL2) by NgoMIV (NEB) and transformed into CaLC239 (7, 8). Transformants were selected by plating onto YPD plates supplemented with 150 µg/mL NAT. Correct integration of the construct at one NEUT5L region by confirmed by PCR using oLC8032 and oLC8033.

**CaLC6489:** Both alleles of *BCY1* were deleted using a transient CRISPR approach adapted from Min and colleagues (4). The *CaCAS9* cassette was amplified from pLC963 using oLC6924 and oLC6925.The repair cassette was PCR amplified from pLC1211 using oLC8430 and oLC8431. The sgRNA fusion cassette was made by PCR amplifying from pLC1081 with oLC6926 and oLC8428 (fragment A), as well as oLC8429 and oLC6927 (fragment B), at which point fusion PCR was performed on the fragments using the nested primers oLC6928 and oLC6929 (5). The repair cassette, sgRNA, and Cas9 DNA were transformed into CaLC239. Transformants were selected by plating onto YPD plates supplemented with 600 µg/mL hygromycin B. Transformants with *BCY1* deletion were distinguishable by their yellow colony color.

**CaLC6699**: Both alleles of *TPK1* were deleted using a transient CRISPR approach adapted from Min and colleagues (4). The *CaCAS9* cassette was amplified from pLC963 using oLC6924 and oLC6925.The repair cassette was PCR amplified from pLC1100 using oLC8596 and oLC8565. The sgRNA fusion cassette was made by PCR amplifying from pLC1081 with oLC6926 and oLC8562 (fragment A), as well as oLC8563 and oLC6927 (fragment B), at which point fusion PCR was performed on the fragments using the nested primers oLC6928 and oLC6929 (5). The repair cassette, sgRNA, and Cas9 DNA were transformed into CaLC239. Transformants were selected by plating onto YNB SC plates lacking arginine. Lack of a wild-type allele was verified by PCR using oLC5367 and oLC5370.

**CaLC6702**: Both alleles of *TPK2* were deleted using a transient CRISPR approach adapted from Min and colleagues (4). The *CaCAS9* cassette was amplified from pLC963 using oLC6924 and oLC6925.The repair cassette was PCR amplified from pLC1100 using oLC8597 and oLC8571. The sgRNA fusion cassette was made by PCR amplifying from pLC1081 with oLC6926 and oLC8568 (fragment A), as well as oLC8569 and oLC6927 (fragment B), at which point fusion PCR was performed on the fragments using the nested primers oLC6928 and oLC6929 (5). The repair cassette, sgRNA, and Cas9 DNA were transformed into CaLC239. Transformants were selected by plating onto YNB SC plates lacking arginine. Lack of a wild-type allele was verified by PCR using oLC5371 and oLC5374.

**CaLC6818:** The *tpk1::pTPK1-GFP-TPK1-HIS1/tpk1::pTPK1-GFP-TPK1-SAT1* strain was constructed in two steps. First, the repair template was digested from pLC1372 using BamHI (NEB) and SacII (NEB) and transformed into CaLC6699 (*tpk1::ARG4/tpk1::ARG4*). Transformants were selected by plating on YNB SC plates lacking histidine. Reintroduction of a *GFP-TPK1* allele downstream of the native *TPK1* promoter was PCR tested using oLC814 and oLC5368. GFP-Tpk1 expression was confirmed by immunoblotting. This strain was then transformed using the repair template digested from pLC1402 using BamHI (NEB) and SacII (NEB). Transformants were selected by plating onto YNB SC plates lacking histidine and supplemented with 150 µg/mL NAT. Presence of two alleles of *GFP-TPK1* was confirmed by arginine auxotrophy and PCR confirmed using oLC814 and oLC6942. Anti-GFP immunoblotting confirmed these strains express more GFP-Tpk1 than the parental strain with one copy of *GFP-TPK1*.

**CaLC6826**: The *tpk2::pTPK2-GFP-TPK2-HIS1/tpk2::pTPK2-GFP-TPK2-SAT1* strain was constructed in two steps. First, the repair template was digested from pLC1375 using BamHI (NEB) and SacII (NEB) and transformed into CaLC6702 (*tpk2::ARG4/tpk2::ARG4*). Transformants were selected by plating on YNB SC plates lacking histidine. Reintroduction of a *GFP-TPK2* allele downstream of the native *TPK2* promoter was PCR tested using oLC1077 and oLC5372. GFP-Tpk2 expression was confirmed by immunoblotting. This strain was then transformed using the repair template digested from pLC1406 using BamHI (NEB) and SacII (NEB). Transformants were selected by plating onto YNB SC plates lacking histidine and supplemented with 150 µg/mL NAT. Presence of two alleles of *GFP-TPK2* was confirmed by arginine auxotrophy and PCR confirmed using oLC1077 and oLC6942. Anti-GFP immunoblotting confirmed these strains express more GFP-Tpk2 than the parental strain with one copy of *GFP-TPK2*.

**CaLC6836**: Both alleles of *TPK1* and *TPK2* were deleted using a transient CRISPR approach adapted from Min and colleagues (Min et al. 2016). The Ca*CAS9* cassette was amplified from pLC963 using oLC6924 and oLC6925. The *tpk1Δ::HYGB* cassette with homology to the *TPK1* locus was PCR amplified from pLC1211 using oLC8596 and oLC8565. The *tpk2Δ::HYGB* cassette with homology to the *TPK2* locus was PCR amplified from pLC1211 using oLC8597 and oLC8571. For *TPK1* the sgRNA fusion cassette was made by PCR amplifying from pLC1081 with oLC6926 and oLC8562 (fragment A), as well as oLC6927 and oLC8563 (fragment B), at which point fusion PCR was performed on the fragments using the nested primers oLC6928 and oLC6929. For *TPK2*, the sgRNA fusion cassette was made by PCR amplifying from pLC1081 with oLC6926 and oLC8568 (fragment A), as well as oLC6927 and oLC8569 (fragment B), at which point fusion PCR was performed on the fragments using the nested primers oLC6928 and oLC6929. The *tpk1Δ::HYGB* cassette, *tpk2Δ::HYGB* cassette, *TPK1* sgRNA, *TPK2* sgRNA and Cas9 DNA were transformed into CaLC239. Transformants were selected by plating onto YPD plates supplemented with 600 µg/mL hygromycin B. Colonies with homozygous deletion of both genes were identified by slow growth and white color. Lack of a wild-type *TPK1* allele was verified by PCR using oLC5367 and oLC5370. Downstream integration of the cassette was confirmed by PCR using oLC5377 and oLC1824. Lack of a wild-type *TPK2* allele was verified by PCR using oLC5371 and oLC5374. To check for correct integration, the entire *TPK2* locus was amplified using oLC5698 and oLC7318. The wild-type amplicon was 1729bp, whereas the *tpk2Δ::HYGB* amplicon was 2181bp.

**CaLC6852**: The *tpk1::pTPK1-GFP-TPK1-HIS1/tpk1::pTPK1-GFP-TPK1-SAT1 tpk2::ARG4/tpk2::ARG4* strain was constructed same as CaLC6702, except the *tpk2::ARG4* cassette was transformed into CaLC6818.

**CaLC6860:** The *tpk2::pTPK2-GFP-TPK2-HIS1/tpk2::pTPK2-GFP-TPK2-SAT1 tpk1::ARG4/tpk1::ARG4* strain was constructed same as CaLC6699 except the *tpk1::ARG4* cassette was transformed into CaLC6826.

**CaLC6978**: Both alleles of *BCY1* were C-terminally tagged using a transient CRISPR approach adapted from Min and colleagues (4). The *CaCAS9* cassette was amplified from pLC963 using oLC6924 and oLC6925.The repair cassette was PCR amplified from pLC1206 using oLC8575 and oLC8576. The sgRNA fusion cassette was made by PCR amplifying from pLC1081 with oLC6926 and oLC8573 (fragment A), as well as oLC8574 and oLC6927 (fragment B), at which point fusion PCR was performed on the fragments using the nested primers oLC6928 and oLC6929 (5). The repair cassette, sgRNA, and Cas9 DNA were transformed into CaLC239. Transformants were selected by plating onto YNB SC plates lacking histidine. Lack of the native 3' region of *BCY1* was confirmed by PCR using oLC8577 and oLC8578. Expression of a GFP protein was confirmed by both immunoblot and microscopic analysis.

**CaLC6985:** To tag *VPH1* C-terminally with RFP, the repair cassette was amplified from pLC1215 using oLC9038 and oLC9039 and transformed into CaLC6818. Transformants were selected by plating on YPD plates supplemented with 600 µg/mL hygromycin B. Correct integration was tested by PCR using oLC9040 and oLC5951.

**CaLC6987:** This strain was constructed same as CaLC6985 except the repair cassette was transformed into CaLC6826.

**CaLC6993:** This strain was constructed same as CaLC6985 except the repair cassette was transformed into CaLC6978.

**CaLC7700:** Both alleles of *TPK1* were deleted using a transient CRISPR approach adapted from Min and colleagues (4). The *CaCAS9* cassette was amplified from pLC963 using oLC6924 and oLC6925.The repair cassette was PCR amplified from pLC1087 using oLC8596 and oLC8565. The sgRNA fusion cassette was made by PCR amplifying from pLC1081 with oLC6926 and oLC8562 (fragment A), as well as oLC8563 and oLC6927 (fragment B), at which point fusion PCR was performed on the fragments using the nested primers oLC6928 and oLC6929 (5). The repair cassette, sgRNA, and Cas9 DNA were transformed into CaLC6993. Transformants were selected by plating onto YPD plates supplemented with 150 µg/mL NAT. Lack of a wild-type allele was verified by PCR using oLC5367 and oLC5370.

**CaLC7702:** Both alleles of *TPK2* were deleted using a transient CRISPR approach adapted from Min and colleagues (4). The *CaCAS9* cassette was amplified from pLC963 using oLC6924 and oLC6925.The repair cassette was PCR amplified from pLC1087 using oLC8597 and oLC8571. The sgRNA fusion cassette was made by PCR amplifying from pLC1081 with oLC6926 and oLC8568 (fragment A), as well as oLC8569 and oLC6927 (fragment B), at which point fusion PCR was performed on the fragments using the nested primers oLC6928 and oLC6929 (5). The repair cassette, sgRNA, and Cas9 DNA were transformed into CaLC6993. Transformants were selected by plating onto YPD plates supplemented with 150 µg/mL NAT. Lack of a wild-type allele was verified by PCR using oLC5371 and oLC5374.

**CaLC7878:** The *tpk1::pTPK1-NLS-GFP-TPK1-HIS1/tpk1::pTPK1-NLS-GFP-TPK1-SAT1* strain was made in two steps. First, the repair template from pLC1527 was digested using BamHI (NEB) and SacII (NEB) and transformed into CaLC6699. Transformants were selected by plating on YNB SC plates lacking histidine. Reintroduction of one *NLS-GFP-TPK1* allele downstream of the native *TPK1* promoter was PCR tested using oLC814 and oLC5368. This strain was then transformed using the repair template digested from pLC1529 using BamHI (NEB) and SacII (NEB). Transformants were selected by plating onto YPD plates supplemented with 150 µg/mL NAT. Presence of two alleles of *NLS-GFP-TPK1* was confirmed by histidine prototrophy and arginine auxotrophy. This strain showed stronger GFP signal compared to the parental strain with only one *NLS-GFP-TPK1* allele as determined by flow cytometry.

**CaLC7884:** The *tpk2::pTPK1-NLS-GFP-TPK1-HIS1/tpk2::pTPK1-NLS-GFP-TPK1-SAT1* strain was made in two steps. First, the repair template from pLC1528 was digested using BamHI (NEB) and SacII (NEB) and transformed into CaLC6702. Transformants were selected by plating on YNB SC plates lacking histidine. Reintroduction of one *NLS-GFP-TPK2* allele downstream of the native *TPK2* promoter was PCR tested using oLC1077 and oLC5372. This strain was then transformed using the repair template digested from pLC1530 using BamHI (NEB) and SacII (NEB). Transformants were selected by plating onto YPD plates supplemented with 150 µg/mL NAT. Presence of two alleles of *NLS-GFP-TPK1* was confirmed by histidine prototrophy and arginine auxotrophy. This strain showed stronger GFP signal compared to the parental strain with only one *NLS-GFP-TPK1* allele as determined by flow cytometry.

**CaLC8597:** This strain was constructed same as CaLC7700 except the repair cassette was transformed into CaLC155.

**CaLC8599:** Both alleles *TPK2* were deleted using a transient CRISPR approach adapted from Min and colleagues (Min et al. 2016). The Ca*CAS9* cassette was amplified from pLC963 using oLC6924 and oLC6925. The *tpk2Δ::HYGB* cassette with homology to the *TPK2* locus was PCR amplified from pLC1211 using oLC8597 and oLC8571. For *TPK2*, the sgRNA fusion cassette was made by PCR amplifying from pLC1081 with oLC6926 and oLC8568 (fragment A), as well as oLC6927 and oLC8569 (fragment B), at which point fusion PCR was performed on the fragments using the nested primers oLC6928 and oLC6929. The repair cassette, sgRNA and Cas9 DNA were transformed into CaLC155. Transformants were selected by plating onto YPD plates supplemented with 600 µg/mL hygromycin B. Lack of a wild-type *TPK2* allele was verified by PCR using oLC5371 and oLC5374.

**CaLC8601:** Both alleles of *TPK1* and *TPK2* were deleted using a transient CRISPR approach adapted from Min and colleagues (Min et al. 2016). The Ca*CAS9* cassette was amplified from pLC963 using oLC6924 and oLC6925. The *tpk1Δ::SAT1* cassette with homology to the *TPK1* locus was PCR amplified from pLC1087 using oLC8596 and oLC8565. The *tpk2Δ::HYGB* cassette with homology to the *TPK2* locus was PCR amplified from pLC1211 using oLC8597 and oLC8571. For *TPK1* the sgRNA fusion cassette was made by PCR amplifying from pLC1081 with oLC6926 and oLC8562 (fragment A), as well as oLC6927 and oLC8563 (fragment B), at which point fusion PCR was performed on the fragments using the nested primers oLC6928 and oLC6929. For *TPK2*, the sgRNA fusion cassette was made by PCR amplifying from pLC1081 with oLC6926 and oLC8568 (fragment A), as well as oLC6927 and oLC8569 (fragment B), at which point fusion PCR was performed on the fragments using the nested primers oLC6928 and oLC6929. The *tpk1Δ::SAT1* cassette, *tpk2Δ::HYGB* cassette, *TPK1* sgRNA, *TPK2* sgRNA and Cas9 DNA were transformed into CaLC239. Transformants were selected by plating onto YPD plates supplemented with 150 µg/mL NAT and 600 µg/mL hygromycin B. Colonies with homozygous deletion of both genes were identified by slow growth and white color. Lack of a wild-type *TPK1* allele was verified by PCR using oLC5367 and oLC5370. Lack of a wild-type *TPK2* allele was verified by PCR using oLC5371 and oLC5374.

**CaLC8867**: The *tpk1::pTPK1-GFP-(TPK2N+TPK1C)-HIS1/tpk1::pTPK1-GFP-(TPK2N+TPK1C)-SAT1* strain was constructed in two steps. First, the repair template from pLC1668 was digested using BamHI (NEB) and SacII (NEB) and transformed into CaLC6699. Transformants were selected by plating on YNB SC plates lacking histidine. Reintroduction of one *GFP-(TPK2N+TPK1C)* hybrid allele downstream of the native *TPK1* promoter was PCR tested using oLC814 and oLC10761. This strain was then transformed using the repair template digested from pLC1682 using BamHI (NEB) and SacII (NEB). Transformants were selected by plating onto YNB SC plates lacking histidine and supplemented with 150 µg/mL NAT. Presence of two alleles of *GFP-(TPK2N+TPK1C)* was confirmed by arginine auxotrophy and PCR confirmed using oLC6943 and oLC8604. Anti-GFP immunoblotting and fluorescence microscopy confirmed that this strain expresses more GFP compared to the parental strain with only one *GFP-(TPK2N+TPK1C)* allele.

**CaLC8869:** The *tpk1::pTPK1-GFP-TPK2-HIS1/tpk1::pTPK1-GFP-TPK2-SAT1* strain was constructed in two steps. First, the repair template from pLC1669 was digested using BamHI (NEB) and SacII (NEB) and transformed into CaLC6699. Transformants were selected by plating on YNB SC plates lacking histidine. Reintroduction of one *GFP-TPK2* allele downstream of the native *TPK1* promoter was PCR tested using oLC814 and oLC10761. This strain was then transformed using the repair template digested from pLC1683 using BamHI (NEB) and SacII (NEB). Transformants were selected by plating onto YNB SC plates lacking histidine and supplemented with 150 µg/mL NAT. Presence of two alleles of *GFP-TPK2* was confirmed by arginine auxotrophy and PCR confirmed using oLC6943 and oLC8604. Anti-GFP immunoblotting and fluorescence microscopy confirmed that this strain expresses more GFP compared to the parental strain with only one *GFP-TPK2* allele.

**CaLC8873:** The *tpk1::pTPK1-GFP-(TPK1N+TPK2C)-HIS1/tpk1::pTPK1-GFP-(TPK1N+TPK2C)-SAT1* strain was constructed in two steps. First, the repair template from pLC1676 was digested using BamHI (NEB) and SacII (NEB) and transformed into CaLC6699. Transformants were selected by plating on YNB SC plates lacking histidine. Reintroduction of one *GFP-(TPK1N+TPK2C)* hybrid allele downstream of the native *TPK1* promoter was PCR tested using oLC814 and oLC10761. This strain was then transformed using the repair template digested from pLC1685 using BamHI (NEB) and SacII (NEB). Transformants were selected by plating onto YNB SC plates lacking histidine and supplemented with 150 µg/mL NAT. Presence of two alleles of *GFP-(TPK1N+TPK2C)* was confirmed by arginine auxotrophy and PCR confirmed using oLC6943 and oLC8604. Anti-GFP immunoblotting and fluorescence microscopy confirmed that this strain expresses more GFP compared to the parental strain with only one *GFP-(TPK1N+TPK2C)* allele.

**CaLC8946**: To tag *VPH1* C-terminally with mNeonGreen, the repair cassette was amplified from pLC1696 using oLC9038 and oLC9039 and transformed into CaLC239. Transformants were selected by plating on YPD plates supplemented with 600 µg/mL hygromycin B. Upstream integration of the cassette was confirmed by PCR using oLC9040 and oLC7958 and downstream integration was confirmed using oLC7959 and oLC9041.

**CaLC8948**: This strain was constructed same as CaLC8946 except the repair cassette was transformed into CaLC6699.

**CaLC8950**: This strain was constructed same as CaLC8946 except the repair cassette was transformed into CaLC6702.

**CaLC8966:** The *tpk2::pTPK2-GFP-(TPK1N+TPK2C)-HIS1/tpk2::pTPK2-GFP-(TPK1N+TPK2C)-SAT1 tpk1::HygB/tpk1::HygB* strain was constructed in three steps. First, the repair template from pLC1671 was digested using BamHI (NEB) and SacII (NEB) and transformed into CaLC6702. Transformants were selected by plating on YNB SC plates lacking histidine. Reintroduction of one *GFP-(TPK1N+TPK2C)* hybrid allele downstream of the native *TPK2* promoter was PCR tested using oLC10760 and oLC10761 for presence of GFP coding sequence and 3' integration was PCR confirmed using oLC6916 and oLC8572. This strain was then transformed using the repair template digested from pLC1686 using BamHI (NEB) and SacII (NEB). Transformants were selected by plating onto YNB SC plates lacking histidine and supplemented with 150 µg/mL NAT. Presence of two alleles of *GFP-(TPK1N+TPK2C)* was confirmed by arginine auxotrophy and PCR confirmed using oLC6943 and oLC8572 for 3' integration. Anti-GFP immunoblotting and fluorescence microscopy confirmed that this strain expresses more GFP compared to the parental strain with only one *GFP-(TPK1N+TPK2C)* allele.

Next, both alleles of *TPK1* were deleted using a transient CRISPR approach adapted from Min and colleagues (Min et al. 2016). The Ca*CAS9* cassette was amplified from pLC963 using oLC6924 and oLC6925. The repair cassette was PCR amplified from pLC1211 using oLC8596 and oLC8565. The sgRNA fusion cassette was made by PCR amplifying from pLC1081 with oLC6926 and oLC8562 (fragment A), as well as oLC6927 and oLC8563 (fragment B), at which point fusion PCR was performed on the fragments using the nested primers oLC6928 and oLC6929. The repair cassette, sgRNA, and Cas9 DNA were transformed into CaLC239. Transformants were selected by plating onto YPD plates supplemented with 600 µg/mL hygromycin B. Lack of a wild-type *TPK1* allele was verified by PCR using oLC5367 and oLC5370.

**CaLC8968:** The *tpk2::pTPK2-GFP-TPK1-HIS1/tpk2::pTPK2-GFP-TPK1-SAT1 tpk1::HygB/tpk1::HygB* strain was constructed in three steps. First, the repair template from pLC1672 was digested using BamHI (NEB) and SacII (NEB) and transformed into CaLC6702. Transformants were selected by plating on YNB SC plates lacking histidine. Reintroduction of one *GFP-TPK1* allele downstream of the native *TPK2* promoter was PCR tested using oLC10760 and oLC10761 for presence of GFP coding sequence and 3' integration was PCR confirmed using oLC6916 and oLC8572. This strain was then transformed using the repair template digested from pLC1687 using BamHI (NEB) and SacII (NEB). Transformants were selected by plating onto YNB SC plates lacking histidine and supplemented with 150 µg/mL NAT. Presence of two alleles of *GFP-TPK1* was confirmed by arginine auxotrophy and PCR confirmed using oLC6943 and oLC8572 for 3' integration. Anti-GFP immunoblotting and fluorescence microscopy confirmed that this strain expresses more GFP compared to the parental strain with only one *GFP-TPK1* allele. Both alleles of *TPK1* were subsequently deleted as described for CaLC8966.

**CaLC8972:** The *tpk2::pTPK2-GFP-(TPK2N+TPK1C)-HIS1/tpk2::pTPK2-GFP-(TPK2N+TPK1C)-SAT1 tpk1::HygB/tpk1::HygB* strain was constructed in three steps. First, the repair template from pLC1677 was digested using BamHI (NEB) and SacII (NEB) and transformed into CaLC6702. Transformants were selected by plating on YNB SC plates lacking histidine. Reintroduction of one *GFP-(TPK2N+TPK1C)* hybrid allele downstream of the native *TPK2* promoter was PCR tested using oLC10760 and oLC10761 for presence of the GFP coding sequence and 3' integration was PCR confirmed using oLC6916 and oLC8572. This strain was then transformed using the repair template digested from pLC1689 using BamHI (NEB) and SacII (NEB). Transformants were selected by plating onto YNB SC plates lacking histidine and supplemented with 150 µg/mL NAT. Presence of two alleles of *GFP-(TPK2N+TPK1C)* was confirmed by arginine auxotrophy and PCR confirmed using oLC6943 and oLC8572 for 3' integration. Anti-GFP immunoblotting and fluorescence microscopy confirmed that this strain expresses more GFP compared to the parental strain with only one *GFP-(TPK2N+TPK1C)* allele. Both alleles of *TPK1* were subsequently deleted as described for CaLC8966.

**CaLC8995:** Both alleles of *TPK2* were C-terminally tagged using a transient CRISPR approach adapted from Min and colleagues (4). The *CaCAS9* cassette was amplified from pLC963 using oLC6924 and oLC6925.The repair cassette was PCR amplified from pLC1214 using oLC8570 and oLC8571. The sgRNA fusion cassette was made by PCR amplifying from pLC1081 with oLC6926 and oLC8568 (fragment A), as well as oLC8569 and oLC6927 (fragment B), at which point fusion PCR was performed on the fragments using the nested primers oLC6928 and oLC6929 (5). The repair cassette, sgRNA, and Cas9 DNA were transformed into CaLC6699. Transformants were selected by plating onto YPD plates supplemented with 600 µg/mL hygromycin B . Lack of the native 3' region of *TPK2* was confirmed by PCR using oLC747 and oLC8572.

**CaLC8997:** Both alleles of *TPK1* were C-terminally tagged using a transient CRISPR approach adapted from Min and colleagues (4). The *CaCAS9* cassette was amplified from pLC963 using oLC6924 and oLC6925.The repair cassette was PCR amplified from pLC1214 using oLC8564 and oLC8565. The sgRNA fusion cassette was made by PCR amplifying from pLC1081 with oLC6926 and oLC8562 (fragment A), as well as oLC8563 and oLC6927 (fragment B), at which point fusion PCR was performed on the fragments using the nested primers oLC6928 and oLC6929 (5). The repair cassette, sgRNA, and Cas9 DNA were transformed into CaLC6702. Transformants were selected by plating onto YPD plates supplemented with

600 μg/mL hygromycin B. Lack of the native 3' region of *TPK1* was confirmed by PCR using oLC8566 and oLC8567.

**CaLC9013:** The *tpk1::pTPK1-mNG-TPK1-HIS1/tpk1::pTPK1-mNG-TPK1-SAT1* strain was constructed in two steps. First, the repair template was digested from pLC1699 using BamHI (NEB) and SacII (NEB) and transformed into CaLC6699. Transformants were selected by plating on YNB SC plates lacking histidine. Reintroduction of a *mNG-TPK1* allele downstream of the native *TPK1* promoter was PCR tested using oLC6916 and oLC8604 for 3' integration. The presence of *TPK1* orf was confirmed by PCR using oLC5367 and oLC5370. This strain was then transformed using the repair template digested from pLC1708 using BamHI (NEB) and SacII (NEB). Transformants were selected by plating onto YNB SC plates lacking histidine and supplemented with 150 μg/mL NAT. Presence of two alleles of *mNG-TPK1* was confirmed by arginine auxotrophy and PCR confirmed using oLC6943 and oLC8604 for 3' integration. Upstream integration was confirmed using oLC8566 and oLC6942.

**CaLC9015:** The *tpk2::pTPK2-mNG-TPK2-HIS1/tpk1::pTPK2-mNG-TPK2-SAT1* strain was constructed in two steps. First, the repair template was digested from pLC1700 using BamHI (NEB) and SacII (NEB) and transformed into CaLC6702. Transformants were selected by plating on YNB SC plates lacking histidine. Reintroduction of a *mNG-TPK2* allele downstream of the native *TPK2* promoter was PCR tested using oLC6916 and oLC8572 for 3' integration. The presence of *TPK2* orf was confirmed by PCR using oLC5371 and oLC5374. This strain was then transformed using the repair template digested from pLC1709 using BamHI (NEB) and SacII (NEB). Transformants were selected by plating onto YNB SC plates lacking histidine and supplemented with 150 μg/mL NAT. Presence of two alleles of *mNG-TPK2* was confirmed by arginine auxotrophy and PCR confirmed using oLC6943 and oLC8572 for 3' integration. Upstream integration was confirmed using oLC747 and oLC6942.

**CaLC9049:** The *tpk2::pTPK2-GFP-TPK2-HIS1/tpk2::pTPK2-GFP-TPK2-HIS1 VPH1-mSC-HygB/VPH1* strain was constructed in three steps. First, to tag *VPH1* C-terminally with mScarlet, the targeting cassette was amplified from pLC1696 using oLC9038 and oLC9039 and transformed into CaLC239. Transformants were selected by plating on YPD plates supplemented with 600 μg/mL hygromycin B. Upstream integration of the cassette was confirmed by PCR using oLC9040 and oLC7958 and downstream integration was confirmed using oLC7959 and oLC9041.

Next, both alleles of *TPK2* were deleted using a transient CRISPR approach adapted from Min and colleagues (4). The *CaCAS9* cassette was amplified from pLC963 using oLC6924 and oLC6925. The repair cassette was PCR amplified from pLC1082 using oLC11383 and oLC11384. The sgRNA fusion cassette was made by PCR amplifying from pLC1081 with oLC6926 and oLC8568 (fragment A), as well as oLC8569 and oLC6927 (fragment B), at which point fusion PCR was performed on the fragments using the nested primers oLC6928 and oLC6929 (5). The repair cassette, sgRNA, and Cas9 DNA were transformed into the strain above. Transformants were selected by plating onto YPD plates supplemented with 150 μg/mL NAT. Lack of a wild-type allele was verified by PCR using oLC5371 and oLC5374.

Finally, the two copies of *GFP-TPK2* allele was introduced using a transient CRISPR approach adapted from Min and colleagues (4). The *CaCAS9* cassette was amplified from pLC963 using oLC6924 and oLC6925. The repair template was digested from pLC1375 using BamHI (NEB) and SacII (NEB). The sgRNA fusion cassette was made by PCR amplifying from pLC1081 with oLC6926 and oLC8823 (fragment A), as well as oLC8824 and oLC6927 (fragment B), at which point fusion PCR was performed on the fragments using the nested primers oLC6928 and oLC6929 (5). Transformants were selected by plating onto YNB SC plates lacking histidine. Presence of two alleles of *GFP-TPK2* was confirmed by NAT sensitivity. Expression of the GFP-TPK2 fusion protein was confirmed by anti-GFP immunoblotting.

**CaLC9051:** This strain was constructed same as CaLC9049 except in the final step the repair cassette containing the N-terminus of TPK2 (*GFP-TPK2NΔC*) was digested from pLC1673 using BamHI (NEB) and SacII (NEB) for transformation.

**CaLC9053:** This strain was constructed same as CaLC9049 except in the final step the repair cassette containing an extra translation stop codon just upstream of the start codon (*STOP-GFP-TPK2*) was digested from pLC1717 using BamHI (NEB) and SacII (NEB) for transformation.

**CaLC9055:** This strain was constructed same as CaLC9049 except in the final step the repair cassette containing the N-terminus of TPK2 with an extra translation stop codon just upstream of the start codon (*STOP-GFP-TPK2NΔC*) was digested from pLC1718 using BamHI (NEB) and SacII (NEB) for transformation.

**CaLC9057**: This strain was constructed same as CaLC9049 except in the final step the repair cassette containing the N-terminus of *TPK2* lacking its N-terminal poly glutamine PolyQ tract (*GFP-TPK2NΔC*$^{\Delta Q}$) was digested from pLC1719 using BamHI (NEB) and SacII (NEB) for transformation.

**CaLC9063**: Both alleles of *TPK1* were deleted using a transient CRISPR approach adapted from Min and colleagues (4). The *CaCAS9* cassette was amplified from pLC963 using oLC6924 and oLC6925. The repair cassette was PCR amplified from pLC1082 using oLC11381 and oLC11382. The sgRNA fusion cassette was made by PCR amplifying from pLC1081 with oLC6926 and oLC8562 (fragment A), as well as oLC8563 and oLC6927 (fragment B), at which point fusion PCR was performed on the fragments using the nested primers oLC6928 and oLC6929 (5). The repair cassette, sgRNA, and Cas9 DNA were transformed into CaLC9049. Transformants were selected by plating onto YPD plates supplemented with 150 µg/mL NAT. Lack of a wild-type allele was verified by PCR using oLC5367 and oLC5370.

**CaLC9103:** Both alleles of *VPH1* were deleted using a transient CRISPR approach adapted from Min and colleagues (4). The *CaCAS9* cassette was amplified from pLC963 using oLC6924 and oLC6925.The repair cassette was PCR amplified from pLC1211 using oLC11403 and oLC11404. The sgRNA fusion cassette was made by PCR amplifying from pLC1081 with oLC6926 and oLC11405 (fragment A), as well as oLC11406 and oLC6927 (fragment B), at which point fusion PCR was performed on the fragments using the nested primers oLC6928 and oLC6929 (5). The repair cassette, sgRNA, and Cas9 DNA were transformed into CaLC6118. Transformants were selected by plating onto YPD plates supplemented with 600 µg/mL hygromycin B. Lack of a wild-type allele was verified by PCR using oLC11407 and oLC11408.

**CaLC9154**: Both alleles of *TPK1* were deleted as CaLC8966 except the repair cassette was transformed into CaLC6118.

**CaLC9156**: This strain was constructed same as CaLC8599 except the repair cassette was transformed into CaLC6118.

**CaLC9158**: This strain was constructed same as CaLC6836 except the repair cassettes was transformed into CaLC6118.

**CaLC9458:** To tag *NAB2* C-terminally with mScarlet, the targeting cassette was amplified from pLC1697 using oLC9085 and oLC9086 and transformed into CaLC9013. Transformants were selected by plating on YPD plates supplemented with 600 µg/mL hygromycin B. Upstream integration of the cassette was confirmed by PCR using oLC9087 and oLC11300.

**CaLC9460:** This strain was constructed same as CaLC9048 except the repair cassette was transformed into CaLC9015.

**CaLC9478:** Both alleles of *TPK1* and *TPK2* were deleted using a transient CRISPR approach adapted from Min and colleagues (Min et al. 2016). The Ca*CAS9* cassette was amplified from pLC963 using oLC6924 and oLC6925. The *tpk1Δ::SAT1* cassette with homology to the *TPK1* locus was PCR amplified from

pLC1087 using oLC8596 and oLC8565. The *tpk2Δ::SAT1* cassette with homology to the *TPK2* locus was PCR amplified from pLC1087 using oLC8597 and oLC8571. For *TPK1* the sgRNA fusion cassette was made by PCR amplifying from pLC1081 with oLC6926 and oLC8562 (fragment A), as well as oLC6927 and oLC8563 (fragment B), at which point fusion PCR was performed on the fragments using the nested primers oLC6928 and oLC6929. For *TPK2*, the sgRNA fusion cassette was made by PCR amplifying from pLC1081 with oLC6926 and oLC8568 (fragment A), as well as oLC6927 and oLC8569 (fragment B), at which point fusion PCR was performed on the fragments using the nested primers oLC6928 and oLC6929. The *tpk1Δ::SAT1* cassette, *tpk2Δ::SAT1* cassette, *TPK1* sgRNA, *TPK2* sgRNA and Cas9 DNA were transformed into CaLC6118. Transformants were selected by plating onto YPD plates supplemented with 150 µg/mL NAT. Colonies with homozygous deletion of both genes were identified by slow growth and white color. Lack of a wild-type *TPK1* allele was verified by PCR using oLC5367 and oLC5370. Lack of a wild-type *TPK2* allele was verified by PCR using oLC5371 and oLC5374.

**CaLC10330**: To tag *PAB1* C-terminally with mScarlet, the targeting cassette was amplified from pLC1693 using oLC12941 and oLC12942 and transformed into CaLC9013. Transformants were selected by plating on YNB SC plates lacking arginine. Upstream integration of the cassette was confirmed by PCR using oLC4434 and oLC6971 and downstream integration was confirmed using oLC6970 and oLC4435.

**CaLC10332:** This strain was constructed same as CaLC10330 except the repair cassette was transformed into CaLC9015.

**CaLC10334:** This strain was constructed same as CaLC10330 except the repair cassette was transformed into CaLC6978.

**CaLC10371:** Both alleles of *DHH1* was C-terminally with mScarlet in two steps. First, the targeting cassette was amplified from pLC1693 using oLC12943 and oLC12593 and transformed into CaLC9013. Transformants were selected by plating on YNB SC plates lacking arginine. Upstream integration of the cassette was confirmed by PCR using oLC6526 and oLC6971 and downstream integration was confirmed using oLC6970 and oLC6527. This strain was subsequently transformed using the repair template amplified from pLC1697 using oLC12943 and oLC12593. Transformants were selected by plating on YNB SC plates lacking arginine and supplemented with 600 µg/mL hygromycin B. Upstream integration of the cassette was confirmed by PCR using oLC6526 and oLC7958 and downstream integration was confirmed using oLC7959 and oLC6527. Lack of the native 3' region of *DHH1* was confirmed by PCR using oLC6526 and oLC6527.

**CaLC10373:** This strain was constructed same as CaLC10371 except the repair cassette was transformed into CaLC9015.

**CaLC10375:** This strain was constructed same as CaLC10371 except the repair cassette was transformed into CaLC6978.

**CaLC10445:** Both alleles of *TPK2* were deleted as CaLC8599 except the repair cassette was transformed into CaLC9013.

**CaLC10447:** Both alleles of *TPK1* were deleted as CaLC8966 except the repair cassette was transformed into CaLC9015.

**CaLC10572:** The *tpk1::pTPK1-NLS-TPK1-HIS1/tpk1::pTPK1-NLS-TPK1-HIS1* strain was made using two steps. First, both alleles of *TPK1* were deleted as CaLC9063 except the repair cassette was transformed into CaLC239. Next, the two copies of *NLS-TPK1* allele was introduced using a transient a CRISPR approach adapted from Min and colleagues (4). The *CaCAS9* cassette was amplified from pLC963 using

oLC6924 and oLC6925. The repair template was digested from pLC2041 using BamHI (NEB) and SacII (NEB). The sgRNA fusion cassette was made by PCR amplifying from pLC1081 with oLC6926 and oLC8823 (fragment A), as well as oLC8824 and oLC6927 (fragment B), at which point fusion PCR was performed on the fragments using the nested primers oLC6928 and oLC6929 (5). The repair cassette, sgRNA, and Cas9 DNA were transformed into the *TPK1* deletion strain. Transformants were selected by plating onto YNB SC plates lacking histidine. Presence of two alleles of *NLS-TPK1* was confirmed by NAT sensitivity.

**CaLC10575:** The *tpk2::pTPK2-NLS-TPK2-HIS1/tpk2::pTPK2-NLS-TPK2-HIS1* strain was made using two steps. First, both alleles of *TPK2* were deleted as described for CaLC9049 except the repair cassette was transformed into CaLC239. Next, the two copies of *NLS-TPK2* allele was introduced using a transient a CRISPR approach adapted from Min and colleagues (4). The *CaCAS9* cassette was amplified from pLC963 using oLC6924 and oLC6925. The repair template was digested from pLC2042 using BamHI (NEB) and SacII (NEB). The sgRNA fusion cassette was made by PCR amplifying from pLC1081 with oLC6926 and oLC8823 (fragment A), as well as oLC8824 and oLC6927 (fragment B), at which point fusion PCR was performed on the fragments using the nested primers oLC6928 and oLC6929 (5). The repair cassette, sgRNA, and Cas9 DNA were transformed into the *TPK2* deletion strain. Transformants were selected by plating onto YNB SC plates lacking histidine. Presence of two alleles of *NLS-TPK2* was confirmed by NAT sensitivity.

## Plasmid sequences:

>pLC1372_pFA-GFP-TPK1-HIS

GAAGATCCTTTGATCTTTTCTACGGGGTCTGACGCTCAGTGGAACGAAAACTCACGTTAAGGGATTTTGGTCATGAGATTATCAAAAAGGATCTTCACCTAGATCCTTTTAAAT
TAAAAATGAAGTTTTAAATCAATCTAAAGTATATATGAGTAAACTTGGTCTGACAGTTACCAATGCTTAATCAGTGAGGCACCTATCTCAGCGATCTGTCTATTTCGTTCATCCA
TAGTTGCCTGACTCCCCGTCGTGTAGATAACTACGATACGGGAGGGCTTACCATCTGGCCCCAGTGCTGCAATGATACCGCGAGACCCACGCTCACCGGCTCCAGATTTAT
CAGCAATAAACCAGCCAGCCGGAAGGGCCGAGCGCAGAAGTGGTCCTGCAACTTTATCCGCCTCCATCCAGTCTATTAATTGTTGCCGGGAAGCTAGAGTAAGTAGTTCGC
CAGTTAATAGTTTGCGCAACGTTGTTGCCATTGCTACAGGCATCGTGGTGTCACGCTCGTCGTTTGGTATGGCTTCATTCAGCTCCGGTTCCCAACGATCAAGGCGAGTTAC
ATGATCCCCCATGTTGTGCAAAAAAGCGGTTAGCTCCTTCGGTCCTCCGATCGTTGTCAGAAGTAAGTTGGCCGCAGTGTTATCACTCATGGTTATGGCAGCACTGCATAATT
CTCTTACTGTCATGCCATCCGTAAGATGCTTTTCTGTGACTGGTGAGTACTCAACCAAGTCATTCTGAGAATAGTGTATGCGGCGACCGAGTTGCTCTTGCCCGGCGTCAATA
CGGGATAATACCGCGCCACATAGCAGAACTTTAAAAGTGCTCATCATTGGAAAACGTTCTTCGGGGCGAAAACTCTCAAGGATCTTACCGCTGTTGAGATCCAGTTCGATGT
AACCCACTCGTGCACCCAACTGATCTTCAGCATCTTTTACTTTCACCAGCGTTTCTGGGTGAGCAAAAACAGGAAGGCAAAATGCCGCAAAAAAGGGAATAAGGGCGACACG
GAAATGTTGAATACTCATACTCTTCCTTTTTCAATATTATTGAAGCATTTATCAGGGTTATTGTCTCATGAGCGGATACATATTTGAATGTATTTAGAAAAATAAACAAATAGGG
GTTCCGCGCACATTTCCCCGAAAAGTGCCACCTGACGTCTAAGAAACCATTATTATCATGACATTAACCTATAAAAATAGGCGTATCACGAGGCCCTTTCGTCTCGCGCGTTT
CGGTGATGACGGTGAAAACCTCTGACACATGCAGCTCCCGGAGACGGTCACAGCTTGTCTGTAAGCGGATGCCGGGAGCAGACAAGCCCGTCAGGGCGCGTCAGCGGGT
GTTGGCGGGTGTCGGGGCTGGCTTAACTATGCGGCATCAGAGCAGATTGTACTGAGAGTGCACCATATGGACATATTGTCGTTAGAACGCGGCTACAATTAATACATAACCT
TATGTATCATACACATACGATTTAGGTGACACTATAGAACGCGGCCGCCAGCTGAAGCTTCGTACGCTGCAGGTCGACGGATCCAGACAGAAGATAATATTTTTTCTTAACTG
TCACGCAAAAGTTTTAACTAACTAACTAACTAACTAATTAGACCAATTCCCCTAAAGGACTTAAGGACTAAAATAAGCTTATTTCAATTAATATTATCATTGAATAATAGATACTT
ATAGCAGTTATAGTAGAATTTAATTTCTTTTTTTTTTCTTTCTTTCTTTTGTCACACATTTATCCGTTTTGACAGACTAAATTAAACAAAAAATTTTTGAACATCAGGGAACGACCA
TATACAAATAAAAGCAGAAGAAAAAAACGGAAGAAGAAGAAGAAGAAAAAAAAGTCAACCAAAAAATTGAACAAGAGGTCTAAAGAACCACAACATTATACAAAAGAAAAGAA
ATTAAATTAAATTACATTAAAAGAGAATTGAAACATAACACTTGAAGAAGAAAATAAAAGTATTTTTATATTATATATTCCGACTAGTATTATCTGAGATCAATATATCCTATCGTT
ATCCTCCTCCTTCTCCTTTCAACTTTTGAAAAAGGTGATATTATTCAACACTGTTTTTGTTTTATCAACCAAAACCAGGATGTCTAAAGGTGAAGAATTATTCACTGGTGTTGTC
CCAATTTTGGTTGAATTAGATGGTGATGTTAATGGTCACAAATTTTCTGTCTCCGGTGAAGGTGAAGGTGATGCTACTTACGGTAAATTGACCTTAAAATTTATTTGTACTACTG
GTAAATTGCCAGTTCCATGGCCAACCTTAGTCACTACTTTCGGTTATGGTGTTCAATGTTTTGCGAGATACCCAGATCATATGAAACAACATGACTTTTTCAAGTCTGCCATGC
CAGAAGGTTATGTTCAAGAAAGAACTATTTTTTTCAAAGATGACGGTAACTACAAGACCAGAGCTGAAGTCAAGTTTGAAGGTGATACCTTAGTTAATAGAATCGAATTAAAAG
GTATTGATTTTAAAGAAGATGGTAACATTTTAGGTCACAAATTGGAATACAACTATAACTCTCACAATGTTTACATCATGGCTGACAAACAAAAGAATGGTATCAAAGTTAACTT
CAAAATTAGACACAACATTGAAGATGGTTCTGTTCAATTAGCTGACCATTATCAACAAAATACTCCAATTGGTGATGGTCCAGTCTTGTTACCAGACAACCATTACTTATCCAC
TCAATCTGCCTTATCCAAAGATCCAAACGAAAAGAGAGACCACATGGTCTTGTTAGAATTTGTTACTGCTGCTGGTATTACCCATGGTATGGATGAATTGTACAAAGGTGGTG
GTGGTACATCCATGGAACCAGCAGACACAAGCATCAGGTCATTAAACGACATCAACTTACAAGAACTTGCCAACAAACAACATCAACGAAATATATATGCTCAAGGATCACCT
ACACTAGAAGATTCAATAACATCAAATGGTAATGATATTAATAATCCCAACAATAATATAAATAAAACCAATAATAACAGTAATTCAAGTCTTTCCTTGACCAAGAAAACATCAAG
ACATATCAATAAAGACACTACAACTAAAGGCAAATATACATTAAATGATTTTCAAATATTACGAACATTAGGAACAGGTTCCTTTGGTAGAGTTCATTTAACTCGATCGATTCAT
AATGGTAGATTTTATGCTATGAAAGTATTAAAGAAACAACGAGTAGTACAAATGAAACAAATCGAACATACTAATGATGAAAGAAGAATGTTAAAATTAGCTCAACATCCATTTA
TAATTCGAATGTGGGGGACATTCCAAGATTGTCATAATTTATTTATGATTATGGATTATATAGAAGGAGGTGAATTATTTTCATTATTAAGAAAATCCCAACGATTCCCTACTCC
AGTAGCTAAATTTTATGCTGCCGAAGTATTTTTGGCTATTGAATATTTACACAGTTTAGACATTATTTATCGAGATTTAAAACCAGAAAATATATTATTGGATAAAAATGGTCATA
TCAAATTAACTGATTTTGGATTTGCTAAAGAAGTTCAAGATGTGACTTATACTTTATGTGGGACTCCAGATTATATTGCTCCTGAAGTGGTGGCAACAAAACCTTATAATAAATC
AGTTGATTGGTGGTCATTTGGTATATTAATATTTGAAATGTTAACTGGTTATACTCCATTCTATGATCCTACTCCAATGAAAACTTATGAAAACATTTTAAATGGATCAATTACTT
ATCCTGATTATTTACCTCCAGATATATTGGATCTTCTACAAAAATTAATTGTCAAAGATTTAACTCAAAGATTAGGTAATTTACAAGGTGGTTCTGATGATGTGAAAAATCATCC
GTGGTTTAAAGAAGTTATTTGGGAAAGATTGTTGTCACGTGATATAGAAACTCCATATGAACCACCTATAACTTCTGGTGTTGGTGATACTTCACAATTTGATCGATACCCAGA
AGATAAAGATTTGGATTATGGTATAAGTGGAGTTGAAGACCCATATCGTGATCAATTCCAGGACTTTTAAATGTAAAAATAAAATTGTATTTATTGGCACAACAATGTTAATTAT
GATAACTAGTTTTATAGATGTATAATTAATTTGCAAGAAAGAAGAAGACGAAGATTTGATTAGTGAATTTTTTAAAGCTAAAAAAACACTTCTTGGTGTATCAAAAGTAATTTTGG
CAAATCTTATAGACAGCTAAAATATAAGCAGTGTCTTATTTTTAAACCAATGATCACTATATGAATTGGCTTCCGGCGCGCCATTATAAGTAAATGCATGTATACTAAACTCACA
AATTAGAGCTTCAATTTAATTATATCAGTTATTACCCGGGAATCTCGGTCGTAATGATTTCTATAATGACGAAAAAAAAAAAATTGGAAAGAAAAAGCTTCATGGCCTTTATAAAA
AGGAACTATCCAATACCTCGCCAGAACCAAGTAACAGTATTTTACGGGGCACAAATCAAGAACAATAAGACAGGACTGTAAAGATGGACGCATAGATCCTGGAGGATGAGGA
GACAGAAGTTAGTAGTAACAATTTGGGGACAGAAGAGGATACAGTGACATCAGAGGAAGAAGACAGCGAAGGTATAGTCAAAAAGCAATAGGACAGCAAGACAATTTATTTA
TTTAATAGTTTTTTATAAATGTAAAATCTATCACAACATGTGTACTTAATTTTCTGTGGTAGGGGAGGCCATTTCGTTGAAACGTGTCGCGAGCGGTTGTGAGAATTGACTCATT
TTGATTATTACGCAACCATTGAAATTTTTTATTTTTTTTTGGTGAAGATTTTCCCACACAACTTCTTCTTTTACTTAACCACCAACTACCGATAATGGATTTAGTCAATCATTTAC
CAGACCGTTTGTTATTTGCTGTTCCTAAAAAGGGCAGATTATACGAAAAATGCTGTAACTTATTGAGTGGTGCCGATATACAGTTTAGAAGATCTAATAGATTAGATATAGCAC
TTTCTACAAACTTGCCAATTGCATTAATCTTCTTGCCTGCAGCTGATATCCCAGTTTTCGTTGGAGAAGGTAATTGTGACTTGGGTATAACTGGGTTAGACCAAATCAAAGAAG
CTGAACAATTCGACAACATCGAGGACTTGTTGGATTTGAAATTTGGTTCATGCAAATTGCAGATCCAAGTTCCAGCAGATGGCGAGTACGAAAAGCCAGAACAGCTTGTTGG
AAAGAAAATTGTGTCTTCATTTACAAAATTGAGTACCGACTATTTCAAACAATTGTCAGACAAACCTACTAATATCAGATATGTCGGTGGTTCCGTTGAGGCTTCTTGTGCCTT
GGGTGTTGCTGATGCTATTGTCGATTTGGTTGAAAGTGGTGAAACTATGAAAGCAGCTGGGTTAAAGGCCATAGAAACCATATTGGAGACTTCGGCTCATTTGATTTCGTCAA
AAAAAAGTAAATTCCCAGAAATGGTCAATATAATCGTCCAAAGACTTCAAGGTGTTTTAGCTGCCCAGGAATATGTCTTGTGTAACTACAATGCCCCAAAATCCATCCAAGCAA
AATGTTTAACCATTACTCCAGGCAGAAGGGCCGCTACTGTCTCTACTTTGGACAAACACAGCGACGACGAAGAGGACTGGGTTGCCATCTCATCCATGGTTAATAGAAAGGA
AATCGGTAATGTAATGGACGAATTGAAGAAAGCTGGTGCAACCGATATATTGGTACTCGAGATATCAAATTGTAGAGTTTAAAAGAAGTGATAGTTTCTCATAAATATTATACA
TATATACTTATAGATCTACTTCAATTTGACTCAATCTGTCTTATACCATCTTTATCTACCACCTTGATGTACACACCTTTAAAGTCAATAGGCATTCTCGTTTAAACGAGCTCCAC
AATAGTTCAATATTGGAATCAAACATAATAGCGAGAATTGGTTTTTATCTGAGTACCACATTGAGAATGGTATTGGATGTTATTAAGTTGCCATTTTTGAATGAACTTCTCACTG
ATGATTCTTTTTATTAAATCCAGGGTCCTTTTTTTAGGGTATTCTACTAAAACTTGATATTTGGGATGATTTGGTAGTTCTATTTTGATTTCCACTAATGAAAAGCATTTCTAACAA
GTTTTGGAAATTTGACTGAGATGTGCATTACTAAGAGCTTCGTTGTTACGGTGATACTAAAGTTTGTCAAGTCTCTTAAACATTACATTGAATACTATTTGATAGTTGGTGATAA
AAAAAACTCCGTAAACAATAATTTCGTGTTTAATTCACATTTTATTGGAGCATGAAAATTATTTAACCGAACGCGTTCTTGAATGTGTATAAAAATAGAAATCCCATATTATCGAA
TTAGAGGTCTCCGCGGATCTGCCGGTCTCCCTATAGTGAGTCGTATTAATTTCGATAAGCCAGGTTAACCTGCATTAATGAATCGGCCAACGCGCGGGGAGAGGCGGTTTG
CGTATTGGGCGCTCTTCCGCTTCCTCGCTCACTGACTCGCTGCGCTCGGTCGTTCGGCTGCGGCGAGCGGTATCAGCTCACTCAAAGGCGGTAATACGGTTATCCACAGAA
TCAGGGGATAACGCAGGAAAGAACATGTGAGCAAAAGGCCAGCAAAAGGCCAGGAACCGTAAAAAGGCCGCGTTGCTGGCGTTTTTCCATAGGCTCCGCCCCCCTGACGA
GCATCACAAAAATCGACGCTCAAGTCAGAGGTGGCGAAACCCGACAGGACTATAAAGATACCAGGCGTTTCCCCCTGGAAGCTCCCTCGTGCGCTCTCCTGTTCCGACCCT
GCCGCTTACCGGATACCTGTCCGCCTTTCTCCCTTCGGGAAGCGTGGCGCTTTCTCATAGCTCACGCTGTAGGTATCTCAGTTCGGTGTAGGTCGTTCGCTCCAAGCTGGG
CTGTGTGCACGAACCCCCCGTTCAGCCCGACCGCTGCGCCTTATCCGGTAACTATCGTCTTGAGTCCAACCCGGTAAGACACGACTTATCGCCACTGGCAGCAGCCACTGG
TAACAGGATTAGCAGAGCGAGGTATGTAGGCGGTGCTACAGAGTTCTTGAAGTGGTGGCCTAACTACGGCTACACTAGAAGAACAGTATTTGGTATCTGCGCTCTGCTGAA
GCCAGTTACCTTCGGAAAAAGAGTTGGTAGCTCTTGATCCGGCAAACAAACCACCGCTGGTAGCGGTGGTTTTTTTGTTTGCAAGCAGCAGATTACGCGCAGAAAAAAAGGA
TCTCAA

*TPK1* promoter; GFP+GGGGlinker; *TPK1* ORF; *HIS1* marker (replaced with a *SAT1* marker in pLC1402); *TPK1* 3’ HR

> pLC1375_pFA-GFP-TPK2-HIS

GAAGATCCTTTGATCTTTTCTACGGGGTCTGACGCTCAGTGGAACGAAAACTCACGTTAAGGGATTTTGGTCATGAGATTATCAAAAAGGATCTTCACCTAGATCCTTTTAAAT
TAAAAATGAAGTTTTAAATCAATCTAAAGTATATATGAGTAAACTTGGTCTGACAGTTACCAATGCTTAATCAGTGAGGCACCTATCTCAGCGATCTGTCTATTTCGTTCATCCA
TAGTTGCCTGACTCCCCGTCGTGTAGATAACTACGATACGGGAGGGCTTACCATCTGGCCCCAGTGCTGCAATGATACCGCGAGACCCACGCTCACCGGCTCCAGATTTAT
CAGCAATAAACCAGCCAGCCGGAAGGGCCGAGCGCAGAAGTGGTCCTGCAACTTTATCCGCCTCCATCCAGTCTATTAATTGTTGCCGGGAAGCTAGAGTAAGTAGTTCGC
CAGTTAATAGTTTGCGCAACGTTGTTGCCATTGCTACAGGCATCGTGGTGTCACGCTCGTCGTTTGGTATGGCTTCATTCAGCTCCGGTTCCCAACGATCAAGGCGAGTTAC
ATGATCCCCCATGTTGTGCAAAAAAGCGGTTAGCTCCTTCGGTCCTCCGATCGTTGTCAGAAGTAAGTTGGCCGCAGTGTTATCACTCATGGTTATGGCAGCACTGCATAATT
CTCTTACTGTCATGCCATCCGTAAGATGCTTTTCTGTGACTGGTGAGTACTCAACCAAGTCATTCTGAGAATAGTGTATGCGGCGACCGAGTTGCTCTTGCCCGGCGTCAATA
CGGGATAATACCGCGCCACATAGCAGAACTTTAAAAGTGCTCATCATTGGAAAACGTTCTTCGGGGCGAAAACTCTCAAGGATCTTACCGCTGTTGAGATCCAGTTCGATGT
AACCCACTCGTGCACCCAACTGATCTTCAGCATCTTTTACTTTCACCAGCGTTTCTGGGTGAGCAAAAACAGGAAGGCAAAATGCCGCAAAAAAGGGAATAAGGGCGACACG
GAAATGTTGAATACTCATACTCTTCCTTTTTCAATATTATTGAAGCATTTATCAGGGTTATTGTCTCATGAGCGGATACATATTTGAATGTATTTAGAAAAATAAACAAATAGGG
GTTCCGCGCACATTTCCCCGAAAAGTGCCACCTGACGTCTAAGAAACCATTATTATCATGACATTAACCTATAAAAATAGGCGTATCACGAGGCCCTTTCGTCTCGCGCGTTT
CGGTGATGACGGTGAAAACCTCTGACACATGCAGCTCCCGGAGACGGTCACAGCTTGTCTGTAAGCGGATGCCGGGAGCAGACAAGCCCGTCAGGGCGCGTCAGCGGGT
GTTGGCGGGTGTCGGGGCTGGCTTAACTATGCGGCATCAGAGCAGATTGTACTGAGAGTGCACCATATGGACATATTGTCGTTAGAACGCGGCTACAATTAATACATAACCT
TATGTATCATACACATACGATTTAGGTGACACTATAGAACGCGGCCGCCAGCTGAAGCTTCGTACGCTGCAGGTCGACGGATCCACATTGTTAAAAGTCGATACTTCAGTTG
CTTTTTTTTATTATTTTCTATCCAGCAAAAAACACATTGTCATTGTTTGTGTATCATTTTTTGTCCAGATAACAAACACCAAATTGAGGTCAACTAAACAACAACTTGTATAGTTCT
ACCACACTATTTTGAAGAGTCAGCTACAGTTCCTAGTTTCTCAACATAATATATCAACCTATCAATACTCGGATTGCAGGTCAATTTAGTTTACAACCACCAATTGCCAAAGTAT
TGGATTCTACTTTTTCCGACTTGTAAGTGAACACAAAGAAGATTAGACAAACAATCACCCACTCACACCTACTACTCACCCAATTTCCATTCCACCTTTTTTTTTCGAAAAAAAT
AACCTTCCCCCTTTTTGATGGTGAATCTTTTAAAGAAACTTCACATCACCAAGCTGCATCAACTGAATCAATCCAATTCGGACAGTAATTCCTTAAACTCAAACACATCAATGTC
TAAAGGTGAAGAATTATTCACTGGTGTTGTCCCAATTTTGGTTGAATTAGATGGTGATGTTAATGGTCACAAATTTTCTGTCTCCGGTGAAGGTGAAGGTGATGCTACTTACGG
TAAATTGACCTTAAAATTTATTTGTACTACTGGTAAATTGCCAGTTCCATGGCCAACCTTAGTCACTACTTTCGGTTATGGTGTTCAATGTTTTGCGAGATACCCAGATCATATG
AAACAACATGACTTTTTCAAGTCTGCCATGCCAGAAGGTTATGTTCAAGAAAGAACTATTTTTTTCAAAGATGACGGTAACTACAAGACCAGAGCTGAAGTCAAGTTTGAAGGT
GATACCTTAGTTAATAGAATCGAATTAAAAGGTATTGATTTTAAAGAAGATGGTAACATTTTAGGTCACAAATTGGAATACAACTATAACTCTCACAATGTTTACATCATGGCTG
ACAAACAAAAGAATGGTATCAAAGTTAACTTCAAAATTAGACACAACATTGAAGATGGTTCTGTTCAATTAGCTGACCATTATCAACAAAATACTCCAATTGGTGATGGTCCAG
TCTTGTTACCAGACAACCATTACTTATCCACTCAATCTGCCTTATCCAAAGATCCAAACGAAAAGAGAGACCACATGGTCTTGTTAGAATTTGTTACTGCTGCTGGTATTACCC
ATGGTATGGATGAATTGTACAAAGGTGGTGGTGGTGACAATCATCAACAACAACAGCAGCTTCAGCATCAGCAATATCAGCAACAATTTCAACAACCGCAGCAACAACTTTAT
CCAGGCGAACAAATAGTTCACCCTGCTGCAGCCCAAACTGGACAAAATACTACAAATGTAACAGCAGTGTCGAGCTCCAACATTACCCAATCTGCTACCTCATCACTCCATTC
ACAGCAATTGCAGCATGTAGATGTTTCCAAATCGGCTGCTGAAGAAGCCATCAGAAGATCGTTGTTGCCTGAACGTTCTACTGTTTCAAAGGGGAAATACTCTTTGACTGATT
TCTCAATTATGAGAACTTTGGGAACAGGTTCCTTTGGTAGAGTACATTTAGTGAGATCAGTTCACAATGGTAGATATTATGCTATCAAAGTTTTGAAGAAGCATCAAGTTGTGA
AAATGAAGCAAGTTGAACACACAAATGATGAAAGAAGAATGTTGAAGTTGGTTGAACATCCATTTTTGATTAGAATGTGGGGGACTTTCCAAGATTCCAAAAATTTATTTATGG
TTATGGACTATATTGAAGGGGGAGAGTTATTTTCATTGTTAAGAAAGTCTCAGAGATTTCCCAATCCAGTAGCCAAGTTTTATGCTGCAGAGGTTACCTTGGCTTTGGAGTATT
TGCACAGTCATGATATTATCTATCGTGATTTGAAACCAGAAAATATATTATTGGATAGAAATGGTCATATAAAGATTACAGATTTTGGATTTGCCAAAGAAGTTAGTACCGTTAC
ATGGACGTTATGTGGTACCCCCGATTATATTGCCCCAGAAGTCATAACAACCAAACCCTATAACAAGTCGGTGGACTGGTGGTCGTTGGGGGTATTGATTTTTGAAATGTTG
GCAGGTTACACTCCATTTTATGATTCGACTCCAATGAAAACTTATGAAAAAATTTTGGCAGGCAAAATACATTACCCAAGTTTTTTTCAACCTGATGTGATTGATTTGTTGACTA
AATTAATAACTGCTGATTTGACAAGAAGATTGGGTAATTTGATCAACGGACCAGCAGATATCAGAAATCATCCCTGGTTTCTGGAAGTGGTATGGGAGAAATTGTTGGCAAAG
GATATTGAAACTCCTTATGAACCACCAATCACAGCTGGGGTTGGTGATTCGTCATTGTTTGACCATTATCCAGAAGAACAATTAGACTACGGAAGCCAAGGAGAAGATCCTTA
TGCTCTGTATTTCCTTGACTTTTGAATTGAGAGGAGATGAACTTTCATAAATGAATAAATGAGGCGCGCCATTATAAGTAAATGCATGTATACTAAACTCACAAATTAGAGCTTC
AATTTAATTATATCAGTTATTACCCGGGAATCTCGGTCGTAATGATTTCTATAATGACGAAAAAAAAAAAAATTGGAAAGAAAAAGCTTCATGGCCTTTATAAAAAGGAACTATCC
AATACCTCGCCAGAACCAAGTAACAGTATTTTACGGGGCACAAATCAAGAACAATAAGACAGGACTGTAAAGATGGACGCATAGATCCTGGAGGATGAGGAGACAGAAGTTA
GTAGTAACAATTTGGGGACAGAAGAGGATACAGTGACATCAGAGGAAGAAGACAGCGAAGGTATAGTCAAAAAGCAATAGGACAGCAAGACAATTTATTTATTTAATAGTTTT
TTATAAATGTAAAATCTATCACAACATGTGTACTTAATTTTCTGTGGTAGGGGAGGCCATTTCGTTGAAACGTGTCGCGAGCGGTTGTGAGAATTGACTCATTTTGATTATTAC
GCAACCATTGAAATTTTTTATTTTTTTTTGGTGAAGATTTTTCCCACACAACTTCTTCTTTTACTTAACCACCAACTACCGATAATGGATTTAGTCAATCATTTACCAGACCGTTT
GTTATTTGCTGTTCCTAAAAAGGGCAGATTATACGAAAAATGCTGTAACTTATTGAGTGGTGCCGATATACAGTTTAGAAGATCTAATAGATTAGATATAGCACTTTCTACAAAC
TTGCCAATTGCATTAATCTTCTTGCCTGCAGCTGATATCCCAGTTTTCGTTGGAGAAGGTAATTGTGACTTGGGTATAACTGGGTTAGACCAAATCAAAGAAGCTGAACAATTC
GACAACATCGAGGACTTGTTGGATTTGAAATTTGGTTCATGCAAATTGCAGATCCAAGTTCCAGCAGATGGCGAGTACGAAAAGCCAGAACAGCTTGTTGGAAAGAAAATTG
TGTCTTCATTTACAAAATTGAGTACCGACTATTTCAAACAATTGTCAGACAAACCTACTAATATCAGATATGTCGGTGGTTCCGTTGAGGCTTCTTGTGCCTTGGGTGTTGCTG
ATGCTATTGTCGATTTGGTTGAAAGTGGTGAAACTATGAAAGCAGCTGGGTTAAAGGCCATAGAAACCATATTGGAGACTTCGGCTCATTTGATTTCGTCAAAAAAAAGTAAA
TTCCCAGAAATGGTCAATATAATCGTCCAAAGACTTCAAGGTGTTTTAGCTGCCCAGGAATATGTCTTGTGTAACTACAATGCCCCAAAATCCATCCAAGCAAAATGTTTAACC
ATTACTCCAGGCAGAAGGGCCGCTACTGTCTCTACTTTGGACAAACACAGCGACGACGAAGAGGACTGGGTTGCCATCTCATCCATGGTTAATAGAAAGGAAATCGGTAATG
TAATGGACGAATTGAAGAAAGCTGGTGCAACCGATATATTGGTACTCGAGATATCAAATTGTAGAGTTTAAAAGAAGTGATAGTTTCTCATAAATATTATACATATATACTTATA
GATCTACTTCAATTTGACTCAATCTGTCTTATACCATCTTTATCTACCACCTTGATGTACACACCTTTAAAGTCAATAGGCATTCTCGTTTAAACGAGCTCAACATGTTGACTCT
TCCTTGTCTGCTTTGTTTTCGGTATTTTTTTTTTATTTCTTTGCCTTTATGTATCTATATTAAATGTACAAAATATATTACATGATATTCCAAATATCTTGCTTTCTTAAAAGTCTTT
GAAAACTCAAACTAGCTATGGCCCTGTTGTATTCAATAAAAATTACAATGCATGATAGTAAACACACTAACAATACCCTTGACATGAGTCAAAATCAACCGCGGATCTGCCGGT
CTCCCTATAGTGAGTCGTATTAATTTCGATAAGCCAGGTTAACCTGCATTAATGAATCGGCCAACGCGCGGGGAGAGGCGGTTTGCGTATTGGGCGCTCTTCCGCTTCCTCG
CTCACTGACTCGCTGCGCTCGGTCGTTCGGCTGCGGCGAGCGGTATCAGCTCACTCAAAGGCGGTAATACGGTTATCCACAGAATCAGGGGATAACGCAGGAAAGAACAT
GTGAGCAAAAGGCCAGCAAAAGGCCAGGAACCGTAAAAAGGCCGCGTTGCTGGCGTTTTTCCATAGGCTCCGCCCCCCTGACGAGCATCACAAAAATCGACGCTCAAGTC
AGAGGTGGCGAAACCCGACAGGACTATAAAGATACCAGGCGTTTCCCCCTGGAAGCTCCCTCGTGCGCTCTCCTGTTCCGACCCTGCCGCTTACCGGATACCTGTCCGCCT
TTCTCCCTTCGGGAAGCGTGGCGCTTTCTCATAGCTCACGCTGTAGGTATCTCAGTTCGGTGTAGGTCGTTCGCTCCAAGCTGGGCTGTGTGCACGAACCCCCCGTTCAGC
CCGACCGCTGCGCCTTATCCGGTAACTATCGTCTTGAGTCCAACCCGGTAAGACACGACTTATCGCCACTGGCAGCAGCCACTGGTAACAGGATTAGCAGAGCGAGGTATG
TAGGCGGTGCTACAGAGTTCTTGAAGTGGTGGCCTAACTACGGCTACACTAGAAGAACAGTATTTGGTATCTGCGCTCTGCTGAAGCCAGTTACCTTCGGAAAAAGAGTTGG
TAGCTCTTGATCCGGCAAACAAACCACCGCTGGTAGCGGTGGTTTTTTTGTTTGCAAGCAGCAGATTACGCGCAGAAAAAAAGGATCTCAA

*TPK2* promoter; GFP+GGGGlinker; *TPK2* ORF; *HIS1* marker (replaced with a *SAT1* marker in pLC1406); *TPK2* 3’ HR

>pLC1527_pFA-NLS-GFP-TPK1-HIS

GAAGATCCTTTGATCTTTTCTACGGGGTCTGACGCTCAGTGGAACGAAAACTCACGTTAAGGGATTTTGGTCATGAGATTATCAAAAAGGATCTTCACCTAGATCCTTTTAAAT
TAAAAATGAAGTTTTAAATCAATCTAAAGTATATATGAGTAAACTTGGTCTGACAGTTACCAATGCTTAATCAGTGAGGCACCTATCTCAGCGATCTGTCTATTTCGTTCATCCA
TAGTTGCCTGACTCCCCGTCGTGTAGATAACTACGATACGGGAGGGCTTACCATCTGGCCCCAGTGCTGCAATGATACCGCGAGACCCACGCTCACCGGCTCCAGATTTAT
CAGCAATAAACCAGCCAGCCGGAAGGGCCGAGCGCAGAAGTGGTCCTGCAACTTTATCCGCCTCCATCCAGTCTATTAATTGTTGCCGGGAAGCTAGAGTAAGTAGTTCGC
CAGTTAATAGTTTGCGCAACGTTGTTGCCATTGCTACAGGCATCGTGGTGTCACGCTCGTCGTTTGGTATGGCTTCATTCAGCTCCGGTTCCCAACGATCAAGGCGAGTTAC
ATGATCCCCCATGTTGTGCAAAAAAGCGGTTAGCTCCTTCGGTCCTCCGATCGTTGTCAGAAGTAAGTTGGCCGCAGTGTTATCACTCATGGTTATGGCAGCACTGCATAATT
CTCTTACTGTCATGCCATCCGTAAGATGCTTTTCTGTGACTGGTGAGTACTCAACCAAGTCATTCTGAGAATAGTGTATGCGGCGACCGAGTTGCTCTTGCCCGGCGTCAATA
CGGGATAATACCGCGCCACATAGCAGAACTTTAAAAGTGCTCATCATTGGAAAACGTTCTTCGGGGCGAAAACTCTCAAGGATCTTACCGCTGTTGAGATCCAGTTCGATGT
AACCCACTCGTGCACCCAACTGATCTTCAGCATCTTTTACTTTCACCAGCGTTTCTGGGTGAGCAAAAACAGGAAGGCAAAATGCCGCAAAAAAGGGAATAAGGGCGACACG
GAAATGTTGAATACTCATACTCTTCCTTTTTCAATATTATTGAAGCATTTATCAGGGTTATTGTCTCATGAGCGGATACATATTTGAATGTATTTAGAAAAATAAACAAATAGGG
GTTCCGCGCACATTTCCCCGAAAAGTGCCACCTGACGTCTAAGAAACCATTATTATCATGACATTAACCTATAAAAATAGGCGTATCACGAGGCCCTTTCGTCTCGCGCGTTT
CGGTGATGACGGTGAAAACCTCTGACACATGCAGCTCCCGGAGACGGTCACAGCTTGTCTGTAAGCGGATGCCGGGAGCAGACAAGCCCGTCAGGGCGCGTCAGCGGGT
GTTGGCGGGTGTCGGGGCTGGCTTAACTATGCGGCATCAGAGCAGATTGTACTGAGAGTGCACCATATGGACATATTGTCGTTAGAACGCGGCTACAATTAATACATAACCT
TATGTATCATACACATACGATTTAGGTGACACTATAGAACGCGGCCGCCAGCTGAAGCTTCGTACGCTGCAGGTCGACGGATCCAGACAGAAGATAATATTTTTTCTTAACTG
TCACGCAAAAGTTTTAACTAACTAACTAACTAACTAATTAGACCAATTCCCCTAAAGGACTTAAGGACTAAAATAAGCTTATTTCAATTAATATTATCATTGAATAATAGATACTT
ATAGCAGTTATAGTAGAATTTAATTTCTTTTTTTTTTTCTTTCTTTCTTTTGTCACACATTTATCCGTTTTGACAGACTAAATTAAACAAAAAATTTTTGAACATCAGGGAACGACC
ATATACAAATAAAAGCAGAAGAAAAAAACGGAAGAAGAAGAAGAAGAAAAAAAAGTCAACCAAAAAATTGAACAAGAGGTCTAAAGAACCACAACATTATACAAAGAAAAGA
AATTAAATTAAATTACATTAAAAGAGAATTGAAACATAACACTTGAAGAAGAAAATAAAAGTATTTTTATATTATATATTCCGACTAGTATTATCTGAGATCAATATATCCTATCGT
TATCCTCCTCCTTCTCCTTTCAACTTTTGAAAAAGGTGATATTATTCAACACTGTTTTTGTTTTATCAACCAAAACCAGGATGGGTGCTCCTCCAAAAAAGAAGAGAAAGGTAG
GTACTGGTGGTGGTTCTAAAGGTGAAGAATTATTCACTGGTGTTGTCCCAATTTTGGTTGAATTAGATGGTGATGTTAATGGTCACAAATTTTCTGTCTCCGGTGAAGGTGAA
GGTGATGCTACTTACGGTAAATTGACCTTAAAATTTATTTGTACTACTGGTAAATTGCCAGTTCCATGGCCAACCTTAGTCACTACTTTCGGTTATGGTGTTCAATGTTTTGCGA
GATACCCAGATCATATGAAACAACATGACTTTTTCAAGTCTGCCATGCCAGAAGGTTATGTTCAAGAAAGAACTATTTTTTTCAAAGATGACGGTAACTACAAGACCAGAGCTG
AAGTCAAGTTTGAAGGTGATACCTTAGTTAATAGAATCGAATTAAAAGGTATTGATTTTAAAGAAGATGGTAACATTTTAGGTCACAAATTGGAATACAACTATAACTCTCACAA
TGTTTACATCATGGCTGACAAACAAAAGAATGGTATCAAAGTTAACTTCAAAATTAGACACAACATTGAAGATGGTTCTGTTCAATTAGCTGACCATTATCAACAAAATACTCCA
ATTGGTGATGGTCCAGTCTTGTTACCAGACAACCATTACTTATCCACTCAATCTGCCTTATCCAAAGATCCAAACGAAAAGAGAGACCACATGGTCTTGTTAGAATTTGTTACT
GCTGCTGGTATTACCCATGGTATGGATGAATTGTACAAAGGTGGTGGTGGTACATCCATGGAACCAGCAGACACAAGCATCAGGTCATTAAACGACATCAACTTACAAGAAC
TTGCCAACAAACAACATCAACGAAATATATATGCTCAAGGATCACCTACACTAGAAGATTCAATAACATCAAATGGTAATGATATTAATAATCCCAACAATAATATAAATAAAAC
CAATAATAACAGTAATTCAAGTCTTTCCTTGACCAAGAAAACATCAAGACATATCAATAAAGACACTACAACTAAAGGCAAATATACATTAAATGATTTTCAAATATTACGAACAT
TAGGAACAGGTTCCTTTGGTAGAGTTCATTTAACTCGATCGATTCATAATGGTAGATTTTATGCTATGAAAGTATTAAAGAAACAACGAGTAGTACAAATGAAACAAATCGAAC
ATACTAATGATGAAAGAAGAATGTTAAAATTAGCTCAACATCCATTTATAATTCGAATGTGGGGGACATTCCAAGATTGTCATAATTTATTTATGATTATGGATTATATAGAAGG
AGGTGAATTATTTTCATTATTAAGAAAATCCCAACGATTCCCTACTCCAGTAGCTAAATTTTATGCTGCCGAAGTATTTTTGGCTATTGAATATTTACACAGTTTAGACATTATTT
ATCGAGATTTAAAACCAGAAAATATATTATTGGATAAAAATGGTCATATCAAATTAACTGATTTTGGATTTGCTAAAGAAGTTCAAGATGTGACTTATACTTTATGTGGGACTCC
AGATTATATTGCTCCTGAAGTGGTGGCAACAAAACCTTATAATAAATCAGTTGATTGGTGGTCATTTGGTATATTAATATTTGAAATGTTAACTGGTTATACTCCATTCTATGAT
CCTACTCCAATGAAAACTTATGAAAACATTTTAAATGGATCAATTACTTATCCTGATTATTTACCTCCAGATATATTGGATCTTCTACAAAAATTAATTGTCAAAGATTTAACTCA
AAGATTAGGTAATTTACAAGGTGGTTCTGATGATGTGAAAAATCATCCGTGGTTTAAAGAAGTTATTTGGGAAAGATTGTTGTCACGTGATATAGAAACTCCATATGAACCACC
TATAACTTCTGGTGTTGGTGATACTTCACAATTTGATCGATACCCAGAAGATAAAGATTTGGATTATGGTATAAGTGGAGTTGAAGACCCATATCGTGATCAATTCCAGGACTT
TTAAATGTAAAAATAAAATTGTATTTATTGGCACAACAATGTTAATTATGATAACTAGTTTTATAGATGTATAATTAATTTGCAAGAAAGAAGAAGACGAAGATTTGATTAGTGAA
TTTTTTAAAGCTAAAAAAACACTTCTTGGTGTATCAAAAGTAATTTTGGCAAATCTTATAGACAGCTAAAATATAAGCAGTGTCTTATTTTTAAACCAATGATCACTATATGAATT
GGCTTCCGGCGCGCCATTATAAGTAAATGCATGTATACTAAACTCACAAATTAGAGCTTCAATTTAATTATATCAGTTATTACCCGGGAATCTCGGTCGTAATGATTTCTATAAT
GACGAAAAAAAAAAAAATTGGAAAGAAAAAGCTTCATGGCCTTTATAAAAAGGAACTATCCAATACCTCGCCAGAACCAAGTAACAGTATTTTACGGGGCACAAATCAAGAACA
ATAAGACAGGACTGTAAAGATGGACGCATAGATCCTGGAGGATGAGGAGACAGAAGTTAGTAGTAACAATTTGGGGACAGAAGAGGATACAGTGACATCAGAGGAAGAAGA
CAGCGAAGGTATAGTCAAAAAGCAATAGGACAGCAAGACAATTTATTTATTTAATAGTTTTTTATAAATGTAAAATCTATCACAACATGTGTACTTAATTTTCTGTGGTAGGGGA
GGCCATTTCGTTGAAACGTGTCGCGAGCGGTTGTGAGAATTGACTCATTTTGATTATTACGCAACCATTGAAATTTTTTATTTTTTTTTGGTGAAGATTTTTCCCACACAACTTC
TTCTTTTACTTAACCACCAACTACCGATAATGGATTTAGTCAATCATTTACCAGACCGTTTGTTATTTGCTGTTCCTAAAAAGGGCAGATTATACGAAAAATGCTGTAACTTATT
GAGTGGTGCCGATATACAGTTTAGAAGATCTAATAGATTAGATATAGCACTTTCTACAAACTTGCCAATTGCATTAATCTTCTTGCCTGCAGCTGATATCCCAGTTTTCGTTGG
AGAAGGTAATTGTGACTTGGGTATAACTGGGTTAGACCAAATCAAAGAAGCTGAACAATTCGACAACATCGAGGACTTGTTGGATTTGAAATTTGGTTCATGCAAATTGCAGA
TCCAAGTTCCAGCAGATGGCGAGTACGAAAAGCCAGAACAGCTTGTTGGAAAGAAAATTGTGTCTTCATTTACAAAATTGAGTACCGACTATTTCAAACAATTGTCAGACAAA
CCTACTAATATCAGATATGTCGGTGGTTCCGTTGAGGCTTCTTGTGCCTTGGGTGTTGCTGATGCTATTGTCGATTTGGTTGAAAGTGGTGAAACTATGAAAGCAGCTGGGTT
AAAGGCCATAGAAACCATATTGGAGACTTCGGCTCATTTGATTTCGTCAAAAAAAAGTAAATTCCCAGAAATGGTCAATATAATCGTCCAAAGACTTCAAGGTGTTTTAGCTGC
CCAGGAATATGTCTTGTGTAACTACAATGCCCCAAAATCCATCCAAGCAAAATGTTTAACCATTACTCCAGGCAGAAGGGCCGCTACTGTCTCTACTTTGGACAAACACAGCG
ACGACGAAGAGGACTGGGTTGCCATCTCATCCATGGTTAATAGAAAGGAAATCGGTAATGTAATGGACGAATTGAAGAAAGCTGGTGCAACCGATATATTGGTACTCGAGAT
ATCAAATTGTAGAGTTTAAAAGAAGTGATAGTTTCTCATAAATATTATACATATATACTTATAGATCTACTTCAATTTGACTCAATCTGTCTTATACCATCTTTATCTACCACCTTG
ATGTACACACCTTTAAAGTCAATAGGCATTCTCGTTTAAACGAGCTCCACAATAGTTCAATATTGGAATCAAACATAATAGCGAGAATTGGTTTTTATCTGAGTACCACATTGA
GAATGGTATTGGATGTTATTAAGTTGCCATTTTTGAATGAACTTCTCACTGATGATTCTTTTTATTAAATCCAGGGTCCTTTTTTTAGGGTATTCTACTAAAACTTGATATTTGGG
ATGATTTGGTAGTTCTATTTTGATTTCCACTAATGAAAAGCATTTCTAACAAGTTTTGGAAATTTGACTGAGATGTGCATTACTAAGAGCTTCGTTGTTACGGTGATACTAAAGT
TTGTCAAGTCTCTTAAACATTACATTGAATACTATTTGATAGTTGGTGATAAAAAAAAACTCCGTAAACAATAATTTCGTGTTTAATTCACATTTTATTGGAGCATGAAAATTATTTA
ACCGAACGCGTTCTTGAATGTGTATAAAAATAGAAATCCCATATTATCGAATTAGAGGTCTCCGCGGATCTGCCGGTCTCCCTATAGTGAGTCGTATTAATTTCGATAAGCCA
GGTTAACCTGCATTAATGAATCGGCCAACGCGCGGGGAGAGGCGGTTTGCGTATTGGGCGCTCTTCCGCTTCCTCGCTCACTGACTCGCTGCGCTCGGTCGTTCGGCTGC
GGCGAGCGGTATCAGCTCACTCAAAGGCGGTAATACGGTTATCCACAGAATCAGGGGATAACGCAGGAAAGAACATGTGAGCAAAAGGCCAGCAAAAGGCCAGGAACCGT
AAAAAGGCCGCGTTGCTGGCGTTTTTCCATAGGCTCCGCCCCCCTGACGAGCATCACAAAAATCGACGCTCAAGTCAGAGGTGGCGAAACCCGACAGGACTATAAAGATAC
CAGGCGTTTCCCCCTGGAAGCTCCCTCGTGCGCTCTCCTGTTCCGACCCTGCCGCTTACCGGATACCTGTCCGCCTTTCTCCCTTCGGGAAGCGTGGCGCTTTCTCATAGC
TCACGCTGTAGGTATCTCAGTTCGGTGTAGGTCGTTCGCTCCAAGCTGGGCTGTGTGCACGAACCCCCCGTTCAGCCCGACCGCTGCGCCTTATCCGGTAACTATCGTCTT
GAGTCCAACCCGGTAAGACACGACTTATCGCCACTGGCAGCAGCCACTGGTAACAGGATTAGCAGAGCGAGGTATGTAGGCGGTGCTACAGAGTTCTTGAAGTGGTGGCC
TAACTACGGCTACACTAGAAGAACAGTATTTGGTATCTGCGCTCTGCTGAAGCCAGTTACCTTCGGAAAAAGAGTTGGTAGCTCTTGATCCGGCAAACAAACCACCGCTGGT
AGCGGTGGTTTTTTTGTTTGCAAGCAGCAGATTACGCGCAGAAAAAAAGGATCTCAA

*TPK1* promoter; SV40 NLS; GFP+GGGGlinker; *TPK1* ORF; *HIS1* marker (replaced with a *SAT1* marker in pLC1529); *TPK1* 3' HR

> pLC1528_pFA-NLS-GFP-TPK2-HIS

GAAGATCCTTTGATCTTTTCTACGGGGTCTGACGCTCAGTGGAACGAAAACTCACGTTAAGGGATTTTGGTCATGAGATTATCAAAAAGGATCTTCACCTAGATCCTTTTAAAT
TAAAAATGAAGTTTTAAATCAATCTAAAGTATATATGAGTAAACTTGGTCTGACAGTTACCAATGCTTAATCAGTGAGGCACCTATCTCAGCGATCTGTCTATTTCGTTCATCCA
TAGTTGCCTGACTCCCCGTCGTGTAGATAACTACGATACGGGAGGGCTTACCATCTGGCCCCAGTGCTGCAATGATACCGCGAGACCCACGCTCACCGGCTCCAGATTTAT
CAGCAATAAACCAGCCAGCCGGAAGGGCCGAGCGCAGAAGTGGTCCTGCAACTTTATCCGCCTCCATCCAGTCTATTAATTGTTGCCGGGAAGCTAGAGTAAGTAGTTCGC
CAGTTAATAGTTTGCGCAACGTTGTTGCCATTGCTACAGGCATCGTGGTGTCACGCTCGTCGTTTGGTATGGCTTCATTCAGCTCCGGTTCCCAACGATCAAGGCGAGTTAC
ATGATCCCCCATGTTGTGCAAAAAAGCGGTTAGCTCCTTCGGTCCTCCGATCGTTGTCAGAAGTAAGTTGGCCGCAGTGTTATCACTCATGGTTATGGCAGCACTGCATAATT
CTCTTACTGTCATGCCATCCGTAAGATGCTTTTCTGTGACTGGTGAGTACTCAACCAAGTCATTCTGAGAATAGTGTATGCGGCGACCGAGTTGCTCTTGCCCGGCGTCAATA
CGGGATAATACCGCGCCACATAGCAGAACTTTAAAAGTGCTCATCATTGGAAAACGTTCTTCGGGGCGAAAACTCTCAAGGATCTTACCGCTGTTGAGATCCAGTTCGATGT
AACCCACTCGTGCACCCAACTGATCTTCAGCATCTTTTACTTTCACCAGCGTTTCTGGGTGAGCAAAAACAGGAAGGCAAAATGCCGCAAAAAAGGGAATAAGGGCGACACG
GAAATGTTGAATACTCATACTCTTCCTTTTTCAATATTATTGAAGCATTTATCAGGGTTATTGTCTCATGAGCGGATACATATTTGAATGTATTTAGAAAAATAAACAAATAGGG
GTTCCGCGCACATTTCCCCGAAAAGTGCCACCTGACGTCTAAGAAACCATTATTATCATGACATTAACCTATAAAAATAGGCGTATCACGAGGCCCTTTCGTCTCGCGCGTTT
CGGTGATGACGGTGAAAACCTCTGACACATGCAGCTCCCGGAGACGGTCACAGCTTGTCTGTAAGCGGATGCCGGGAGCAGACAAGCCCGTCAGGGCGCGTCAGCGGGT
GTTGGCGGGTGTCGGGGCTGGCTTAACTATGCGGCATCAGAGCAGATTGTACTGAGAGTGCACCATATGGACATATTGTCGTTAGAACGCGGCTACAATTAATACATAACCT
TATGTATCATACACATACGATTTAGGTGACACTATAGAACGCGGCCGCCAGCTGAAGCTTCGTACGCTGCAGGTCGACGGATCCACATTGTTAAAAGTCGATACTTCAGTTG
CTTTTTTTTATTATTTTCTATCCAGCAAAAAACACATTGTCATTGTTTGTGTATCATTTTTTGTCCAGATAACAAACACCAAATTGAGGTCAACTAAACAACAACTTGTATAGTTCT
ACCACACTATTTTGAAGAGTCAGCTACAGTTCCTAGTTTCTCAACATAATATATCAACCTATCAATACTCGGATTGCAGGTCAATTTAGTTTACAACCACCAATTGCCAAAGTAT
TGGATTCTACTTTTTCCGACTTGTAAGTGAACACAAAGAAGATTAGACAAACAATCACCCACTCACACCTACTACTCACCCAATTTCCATTCCACCTTTTTTTTTCGAAAAAAAT
AACCTTCCCCCTTTTTGATGGTGAATCTTTTAAAGAAACTTCACATCACCAAGCTGCATCAACTGAATCAATCCAATTCGGACAGTAATTCCTTAAACTCAAACACATCAATGG
GTGCTCCTCCAAAAAAGAAGAGAAAGGTAGGTACTGGTGGTGGTTCTAAAGGTGAAGAATTATTCACTGGTGTTGTCCCAATTTTGGTTGAATTAGATGGTGATGTTAATGGT
CACAAATTTTCTGTCTCCGGTGAAGGTGAAGGTGATGCTACTTACGGTAAATTGACCTTAAAATTTATTTGTACTACTGGTAAATTGCCAGTTCCATGGCCAACCTTAGTCACT
ACTTTCGGTTATGGTGTTCAATGTTTTGCGAGATACCCAGATCATATGAAACAACATGACTTTTTCAAGTCTGCCATGCCAGAAGGTTATGTTCAAGAAAGAACTATTTTTTTCA
AAGATGACGGTAACTACAAGACCAGAGCTGAAGTCAAGTTTGAAGGTGATACCTTAGTTAATAGAATCGAATTAAAAGGTATTGATTTTAAAGAAGATGGTAACATTTTAGGTC
ACAAATTGGAATACAACTATAACTCTCACAATGTTTACATCATGGCTGACAAACAAAAGAATGGTATCAAAGTTAACTTCAAAATTAGACACAACATTGAAGATGGTTCTGTTCA
ATTAGCTGACCATTATCAACAAAATACTCCAATTGGTGATGGTCCAGTCTTGTTACCAGACAACCATTACTTATCCACTCAATCTGCCTTATCCAAAGATCCAAACGAAAAGAG
AGACCACATGGTCTTGTTAGAATTTGTTACTGCTGCTGGTATTACCCATGGTATGGATGAATTGTACAAAGGTGGTGGTGACAATCATCAACAACAACAGCAGCTTCAGC
ATCAGCAATATCAGCAACAATTTCAACAACCGCAGCAACAACTTTATCCAGGCGAACAAATAGTTCACCCTGCTGCAGCCCAAACTGGACAAAATACTACAAATGTAACAGCA
GTGTCGAGCTCCAACATTACCCAATCTGCTACCTCATCACTCCATTCACAGCAATTGCAGCATGTAGATGTTTCCAAATCGGCTGCTGAAGAAGCCATCAGAAGATCGTTGTT
GCCTGAACGTTCTACTGTTTCAAAGGGGAAATACTCTTTGACTGATTTCTCAATTATGAGAACTTTGGGAACAGGTTCCTTTGGTAGAGTACATTTAGTGAGATCAGTTCACAA
TGGTAGATATTATGCTATCAAAGTTTTGAAGAAGCATCAAGTTGTGAAAATGAAGCAAGTTGAACACACAAATGATGAAAGAAGAATGTTGAAGTTGGTTGAACATCCATTTTT
GATTAGAATGTGGGGGACTTTCCAAGATTCCAAAAATTTATTTATGGTTATGGACTATATTGAAGGGGGAGAGTTATTTTCATTGTTAAGAAAGTCTCAGAGATTTCCCAATCC
AGTAGCCAAGTTTTATGCTGCAGAGGTTACCTTGGCTTTGGAGTATTTGCACAGTCATGATATTATCTATCGTGATTTGAAACCAGAAAATATATTATTGGATAGAAATGGTCA
TATAAAGATTACAGATTTTGGATTTGCCAAAGAAGTTAGTACCGTTACATGGACGTTATGTGGTACCCCCGATTATATTGCCCCAGAAGTCATAACAACCAAACCCTATAACAA
GTCGGTGGACTGGTGGTCGTTGGGGGTATTGATTTTTGAAATGTTGGCAGGTTACACTCCATTTTATGATTCGACTCCAATGAAAACTTATGAAAAAATTTTGGCAGGCAAAA
TACATTACCCAAGTTTTTTTCAACCTGATGTGATTGATTTGTTGACTAAATTAATAACTGCTGATTTGACAAGAAGATTGGGTAATTTGATCAACGGACCAGCAGATATCAGAAA
TCATCCCTGGTTTCTGGAAGTGGTATGGGAGAAATTGTTGGCAAAGGATATTGAAACTCCTTATGAACCACCAATCACAGCTGGGGTTGGTGATTCGTCATTGTTTGACCATT
ATCCAGAAGAACAATTAGACTACGGAAGCCAAGGAGAAGATCCTTATGCTCTGTATTTCCTTGACTTTTGAATTGAGAGGAGATGAACTTTCATAAATGAATAAATGAGGCGC
GCCATTATAAGTAAATGCATGTATACTAAACTCACAAATTAGAGCTTCAATTTAATTATATCAGTTATTACCCGGGAATCTCGGTCGTAATGATTTCTATAATGACGAAAAAAA
AAAATTGGAAAGAAAAAGCTTCATGGCCTTTATAAAAAGGAACTATCCAATACCTCGCCAGAACCAAGTAACAGTATTTTACGGGGCACAAATCAAGAACAATAAGACAGGAC
TGTAAAGATGGACGCATAGATCCTGGAGGATGAGGAGACAGAAGTTAGTAGTAACAATTTGGGGACAGAAGAGGATACAGTGACATCAGAGGAAGAAGACAGCGAAGGTAT
AGTCAAAAAGCAATAGGACAGCAAGACAATTTATTTATTTAATAGTTTTTATAAATGTAAAATCTATCACAACATGTGTACTTAATTTTCTGTGGTAGGGGAGGCCATTTCGTT
GAAACGTGTCGCGAGCGGTTGTGAGAATTGACTCATTTTGATTATTACGCAACCATTGAAATTTTTTATTTTTTTTTGGTGAAGATTTTCCCACACAACTTCTTCTTTTACTTAA
CCACCAACTACCGATAATGGATTTAGTCAATCATTTACCAGACCGTTTGTTATTTGCTGTTCCTAAAAAGGGCAGATTATACGAAAAATGCTGTAACTTATTGAGTGGTGCCGA
TATACAGTTTAGAAGATCTAATAGATTAGATATAGCACTTTCTACAAACTTGCCAATTGCATTAATCTTCTTGCCTGCAGCTGATATCCCAGTTTTCGTTGGAGAAGGTAATTGT
GACTTGGGTATAACTGGGTTAGACCAAATCAAAGAAGCTGAACAATTCGACAACATCGAGGACTTGTTGGATTTGAAATTTGGTTCATGCAAATTGCAGATCCAAGTTCCAGC
AGATGGCGAGTACGAAAAGCCAGAACAGCTTGTTGGAAAGAAAATTGTGTCTTCATTTACAAAATTGAGTACCGACTATTTCAAACAATTGTCAGACAAACCTACTAATATCAG
ATATGTCGGTGGTTCCGTTGAGGCTTCTTGTGCCTTGGGTGTTGCTGATGCTATTGTCGATTTGGTTGAAAGTGGTGAAACTATGAAAGCAGCTGGGTTAAAGGCCATAGAA
ACCATATTGGAGACTTCGGCTCATTTGATTTCGTCAAAAAAAAGTAAATTCCCAGAAATGGTCAATATAATCGTCCAAAGACTTCAAGGTGTTTTAGCTGCCCAGGAATATGTC
TTGTGTAACTACAATGCCCCAAAATCCATCCAAGCAAAATGTTTAACCATTACTCCAGGCAGAAGGGCCGCTACTGTCTCTACTTTGGACAAACACAGCGACGACGAAGAGG
ACTGGGTTGCCATCTCATCCATGGTTAATAGAAAGGAAATCGGTAATGTAATGGACGAATTGAAGAAAGCTGGTGCAACCGATATATTGGTACTCGAGATATCAAATTGTAGA
GTTTAAAAGAAGTGATAGTTTCTCATAAATATTATACATATATACTTATAGATCTACTTCAATTTGACTCAATCTGTCTTATACCATCTTTATCTACCACCTTGATGTACACACCTT
TAAAGTCAATAGGCATTCTCGTTTAAACGAGCTCAACATGTTGACTCTTCCTTGTCTGCTTTGTTTTTCGGTATTTTTTTTTTATTTCTTTGCCTTTATGTATCTATATTAAATGTA
CAAAATATATTACATGATATTCCAAATATCTTGCTTTCTTAAAAGTCTTTGAAAACTCAAACTAGCTATGGCCCTGTTGTATTCAATAAAAATTACAATGCATGATAGTAAACACA
CTAACAATACCCTTGACATGAGTCAAAATCAACCGCGGATCTGCCGGTCTCCCTATAGTGAGTCGTATTAATTTCGATAAGCCAGGTTAACCTGCATTAATGAATCGGCCAAC
GCGCGGGGAGAGGCGGTTTGCGTATTGGGCGCTCTTCCGCTTCCTCGCTCACTGACTCGCTGCGCTCGGTCGTTCGGCTGCGGCGAGCGGTATCAGCTCACTCAAAGGC
GGTAATACGGTTATCCACAGAATCAGGGGATAACGCAGGAAAGAACATGTGAGCAAAAGGCCAGCAAAAGGCCAGGAACCGTAAAAAGGCCGCGTTGCTGGCGTTTTTCCA
TAGGCTCCGCCCCCCTGACGAGCATCACAAAAATCGACGCTCAAGTCAGAGGTGGCGAAACCCGACAGGACTATAAAGATACCAGGCGTTTCCCCCTGGAAGCTCCCTCGT
GCGCTCTCCTGTTCCGACCCTGCCGCTTACCGGATACCTGTCCGCCTTTCTCCCTTCGGGAAGCGTGGCGCTTTCTCATAGCTCACGCTGTAGGTATCTCAGTTCGGTGTA
GGTCGTTCGCTCCAAGCTGGGCTGTGTGCACGAACCCCCCGTTCAGCCCGACCGCTGCGCCTTATCCGGTAACTATCGTCTTGAGTCCAACCCGGTAAGACACGACTTATC
GCCACTGGCAGCAGCCACTGGTAACAGGATTAGCAGAGCGAGGTATGTAGGCGGTGCTACAGAGTTCTTGAAGTGGTGGCCTAACTACGGCTACACTAGAAGAACAGTATT
TGGTATCTGCGCTCTGCTGAAGCCAGTTACCTTCGGAAAAAGAGTTGGTAGCTCTTGATCCGGCAAACAAACCACCGCTGGTAGCGGTGGTTTTTTTGTTTGCAAGCAGCAG
ATTACGCGCAGAAAAAAAGGATCTCAA

*TPK2* promoter; SV40 NLS; GFP+GGGGlinker; *TPK2* ORF; *HIS1* marker (replaced with a *SAT1* marker in pLC1530); *TPK2* 3' HR

>pLC1668_pFA-pTpk1-GFP-2N1C-HIS

GAAGATCCTTTGATCTTTTCTACGGGGTCTGACGCTCAGTGGAACGAAAACTCACGTTAAGGGATTTTGGTCATGAGATTATCAAAAAGGATCTTCACCTAGATCCTTTTAAAT
TAAAAATGAAGTTTTAAATCAATCTAAAGTATATATGAGTAAACTTGGTCTGACAGTTACCAATGCTTAATCAGTGAGGCACCTATCTCAGCGATCTGTCTATTTCGTTCATCCA
TAGTTGCCTGACTCCCCGTCGTGTAGATAACTACGATACGGGAGGGCTTACCATCTGGCCCCAGTGCTGCAATGATACCGCGAGACCCACGCTCACCGGCTCCAGATTTAT
CAGCAATAAACCAGCCAGCCGGAAGGGCCGAGCGCAGAAGTGGTCCTGCAACTTTATCCGCCTCCATCCAGTCTATTAATTGTTGCCGGGAAGCTAGAGTAAGTAGTTCGC
CAGTTAATAGTTTGCGCAACGTTGTTGCCATTGCTACAGGCATCGTGGTGTCACGCTCGTCGTTTGGTATGGCTTCATTCAGCTCCGGTTCCCAACGATCAAGGCGAGTTAC
ATGATCCCCCATGTTGTGCAAAAAAGCGGTTAGCTCCTTCGGTCCTCCGATCGTTGTCAGAAGTAAGTTGGCCGCAGTGTTATCACTCATGGTTATGGCAGCACTGCATAATT
CTCTTACTGTCATGCCATCCGTAAGATGCTTTTCTGTGACTGGTGAGTACTCAACCAAGTCATTCTGAGAATAGTGTATGCGGCGACCGAGTTGCTCTTGCCCGGCGTCAATA
CGGGATAATACCGCGCCACATAGCAGAACTTTAAAAGTGCTCATCATTGGAAAACGTTCTTCGGGGCGAAAACTCTCAAGGATCTTACCGCTGTTGAGATCCAGTTCGATGT
AACCCACTCGTGCACCCAACTGATCTTCAGCATCTTTTACTTTCACCAGCGTTTCTGGGTGAGCAAAAACAGGAAGGCAAAATGCCGCAAAAAAGGGAATAAGGGCGACACG
GAAATGTTGAATACTCATACTCTTCCTTTTTCAATATTATTGAAGCATTTATCAGGGTTATTGTCTCATGAGCGGATACATATTTGAATGTATTTAGAAAAATAAACAAATAGGG
GTTCCGCGCACATTTCCCCGAAAAGTGCCACCTGACGTCTAAGAAACCATTATTATCATGACATTAACCTATAAAAATAGGCGTATCACGAGGCCCTTTCGTCTCGCGCGTTT
CGGTGATGACGGTGAAAACCTCTGACACATGCAGCTCCCGGAGACGGTCACAGCTTGTCTGTAAGCGGATGCCGGGAGCAGACAAGCCCGTCAGGGCGCGTCAGCGGGT
GTTGGCGGGTGTCGGGGCTGGCTTAACTATGCGGCATCAGAGCAGATTGTACTGAGAGTGCACCATATGGACATATTGTCGTTAGAACGCGGCTACAATTAATACATAACCT
TATGTATCATACACATACGATTTAGGTGACACTATAGAACGCGGCCGCCAGCTGAAGCTTCGTACGCTGCAGGTCGACGGATCCAGACAGAAGATAATATTTTTTCTTAACTG
TCACGCAAAAGTTTTAACTAACTAACTAACTAACTAATTAGACCAATTCCCCTAAAGGACTTAAGGACTAAAATAAGCTTATTTCAATTAATATTATCATTGAATAATAGATACTT
ATAGCAGTTATAGTAGAATTTAATTTCTTTTTTTTTTCTTTCTTTCTTTTGTCACACATTTATCCGTTTTGACAGACTAAATTAAACAAAAAATTTTTGAACATCAGGGAACGACCA
TATACAAATAAAAGCAGAAGAAAAAAACGGAAGAAGAAGAAGAAGAAAAAAAAGTCAACCAAAAAATTGAACAAGAGGTCTAAAGAACCACAACATTATACAAAAGAAAAGAA
ATTAAATTAAATTACATTAAAAGAGAATTGAAACATAACACTTGAAGAAGAAAATAAAAGTATTTTTATATTATATATTCCGACTAGTATTATCTGAGATCAATATATCCTATCGTT
ATCCTCCTCCTTCTCCTTTCAACTTTTGAAAAAGGTGATATTATTCAACACTGTTTTTGTTTTATCAACCAAAACCAGGATGTCTAAAGGTGAAGAATTATTCACTGGTGTTGTC
CCAATTTTGGTTGAATTAGATGGTGATGTTAATGGTCACAAATTTTCTGTCTCCGGTGAAGGTGAAGGTGATGCTACTTACGGTAAATTGACCTTAAAATTTATTTGTACTACTG
GTAAATTGCCAGTTCCATGGCCAACCTTAGTCACTACTTTCGGTTATGGTGTTCAATGTTTTGCGAGATACCCAGATCATATGAAACAACATGACTTTTTCAAGTCTGCCATGC
CAGAAGGTTATGTTCAAGAAAGAACTATTTTTTTCAAAGATGACGGTAACTACAAGACCAGAGCTGAAGTCAAGTTTGAAGGTGATACCTTAGTTAATAGAATCGAATTAAAAG
GTATTGATTTTAAAGAAGATGGTAACATTTTAGGTCACAAATTGGAATACAACTATAACTCTCACAATGTTTACATCATGGCTGACAAACAAAAGAATGGTATCAAAGTTAACTT
CAAAATTAGACACAACATTGAAGATGGTTCTGTTCAATTAGCTGACCATTATCAACAAAATACTCCAATTGGTGATGGTCCAGTCTTGTTACCAGACAACCATTACTTATCCAC
TCAATCTGCCTTATCCAAAGATCCAAACGAAAAGAGAGACCACATGGTCTTGTTAGAATTTGTTACTGCTGCTGGTATTACCCATGGTATGGATGAATTGTACAAAGGTGGTG
GTGGTGACAATCATCAACAACAACAGCAGCTTCAGCATCAGCAATATCAGCAACAATTTCAACAACCGCAGCAACAACTTTATCCAGGCGAACAAATAGTTCACCCTGCTGCA
GCCCAAACTGGACAAAATACTACAAATGTAACAGCAGTGTCGAGCTCCAACATTACCCAATCTGCTACCTCATCACTCCATTCACAGCAATTGCAGCATGTAGATGTTTCCAA
ATCGGCTGCTGAAGAAGCCATCAGAAGATCGTTGTTGCCTGAACGTTCTACTGTTTCAAAAGGCAAATATACATTAAATGATTTTCAAATATTACGAACATTAGGAACAGGTTC
CTTTGGTAGAGTTCATTTAACTCGATCGATTCATAATGGTAGATTTTATGCTATGAAAGTATTAAAGAAACAACGAGTAGTACAAATGAAACAAATCGAACATACTAATGATGAA
AGAAGAATGTTAAAATTAGCTCAACATCCATTTATAATTCGAATGTGGGGGACATTCCAAGATTGTCATAATTTATTTATGATTATGGATTATATAGAAGGAGGTGAATTATTTT
CATTATTAAGAAAATCCCAACGATTCCCTACTCCAGTAGCTAAATTTTATGCTGCCGAAGTATTTTTGGCTATTGAATATTTACACAGTTTAGACATTATTTATCGAGATTTAAAA
CCAGAAAATATATTATTGGATAAAAATGGTCATATCAAATTAACTGATTTTGGATTTGCTAAAGAAGTTCAAGATGTGACTTATACTTTATGTGGGACTCCAGATTATATTGCTC
CTGAAGTGGTGGCAACAAAACCTTATAATAAATCAGTTGATTGGTGGTCATTTGGTATATTAATATTTGAAATGTTAACTGGTTATACTCCATTCTATGATCCTACTCCAATGAA
AACTTATGAAAACATTTTAAATGGATCAATTACTTATCCTGATTATTTACCTCCAGATATATTGGATCTTCTACAAAAATTAATTGTCAAAGATTTAACTCAAAGATTAGGTAATTT
ACAAGGTGGTTCTGATGATGTGAAAAATCATCCGTGGTTTAAAGAAGTTATTTGGGAAAGATTGTTGTCACGTGATATAGAAACTCCATATGAACCACCTATAACTTCTGGTGT
TGGTGATACTTCACAATTTGATCGATACCCAGAAGATAAAGATTTGGATTATGGTATAAGTGGAGTTGAAGACCCATATCGTGATCAATTCCAGGACTTTTAAATGTAAAAATA
AAATTGTATTTATTGGCACAACAATGTTAATTATGATAACTAGTTTTATAGATGTATAATTAATTTGCAAGAAAGAAGAAGACGAAGATTTGATTAGTGAATTTTTTAAAGCTAAA
AAAACACTTCTTGGTGTATCAAAAGTAATTTTGGCAAATCTTATAGACAGCTAAAATATAAGCAGTGTCTTATTTTTAAACCAATGATCACTATATGAATTGGCTTCCGGCGCGC
CATTATAAGTAAATGCATGTATACTAAACTCACAAATTAGAGCTTCAATTTAATTATATCAGTTATTACCCGGGAATCTCGGTCGTAATGATTTCTATAATGACGAAAAAAAAAAA
ATTGGAAAGAAAAAGCTTCATGGCCTTTATAAAAAGGAACTATCCAATACCTCGCCAGAACCAAGTAACAGTATTTTACGGGGCACAAATCAAGAACAATAAGACAGGACTGT
AAAGATGGACGCATAGATCCTGGAGGATGAGGAGACAGAAGTTAGTAGTAACAATTTGGGGACAGAAGAGGATACAGTGACATCAGAGGAAGAAGACAGCGAAGGTATAGT
CAAAAAGCAATAGGACAGCAAGACAATTTATTTATTTAATAGTTTTTTATAAATGTAAAATCTATCACAACATGTGTACTTAATTTTCTGTGGTAGGGGAGGCCATTTCGTTGAA
ACGTGTCGCGAGCGGTTGTGAGAATTGACTCATTTTGATTATTACGCAACCATTGAAATTTTTTATTTTTTTTTGGTGAAGATTTTCCCACACAACTTCTTCTTTTACTTAACCA
CCAACTACCGATAATGGATTTAGTCAATCATTTACCAGACCGTTTGTTATTTGCTGTTCCTAAAAAGGGCAGATTATACGAAAAATGCTGTAACTTATTGAGTGGTGCCGATAT
ACAGTTTAGAAGATCTAATAGATTAGATATAGCACTTTCTACAAACTTGCCAATTGCATTAATCTTCTTGCCTGCAGCTGATATCCCAGTTTTCGTTGGAGAAGGTAATTGTGA
CTTGGGTATAACTGGGTTAGACCAAATCAAAGAAGCTGAACAATTCGACAACATCGAGGACTTGTTGGATTTGAAATTTGGTTCATGCAAATTGCAGATCCAAGTTCCAGCAG
ATGGCGAGTACGAAAAGCCAGAACAGCTTGTTGGAAAGAAAATTGTGTCTTCATTTACAAAATTGAGTACCGACTATTTCAAACAATTGTCAGACAAACCTACTAATATCAGAT
ATGTCGGTGGTTCCGTTGAGGCTTCTTGTGCCTTGGGTGTTGCTGATGCTATTGTCGATTTGGTTGAAAGTGGTGAAACTATGAAAGCAGCTGGGTTAAAGGCCATAGAAAC
CATATTGGAGACTTCGGCTCATTTGATTTCGTCAAAAAAAAAGTAAATTCCCAGAAATGGTCAATATAATCGTCCAAAGACTTCAAGGTGTTTTAGCTGCCCAGGAATATGTCTT
GTGTAACTACAATGCCCCAAAATCCATCCAAGCAAAATGTTTAACCATTACTCCAGGCAGAAGGGCCGCTACTGTCTCTACTTTGGACAAACACAGCGACGACGAAGAGGAC
TGGGTTGCCATCTCATCCATGGTTAATAGAAAGGAAATCGGTAATGTAATGGACGAATTGAAGAAAGCTGGTGCAACCGATATATTGGTACTCGAGATATCAAATTGTAGAGT
TTAAAAGAAGTGATAGTTTCTCATAAATATTATACATATATACTTATAGATCTACTTCAATTTGACTCAATCTGTCTTATACCATCTTTATCTACCACCTTGATGTACACACCTTTA
AAGTCAATAGGCATTCTCGTTTAAACGAGCTCCACAATAGTTCAATATTGGAATCAAACATAATAGCGAGAATTGGTTTTTATCTGAGTACCACATTGAGAATGGTATTGGATG
TTATTAAGTTGCCATTTTTGAATGAACTTCTCACTGATGATTCTTTTTATTAAATCCAGGGTCCTTTTTTTAGGGTATTCTACTAAAACTTGATATTTGGGATGATTTGGTAGTTC
TATTTTGATTTCCACTAATGAAAAGCATTTCTAACAAGTTTTGGAAATTTGACTGAGATGTGCATTACTAAGAGCTTCGTTGTTACGGTGATACTAAAGTTTGTCAAGTCTCTTA
AACATTACATTGAATACTATTTGATAGTTGGTGATAAAAAAAACTCCGTAAACAATAATTTCGTGTTTAATTCACATTTTATTGGAGCATGAAAATTATTTAACCGAACGCGTTCT
TGAATGTGTATAAAAATAGAAATCCCATATTATCGAATTAGAGGTCTCCGCGGATCTGCCGGTCTCCCTATAGTGAGTCGTATTAATTTCGATAAGCCAGGTTAACCTGCATTA
ATGAATCGGCCAACGCGCGGGGAGAGGCGGTTTGCGTATTGGGCGCTCTTCCGCTTCCTCGCTCACTGACTCGCTGCGCTCGGTCGTTCGGCTGCGGCGAGCGGTATCA
GCTCACTCAAAGGCGGTAATACGGTTATCCACAGAATCAGGGGATAACGCAGGAAAGAACATGTGAGCAAAAGGCCAGCAAAAGGCCAGGAACCGTAAAAAGGCCGCGTT
GCTGGCGTTTTTCCATAGGCTCCGCCCCCCTGACGAGCATCACAAAAATCGACGCTCAAGTCAGAGGTGGCGAAACCCGACAGGACTATAAAGATACCAGGCGTTTCCCCC
TGGAAGCTCCCTCGTGCGCTCTCCTGTTCCGACCCTGCCGCTTACCGGATACCTGTCCGCCTTTCTCCCTTCGGGAAGCGTGGCGCTTTCTCATAGCTCACGCTGTAGGTA
TCTCAGTTCGGTGTAGGTCGTTCGCTCCAAGCTGGGCTGTGTGCACGAACCCCCCGTTCAGCCCGACCGCTGCGCCTTATCCGGTAACTATCGTCTTGAGTCCAACCCGGT
AAGACACGACTTATCGCCACTGGCAGCAGCCACTGGTAACAGGATTAGCAGAGCGAGGTATGTAGGCGGTGCTACAGAGTTCTTGAAGTGGTGGCCTAACTACGGCTACAC
TAGAAGAACAGTATTTGGTATCTGCGCTCTGCTGAAGCCAGTTACCTTCGGAAAAAGAGTTGGTAGCTCTTGATCCGGCAAACAAACCACCGCTGGTAGCGGTGGTTTTTTT
GTTTGCAAGCAGCAGATTACGCGCAGAAAAAAAGGATCTCAA

*TPK1* promoter; GFP+GGGGlinker; *TPK2N+TPK1C* coding sequence; *HIS1* marker (replaced with a *SAT1* marker in pLC1682); *TPK1* 3’ HR

>pLC1669_pFA-pTpk1-GFP-2FL-HIS

GAAGATCCTTTGATCTTTTCTACGGGGTCTGACGCTCAGTGGAACGAAAACTCACGTTAAGGGATTTTGGTCATGAGATTATCAAAAAGGATCTTCACCTAGATCCTTTTAAAT
TAAAAATGAAGTTTTAAATCAATCTAAAGTATATATGAGTAAACTTGGTCTGACAGTTACCAATGCTTAATCAGTGAGGCACCTATCTCAGCGATCTGTCTATTTCGTTCATCCA
TAGTTGCCTGACTCCCCGTCGTGTAGATAACTACGATACGGGAGGGCTTACCATCTGGCCCCAGTGCTGCAATGATACCGCGAGACCCACGCTCACCGGCTCCAGATTTAT
CAGCAATAAACCAGCCAGCCGGAAGGGCCGAGCGCAGAAGTGGTCCTGCAACTTTATCCGCCTCCATCCAGTCTATTAATTGTTGCCGGGAAGCTAGAGTAAGTAGTTCGC
CAGTTAATAGTTTGCGCAACGTTGTTGCCATTGCTACAGGCATCGTGGTGTCACGCTCGTCGTTTGGTATGGCTTCATTCAGCTCCGGTTCCCAACGATCAAGGCGAGTTAC
ATGATCCCCCATGTTGTGCAAAAAAGCGGTTAGCTCCTTCGGTCCTCCGATCGTTGTCAGAAGTAAGTTGGCCGCAGTGTTATCACTCATGGTTATGGCAGCACTGCATAATT
CTCTTACTGTCATGCCATCCGTAAGATGCTTTTCTGTGACTGGTGAGTACTCAACCAAGTCATTCTGAGAATAGTGTATGCGGCGACCGAGTTGCTCTTGCCCGGCGTCAATA
CGGGATAATACCGCGCCACATAGCAGAACTTTAAAAGTGCTCATCATTGGAAAACGTTCTTCGGGGCGAAAACTCTCAAGGATCTTACCGCTGTTGAGATCCAGTTCGATGT
AACCCACTCGTGCACCCAACTGATCTTCAGCATCTTTTACTTTCACCAGCGTTTCTGGGTGAGCAAAAACAGGAAGGCAAAATGCCGCAAAAAAGGGAATAAGGGCGACACG
GAAATGTTGAATACTCATACTCTTCCTTTTTCAATATTATTGAAGCATTTATCAGGGTTATTGTCTCATGAGCGGATACATATTTGAATGTATTTAGAAAAATAAACAAATAGGG
GTTCCGCGCACATTTCCCCGAAAAGTGCCACCTGACGTCTAAGAAACCATTATTATCATGACATTAACCTATAAAAATAGGCGTATCACGAGGCCCTTTCGTCTCGCGCGTTT
CGGTGATGACGGTGAAAACCTCTGACACATGCAGCTCCCGGAGACGGTCACAGCTTGTCTGTAAGCGGATGCCGGGAGCAGACAAGCCCGTCAGGGCGCGTCAGCGGGT
GTTGGCGGGTGTCGGGGCTGGCTTAACTATGCGGCATCAGAGCAGATTGTACTGAGAGTGCACCATATGGACATATTGTCGTTAGAACGCGGCTACAATTAATACATAACCT
TATGTATCATACACATACGATTTAGGTGACACTATAGAACGCGGCCGCCAGCTGAAGCTTCGTACGCTGCAGGTCGACGGATCCAGACAGAAGATAATATTTTTTCTTAACTG
TCACGCAAAAGTTTTAACTAACTAACTAACTAACTAATTAGACCAATTCCCCTAAAGGACTTAAGGACTAAAATAAGCTTATTTCAATTAATATTATCATTGAATAATAGATACTT
ATAGCAGTTATAGTAGAATTTAATTTCTTTTTTTTTTCTTTCTTTCTTTTGTCACACATTTATCCGTTTTGACAGACTAAATTAAACAAAAAATTTTTGAACATCAGGGAACGACCA
TATACAAATAAAAGCAGAAGAAAAAAACGGAAGAAGAAGAAGAAGAAAAAAAAGTCAACCAAAAAATTGAACAAGAGGTCTAAAGAACCACAACATTATACAAAAGAAAAGAA
ATTAAATTAAATTACATTAAAAGAGAATTGAAACATAACACTTGAAGAAGAAAATAAAAGTATTTTTATATTATATATTCCGACTAGTATTATCTGAGATCAATATATCCTATCGTT
ATCCTCCTCCTTCTCCTTTCAACTTTTGAAAAAGGTGATATTATTCAACACTGTTTTTGTTTTATCAACCAAAACCAGGATGTCTAAAGGTGAAGAATTATTCACTGGTGTTGTC
CCAATTTTGGTTGAATTAGATGGTGATGTTAATGGTCACAAATTTTCTGTCTCCGGTGAAGGTGAAGGTGATGCTACTTACGGTAAATTGACCTTAAAATTTATTTGTACTACTG
GTAAATTGCCAGTTCCATGGCCAACCTTAGTCACTACTTTCGGTTATGGTGTTCAATGTTTTGCGAGATACCCAGATCATATGAAACAACATGACTTTTTCAAGTCTGCCATGC
CAGAAGGTTATGTTCAAGAAAGAACTATTTTTTTCAAAGATGACGGTAACTACAAGACCAGAGCTGAAGTCAAGTTTGAAGGTGATACCTTAGTTAATAGAATCGAATTAAAAG
GTATTGATTTTAAAGAAGATGGTAACATTTTAGGTCACAAATTGGAATACAACTATAACTCTCACAATGTTTACATCATGGCTGACAAACAAAAGAATGGTATCAAAGTTAACTT
CAAAATTAGACACAACATTGAAGATGGTTCTGTTCAATTAGCTGACCATTATCAACAAAATACTCCAATTGGTGATGGTCCAGTCTTGTTACCAGACAACCATTACTTATCCAC
TCAATCTGCCTTATCCAAAGATCCAAACGAAAAGAGAGACCACATGGTCTTGTTAGAATTTGTTACTGCTGCTGGTATTACCCATGGTATGGATGAATTGTACAAAGGTGGTG
GTGGTGACAATCATCAACAACAACAGCAGCTTCAGCATCAGCAATATCAGCAACAATTTCAACAACCGCAGCAACAACTTTATCCAGGCGAACAAATAGTTCACCCTGCTGCA
GCCCAAACTGGACAAAATACTACAAATGTAACAGCAGTGTCGAGCTCCAACATTACCCAATCTGCTACCTCATCACTCCATTCACAGCAATTGCAGCATGTAGATGTTTCCAA
ATCGGCTGCTGAAGAAGCCATCAGAAGATCGTTGTTGCCTGAACGTTCTACTGTTTCAAAGGGGAAATACTCTTTGACTGATTTCTCAATTATGAGAACTTTGGGAACAGGTT
CCTTTGGTAGAGTACATTTAGTGAGATCAGTTCACAATGGTAGATATTATGCTATCAAAGTTTTGAAGAAGCATCAAGTTGTGAAAATGAAGCAAGTTGAACACACAAATGATG
AAAGAAGAATGTTGAAGTTGGTTGAACATCCATTTTTGATTAGAATGTGGGGGACTTTCCAAGATTCCAAAAATTTATTTATGGTTATGGACTATATTGAAGGGGGAGAGTTAT
TTTCATTGTTAAGAAAGTCTCAGAGATTTCCCAATCCAGTAGCCAAGTTTTATGCTGCAGAGGTTACCTTGGCTTTGGAGTATTTGCACAGTCATGATATTATCTATCGTGATTT
GAAACCAGAAAATATATTATTGGATAGAAATGGTCATATAAAGATTACAGATTTTGGATTTGCCAAAGAAGTTAGTACCGTTACATGGACGTTATGTGGTACCCCCGATTATAT
TGCCCCAGAAGTCATAACAACCAAACCCTATAACAAGTCGGTGGACTGGTGGTCGTTGGGGGTATTGATTTTTGAAATGTTGGCAGGTTACACTCCATTTTATGATTCGACTC
CAATGAAAACTTATGAAAAAATTTTGGCAGGCAAAATACATTACCCAAGTTTTTTTCAACCTGATGTGATTGATTTGTTGACTAAATTAATAACTGCTGATTTGACAAGAAGATT
GGGTAATTTGATCAACGGACCAGCAGATATCAGAAATCATCCCTGGTTTCTGGAAGTGGTATGGGAGAAATTGTTGGCAAAGGATATTGAAACTCCTTATGAACCACCAATCA
CAGCTGGGGTTGGTGATTCGTCATTGTTTGACCATTATCCAGAAGAACAATTAGACTACGGAAGCCAAGGAGAAGATCCTTATGCTCTGTATTTCCTTGACTTTTGAATTGAG
AGGAGATGAACTTTCATAAATGAATAAATGAGGCGCGCCATTATAAGTAAATGCATGTATACTAAACTCACAAATTAGAGCTTCAATTTAATTATATCAGTTATTACCCGGGAAT
CTCGGTCGTAATGATTTCTATAATGACGAAAAAAAAAAAATTGGAAAGAAAAAGCTTCATGGCCTTTATAAAAAGGAACTATCCAATACCTCGCCAGAACCAAGTAACAGTATTT
TACGGGGCACAAATCAAGAACAATAAGACAGGACTGTAAAGATGGACGCATAGATCCTGGAGGATGAGGAGACAGAAGTTAGTAGTAACAATTTGGGGACAGAAGAGGATA
CAGTGACATCAGAGGAAGAAGACAGCGAAGGTATAGTCAAAAAGCAATAGGACAGCAAGACAATTTATTTATTTAATAGTTTTTTATAAATGTAAAATCTATCACAACATGTGT
ACTTAATTTTCTGTGGTAGGGGAGGCCATTTCGTTGAAACGTGTCGCGAGCGGTTGTGAGAATTGACTCATTTTGATTATTACGCAACCATTGAAATTTTTTATTTTTTTTTGGT
GAAGATTTTTCCCACACAACTTCTTCTTTTACTTAACCACCAACTACCGATAATGGATTTAGTCAATCATTTACCAGACCGTTTGTTATTTGCTGTTCCTAAAAAGGGCAGATTA
TACGAAAAATGCTGTAACTTATTGAGTGGTGCCGATATACAGTTTAGAAGATCTAATAGATTAGATATAGCACTTTCTACAAACTTGCCAATTGCATTAATCTTCTTGCCTGCAG
CTGATATCCCAGTTTTCGTTGGAGAAGGTAATTGTGACTTGGGTATAACTGGGTTAGACCAAATCAAAGAAGCTGAACAATTCGACAACATCGAGGACTTGTTGGATTTGAAA
TTTGGTTCATGCAAATTGCAGATCCAAGTTCCAGCAGATGGCGAGTACGAAAAGCCAGAACAGCTTGTTGGAAAGAAAATTGTGTCTTCATTTACAAAATTGAGTACCGACTA
TTTCAAACAATTGTCAGACAAACCTACTAATATCAGATATGTCGGTGGTTCCGTTGAGGCTTCTTGTGCCTTGGGTGTTGCTGATGCTATTGTCGATTTGGTTGAAAGTGGTG
AAACTATGAAAGCAGCTGGGTTAAAGGCCATAGAAACCATATTGGAGACTTCGGCTCATTTGATTTCGTCAAAAAAAAGTAAATTCCCAGAAATGGTCAATATAATCGTCCAAA
GACTTCAAGGTGTTTTAGCTGCCCAGGAATATGTCTTGTGTAACTACAATGCCCCAAAATCCATCCAAGCAAAATGTTTAACCATTACTCCAGGCAGAAGGGCCGCTACTGTC
TCTACTTTGGACAAACACAGCGACGACGAAGAGGACTGGGTTGCCATCTCATCCATGGTTAATAGAAAGGAAATCGGTAATGTAATGGACGAATTGAAGAAAGCTGGTGCAA
CCGATATATTGGTACTCGAGATATCAAATTGTAGAGTTTAAAAGAAGTGATAGTTTCTCATAAATATTATACATATATACTTATAGATCTACTTCAATTTGACTCAATCTGTCTTA
TACCATCTTTATCTACCACCTTGATGTACACACCTTTAAAGTCAATAGGCATTCTCGTTTAAACGAGCTCCACAATAGTTCAATATTGGAATCAAACATAATAGCGAGAATTGGT
TTTTATCTGAGTACCACATTGAGAATGGTATTGGATGTTATTAAGTTGCCATTTTTGAATGAACTTCTCACTGATGATTCTTTTTATTAAATCCAGGGTCCTTTTTTTAGGGTATT
CTACTAAAACTTGATATTTGGGATGATTTGGTAGTTCTATTTTGATTTCCACTAATGAAAAGCATTTCTAACAAGTTTTGGAAATTTGACTGAGATGTGCATTACTAAGAGCTTC
GTTGTTACGGTGATACTAAAGTTTGTCAAGTCTCTTAAACATTACATTGAATACTATTTGATAGTTGGTGATAAAAAAAACTCCGTAAACAATAATTTCGTGTTTAATTCACATTT
TATTGGAGCATGAAAATTATTTAACCGAACGCGTTCTTGAATGTGTATAAAAATAGAAATCCCATATTATCGAATTAGAGGTCTCCGCGGATCTGCCGGTCTCCCTATAGTGAG
TCGTATTAATTTCGATAAGCCAGGTTAACCTGCATTAATGAATCGGCCAACGCGCGGGGAGAGGCGGTTTGCGTATTGGGCGCTCTTCCGCTTCCTCGCTCACTGACTCGCT
GCGCTCGGTCGTTCGGCTGCGGCGAGCGGTATCAGCTCACTCAAAGGCGGTAATACGGTTATCCACAGAATCAGGGGATAACGCAGGAAAGAACATGTGAGCAAAAGGCC
AGCAAAAGGCCAGGAACCGTAAAAAGGCCGCGTTGCTGGCGTTTTTCCATAGGCTCCGCCCCCCTGACGAGCATCACAAAAATCGACGCTCAAGTCAGAGGTGGCGAAAC
CCGACAGGACTATAAAGATACCAGGCGTTTCCCCCTGGAAGCTCCCTCGTGCGCTCTCCTGTTCCGACCCTGCCGCTTACCGGATACCTGTCCGCCTTTCTCCCTTCGGGA
AGCGTGGCGCTTTCTCATAGCTCACGCTGTAGGTATCTCAGTTCGGTGTAGGTCGTTCGCTCCAAGCTGGGCTGTGTGCACGAACCCCCCGTTCAGCCCGACCGCTGCGC
CTTATCCGGTAACTATCGTCTTGAGTCCAACCCGGTAAGACACGACTTATCGCCACTGGCAGCAGCCACTGGTAACAGGATTAGCAGAGCGAGGTATGTAGGCGGTGCTAC
AGAGTTCTTGAAGTGGTGGCCTAACTACGGCTACACTAGAAGAACAGTATTTGGTATCTGCGCTCTGCTGAAGCCAGTTACCTTCGGAAAAAGAGTTGGTAGCTCTTGATCC
GGCAAACAAACCACCGCTGGTAGCGGTGGTTTTTTTGTTTGCAAGCAGCAGATTACGCGCAGAAAAAAAGGATCTCAA

*TPK1* promoter; GFP+GGGGlinker; *TPK2* ORF; *HIS1* marker (replaced with a *SAT1* marker in pLC1683); *TPK1* 3' HR

>pLC1676_pFA-pTpk1-GFP-1N2C-HIS

GAAGATCCTTTGATCTTTTCTACGGGGTCTGACGCTCAGTGGAACGAAAACTCACGTTAAGGGATTTTGGTCATGAGATTATCAAAAAGGATCTTCACCTAGATCCTTTTAAAT
TAAAAATGAAGTTTTAAATCAATCTAAAGTATATATGAGTAAACTTGGTCTGACAGTTACCAATGCTTAATCAGTGAGGCACCTATCTCAGCGATCTGTCTATTTCGTTCATCCA
TAGTTGCCTGACTCCCCGTCGTGTAGATAACTACGATACGGGAGGGCTTACCATCTGGCCCCAGTGCTGCAATGATACCGCGAGACCCACGCTCACCGGCTCCAGATTTAT
CAGCAATAAACCAGCCAGCCGGAAGGGCCGAGCGCAGAAGTGGTCCTGCAACTTTATCCGCCTCCATCCAGTCTATTAATTGTTGCCGGGAAGCTAGAGTAAGTAGTTCGC
CAGTTAATAGTTTGCGCAACGTTGTTGCCATTGCTACAGGCATCGTGGTGTCACGCTCGTCGTTTGGTATGGCTTCATTCAGCTCCGGTTCCCAACGATCAAGGCGAGTTAC
ATGATCCCCCATGTTGTGCAAAAAAGCGGTTAGCTCCTTCGGTCCTCCGATCGTTGTCAGAAGTAAGTTGGCCGCAGTGTTATCACTCATGGTTATGGCAGCACTGCATAATT
CTCTTACTGTCATGCCATCCGTAAGATGCTTTTCTGTGACTGGTGAGTACTCAACCAAGTCATTCTGAGAATAGTGTATGCGGCGACCGAGTTGCTCTTGCCCGGCGTCAATA
CGGGATAATACCGCGCCACATAGCAGAACTTTAAAAGTGCTCATCATTGGAAAACGTTCTTCGGGGCGAAAACTCTCAAGGATCTTACCGCTGTTGAGATCCAGTTCGATGT
AACCCACTCGTGCACCCAACTGATCTTCAGCATCTTTTACTTTCACCAGCGTTTCTGGGTGAGCAAAAACAGGAAGGCAAAATGCCGCAAAAAAGGGAATAAGGGCGACACG
GAAATGTTGAATACTCATACTCTTCCTTTTTCAATATTATTGAAGCATTTATCAGGGTTATTGTCTCATGAGCGGATACATATTTGAATGTATTTAGAAAAATAAACAAATAGGG
GTTCCGCGCACATTTCCCCGAAAAGTGCCACCTGACGTCTAAGAAACCATTATTATCATGACATTAACCTATAAAAATAGGCGTATCACGAGGCCCTTTCGTCTCGCGCGTTT
CGGTGATGACGGTGAAAACCTCTGACACATGCAGCTCCCGGAGACGGTCACAGCTTGTCTGTAAGCGGATGCCGGGAGCAGACAAGCCCGTCAGGGCGCGTCAGCGGGT
GTTGGCGGGTGTCGGGGCTGGCTTAACTATGCGGCATCAGAGCAGATTGTACTGAGAGTGCACCATATGGACATATTGTCGTTAGAACGCGGCTACAATTAATACATAACCT
TATGTATCATACACATACGATTTAGGTGACACTATAGAACGCGGCCGCCAGCTGAAGCTTCGTACGCTGCAGGTCGACGGATCCAGACAGAAGATAATATTTTTTCTTAACTG
TCACGCAAAAGTTTTAACTAACTAACTAACTAACTAATTAGACCAATTCCCCTAAAGGACTTAAGGACTAAAATAAGCTTATTTCAATTAATATTATCATTGAATAATAGATACTT
ATAGCAGTTATAGTAGAATTTAATTTCTTTTTTTTTTTCTTTCTTTCTTTTGTCACACATTTATCCGTTTTGACAGACTAAATTAAACAAAAAATTTTTGAACATCAGGGAACGACC
ATATACAAATAAAAGCAGAAGAAAAAAACGGAAGAAGAAGAAGAAGAAAAAAAAAGTCAACCAAAAAATTGAACAAGAGGTCTAAAGAACCACAACATTATACAAAAGAAAAGA
AATTAAATTAAATTACATTAAAAGAGAATTGAAACATAACACTTGAAGAAGAAAATAAAAGTATTTTTATATTATATATTCCGACTAGTATTATCTGAGATCAATATATCCTATCGT
TATCCTCCTCCTTCTCCTTTCAACTTTTGAAAAAGGTGATATTATTCAACACTGTTTTTGTTTTATCAACCAAAACCAGGATGTCTAAAGGTGAAGAATTATTCACTGGTGTTGT
CCCAATTTTGGTTGAATTAGATGGTGATGTTAATGGTCACAAATTTTCTGTCTCCGGTGAAGGTGAAGGTGATGCTACTTACGGTAAATTGACCTTAAAATTTATTTGTACTACT
GGTAAATTGCCAGTTCCATGGCCAACCTTAGTCACTACTTTCGGTTATGGTGTTCAATGTTTTGCGAGATACCCAGATCATATGAAACAACATGACTTTTTCAAGTCTGCCATG
CCAGAAGGTTATGTTCAAGAAAGAACTATTTTTTTCAAAGATGACGGTAACTACAAGACCAGAGCTGAAGTCAAGTTTGAAGGTGATACCTTAGTTAATAGAATCGAATTAAAA
GGTATTGATTTTAAAGAAGATGGTAACATTTTAGGTCACAAATTGGAATACAACTATAACTCTCACAATGTTTACATCATGGCTGACAAACAAAAGAATGGTATCAAAGTTAACT
TCAAAATTAGACACAACATTGAAGATGGTTCTGTTCAATTAGCTGACCATTATCAACAAAATACTCCAATTGGTGATGGTCCAGTCTTGTTACCAGACAACCATTACTTATCCA
CTCAATCTGCCTTATCCAAAGATCCAAACGAAAAGAGAGACCACATGGTCTTGTTAGAATTTGTTACTGCTGCTGGTATTACCCATGGTATGGATGAATTGTACAAAGGTGGT
GGTGGTACATCCATGGAACCAGCAGACACAAGCATCAGGTCATTAAACGACATCAACTTACAAGAACTTGCCAACAAACAACATCAACGAAATATATATGCTCAAGGATCACC
TACACTAGAAGATTCAATAACATCAAATGGTAATGATATTAATAATCCCAACAATAATATAAATAAAACCAATAATAACAGTAATTCAAGTCTTTCCTTGACCAAGAAAACATCAA
GACATATCAATAAAGACACTACAACTAAGGGGAAATACTCTTTGACTGATTTCTCAATTATGAGAACTTTGGGAACAGGTTCCTTTGGTAGAGTACATTTAGTGAGATCAGTTC
ACAATGGTAGATATTATGCTATCAAAGTTTTGAAGAAGCATCAAGTTGTGAAAATGAAGCAAGTTGAACACACAAATGATGAAAGAAGAATGTTGAAGTTGGTTGAACATCCAT
TTTTGATTAGAATGTGGGGGACTTTCCAAGATTCCAAAAATTTATTTATGGTTATGGACTATATTGAAGGGGGAGAGTTATTTTCATTGTTAAGAAAGTCTCAGAGATTTCCCAA
TCCAGTAGCCAAGTTTTATGCTGCAGAGGTTACCTTGGCTTTGGAGTATTTGCACAGTCATGATATTATCTATCGTGATTTGAAACCAGAAAATATATTATTGGATAGAAATGG
TCATATAAAGATTACAGATTTTGGATTTGCCAAAGAAGTTAGTACCGTTACATGGACGTTATGTGGTACCCCCGATTATATTGCCCCAGAAGTCATAACAACCAAACCCTATAA
CAAGTCGGTGGACTGGTGGTCGTTGGGGGTATTGATTTTGAAATGTTGGCAGGTTACACTCCATTTTATGATTCGACTCCAATGAAAACTTATGAAAAAATTTTGGCAGGCA
AAATACATTACCCAAGTTTTTTTCAACCTGATGTGATTGATTTGTTGACTAAATTAATAACTGCTGATTTGACAAGAAGATTGGGTAATTTGATCAACGGACCAGCAGATATCAG
AAATCATCCCTGGTTTCTGGAAGTGGTATGGGAGAAATTGTTGGCAAAGGATATTGAAACTCCTTATGAACCACCAATCACAGCTGGGGTTGGTGATTCGTCATTGTTTGACC
ATTATCCAGAAGAACAATTAGACTACGGAAGCCAAGGAGAAGATCCTTATGCTCTGTATTTCCTTGACTTTTGAATTGAGAGGAGATGAACTTTCATAAATGAATAAATGAGGC
GCGCCATTATAAGTAAATGCATGTATACTAAACTCACAAATTAGAGCTTCAATTTAATTATATCAGTTATTACCCGGGAATCTCGGTCGTAATGATTTCTATAATGACGAAAAAA
AAAAAATTGGAAAGAAAAAGCTTCATGGCCTTTATAAAAAGGAACTATCCAATACCTCGCCAGAACCAAGTAACAGTATTTTACGGGGCACAAATCAAGAACAATAAGACAGG
ACTGTAAAGATGGACGCATAGATCCTGGAGGATGAGGAGACAGAAGTTAGTAGTAACAATTTGGGGACAGAAGAGGATACAGTGACATCAGAGGAAGAAGACAGCGAAGGT
ATAGTCAAAAAGCAATAGGACAGCAAGACAATTTATTTATTTAATAGTTTTTTATAAATGTAAAATCTATCACAACATGTGTACTTAATTTTCTGTGGTAGGGGAGGCCATTTCG
TTGAAACGTGTCGCGAGCGGTTGTGAGAATTGACTCATTTTGATTATTACGCAACCATTGAAATTTTTTATTTTTTTTTGGTGAAGATTTTTCCCACACAACTTCTTCTTTTACTT
AACCACCAACTACCGATAATGGATTTAGTCAATCATTTACCAGACCGTTTGTTATTTGCTGTTCCTAAAAAGGGCAGATTATACGAAAAATGCTGTAACTTATTGAGTGGTGCC
GATATACAGTTTAGAAGATCTAATAGATTAGATATAGCACTTTCTACAAACTTGCCAATTGCATTAATCTTCTTGCCTGCAGCTGATATCCCAGTTTTCGTTGGAGAAGGTAATT
GTGACTTGGGTATAACTGGGTTAGACCAAATCAAAGAAGCTGAACAATTCGACAACATCGAGGACTTGTTGGATTTGAAATTTGGTTCATGCAAATTGCAGATCCAAGTTCCA
GCAGATGGCGAGTACGAAAAGCCAGAACAGCTTGTTGGAAAGAAAATTGTGTCTTCATTTACAAAATTGAGTACCGACTATTTCAAACAATTGTCAGACAAACCTACTAATATC
AGATATGTCGGTGGTTCCGTTGAGGCTTCTTGTGCCTTGGGTGTTGCTGATGCTATTGTCGATTTGGTTGAAAGTGGTGAAACTATGAAAGCAGCTGGGTTAAAGGCCATAG
AAACCATATTGGAGACTTCGGCTCATTTGATTTCGTCAAAAAAAAGTAAATTCCCAGAAATGGTCAATATAATCGTCCAAAGACTTCAAGGTGTTTTAGCTGCCCAGGAATATG
TCTTGTGTAACTACAATGCCCCAAAATCCATCCAAGCAAAATGTTTAACCATTACTCCAGGCAGAAGGGCCGCTACTGTCTCTACTTTGGACAAACACAGCGACGACGAAGA
GGACTGGGTTGCCATCTCATCCATGGTTAATAGAAAGGAAATCGGTAATGTAATGGACGAATTGAAGAAAGCTGGTGCAACCGATATATTGGTACTCGAGATATCAAATTGTA
GAGTTTAAAAGAAGTGATAGTTTCTCATAAATATTATACATATATACTTATAGATCTACTTCAATTTGACTCAATCTGTCTTATACCATCTTTATCTACCACCTTGATGTACACAC
CTTTAAAGTCAATAGGCATTCTCGTTTAAACGAGCTCCACAATAGTTCAATATTGGAATCAAACATAATAGCGAGAATTGGTTTTTATCTGAGTACCACATTGAGAATGGTATTG
GATGTTATTAAGTTGCCATTTTTGAATGAACTTCTCACTGATGATTCTTTTTATTAAATCCAGGGTCCTTTTTTTAGGGTATTCTACTAAAACTTGATATTTGGGATGATTTGGTA
GTTCTATTTTGATTTCCACTAATGAAAAGCATTTCTAACAAGTTTTGGAAATTTGACTGAGATGTGCATTACTAAGAGCTTCGTTGTTACGGTGATACTAAAGTTTGTCAAGTCT
CTTAAACATTACATTGAATACTATTTGATAGTTGGTGATAAAAAAAACTCCGTAAACAATAATTTCGTGTTTAATTCACATTTTATTGGAGCATGAAAATTATTTAACCGAACGCG
TTCTTGAATGTGTATAAAAATAGAAATCCCATATTATCGAATTAGAGGTCTCCGCGGATCTGCCGGTCTCCCTATAGTGAGTCGTATTAATTTCGATAAGCCAGGTTAACCTGC
ATTAATGAATCGGCCAACGCGCGGGGAGAGGCGGTTTGCGTATTGGGCGCTCTTCCGCTTCCTCGCTCACTGACTCGCTGCGCTCGGTCGTTCGGCTGCGGCGAGCGGTA
TCAGCTCACTCAAAGGCGGTAATACGGTTATCCACAGAATCAGGGGATAACGCAGGAAAGAACATGTGAGCAAAAGGCCAGCAAAAGGCCAGGAACCGTAAAAAGGCCGC
GTTGCTGGCGTTTTTCCATAGGCTCCGCCCCCCTGACGAGCATCACAAAAATCGACGCTCAAGTCAGAGGTGGCGAAACCCGACAGGACTATAAAGATACCAGGCGTTTCC
CCCTGGAAGCTCCCTCGTGCGCTCTCCTGTTCCGACCCTGCCGCTTACCGGATACCTGTCCGCCTTTCTCCCTTCGGGAAGCGTGGCGCTTTCTCATAGCTCACGCTGTAG
GTATCTCAGTTCGGTGTAGGTCGTTCGCTCCAAGCTGGGCTGTGTGCACGAACCCCCCGTTCAGCCCGACCGCTGCGCCTTATCCGGTAACTATCGTCTTGAGTCCAACCC
GGTAAGACACGACTTATCGCCACTGGCAGCAGCCACTGGTAACAGGATTAGCAGAGCGAGGTATGTAGGCGGTGCTACAGAGTTCTTGAAGTGGTGGCCTAACTACGGCTA
CACTAGAAGAACAGTATTTGGTATCTGCGCTCTGCTGAAGCCAGTTACCTTCGGAAAAAGAGTTGGTAGCTCTTGATCCGGCAAACAAACCACCGCTGGTAGCGGTGGTTTT
TTTGTTTGCAAGCAGCAGATTACGCGCAGAAAAAAAGGATCTCAA

*TPK1* promoter; GFP+GGGGlinker; *TPK1N+TPK2C* coding sequence; *HIS1* marker (replaced with a *SAT1* marker in pLC1685); *TPK1* 3’ HR

>pLC1673_pFA-pTpk2-GFP-2N-HIS

GAAGATCCTTTGATCTTTTCTACGGGGTCTGACGCTCAGTGGAACGAAAACTCACGTTAAGGGATTTTGGTCATGAGATTATCAAAAAGGATCTTCACCTAGATCCTTTTAAAT
TAAAAATGAAGTTTTAAATCAATCTAAAGTATATATGAGTAAACTTGGTCTGACAGTTACCAATGCTTAATCAGTGAGGCACCTATCTCAGCGATCTGTCTATTTCGTTCATCCA
TAGTTGCCTGACTCCCCGTCGTGTAGATAACTACGATACGGGAGGGCTTACCATCTGGCCCCAGTGCTGCAATGATACCGCGAGACCCACGCTCACCGGCTCCAGATTTAT
CAGCAATAAACCAGCCAGCCGGAAGGGCCGAGCGCAGAAGTGGTCCTGCAACTTTATCCGCCTCCATCCAGTCTATTAATTGTTGCCGGGAAGCTAGAGTAAGTAGTTCGC
CAGTTAATAGTTTGCGCAACGTTGTTGCCATTGCTACAGGCATCGTGGTGTCACGCTCGTCGTTTGGTATGGCTTCATTCAGCTCCGGTTCCCAACGATCAAGGCGAGTTAC
ATGATCCCCCATGTTGTGCAAAAAAGCGGTTAGCTCCTTCGGTCCTCCGATCGTTGTCAGAAGTAAGTTGGCCGCAGTGTTATCACTCATGGTTATGGCAGCACTGCATAATT
CTCTTACTGTCATGCCATCCGTAAGATGCTTTTCTGTGACTGGTGAGTACTCAACCAAGTCATTCTGAGAATAGTGTATGCGGCGACCGAGTTGCTCTTGCCCGGCGTCAATA
CGGGATAATACCGCGCCACATAGCAGAACTTTAAAAGTGCTCATCATTGGAAAACGTTCTTCGGGGCGAAAACTCTCAAGGATCTTACCGCTGTTGAGATCCAGTTCGATGT
AACCCACTCGTGCACCCAACTGATCTTCAGCATCTTTTACTTTCACCAGCGTTTCTGGGTGAGCAAAAACAGGAAGGCAAAATGCCGCAAAAAAGGGAATAAGGGCGACACG
GAAATGTTGAATACTCATACTCTTCCTTTTTCAATATTATTGAAGCATTTATCAGGGTTATTGTCTCATGAGCGGATACATATTTGAATGTATTTAGAAAAATAAACAAATAGGG
GTTCCGCGCACATTTCCCCGAAAAGTGCCACCTGACGTCTAAGAAACCATTATTATCATGACATTAACCTATAAAAATAGGCGTATCACGAGGCCCTTTCGTCTCGCGCGTTT
CGGTGATGACGGTGAAAACCTCTGACACATGCAGCTCCCGGAGACGGTCACAGCTTGTCTGTAAGCGGATGCCGGGAGCAGACAAGCCCGTCAGGGCGCGTCAGCGGGT
GTTGGCGGGTGTCGGGGCTGGCTTAACTATGCGGCATCAGAGCAGATTGTACTGAGAGTGCACCATATGGACATATTGTCGTTAGAACGCGGCTACAATTAATACATAACCT
TATGTATCATACACATACGATTTAGGTGACACTATAGAACGCGGCCGCCAGCTGAAGCTTCGTACGCTGCAGGTCGACGGATCCACATTGTTAAAAGTCGATACTTCAGTTG
CTTTTTTTTATTATTTTCTATCCAGCAAAAAACACATTGTCATTGTTTGTGTATCATTTTTTGTCCAGATAACAAACACCAAATTGAGGTCAACTAAACAACAACTTGTATAGTTCT
ACCACACTATTTTGAAGAGTCAGCTACAGTTCCTAGTTTCTCAACATAATATATCAACCTATCAATACTCGGATTGCAGGTCAATTTAGTTTACAACCACCAATTGCCAAAGTAT
TGGATTCTACTTTTTCCGACTTGTAAGTGAACACAAAGAAGATTAGACAAACAATCACCCACTCACACCTACTACTCACCCAATTTCCATTCCACCTTTTTTTTTCGAAAAAAAT
AACCTTCCCCCTTTTTGATGGTGAATCTTTTAAAGAAACTTCACATCACCAAGCTGCATCAACTGAATCAATCCAATTCGGACAGTAATTCCTTAAACTCAAACACATCAATGTC
TAAAGGTGAAGAATTATTCACTGGTGTTGTCCCAATTTTGGTTGAATTAGATGGTGATGTTAATGGTCACAAATTTTCTGTCTCCGGTGAAGGTGAAGGTGATGCTACTTACGG
TAAATTGACCTTAAAATTTATTTGTACTACTGGTAAATTGCCAGTTCCATGGCCAACCTTAGTCACTACTTTCGGTTATGGTGTTCAATGTTTTGCGAGATACCCAGATCATATG
AAACAACATGACTTTTTCAAGTCTGCCATGCCAGAAGGTTATGTTCAAGAAAGAACTATTTTTTTCAAAGATGACGGTAACTACAAGACCAGAGCTGAAGTCAAGTTTGAAGGT
GATACCTTAGTTAATAGAATCGAATTAAAAGGTATTGATTTTAAAGAAGATGGTAACATTTTAGGTCACAAATTGGAATACAACTATAACTCTCACAATGTTTACATCATGGCTG
ACAAACAAAAGAATGGTATCAAAGTTAACTTCAAAATTAGACACAACATTGAAGATGGTTCTGTTCAATTAGCTGACCATTATCAACAAAATACTCCAATTGGTGATGGTCCAG
TCTTGTTACCAGACAACCATTACTTATCCACTCAATCTGCCTTATCCAAAGATCCAAACGAAAAGAGAGACCACATGGTCTTGTTAGAATTTGTTACTGCTGCTGGTATTACCC
ATGGTATGGATGAATTGTACAAAGGTGGTGGTGACAATCATCAACAACAACAGCAGCTTCAGCATCAGCAATATCAGCAACAATTTCAACAACCGCAGCAACAACTTTAT
CCAGGCGAACAAATAGTTCACCCTGCTGCAGCCCAAACTGGACAAAATACTACAAATGTAACAGCAGTGTCGAGCTCCAACATTACCCAATCTGCTACCTCATCACTCCATTC
ACAGCAATTGCAGCATGTAGATGTTTCCAAATCGGCTGCTGAAGAAGCCATCAGAAGATCGTTGTTGCCTGAACGTTCTACTGTTTCATAAGGCGCGCCATTATAAGTAAATG
CATGTATACTAAACTCACAAATTAGAGCTTCAATTTAATTATATCAGTTATTACCCGGGAATCTCGGTCGTAATGATTTCTATAATGACGAAAAAAAAAAAATTGGAAAGAAAAA
GCTTCATGGCCTTTATAAAAAGGAACTATCCAATACCTCGCCAGAACCAAGTAACAGTATTTTACGGGGCACAAATCAAGAACAATAAGACAGGACTGTAAAGATGGACGCAT
AGATCCTGGAGGATGAGGAGACAGAAGTTAGTAGTAACAATTTGGGGACAGAAGAGGATACAGTGACATCAGAGGAAGAAGACAGCGAAGGTATAGTCAAAAAGCAATAGG
ACAGCAAGACAATTTATTTATTTAATAGTTTTTATAAATGTAAAATCTATCACAACATGTGTACTTAATTTTCTGTGGTAGGGGAGGCCATTTCGTTGAAACGTGTCGCGAGCG
GTTGTGAGAATTGACTCATTTTGATTATTACGCAACCATTGAAATTTTTTATTTTTTTTTGGTGAAGATTTTTCCCACACAACTTCTTCTTTTACTTAACCACCAACTACCGATAAT
GGATTTAGTCAATCATTTACCAGACCGTTTGTTATTTGCTGTTCCTAAAAAGGGCAGATTATACGAAAAATGCTGTAACTTATTGAGTGGTGCCGATATACAGTTTAGAAGATC
TAATAGATTAGATATAGCACTTTCTACAAACTTGCCAATTGCATTAATCTTCTTGCCTGCAGCTGATATCCCAGTTTTCGTTGGAGAAGGTAATTGTGACTTGGGTATAACTGG
GTTAGACCAAATCAAAGAAGCTGAACAATTCGACAACATCGAGGACTTGTTGGATTTGAAATTTGGTTCATGCAAATTGCAGATCCAAGTTCCAGCAGATGGCGAGTACGAAA
AGCCAGAACAGCTTGTTGGAAAGAAAATTGTGTCTTCATTTACAAAATTGAGTACCGACTATTTCAAACAATTGTCAGACAAACCTACTAATATCAGATATGTCGGTGGTTCCG
TTGAGGCTTCTTGTGCCTTGGGTGTTGCTGATGCTATTGTCGATTTGGTTGAAAGTGGTGAAACTATGAAAGCAGCTGGGTTAAAGGCCATAGAAACCATATTGGAGACTTCG
GCTCATTTGATTTCGTCAAAAAAAAGTAAATTCCCAGAAATGGTCAATATAATCGTCCAAAGACTTCAAGGTGTTTTAGCTGCCCAGGAATATGTCTTGTGTAACTACAATGCC
CCAAAATCCATCCAAGCAAAATGTTTAACCATTACTCCAGGCAGAAGGGCCGCTACTGTCTCTACTTTGGACAAACACAGCGACGACGAAGAGGACTGGGTTGCCATCTCAT
CCATGGTTAATAGAAAGGAAATCGGTAATGTAATGGACGAATTGAAGAAAGCTGGTGCAACCGATATATTGGTACTCGAGATATCAAATTGTAGAGTTTAAAAGAAGTGATAG
TTTCTCATAAATATTATACATATATACTTATAGATCTACTTCAATTTGACTCAATCTGTCTTATACCATCTTTATCTACCACCTTGATGTACACACCTTTAAAGTCAATAGGCATTC
TCGTTTAAACGAGCTCAACATGTTGACTCTTCCTTGTCTGCTTTGTTTTTCGGTATTTTTTTTTTATTTCTTTGCCTTTATGTATCTATATTAAATGTACAAAATATATTACATGATA
TTCCAAATATCTTGCTTTCTTAAAAGTCTTTGAAAACTCAAACTAGCTATGGCCCTGTTGTATTCAATAAAAATTACAATGCATGATAGTAAACACACTAACAATACCCTTGACAT
GAGTCAAAATCAACCGCGGATCTGCCGGTCTCCCTATAGTGAGTCGTATTAATTTCGATAAGCCAGGTTAACCTGCATTAATGAATCGGCCAACGCGCGGGGAGAGGCGGT
TTGCGTATTGGGCGCTCTTCCGCTTCCTCGCTCACTGACTCGCTGCGCTCGGTCGTTCGGCTGCGGCGAGCGGTATCAGCTCACTCAAAGGCGGTAATACGGTTATCCACA
GAATCAGGGGATAACGCAGGAAAGAACATGTGAGCAAAAGGCCAGCAAAAGGCCAGGAACCGTAAAAAGGCCGCGTTGCTGGCGTTTTTCCATAGGCTCCGCCCCCCTGA
CGAGCATCACAAAAATCGACGCTCAAGTCAGAGGTGGCGAAACCCGACAGGACTATAAAGATACCAGGCGTTTCCCCCTGGAAGCTCCCTCGTGCGCTCTCCTGTTCCGAC
CCTGCCGCTTACCGGATACCTGTCCGCCTTTCTCCCTTCGGGAAGCGTGGCGCTTTCTCATAGCTCACGCTGTAGGTATCTCAGTTCGGTGTAGGTCGTTCGCTCCAAGCT
GGGCTGTGTGCACGAACCCCCCGTTCAGCCCGACCGCTGCGCCTTATCCGGTAACTATCGTCTTGAGTCCAACCCGGTAAGACACGACTTATCGCCACTGGCAGCAGCCA
CTGGTAACAGGATTAGCAGAGCGAGGTATGTAGGCGGTGCTACAGAGTTCTTGAAGTGGTGGCCTAACTACGGCTACACTAGAAGAACAGTATTTGGTATCTGCGCTCTGC
TGAAGCCAGTTACCTTCGGAAAAAGAGTTGGTAGCTCTTGATCCGGCAAACAAACCACCGCTGGTAGCGGTGGTTTTTTTGTTTGCAAGCAGCAGATTACGCGCAGAAAAAA
AGGATCTCAA

*TPK2* promoter; GFP+GGGGlinker; *TPK2N* coding sequence; *HIS1* marker; *TPK2* 3’ HR

>pLC1671_pFA-pTpk2-GFP-1N2C-HIS

GAAGATCCTTTGATCTTTTCTACGGGGTCTGACGCTCAGTGGAACGAAAACTCACGTTAAGGGATTTTGGTCATGAGATTATCAAAAAGGATCTTCACCTAGATCCTTTTAAAT
TAAAAATGAAGTTTTAAATCAATCTAAAGTATATATGAGTAAACTTGGTCTGACAGTTACCAATGCTTAATCAGTGAGGCACCTATCTCAGCGATCTGTCTATTTCGTTCATCCA
TAGTTGCCTGACTCCCCGTCGTGTAGATAACTACGATACGGGAGGGCTTACCATCTGGCCCCAGTGCTGCAATGATACCGCGAGACCCACGCTCACCGGCTCCAGATTTAT
CAGCAATAAACCAGCCAGCCGGAAGGGCCGAGCGCAGAAGTGGTCCTGCAACTTTATCCGCCTCCATCCAGTCTATTAATTGTTGCCGGGAAGCTAGAGTAAGTAGTTCGC
CAGTTAATAGTTTGCGCAACGTTGTTGCCATTGCTACAGGCATCGTGGTGTCACGCTCGTCGTTTGGTATGGCTTCATTCAGCTCCGGTTCCCAACGATCAAGGCGAGTTAC
ATGATCCCCCATGTTGTGCAAAAAAGCGGTTAGCTCCTTCGGTCCTCCGATCGTTGTCAGAAGTAAGTTGGCCGCAGTGTTATCACTCATGGTTATGGCAGCACTGCATAATT
CTCTTACTGTCATGCCATCCGTAAGATGCTTTTCTGTGACTGGTGAGTACTCAACCAAGTCATTCTGAGAATAGTGTATGCGGCGACCGAGTTGCTCTTGCCCGGCGTCAATA
CGGGATAATACCGCGCCACATAGCAGAACTTTAAAAGTGCTCATCATTGGAAAACGTTCTTCGGGGCGAAAACTCTCAAGGATCTTACCGCTGTTGAGATCCAGTTCGATGT
AACCCACTCGTGCACCCAACTGATCTTCAGCATCTTTTACTTTCACCAGCGTTTCTGGGTGAGCAAAAACAGGAAGGCAAAATGCCGCAAAAAAGGGAATAAGGGCGACACG
GAAATGTTGAATACTCATACTCTTCCTTTTTCAATATTATTGAAGCATTTATCAGGGTTATTGTCTCATGAGCGGATACATATTTGAATGTATTTAGAAAAATAAACAAATAGGG
GTTCCGCGCACATTTCCCCGAAAAGTGCCACCTGACGTCTAAGAAACCATTATTATCATGACATTAACCTATAAAAATAGGCGTATCACGAGGCCCTTTCGTCTCGCGCGTTT
CGGTGATGACGGTGAAAACCTCTGACACATGCAGCTCCCGGAGACGGTCACAGCTTGTCTGTAAGCGGATGCCGGGAGCAGACAAGCCCGTCAGGGCGCGTCAGCGGGT
GTTGGCGGGTGTCGGGGCTGGCTTAACTATGCGGCATCAGAGCAGATTGTACTGAGAGTGCACCATATGGACATATTGTCGTTAGAACGCGGCTACAATTAATACATAACCT
TATGTATCATACACATACGATTTAGGTGACACTATAGAACGCGGCCGCCAGCTGAAGCTTCGTACGCTGCAGGTCGACGGATCCACATTGTTAAAAGTCGATACTTCAGTTG
CTTTTTTTTATTATTTTCTATCCAGCAAAAAACACATTGTCATTGTTTGTGTATCATTTTTTGTCCAGATAACAAACACCAAATTGAGGTCAACTAAACAACAACTTGTATAGTTCT
ACCACACTATTTTGAAGAGTCAGCTACAGTTCCTAGTTTCTCAACATAATATATCAACCTATCAATACTCGGATTGCAGGTCAATTTAGTTTACAACCACCAATTGCCAAAGTAT
TGGATTCTACTTTTTCCGACTTGTAAGTGAACACAAAGAAGATTAGACAAACAATCACCCACTCACACCTACTACTCACCCAATTTCCATTCCACCTTTTTTTTTCGAAAAAAAT
AACCTTCCCCCTTTTTGATGGTGAATCTTTTAAAGAAACTTCACATCACCAAGCTGCATCAACTGAATCAATCCAATTCGGACAGTAATTCCTTAAACTCAAACACATCAATGTC
TAAAGGTGAAGAATTATTCACTGGTGTTGTCCCAATTTTGGTTGAATTAGATGGTGATGTTAATGGTCACAAATTTTCTGTCTCCGGTGAAGGTGAAGGTGATGCTACTTACGG
TAAATTGACCTTAAAATTTATTTGTACTACTGGTAAATTGCCAGTTCCATGGCCAACCTTAGTCACTACTTTCGGTTATGGTGTTCAATGTTTTGCGAGATACCCAGATCATATG
AAACAACATGACTTTTTCAAGTCTGCCATGCCAGAAGGTTATGTTCAAGAAAGAACTATTTTTTTCAAAGATGACGGTAACTACAAGACCAGAGCTGAAGTCAAGTTTGAAGGT
GATACCTTAGTTAATAGAATCGAATTAAAAGGTATTGATTTTAAAGAAGATGGTAACATTTTAGGTCACAAATTGGAATACAACTATAACTCTCACAATGTTTACATCATGGCTG
ACAAACAAAAGAATGGTATCAAAGTTAACTTCAAAATTAGACACAACATTGAAGATGGTTCTGTTCAATTAGCTGACCATTATCAACAAAATACTCCAATTGGTGATGGTCCAG
TCTTGTTACCAGACAACCATTACTTATCCACTCAATCTGCCTTATCCAAAGATCCAAACGAAAAGAGAGACCACATGGTCTTGTTAGAATTTGTTACTGCTGCTGGTATTACCC
ATGGTATGGATGAATTGTACAAAGGTGGTGGTGGTACATCCATGGAACCAGCAGACACAAGCATCAGGTCATTAAACGACATCAACTTACAAGAACTTGCCAACAAACAACA
TCAACGAAATATATATGCTCAAGGATCACCTACACTAGAAGATTCAATAACATCAAATGGTAATGATATTAATAATCCCAACAATAATATAAATAAAACCAATAATAACAGTAATT
CAAGTCTTTCCTTGACCAAGAAAACATCAAGACATATCAATAAAGACACTACAACTAAGGGGAAATACTCTTTGACTGATTTCTCAATTATGAGAACTTTGGGAACAGGTTCCT
TTGGTAGAGTACATTTAGTGAGATCAGTTCACAATGGTAGATATTATGCTATCAAAGTTTTGAAGAAGCATCAAGTTGTGAAAATGAAGCAAGTTGAACACACAAATGATGAAA
GAAGAATGTTGAAGTTGGTTGAACATCCATTTTTGATTAGAATGTGGGGGACTTTCCAAGATTCCAAAAATTTATTTATGGTTATGGACTATATTGAAGGGGGAGAGTTATTTT
CATTGTTAAGAAAGTCTCAGAGATTTCCCAATCCAGTAGCCAAGTTTTATGCTGCAGAGGTTACCTTGGCTTTGGAGTATTTGCACAGTCATGATATTATCTATCGTGATTTGA
AACCAGAAAATATATTATTGGATAGAAATGGTCATATAAAGATTACAGATTTTGGATTTGCCAAAGAAGTTAGTACCGTTACATGGACGTTATGTGGTACCCCCGATTATATTG
CCCCAGAAGTCATAACAACCAAACCCTATAACAAGTCGGTGGACTGGTGGTCGTTGGGGGTATTGATTTTTGAAATGTTGGCAGGTTACACTCCATTTTATGATTCGACTCCA
ATGAAAACTTATGAAAAAATTTTGGCAGGCAAAATACATTACCCAAGTTTTTTTCAACCTGATGTGATTGATTTGTTGACTAAATTAATAACTGCTGATTTGACAAGAAGATTGG
GTAATTTGATCAACGGACCAGCAGATATCAGAAATCATCCCTGGTTTCTGGAAGTGGTATGGGAGAAATTGTTGGCAAAGGATATTGAAACTCCTTATGAACCACCAATCACA
GCTGGGGTTGGTGATTCGTCATTGTTTGACCATTATCCAGAAGAACAATTAGACTACGGAAGCCAAGGAGAAGATCCTTATGCTCTGTATTTCCTTGACTTTTGAATTGAGAG
GAGATGAACTTTCATAAATGAATAAATGAGGCGCGCCATTATAAGTAAATGCATGTATACTAAACTCACAAATTAGAGCTTCAATTTAATTATATCAGTTATTACCCGGGAATCT
CGGTCGTAATGATTTCTATAATGACGAAAAAAAAAAATTGGAAAGAAAAAGCTTCATGGCCTTTATAAAAAGGAACTATCCAATACCTCGCCAGAACCAAGTAACAGTATTTTA
CGGGGCACAAATCAAGAACAATAAGACAGGACTGTAAAGATGGACGCATAGATCCTGGAGGATGAGGAGACAGAAGTTAGTAGTAACAATTTGGGGACAGAAGAGGATACA
GTGACATCAGAGGAAGAAGACAGCGAAGGTATAGTCAAAAAGCAATAGGACAGCAAGACAATTTATTTATTTAATAGTTTTTTATAAATGTAAAATCTATCACAACATGTGTAC
TTAATTTTCTGTGGTAGGGGAGGCCATTTCGTTGAAACGTGTCGCGAGCGGTTGTGAGAATTGACTCATTTTGATTATTACGCAACCATTGAAATTTTTTATTTTTTTTTGGTGA
AGATTTTTCCCACACAACTTCTTCTTTTACTTAACCACCAACTACCGATAATGGATTTAGTCAATCATTTACCAGACCGTTTGTTATTTGCTGTTCCTAAAAAGGGCAGATTATA
CGAAAAATGCTGTAACTTATTGAGTGGTGCCGATATACAGTTTAGAAGATCTAATAGATTAGATATAGCACTTTCTACAAACTTGCCAATTGCATTAATCTTCTTGCCTGCAGCT
GATATCCCAGTTTTCGTTGGAGAAGGTAATTGTGACTTGGGTATAACTGGGTTAGACCAAATCAAAGAAGCTGAACAATTCGACAACATCGAGGACTTGTTGGATTTGAAATT
TGGTTCATGCAAATTGCAGATCCAAGTTCCAGCAGATGGCGAGTACGAAAAGCCAGAACAGCTTGTTGGAAAGAAAATTGTGTCTTCATTTACAAAATTGAGTACCGACTATT
TCAAACAATTGTCAGACAAACCTACTAATATCAGATATGTCGGTGGTTCCGTTGAGGCTTCTTGTGCCTTGGGTGTTGCTGATGCTATTGTCGATTTGGTTGAAAGTGGTGAA
ACTATGAAAGCAGCTGGGTTAAAGGCCATAGAAACCATATTGGAGACTTCGGCTCATTTGATTTCGTCAAAAAAAAGTAAATTCCCAGAAATGGTCAATATAATCGTCCAAAG
ACTTCAAGGTGTTTTAGCTGCCCAGGAATATGTCTTGTGTAACTACAATGCCCCAAAATCCATCCAAGCAAAATGTTTAACCATTACTCCAGGCAGAAGGGCCGCTACTGTCT
CTACTTTGGACAAACACAGCGACGACGAAGAGGACTGGGTTGCCATCTCATCCATGGTTAATAGAAAGGAAATCGGTAATGTAATGGACGAATTGAAGAAAGCTGGTGCAAC
CGATATATTGGTACTCGAGATATCAAATTGTAGAGTTTAAAAGAAGTGATAGTTTCTCATAAATATTATACATATATACTTATAGATCTACTTCAATTTGACTCAATCTGTCTTAT
ACCATCTTTATCTACCACCTTGATGTACACACCTTTAAAGTCAATAGGCATTCTCGTTTAAACGAGCTCAACATGTTGACTCTTCCTTGTCTGCTTTGTTTTTCGGTATTTTTTTT
TTATTTCTTTGCCTTTATGTATCTATATTAAATGTACAAAATATATTACATGATATTCCAAATATCTTGCTTTCTTAAAAGTCTTTGAAAACTCAAACTAGCTATGGCCCTGTTGTA
TTCAATAAAAATTACAATGCATGATAGTAAACACACTAACAATACCCTTGACATGAGTCAAAATCAACCGCGGATCTGCCGGTCTCCCTATAGTGAGTCGTATTAATTTCGATA
AGCCAGGTTAACCTGCATTAATGAATCGGCCAACGCGCGGGGAGAGGCGGTTTGCGTATTGGGCGCTCTTCCGCTTCCTCGCTCACTGACTCGCTGCGCTCGGTCGTTCG
GCTGCGGCGAGCGGTATCAGCTCACTCAAAGGCGGTAATACGGTTATCCACAGAATCAGGGGATAACGCAGGAAAGAACATGTGAGCAAAAGGCCAGCAAAAGGCCAGGA
ACCGTAAAAAGGCCGCGTTGCTGGCGTTTTTCCATAGGCTCCGCCCCCCTGACGAGCATCACAAAAATCGACGCTCAAGTCAGAGGTGGCGAAACCCGACAGGACTATAAA
GATACCAGGCGTTTCCCCCTGGAAGCTCCCTCGTGCGCTCTCCTGTTCCGACCCTGCCGCTTACCGGATACCTGTCCGCCTTTCTCCCTTCGGGAAGCGTGGCGCTTTCTC
ATAGCTCACGCTGTAGGTATCTCAGTTCGGTGTAGGTCGTTCGCTCCAAGCTGGGCTGTGTGCACGAACCCCCCGTTCAGCCCGACCGCTGCGCCTTATCCGGTAACTATC
GTCTTGAGTCCAACCCGGTAAGACACGACTTATCGCCACTGGCAGCAGCCACTGGTAACAGGATTAGCAGAGCGAGGTATGTAGGCGGTGCTACAGAGTTCTTGAAGTGGT
GGCCTAACTACGGCTACACTAGAAGAACAGTATTTGGTATCTGCGCTCTGCTGAAGCCAGTTACCTTCGGAAAAAGAGTTGGTAGCTCTTGATCCGGCAAACAAACCACCGC
TGGTAGCGGTGGTTTTTTTGTTTGCAAGCAGCAGATTACGCGCAGAAAAAAAGGATCTCAA

*TPK2* promoter; GFP+GGGGlinker; *TPK1N+TPK2C* coding sequence; *HIS1* marker (replaced with a *SAT1* marker in pLC1686); *TPK2* 3' HR

>pLC1672_pFA-pTpk2-GFP-1FL-HIS

GAAGATCCTTTGATCTTTTCTACGGGGTCTGACGCTCAGTGGAACGAAAACTCACGTTAAGGGATTTTGGTCATGAGATTATCAAAAAGGATCTTCACCTAGATCCTTTTAAAT
TAAAAATGAAGTTTTAAATCAATCTAAAGTATATATGAGTAAACTTGGTCTGACAGTTACCAATGCTTAATCAGTGAGGCACCTATCTCAGCGATCTGTCTATTTCGTTCATCCA
TAGTTGCCTGACTCCCCGTCGTGTAGATAACTACGATACGGGAGGGCTTACCATCTGGCCCCAGTGCTGCAATGATACCGCGAGACCCACGCTCACCGGCTCCAGATTTAT
CAGCAATAAACCAGCCAGCCGGAAGGGCCGAGCGCAGAAGTGGTCCTGCAACTTTATCCGCCTCCATCCAGTCTATTAATTGTTGCCGGGAAGCTAGAGTAAGTAGTTCGC
CAGTTAATAGTTTGCGCAACGTTGTTGCCATTGCTACAGGCATCGTGGTGTCACGCTCGTCGTTTGGTATGGCTTCATTCAGCTCCGGTTCCCAACGATCAAGGCGAGTTAC
ATGATCCCCCATGTTGTGCAAAAAAGCGGTTAGCTCCTTCGGTCCTCCGATCGTTGTCAGAAGTAAGTTGGCCGCAGTGTTATCACTCATGGTTATGGCAGCACTGCATAATT
CTCTTACTGTCATGCCATCCGTAAGATGCTTTTCTGTGACTGGTGAGTACTCAACCAAGTCATTCTGAGAATAGTGTATGCGGCGACCGAGTTGCTCTTGCCCGGCGTCAATA
CGGGATAATACCGCGCCACATAGCAGAACTTTAAAAGTGCTCATCATTGGAAAACGTTCTTCGGGGCGAAAACTCTCAAGGATCTTACCGCTGTTGAGATCCAGTTCGATGT
AACCCACTCGTGCACCCAACTGATCTTCAGCATCTTTTACTTTCACCAGCGTTTCTGGGTGAGCAAAAACAGGAAGGCAAAATGCCGCAAAAAAGGGAATAAGGGCGACACG
GAAATGTTGAATACTCATACTCTTCCTTTTTCAATATTATTGAAGCATTTATCAGGGTTATTGTCTCATGAGCGGATACATATTTGAATGTATTTAGAAAAATAAACAAATAGGG
GTTCCGCGCACATTTCCCCGAAAAGTGCCACCTGACGTCTAAGAAACCATTATTATCATGACATTAACCTATAAAAATAGGCGTATCACGAGGCCCTTTCGTCTCGCGCGTTT
CGGTGATGACGGTGAAAACCTCTGACACATGCAGCTCCCGGAGACGGTCACAGCTTGTCTGTAAGCGGATGCCGGGAGCAGACAAGCCCGTCAGGGCGCGTCAGCGGGT
GTTGGCGGGTGTCGGGGCTGGCTTAACTATGCGGCATCAGAGCAGATTGTACTGAGAGTGCACCATATGGACATATTGTCGTTAGAACGCGGCTACAATTAATACATAACCT
TATGTATCATACACATACGATTTAGGTGACACTATAGAACGCGGCCGCCAGCTGAAGCTTCGTACGCTGCAGGTCGACGGATCCACATTGTTAAAAGTCGATACTTCAGTTG
CTTTTTTTTATTATTTTCTATCCAGCAAAAAACACATTGTCATTGTTTGTGTATCATTTTTTGTCCAGATAACAAACACCAAATTGAGGTCAACTAAACAACAACTTGTATAGTTCT
ACCACACTATTTTGAAGAGTCAGCTACAGTTCCTAGTTTCTCAACATAATATATCAACCTATCAATACTCGGATTGCAGGTCAATTTAGTTTACAACCACCAATTGCCAAAGTAT
TGGATTCTACTTTTTCCGACTTGTAAGTGAACACAAAGAAGATTAGACAAACAATCACCCACTCACACCTACTACTCACCCAATTTCCATTCCACCTTTTTTTTTCGAAAAAAAT
AACCTTCCCCCTTTTTGATGGTGAATCTTTTAAAGAAACTTCACATCACCAAGCTGCATCAACTGAATCAATCCAATTCGGACAGTAATTCCTTAAACTCAAACACATCAATGTC
TAAAGGTGAAGAATTATTCACTGGTGTTGTCCCAATTTTGGTTGAATTAGATGGTGATGTTAATGGTCACAAATTTTCTGTCTCCGGTGAAGGTGAAGGTGATGCTACTTACGG
TAAATTGACCTTAAAATTTATTTGTACTACTGGTAAATTGCCAGTTCCATGGCCAACCTTAGTCACTACTTTCGGTTATGGTGTTCAATGTTTTGCGAGATACCCAGATCATATG
AAACAACATGACTTTTTCAAGTCTGCCATGCCAGAAGGTTATGTTCAAGAAAGAACTATTTTTTTCAAAGATGACGGTAACTACAAGACCAGAGCTGAAGTCAAGTTTGAAGGT
GATACCTTAGTTAATAGAATCGAATTAAAAGGTATTGATTTTAAAGAAGATGGTAACATTTTAGGTCACAAATTGGAATACAACTATAACTCTCACAATGTTTACATCATGGCTG
ACAAACAAAAGAATGGTATCAAAGTTAACTTCAAAATTAGACACAACATTGAAGATGGTTCTGTTCAATTAGCTGACCATTATCAACAAAATACTCCAATTGGTGATGGTCCAG
TCTTGTTACCAGACAACCATTACTTATCCACTCAATCTGCCTTATCCAAAGATCCAAACGAAAAGAGAGACCACATGGTCTTGTTAGAATTTGTTACTGCTGCTGGTATTACCC
ATGGTATGGATGAATTGTACAAAGGTGGTGGTACATCCATGGAACCAGCAGACACAAGCATCAGGTCATTAAACGACATCAACTTACAAGAACTTGCCAACAAACAACA
TCAACGAAATATATATGCTCAAGGATCACCTACACTAGAAGATTCAATAACATCAAATGGTAATGATATTAATAATCCCAACAATAATATAAATAAAACCAATAATAACAGTAATT
CAAGTCTTTCCTTGACCAAGAAAACATCAAGACATATCAATAAAGACACTACAACTAAAGGCAAATATACATTAAATGATTTTCAAATATTACGAACATTAGGAACAGGTTCCTT
TGGTAGAGTTCATTTAACTCGATCGATTCATAATGGTAGATTTTATGCTATGAAAGTATTAAAGAAACAACGAGTAGTACAAATGAAACAAATCGAACATACTAATGATGAAAGA
AGAATGTTAAAATTAGCTCAACATCCATTTATAATTCGAATGTGGGGGACATTCCAAGATTGTCATAATTTATTTATGATTATGGATTATATAGAAGGAGGTGAATTATTTTCATT
ATTAAGAAAATCCCAACGATTCCCTACTCCAGTAGCTAAATTTTATGCTGCCGAAGTATTTTTGGCTATTGAATATTTACACAGTTTAGACATTATTTATCGAGATTTAAAACCA
GAAAATATATTATTGGATAAAAATGGTCATATCAAATTAACTGATTTTGGATTTGCTAAAGAAGTTCAAGATGTGACTTATACTTTATGTGGGACTCCAGATTATATTGCTCCTG
AAGTGGTGGCAACAAAACCTTATAATAAATCAGTTGATTGGTGGTCATTTGGTATATTAATATTTGAAATGTTAACTGGTTATACTCCATTCTATGATCCTACTCCAATGAAAAC
TTATGAAAACATTTTAAATGGATCAATTACTTATCCTGATTATTTACCTCCAGATATATTGGATCTTCTACAAAAATTAATTGTCAAAGATTTAACTCAAAGATTAGGTAATTTACA
AGGTGGTTCTGATGATGTGAAAAATCATCCGTGGTTTAAAGAAGTTATTTGGGAAAGATTGTTGTCACGTGATATAGAAACTCCATATGAACCACCTATAACTTCTGGTGTTGG
TGATACTTCACAATTTGATCGATACCCAGAAGATAAAGATTTGGATTATGGTATAAGTGGAGTTGAAGACCCATATCGTGATCAATTCCAGGACTTTTAAATGTAAAAATAAAAT
TGTATTTATTGGCACAACAATGTTAATTATGATAACTAGTTTTATAGATGTATAATTAATTTGCAAGAAAGAAGAAGACGAAGATTTGATTAGTGAATTCTTTAAAGCTAAAAAAA
CACTTCTTGGTGTATCAAAAGTAATTTTGGCAAATCTTATAGACAGCTAAAATATAAGCAGTGTCTTATTTTTAAACCAATGATCACTATATGAATTGGCTTCCGGCGCGCCATT
ATAAGTAAATGCATGTATACTAAACTCACAAATTAGAGCTTCAATTTAATTATATCAGTTATTACCCGGGAATCTCGGTCGTAATGATTTCTATAATGACGAAAAAAAAAAAATTG
GAAAGAAAAAGCTTCATGGCCTTTATAAAAAGGAACTATCCAATACCTCGCCAGAACCAAGTAACAGTATTTTACGGGGCACAAATCAAGAACAATAAGACAGGACTGTAAAG
ATGGACGCATAGATCCTGGAGGATGAGGAGACAGAAGTTAGTAGTAACAATTTGGGGACAGAAGAGGATACAGTGACATCAGAGGAAGAAGACAGCGAAGGTATAGTCAAA
AAGCAATAGGACAGCAAGACAATTTATTTATTTAATAGTTTTTTATAAATGTAAAATCTATCACAACATGTGTACTTAATTTTCTGTGGTAGGGGAGGCCATTTCGTTGAAACGT
GTCGCGAGCGGTTGTGAGAATTGACTCATTTTGATTATTACGCAACCATTGAAATTTTTTATTTTTTTTTGGTGAAGATTTTTCCCACACAACTTCTTCTTTTACTTAACCACCAA
CTACCGATAATGGATTTAGTCAATCATTTACCAGACCGTTTGTTATTTGCTGTTCCTAAAAAGGGCAGATTATACGAAAAATGCTGTAACTTATTGAGTGGTGCCGATATACAG
TTTAGAAGATCTAATAGATTAGATATAGCACTTTCTACAAACTTGCCAATTGCATTAATCTTCTTGCCTGCAGCTGATATCCCAGTTTTCGTTGGAGAAGGTAATTGTGACTTGG
GTATAACTGGGTTAGACCAAATCAAAGAAGCTGAACAATTCGACAACATCGAGGACTTGTTGGATTTGAAATTTGGTTCATGCAAATTGCAGATCCAAGTTCCAGCAGATGGC
GAGTACGAAAAGCCAGAACAGCTTGTTGGAAAGAAAATTGTGTCTTCATTTACAAAATTGAGTACCGACTATTTCAAACAATTGTCAGACAAACCTACTAATATCAGATATGTC
GGTGGTTCCGTTGAGGCTTCTTGTGCCTTGGGTGTTGCTGATGCTATTGTCGATTTGGTTGAAAGTGGTGAAACTATGAAAGCAGCTGGGTTAAAGGCCATAGAAACCATAT
TGGAGACTTCGGCTCATTTGATTTCGTCAAAAAAAAGTAAATTCCCAGAAATGGTCAATATAATCGTCCAAAGACTTCAAGGTGTTTTAGCTGCCCAGGAATATGTCTTGTGTA
ACTACAATGCCCCAAAATCCATCCAAGCAAAATGTTTAACCATTACTCCAGGCAGAAGGGCCGCTACTGTCTCTACTTTGGACAAACACAGCGACGACGAAGAGGACTGGGT
TGCCATCTCATCCATGGTTAATAGAAAGGAAATCGGTAATGTAATGGACGAATTGAAGAAAGCTGGTGCAACCGATATATTGGTACTCGAGATATCAAATTGTAGAGTTTAAA
AGAAGTGATAGTTTCTCATAAATATTATACATATATACTTATAGATCTACTTCAATTTGACTCAATCTGTCTTATACCATCTTTATCTACCACCTTGATGTACACACCTTTAAAGTC
AATAGGCATTCTCGTTTAAACGAGCTCAACATGTTGACTCTTCCTTGTCTGCTTTGTTTTTCGGTATTTTTTTTTTATTTCTTTGCCTTTATGTATCTATATTAAATGTACAAAATA
TATTACATGATATTCCAAATATCTTGCTTTCTTAAAAGTCTTTGAAAACTCAAACTAGCTATGGCCCTGTTGTATTCAATAAAAATTACAATGCATGATAGTAAACACACTAACAA
TACCCTTGACATGAGTCAAAATCAACCGCGGATCTGCCGGTCTCCCTATAGTGAGTCGTATTAATTTCGATAAGCCAGGTTAACCTGCATTAATGAATCGGCCAACGCGCGG
GGAGAGGCGGTTTGCGTATTGGGCGCTCTTCCGCTTCCTCGCTCACTGACTCGCTGCGCTCGGTCGTTCGGCTGCGGCGAGCGGTATCAGCTCACTCAAAGGCGGTAATA
CGGTTATCCACAGAATCAGGGGATAACGCAGGAAAGAACATGTGAGCAAAAGGCCAGCAAAAGGCCAGGAACCGTAAAAAGGCCGCGTTGCTGGCGTTTTTCCATAGGCTC
CGCCCCCCTGACGAGCATCACAAAAATCGACGCTCAAGTCAGAGGTGGCGAAACCCGACAGGACTATAAAGATACCAGGCGTTTCCCCCTGGAAGCTCCCTCGTGCGCTC
TCCTGTTCCGACCCTGCCGCTTACCGGATACCTGTCCGCCTTTCTCCCTTCGGGAAGCGTGGCGCTTTCTCATAGCTCACGCTGTAGGTATCTCAGTTCGGTGTAGGTCGTT
CGCTCCAAGCTGGGCTGTGTGCACGAACCCCCCGTTCAGCCCGACCGCTGCGCCTTATCCGGTAACTATCGTCTTGAGTCCAACCCGGTAAGACACGACTTATCGCCACTG
GCAGCAGCCACTGGTAACAGGATTAGCAGAGCGAGGTATGTAGGCGGTGCTACAGAGTTCTTGAAGTGGTGGCCTAACTACGGCTACACTAGAAGAACAGTATTTGGTATC
TGCGCTCTGCTGAAGCCAGTTACCTTCGGAAAAAGAGTTGGTAGCTCTTGATCCGGCAAACAAACCACCGCTGGTAGCGGTGGTTTTTTTGTTTGCAAGCAGCAGATTACGC
GCAGAAAAAAAGGATCTCAA

*TPK2* promoter; GFP+GGGGlinker; *TPK1* coding sequence; *HIS1* marker (replaced with a *SAT1* marker in pLC1687); *TPK2* 3’ HR

>pLC1677_pFA-pTpk2-GFP-2N1C-HIS

GAAGATCCTTTGATCTTTTCTACGGGGTCTGACGCTCAGTGGAACGAAAACTCACGTTAAGGGATTTTGGTCATGAGATTATCAAAAAGGATCTTCACCTAGATCCTTTTAAAT
TAAAAATGAAGTTTTAAATCAATCTAAAGTATATATGAGTAAACTTGGTCTGACAGTTACCAATGCTTAATCAGTGAGGCACCTATCTCAGCGATCTGTCTATTTCGTTCATCCA
TAGTTGCCTGACTCCCCGTCGTGTAGATAACTACGATACGGGAGGGCTTACCATCTGGCCCCAGTGCTGCAATGATACCGCGAGACCCACGCTCACCGGCTCCAGATTTAT
CAGCAATAAACCAGCCAGCCGGAAGGGCCGAGCGCAGAAGTGGTCCTGCAACTTTATCCGCCTCCATCCAGTCTATTAATTGTTGCCGGGAAGCTAGAGTAAGTAGTTCGC
CAGTTAATAGTTTGCGCAACGTTGTTGCCATTGCTACAGGCATCGTGGTGTCACGCTCGTCGTTTGGTATGGCTTCATTCAGCTCCGGTTCCCAACGATCAAGGCGAGTTAC
ATGATCCCCCATGTTGTGCAAAAAAGCGGTTAGCTCCTTCGGTCCTCCGATCGTTGTCAGAAGTAAGTTGGCCGCAGTGTTATCACTCATGGTTATGGCAGCACTGCATAATT
CTCTTACTGTCATGCCATCCGTAAGATGCTTTTCTGTGACTGGTGAGTACTCAACCAAGTCATTCTGAGAATAGTGTATGCGGCGACCGAGTTGCTCTTGCCCGGCGTCAATA
CGGGATAATACCGCGCCACATAGCAGAACTTTAAAAGTGCTCATCATTGGAAAACGTTCTTCGGGGCGAAAACTCTCAAGGATCTTACCGCTGTTGAGATCCAGTTCGATGT
AACCCACTCGTGCACCCAACTGATCTTCAGCATCTTTTACTTTCACCAGCGTTTCTGGGTGAGCAAAAACAGGAAGGCAAAATGCCGCAAAAAAGGGAATAAGGGCGACACG
GAAATGTTGAATACTCATACTCTTCCTTTTTCAATATTATTGAAGCATTTATCAGGGTTATTGTCTCATGAGCGGATACATATTTGAATGTATTTAGAAAAATAAACAAATAGGG
GTTCCGCGCACATTTCCCCGAAAAGTGCCACCTGACGTCTAAGAAACCATTATTATCATGACATTAACCTATAAAAATAGGCGTATCACGAGGCCCTTTCGTCTCGCGCGTTT
CGGTGATGACGGTGAAAACCTCTGACACATGCAGCTCCCGGAGACGGTCACAGCTTGTCTGTAAGCGGATGCCGGGAGCAGACAAGCCCGTCAGGGCGCGTCAGCGGGT
GTTGGCGGGTGTCGGGGCTGGCTTAACTATGCGGCATCAGAGCAGATTGTACTGAGAGTGCACCATATGGACATATTGTCGTTAGAACGCGGCTACAATTAATACATAACCT
TATGTATCATACACATACGATTTAGGTGACACTATAGAACGCGGCCGCCAGCTGAAGCTTCGTACGCTGCAGGTCGACGGATCCACATTGTTAAAAGTCGATACTTCAGTTG
CTTTTTTTTATTATTTTCTATCCAGCAAAAAACACATTGTCATTGTTTGTGTATCATTTTTTGTCCAGATAACAAACACCAAATTGAGGTCAACTAAACAACAACTTGTATAGTTCT
ACCACACTATTTTGAAGAGTCAGCTACAGTTCCTAGTTTCTCAACATAATATATCAACCTATCAATACTCGGATTGCAGGTCAATTTAGTTTACAACCACCAATTGCCAAAGTAT
TGGATTCTACTTTTTCCGACTTGTAAGTGAACACAAAGAAGATTAGACAAACAATCACCCACTCACACCTACTACTCACCCAATTTCCATTCCACCTTTTTTTTTCGAAAAAAAT
AACCTTCCCCCTTTTTGATGGTGAATCTTTTAAAGAAACTTCACATCACCAAGCTGCATCAACTGAATCAATCCAATTCGGACAGTAATTCCTTAAACTCAAACACATCAATGTC
TAAAGGTGAAGAATTATTCACTGGTGTTGTCCCAATTTTGGTTGAATTAGATGGTGATGTTAATGGTCACAAATTTTCTGTCTCCGGTGAAGGTGAAGGTGATGCTACTTACGG
TAAATTGACCTTAAAATTTATTTGTACTACTGGTAAATTGCCAGTTCCATGGCCAACCTTAGTCACTACTTTCGGTTATGGTGTTCAATGTTTTGCGAGATACCCAGATCATATG
AAACAACATGACTTTTTCAAGTCTGCCATGCCAGAAGGTTATGTTCAAGAAAGAACTATTTTTTTCAAAGATGACGGTAACTACAAGACCAGAGCTGAAGTCAAGTTTGAAGGT
GATACCTTAGTTAATAGAATCGAATTAAAAGGTATTGATTTTAAAGAAGATGGTAACATTTTAGGTCACAAATTGGAATACAACTATAACTCTCACAATGTTTACATCATGGCTG
ACAAACAAAAGAATGGTATCAAAGTTAACTTCAAAATTAGACACAACATTGAAGATGGTTCTGTTCAATTAGCTGACCATTATCAACAAAATACTCCAATTGGTGATGGTCCAG
TCTTGTTACCAGACAACCATTACTTATCCACTCAATCTGCCTTATCCAAAGATCCAAACGAAAAGAGAGACCACATGGTCTTGTTAGAATTTGTTACTGCTGCTGGTATTACCC
ATGGTATGGATGAATTGTACAAAGGTGGTGGTGACAATCATCAACAACAACAGCAGCTTCAGCATCAGCAATATCAGCAACAATTTCAACAACCGCAGCAACAACTTTAT
CCAGGCGAACAAATAGTTCACCCTGCTGCAGCCCAAACTGGACAAAATACTACAAATGTAACAGCAGTGTCGAGCTCCAACATTACCCAATCTGCTACCTCATCACTCCATTC
ACAGCAATTGCAGCATGTAGATGTTTCCAAATCGGCTGCTGAAGAAGCCATCAGAAGATCGTTGTTGCCTGAACGTTCTACTGTTTCAAAAGGCAAATATACATTAAATGATTT
TCAAATATTACGAACATTAGGAACAGGTTCCTTTGGTAGAGTTCATTTAACTCGATCGATTCATAATGGTAGATTTTATGCTATGAAAGTATTAAAGAAACAACGAGTAGTACAA
ATGAAACAAATCGAACATACTAATGATGAAAGAAGAATGTTAAAATTAGCTCAACATCCATTTATAATTCGAATGTGGGGGACATTCCAAGATTGTCATAATTTATTTATGATTA
TGGATTATATAGAAGGAGGTGAATTATTTTCATTATTAAGAAAATCCCAACGATTCCCTACTCCAGTAGCTAAATTTTATGCTGCCGAAGTATTTTTGGCTATTGAATATTTACA
CAGTTTAGACATTATTTATCGAGATTTAAAACCAGAAAATATATTATTGGATAAAAATGGTCATATCAAATTAACTGATTTTGGATTTGCTAAAGAAGTTCAAGATGTGACTTATA
CTTTATGTGGGACTCCAGATTATATTGCTCCTGAAGTGGTGGCAACAAAACCTTATAATAAATCAGTTGATTGGTGGTCATTTGGTATATTAATATTTGAAATGTTAACTGGTTA
TACTCCATTCTATGATCCTACTCCAATGAAAACTTATGAAAACATTTTAAATGGATCAATTACTTATCCTGATTATTTACCTCCAGATATATTGGATCTTCTACAAAAATTAATTGT
CAAAGATTTAACTCAAAGATTAGGTAATTTACAAGGTGGTTCTGATGATGTGAAAAATCATCCGTGGTTTAAAGAAGTTATTTGGGAAAGATTGTTGTCACGTGATATAGAAAC
TCCATATGAACCACCTATAACTTCTGGTGTTGGTGATACTTCACAATTTGATCGATACCCAGAAGATAAAGATTTGGATTATGGTATAAGTGGAGTTGAAGACCCATATCGTGA
TCAATTCCAGGACTTTTAAATGTAAAAATAAAATTGTATTTATTGGCACAACAATGTTAATTATGATAACTAGTTTTATAGATGTATAATTAATTTGCAAGAAAGAAGAAGACGAA
GATTTGATTAGTGAATTTTTTAAAGCTAAAAAAACACTTCTTGGTGTATCAAAAGTAATTTTGGCAAATCTTATAGACAGCTAAAATATAAGCAGTGTCTTATTTTTAAACCAATG
ATCACTATATGAATTGGCTTCCGGCGCGCCATTATAAGTAAATGCATGTATACTAAACTCACAAATTAGAGCTTCAATTTAATTATATCAGTTATTACCCGGGAATCTCGGTCG
TAATGATTTCTATAATGACGAAAAAAAAAAAATTGGAAAGAAAAAGCTTCATGGCCTTTATAAAAAGGAACTATCCAATACCTCGCCAGAACCAAGTAACAGTATTTTACGGGGC
ACAAATCAAGAACAATAAGACAGGACTGTAAAGATGGACGCATAGATCCTGGAGGATGAGGAGACAGAAGTTAGTAGTAACAATTTGGGGACAGAAGAGGATACAGTGACAT
CAGAGGAAGAAGACAGCGAAGGTATAGTCAAAAAGCAATAGGACAGCAAGACAATTTATTTATTTAATAGTTTTTTATAAATGTAAAATCTATCACAACATGTGTACTTAATTTT
CTGTGGTAGGGGAGGCCATTTCGTTGAAACGTGTCGCGAGCGGTTGTGAGAATTGACTCATTTTGATTATTACGCAACCATTGAAATTTTTTATTTTTTTTTGGTGAAGATTTT
TCCCACACAACTTCTTCTTTTACTTAACCACCAACTACCGATAATGGATTTAGTCAATCATTTACCAGACCGTTTGTTATTTGCTGTTCCTAAAAAGGGCAGATTATACGAAAAA
TGCTGTAACTTATTGAGTGGTGCCGATATACAGTTTAGAAGATCTAATAGATTAGATATAGCACTTTCTACAAACTTGCCAATTGCATTAATCTTCTTGCCTGCAGCTGATATCC
CAGTTTTCGTTGGAGAAGGTAATTGTGACTTGGGTATAACTGGGTTAGACCAAATCAAAGAAGCTGAACAATTCGACAACATCGAGGACTTGTTGGATTTGAAATTTGGTTCA
TGCAAATTGCAGATCCAAGTTCCAGCAGATGGCGAGTACGAAAAGCCAGAACAGCTTGTTGGAAAGAAAATTGTGTCTTCATTTACAAAATTGAGTACCGACTATTTCAAACA
ATTGTCAGACAAACCTACTAATATCAGATATGTCGGTGGTTCCGTTGAGGCTTCTTGTGCCTTGGGTGTTGCTGATGCTATTGTCGATTTGGTTGAAAGTGGTGAAACTATGA
AAGCAGCTGGGTTAAAGGCCATAGAAACCATATTGGAGACTTCGGCTCATTTGATTTCGTCAAAAAAAAGTAAATTCCCAGAAATGGTCAATATAATCGTCCAAAGACTTCAA
GGTGTTTTAGCTGCCCAGGAATATGTCTTGTGTAACTACAATGCCCCAAAATCCATCCAAGCAAAATGTTTAACCATTACTCCAGGCAGAAGGGCCGCTACTGTCTCTACTTT
GGACAAACACAGCGACGACGAAGAGGACTGGGTTGCCATCTCATCCATGGTTAATAGAAAGGAAATCGGTAATGTAATGGACGAATTGAAGAAAGCTGGTGCAACCGATAT
ATTGGTACTCGAGATATCAAATTGTAGAGTTTAAAAGAAGTGATAGTTTCTCATAAATATTATACATATATACTTATAGATCTACTTCAATTTGACTCAATCTGTCTTATACCATC
TTTATCTACCACCTTGATGTACACACCTTTAAAGTCAATAGGCATTCTCGTTTAAACGAGCTCAACATGTTGACTCTTCCTTGTCTGCTTTGTTTTTCGGTATTTTTTTTTTATTT
CTTTGCCTTTATGTATCTATATTAAATGTACAAAATATATTACATGATATTCCAAATATCTTGCTTTCTTAAAAGTCTTTGAAAACTCAAACTAGCTATGGCCCTGTTGTATTCAAT
AAAAATTACAATGCATGATAGTAAACACACTAACAATACCCTTGACATGAGTCAAAATCAACCGCGGATCTGCCGGTCTCCCTATAGTGAGTCGTATTAATTTCGATAAGCCA
GGTTAACCTGCATTAATGAATCGGCCAACGCGCGGGGAGAGGCGGTTTGCGTATTGGGCGCTCTTCCGCTTCCTCGCTCACTGACTCGCTGCGCTCGGTCGTTCGGCTGC
GGCGAGCGGTATCAGCTCACTCAAAGGCGGTAATACGGTTATCCACAGAATCAGGGGATAACGCAGGAAAGAACATGTGAGCAAAAGGCCAGCAAAAGGCCAGGAACCGT
AAAAAGGCCGCGTTGCTGGCGTTTTTCCATAGGCTCCGCCCCCCTGACGAGCATCACAAAAATCGACGCTCAAGTCAGAGGTGGCGAAACCCGACAGGACTATAAAGATAC
CAGGCGTTTCCCCCTGGAAGCTCCCTCGTGCGCTCTCCTGTTCCGACCCTGCCGCTTACCGGATACCTGTCCGCCTTTCTCCCTTCGGGAAGCGTGGCGCTTTCTCATAGC
TCACGCTGTAGGTATCTCAGTTCGGTGTAGGTCGTTCGCTCCAAGCTGGGCTGTGTGCACGAACCCCCCGTTCAGCCCGACCGCTGCGCCTTATCCGGTAACTATCGTCTT
GAGTCCAACCCGGTAAGACACGACTTATCGCCACTGGCAGCAGCCACTGGTAACAGGATTAGCAGAGCGAGGTATGTAGGCGGTGCTACAGAGTTCTTGAAGTGGTGGCC
TAACTACGGCTACACTAGAAGAACAGTATTTGGTATCTGCGCTCTGCTGAAGCCAGTTACCTTCGGAAAAAGAGTTGGTAGCTCTTGATCCGGCAAACAAACCACCGCTGGT
AGCGGTGGTTTTTTTGTTTGCAAGCAGCAGATTACGCGCAGAAAAAAAGGATCTCAA

*TPK2* promoter; GFP+GGGlinker; *TPK2N+TPK1C* coding sequence; *HIS1* marker (replaced with a *SAT1* marker in pLC1689); *TPK2* 3’ HR

>pLC1717_pFA-p2-GFP-stop2FL-HIS

GAAGATCCTTTGATCTTTTCTACGGGGTCTGACGCTCAGTGGAACGAAAACTCACGTTAAGGGATTTTGGTCATGAGATTATCAAAAAGGATCTTCACCTAGATCCTTTTAAAT
TAAAAATGAAGTTTTAAATCAATCTAAAGTATATATGAGTAAACTTGGTCTGACAGTTACCAATGCTTAATCAGTGAGGCACCTATCTCAGCGATCTGTCTATTTCGTTCATCCA
TAGTTGCCTGACTCCCCGTCGTGTAGATAACTACGATACGGGAGGGCTTACCATCTGGCCCCAGTGCTGCAATGATACCGCGAGACCCACGCTCACCGGCTCCAGATTTAT
CAGCAATAAACCAGCCAGCCGGAAGGGCCGAGCGCAGAAGTGGTCCTGCAACTTTATCCGCCTCCATCCAGTCTATTAATTGTTGCCGGGAAGCTAGAGTAAGTAGTTCGC
CAGTTAATAGTTTGCGCAACGTTGTTGCCATTGCTACAGGCATCGTGGTGTCACGCTCGTCGTTTGGTATGGCTTCATTCAGCTCCGGTTCCCAACGATCAAGGCGAGTTAC
ATGATCCCCCATGTTGTGCAAAAAAGCGGTTAGCTCCTTCGGTCCTCCGATCGTTGTCAGAAGTAAGTTGGCCGCAGTGTTATCACTCATGGTTATGGCAGCACTGCATAATT
CTCTTACTGTCATGCCATCCGTAAGATGCTTTTCTGTGACTGGTGAGTACTCAACCAAGTCATTCTGAGAATAGTGTATGCGGCGACCGAGTTGCTCTTGCCCGGCGTCAATA
CGGGATAATACCGCGCCACATAGCAGAACTTTAAAAGTGCTCATCATTGGAAAACGTTCTTCGGGGCGAAAACTCTCAAGGATCTTACCGCTGTTGAGATCCAGTTCGATGT
AACCCACTCGTGCACCCAACTGATCTTCAGCATCTTTTACTTTCACCAGCGTTTCTGGGTGAGCAAAAACAGGAAGGCAAAATGCCGCAAAAAAGGGAATAAGGGCGACACG
GAAATGTTGAATACTCATACTCTTCCTTTTTCAATATTATTGAAGCATTTATCAGGGTTATTGTCTCATGAGCGGATACATATTTGAATGTATTTAGAAAAATAAACAAATAGGG
GTTCCGCGCACATTTCCCCGAAAAGTGCCACCTGACGTCTAAGAAACCATTATTATCATGACATTAACCTATAAAAATAGGCGTATCACGAGGCCCTTTCGTCTCGCGCGTTT
CGGTGATGACGGTGAAAACCTCTGACACATGCAGCTCCCGGAGACGGTCACAGCTTGTCTGTAAGCGGATGCCGGGAGCAGACAAGCCCGTCAGGGCGCGTCAGCGGGT
GTTGGCGGGTGTCGGGGCTGGCTTAACTATGCGGCATCAGAGCAGATTGTACTGAGAGTGCACCATATGGACATATTGTCGTTAGAACGCGGCTACAATTAATACATAACCT
TATGTATCATACACATACGATTTAGGTGACACTATAGAACGCGGCCGCCAGCTGAAGCTTCGTACGCTGCAGGTCGACGGATCCACATTGTTAAAAGTCGATACTTCAGTTG
CTTTTTTTTATTATTTTCTATCCAGCAAAAAACACATTGTCATTGTTTGTGTATCATTTTTTGTCCAGATAACAAACACCAAATTGAGGTCAACTAAACAACAACTTGTATAGTTCT
ACCACACTATTTTGAAGAGTCAGCTACAGTTCCTAGTTTCTCAACATAATATATCAACCTATCAATACTCGGATTGCAGGTCAATTTAGTTTACAACCACCAATTGCCAAAGTAT
TGGATTCTACTTTTTCCGACTTGTAAGTGAACACAAAGAAGATTAGACAAACAATCACCCACTCACACCTACTACTCACCCAATTTCCATTCCACCTTTTTTTTTCGAAAAAAAT
AACCTTCCCCCTTTTTGATGGTGAATCTTTTAAAGAAACTTCACATCACCAAGCTGCATCAACTGAATCAATCCAATTCGGACAGTAATTCCTTAAACTCAAACACATCATAAAT
GTCTAAAGGTGAAGAATTATTCACTGGTGTTGTCCCAATTTTGGTTGAATTAGATGGTGATGTTAATGGTCACAAATTTTCTGTCTCCGGTGAAGGTGAAGGTGATGCTACTTA
CGGTAAATTGACCTTAAAATTTATTTGTACTACTGGTAAATTGCCAGTTCCATGGCCAACCTTAGTCACTACTTTCGGTTATGGTGTTCAATGTTTTGCGAGATACCCAGATCAT
ATGAAACAACATGACTTTTTCAAGTCTGCCATGCCAGAAGGTTATGTTCAAGAAAGAACTATTTTTTTCAAAGATGACGGTAACTACAAGACCAGAGCTGAAGTCAAGTTTGAA
GGTGATACCTTAGTTAATAGAATCGAATTAAAAGGTATTGATTTTAAAGAAGATGGTAACATTTTAGGTCACAAATTGGAATACAACTATAACTCTCACAATGTTTACATCATGG
CTGACAAACAAAAGAATGGTATCAAAGTTAACTTCAAAATTAGACACAACATTGAAGATGGTTCTGTTCAATTAGCTGACCATTATCAACAAAATACTCCAATTGGTGATGGTC
CAGTCTTGTTACCAGACAACCATTACTTATCCACTCAATCTGCCTTATCCAAAGATCCAAACGAAAAGAGAGACCACATGGTCTTGTTAGAATTTGTTACTGCTGCTGGTATTA
CCCATGGTATGGATGAATTGTACAAAGGTGGTGGTGGTGACAATCATCAACAACAACAGCAGCTTCAGCATCAGCAATATCAGCAACAATTTCAACAACCGCAGCAACAACT
TTATCCAGGCGAACAAATAGTTCACCCTGCTGCAGCCCAAACTGGACAAAATACTACAAATGTAACAGCAGTGTCGAGCTCCAACATTACCCAATCTGCTACCTCATCACTCC
ATTCACAGCAATTGCAGCATGTAGATGTTTCCAAATCGGCTGCTGAAGAAGCCATCAGAAGATCGTTGTTGCCTGAACGTTCTACTGTTTCAAAGGGGAAATACTCTTTGACT
GATTTCTCAATTATGAGAACTTTGGGAACAGGTTCCTTTGGTAGAGTACATTTAGTGAGATCAGTTCACAATGGTAGATATTATGCTATCAAAGTTTTGAAGAAGCATCAAGTT
GTGAAAATGAAGCAAGTTGAACACACAAATGATGAAAGAAGAATGTTGAAGTTGGTTGAACATCCATTTTTGATTAGAATGTGGGGGACTTTCCAAGATTCCAAAAATTTATTT
ATGGTTATGGACTATATTGAAGGGGGAGAGTTATTTTCATTGTTAAGAAAGTCTCAGAGATTTCCCAATCCAGTAGCCAAGTTTTATGCTGCAGAGGTTACCTTGGCTTTGGA
GTATTTGCACAGTCATGATATTATCTATCGTGATTTGAAACCAGAAAATATATTATTGGATAGAAATGGTCATATAAAGATTACAGATTTTGGATTTGCCAAAGAAGTTAGTACC
GTTACATGGACGTTATGTGGTACCCCCGATTATATTGCCCCAGAAGTCATAACAACCAAACCCTATAACAAGTCGGTGGACTGGTGGTCGTTGGGGGTATTGATTTTTGAAAT
GTTGGCAGGTTACACTCCATTTTATGATTCGACTCCAATGAAAACTTATGAAAAAATTTTGGCAGGCAAAATACATTACCCAAGTTTTTTTCAACCTGATGTGATTGATTTGTTG
ACTAAATTAATAACTGCTGATTTGACAAGAAGATTGGGTAATTTGATCAACGGACCAGCAGATATCAGAAATCATCCCTGGTTTCTGGAAGTGGTATGGGAGAAATTGTTGGC
AAAGGATATTGAAACTCCTTATGAACCACCAATCACAGCTGGGGTTGGTGATTCGTCATTGTTTGACCATTATCCAGAAGAACAATTAGACTACGGAAGCCAAGGAGAAGATC
CTTATGCTCTGTATTTCCTTGACTTTTGAATTGAGAGGAGATGAACTTTCATAAATGAATAAATGAGGCGCGCCATTATAAGTAAATGCATGTATACTAAACTCACAAATTAGAG
CTTCAATTTAATTATATCAGTTATTACCCGGGAATCTCGGTCGTAATGATTTCTATAATGACGAAAAAAAAAAAAATTGGAAAGAAAAAGCTTCATGGCCTTTATAAAAAGGAACT
ATCCAATACCTCGCCAGAACCAAGTAACAGTATTTTACGGGGCACAAATCAAGAACAATAAGACAGGACTGTAAAGATGGACGCATAGATCCTGGAGGATGAGGAGACAGAA
GTTAGTAGTAACAATTTGGGGACAGAAGAGGATACAGTGACATCAGAGGAAGAAGACAGCGAAGGTATAGTCAAAAAGCAATAGGACAGCAAGACAATTTATTTATTTAATAG
TTTTTTATAAATGTAAAATCTATCACAACATGTGTACTTAATTTTCTGTGGTAGGGGAGGCCATTTCGTTGAAACGTGTCGCGAGCGGTTGTGAGAATTGACTCATTTTGATTAT
TACGCAACCATTGAAATTTTTTATTTTTTTTTGGTGAAGATTTTTCCCACACAACTTCTTCTTTTACTTAACCACCAACTACCGATAATGGATTTAGTCAATCATTTACCAGACCG
TTTGTTATTTGCTGTTCCTAAAAAGGGCAGATTATACGAAAAATGCTGTAACTTATTGAGTGGTGCCGATATACAGTTTAGAAGATCTAATAGATTAGATATAGCACTTTCTACA
AACTTGCCAATTGCATTAATCTTCTTGCCTGCAGCTGATATCCCAGTTTTCGTTGGAGAAGGTAATTGTGACTTGGGTATAACTGGGTTAGACCAAATCAAAGAAGCTGAACA
ATTCGACAACATCGAGGACTTGTTGGATTTGAAATTTGGTTCATGCAAATTGCAGATCCAAGTTCCAGCAGATGGCGAGTACGAAAAGCCAGAACAGCTTGTTGGAAAGAAA
ATTGTGTCTTCATTTACAAAATTGAGTACCGACTATTTCAAACAATTGTCAGACAAACCTACTAATATCAGATATGTCGGTGGTTCCGTTGAGGCTTCTTGTGCCTTGGGTGTT
GCTGATGCTATTGTCGATTTGGTTGAAAGTGGTGAAACTATGAAAGCAGCTGGGTTAAAGGCCATAGAAACCATATTGGAGACTTCGGCTCATTTGATTTCGTCAAAAAAAAG
TAAATTCCCAGAAATGGTCAATATAATCGTCCAAAGACTTCAAGGTGTTTTAGCTGCCCAGGAATATGTCTTGTGTAACTACAATGCCCCAAAATCCATCCAAGCAAAATGTTT
AACCATTACTCCAGGCAGAAGGGCCGCTACTGTCTCTACTTTGGACAAACACAGCGACGACGAAGAGGACTGGGTTGCCATCTCATCCATGGTTAATAGAAAGGAAATCGG
TAATGTAATGGACGAATTGAAGAAAGCTGGTGCAACCGATATATTGGTACTCGAGATATCAAATTGTAGAGTTTAAAAGAAGTGATAGTTTCTCATAAATATTATACATATATAC
TTATAGATCTACTTCAATTTGACTCAATCTGTCTTATACCATCTTTATCTACCACCTTGATGTACACACCTTTAAAGTCAATAGGCATTCTCGTTTAAACGAGCTCAACATGTTGA
CTCTTCCTTGTCTGCTTTGTTTTTCGGTATTTTTTTTTTATTTCTTTGCCTTTATGTATCTATATTAAATGTACAAAATATATTACATGATATTCCAAATATCTTGCTTTCTTAAAAG
TCTTTGAAAACTCAAACTAGCTATGGCCCTGTTGTATTCAATAAAAATTACAATGCATGATAGTAAACACACTAACAATACCCTTGACATGAGTCAAAATCAACCGCGGATCTG
CCGGTCTCCCTATAGTGAGTCGTATTAATTTCGATAAGCCAGGTTAACCTGCATTAATGAATCGGCCAACGCGCGGGGAGAGGCGGTTTGCGTATTGGGCGCTCTTCCGCTT
CCTCGCTCACTGACTCGCTGCGCTCGGTCGTTCGGCTGCGGCGAGCGGTATCAGCTCACTCAAAGGCGGTAATACGGTTATCCACAGAATCAGGGGATAACGCAGGAAAG
AACATGTGAGCAAAAGGCCAGCAAAAGGCCAGGAACCGTAAAAAGGCCGCGTTGCTGGCGTTTTTCCATAGGCTCCGCCCCCCTGACGAGCATCACAAAAATCGACGCTCA
AGTCAGAGGTGGCGAAACCCGACAGGACTATAAAGATACCAGGCGTTTCCCCCTGGAAGCTCCCTCGTGCGCTCTCCTGTTCCGACCCTGCCGCTTACCGGATACCTGTCC
GCCTTTCTCCCTTCGGGAAGCGTGGCGCTTTCTCATAGCTCACGCTGTAGGTATCTCAGTTCGGTGTAGGTCGTTCGCTCCAAGCTGGGCTGTGTGCACGAACCCCCCGTT
CAGCCCGACCGCTGCGCCTTATCCGGTAACTATCGTCTTGAGTCCAACCCGGTAAGACACGACTTATCGCCACTGGCAGCAGCCACTGGTAACAGGATTAGCAGAGCGAG
GTATGTAGGCGGTGCTACAGAGTTCTTGAAGTGGTGGCCTAACTACGGCTACACTAGAAGAACAGTATTTGGTATCTGCGCTCTGCTGAAGCCAGTTACCTTCGGAAAAAGA
GTTGGTAGCTCTTGATCCGGCAAACAAACCACCGCTGGTAGCGGTGGTTTTTTTGTTTGCAAGCAGCAGATTACGCGCAGAAAAAAAGGATCTCAA

*TPK2* promoter; Stop codon; GFP+GGGGlinker; *TPK2* ORF; *HIS1* marker; *TPK2* 3’ HR

>pLC1718_pFA-p2-GFP-stop2N-HIS

GAAGATCCTTTGATCTTTTCTACGGGGTCTGACGCTCAGTGGAACGAAAACTCACGTTAAGGGATTTTGGTCATGAGATTATCAAAAAGGATCTTCACCTAGATCCTTTTAAAT
TAAAAATGAAGTTTTAAATCAATCTAAAGTATATATGAGTAAACTTGGTCTGACAGTTACCAATGCTTAATCAGTGAGGCACCTATCTCAGCGATCTGTCTATTTCGTTCATCCA
TAGTTGCCTGACTCCCCGTCGTGTAGATAACTACGATACGGGAGGGCTTACCATCTGGCCCCAGTGCTGCAATGATACCGCGAGACCCACGCTCACCGGCTCCAGATTTAT
CAGCAATAAACCAGCCAGCCGGAAGGGCCGAGCGCAGAAGTGGTCCTGCAACTTTATCCGCCTCCATCCAGTCTATTAATTGTTGCCGGGAAGCTAGAGTAAGTAGTTCGC
CAGTTAATAGTTTGCGCAACGTTGTTGCCATTGCTACAGGCATCGTGGTGTCACGCTCGTCGTTTGGTATGGCTTCATTCAGCTCCGGTTCCCAACGATCAAGGCGAGTTAC
ATGATCCCCCATGTTGTGCAAAAAAGCGGTTAGCTCCTTCGGTCCTCCGATCGTTGTCAGAAGTAAGTTGGCCGCAGTGTTATCACTCATGGTTATGGCAGCACTGCATAATT
CTCTTACTGTCATGCCATCCGTAAGATGCTTTTCTGTGACTGGTGAGTACTCAACCAAGTCATTCTGAGAATAGTGTATGCGGCGACCGAGTTGCTCTTGCCCGGCGTCAATA
CGGGATAATACCGCGCCACATAGCAGAACTTTAAAAGTGCTCATCATTGGAAAACGTTCTTCGGGGCGAAAACTCTCAAGGATCTTACCGCTGTTGAGATCCAGTTCGATGT
AACCCACTCGTGCACCCAACTGATCTTCAGCATCTTTTACTTTCACCAGCGTTTCTGGGTGAGCAAAAACAGGAAGGCAAAATGCCGCAAAAAAGGGAATAAGGGCGACACG
GAAATGTTGAATACTCATACTCTTCCTTTTTCAATATTATTGAAGCATTTATCAGGGTTATTGTCTCATGAGCGGATACATATTTGAATGTATTTAGAAAAATAAACAAATAGGG
GTTCCGCGCACATTTCCCCGAAAAGTGCCACCTGACGTCTAAGAAACCATTATTATCATGACATTAACCTATAAAAATAGGCGTATCACGAGGCCCTTTCGTCTCGCGCGTTT
CGGTGATGACGGTGAAAACCTCTGACACATGCAGCTCCCGGAGACGGTCACAGCTTGTCTGTAAGCGGATGCCGGGAGCAGACAAGCCCGTCAGGGCGCGTCAGCGGGT
GTTGGCGGGTGTCGGGGCTGGCTTAACTATGCGGCATCAGAGCAGATTGTACTGAGAGTGCACCATATGGACATATTGTCGTTAGAACGCGGCTACAATTAATACATAACCT
TATGTATCATACACATACGATTTAGGTGACACTATAGAACGCGGCCGCCAGCTGAAGCTTCGTACGCTGCAGGTCGACGGATCCACATTGTTAAAAGTCGATACTTCAGTTG
CTTTTTTTTATTATTTTCTATCCAGCAAAAAACACATTGTCATTGTTTGTGTATCATTTTTTGTCCAGATAACAAACACCAAATTGAGGTCAACTAAACAACAACTTGTATAGTTCT
ACCACACTATTTGAAGAGTCAGCTACAGTTCCTAGTTTCTCAACATAATATATCAACCTATCAATACTCGGATTGCAGGTCAATTTAGTTTACAACCACCAATTGCCAAAGTAT
TGGATTCTACTTTTTCCGACTTGTAAGTGAACACAAAGAAGATTAGACAAACAATCACCCACTCACACCTACTACTCACCCAATTTCCATTCCACCTTTTTTTTTCGAAAAAAAT
AACCTTCCCCCTTTTTGATGGTGAATCTTTTAAAGAAACTTCACATCACCAAGCTGCATCAACTGAATCAATCCAATTCGGACAGTAATTCCTTAAACTCAAACACATCATAAAT
GTCTAAAGGTGAAGAATTATTCACTGGTGTTGTCCCAATTTTGGTTGAATTAGATGGTGATGTTAATGGTCACAAATTTTCTGTCTCCGGTGAAGGTGAAGGTGATGCTACTTA
CGGTAAATTGACCTTAAAATTTATTTGTACTACTGGTAAATTGCCAGTTCCATGGCCAACCTTAGTCACTACTTTCGGTTATGGTGTTCAATGTTTTGCGAGATACCCAGATCAT
ATGAAACAACATGACTTTTTCAAGTCTGCCATGCCAGAAGGTTATGTTCAAGAAAGAACTATTTTTTTCAAAGATGACGGTAACTACAAGACCAGAGCTGAAGTCAAGTTTGAA
GGTGATACCTTAGTTAATAGAATCGAATTAAAAGGTATTGATTTTAAAGAAGATGGTAACATTTTAGGTCACAAATTGGAATACAACTATAACTCTCACAATGTTTACATCATGG
CTGACAAACAAAAGAATGGTATCAAAGTTAACTTCAAAATTAGACACAACATTGAAGATGGTTCTGTTCAATTAGCTGACCATTATCAACAAAATACTCCAATTGGTGATGGTC
CAGTCTTGTTACCAGACAACCATTACTTATCCACTCAATCTGCCTTATCCAAAGATCCAAACGAAAAGAGAGACCACATGGTCTTGTTAGAATTTGTTACTGCTGCTGGTATTA
CCCATGGTATGGATGAATTGTACAAAGGTGGTGGTGGTGACAATCATCAACAACAACAGCAGCTTCAGCATCAGCAATATCAGCAACAATTTCAACAACCGCAGCAACAACT
TTATCCAGGCGAACAAATAGTTCACCCTGCTGCAGCCCAAACTGGACAAAATACTACAAATGTAACAGCAGTGTCGAGCTCCAACATTACCCAATCTGCTACCTCATCACTCC
ATTCACAGCAATTGCAGCATGTAGATGTTTCCAAATCGGCTGCTGAAGAAGCCATCAGAAGATCGTTGTTGCCTGAACGTTCTACTGTTTCATAAGGCGCGCCATTATAAGTA
AATGCATGTATACTAAACTCACAAATTAGAGCTTCAATTTAATTATATCAGTTATTACCCGGGAATCTCGGTCGTAATGATTTCTATAATGACGAAAAAAAAAAAATTGGAAAGAA
AAAGCTTCATGGCCTTTATAAAAAGGAACTATCCAATACCTCGCCAGAACCAAGTAACAGTATTTTACGGGGCACAAATCAAGAACAATAAGACAGGACTGTAAAGATGGACG
CATAGATCCTGGAGGATGAGGAGACAGAAGTTAGTAGTAACAATTTGGGGACAGAAGAGGATACAGTGACATCAGAGGAAGAAGACAGCGAAGGTATAGTCAAAAAGCAAT
AGGACAGCAAGACAATTTATTTATTTAATAGTTTTTTATAAATGTAAAATCTATCACAACATGTGTACTTAATTTTCTGTGGTAGGGGAGGCCATTTCGTTGAAACGTGTCGCGA
GCGGTTGTGAGAATTGACTCATTTTGATTATTACGCAACCATTGAAATTTTTTATTTTTTTTTGGTGAAGATTTTTCCCACACAACTTCTTCTTTTACTTAACCACCAACTACCGA
TAATGGATTTAGTCAATCATTTACCAGACCGTTTGTTATTTGCTGTTCCTAAAAAGGGCAGATTATACGAAAAATGCTGTAACTTATTGAGTGGTGCCGATATACAGTTTAGAA
GATCTAATAGATTAGATATAGCACTTTCTACAAACTTGCCAATTGCATTAATCTTCTTGCCTGCAGCTGATATCCCAGTTTTCGTTGGAGAAGGTAATTGTGACTTGGGTATAA
CTGGGTTAGACCAAATCAAAGAAGCTGAACAATTCGACAACATCGAGGACTTGTTGGATTTGAAATTTGGTTCATGCAAATTGCAGATCCAAGTTCCAGCAGATGGCGAGTAC
GAAAAGCCAGAACAGCTTGTTGGAAAGAAAATTGTGTCTTCATTTACAAAATTGAGTACCGACTATTTCAAACAATTGTCAGACAAACCTACTAATATCAGATATGTCGGTGGT
TCCGTTGAGGCTTCTTGTGCCTTGGGTGTTGCTGATGCTATTGTCGATTTGGTTGAAAGTGGTGAAACTATGAAAGCAGCTGGGTTAAAGGCCATAGAAACCATATTGGAGA
CTTCGGCTCATTTGATTTCGTCAAAAAAAAAGTAAATTCCCAGAAATGGTCAATATAATCGTCCAAAGACTTCAAGGTGTTTTAGCTGCCCAGGAATATGTCTTGTGTAACTACA
ATGCCCCAAAATCCATCCAAGCAAAATGTTTAACCATTACTCCAGGCAGAAGGGCCGCTACTGTCTCTACTTTGGACAAACACAGCGACGACGAAGAGGACTGGGTTGCCAT
CTCATCCATGGTTAATAGAAAGGAAATCGGTAATGTAATGGACGAATTGAAGAAAGCTGGTGCAACCGATATATTGGTACTCGAGATATCAAATTGTAGAGTTTAAAAGAAGT
GATAGTTTCTCATAAATATTATACATATATACTTATAGATCTACTTCAATTTGACTCAATCTGTCTTATACCATCTTTATCTACCACCTTGATGTACACACCTTTAAAGTCAATAGG
CATTCTCGTTTAAACGAGCTCAACATGTTGACTCTTCCTTGTCTGCTTTGTTTTTCGGTATTTTTTTTTTTATTTCTTTGCCTTTATGTATCTATATTAAATGTACAAAATATATTACA
TGATATTCCAAATATCTTGCTTTCTTAAAAGTCTTTGAAAACTCAAACTAGCTATGGCCCTGTTGTATTCAATAAAAATTACAATGCATGATAGTAAACACACTAACAATACCCTT
GACATGAGTCAAAATCAACCGCGGATCTGCCGGTCTCCCTATAGTGAGTCGTATTAATTTCGATAAGCCAGGTTAACCTGCATTAATGAATCGGCCAACGCGCGGGGAGAG
GCGGTTTGCGTATTGGGCGCTCTTCCGCTTCCTCGCTCACTGACTCGCTGCGCTCGGTCGTTCGGCTGCGGCGAGCGGTATCAGCTCACTCAAAGGCGGTAATACGGTTAT
CCACAGAATCAGGGGATAACGCAGGAAAGAACATGTGAGCAAAAGGCCAGCAAAAGGCCAGGAACCGTAAAAAGGCCGCGTTGCTGGCGTTTTTCCATAGGCTCCGCCCC
CCTGACGAGCATCACAAAAATCGACGCTCAAGTCAGAGGTGGCGAAACCCGACAGGACTATAAAGATACCAGGCGTTTCCCCCTGGAAGCTCCCTCGTGCGCTCTCCTGTT
CCGACCCTGCCGCTTACCGGATACCTGTCCGCCTTTCTCCCTTCGGGAAGCGTGGCGCTTTCTCATAGCTCACGCTGTAGGTATCTCAGTTCGGTGTAGGTCGTTCGCTCC
AAGCTGGGCTGTGTGCACGAACCCCCCGTTCAGCCCGACCGCTGCGCCTTATCCGGTAACTATCGTCTTGAGTCCAACCCGGTAAGACACGACTTATCGCCACTGGCAGCA
GCCACTGGTAACAGGATTAGCAGAGCGAGGTATGTAGGCGGTGCTACAGAGTTCTTGAAGTGGTGGCCTAACTACGGCTACACTAGAAGAACAGTATTTGGTATCTGCGCT
CTGCTGAAGCCAGTTACCTTCGGAAAAAGAGTTGGTAGCTCTTGATCCGGCAAACAAACCACCGCTGGTAGCGGTGGTTTTTTTGTTTGCAAGCAGCAGATTACGCGCAGAA
AAAAAGGATCTCAA

*TPK2* promoter; Stop codon; GFP+GGGGlinker; *TPK2N* coding sequence; *HIS1* marker; *TPK2* 3’ HR

>pLC1719_pFA-p2-GFP-Qless2N-HIS

GAAGATCCTTTGATCTTTTCTACGGGGTCTGACGCTCAGTGGAACGAAAACTCACGTTAAGGGATTTTGGTCATGAGATTATCAAAAAGGATCTTCACCTAGATCCTTTTAAAT
TAAAAATGAAGTTTTAAATCAATCTAAAGTATATATGAGTAAACTTGGTCTGACAGTTACCAATGCTTAATCAGTGAGGCACCTATCTCAGCGATCTGTCTATTTCGTTCATCCA
TAGTTGCCTGACTCCCCGTCGTGTAGATAACTACGATACGGGAGGGCTTACCATCTGGCCCCAGTGCTGCAATGATACCGCGAGACCCACGCTCACCGGCTCCAGATTTAT
CAGCAATAAACCAGCCAGCCGGAAGGGCCGAGCGCAGAAGTGGTCCTGCAACTTTATCCGCCTCCATCCAGTCTATTAATTGTTGCCGGGAAGCTAGAGTAAGTAGTTCGC
CAGTTAATAGTTTGCGCAACGTTGTTGCCATTGCTACAGGCATCGTGGTGTCACGCTCGTCGTTTGGTATGGCTTCATTCAGCTCCGGTTCCCAACGATCAAGGCGAGTTAC
ATGATCCCCCATGTTGTGCAAAAAAGCGGTTAGCTCCTTCGGTCCTCCGATCGTTGTCAGAAGTAAGTTGGCCGCAGTGTTATCACTCATGGTTATGGCAGCACTGCATAATT
CTCTTACTGTCATGCCATCCGTAAGATGCTTTTCTGTGACTGGTGAGTACTCAACCAAGTCATTCTGAGAATAGTGTATGCGGCGACCGAGTTGCTCTTGCCCGGCGTCAATA
CGGGATAATACCGCGCCACATAGCAGAACTTTAAAAGTGCTCATCATTGGAAAACGTTCTTCGGGGCGAAAACTCTCAAGGATCTTACCGCTGTTGAGATCCAGTTCGATGT
AACCCACTCGTGCACCCAACTGATCTTCAGCATCTTTTACTTTCACCAGCGTTTCTGGGTGAGCAAAAACAGGAAGGCAAAATGCCGCAAAAAAGGGAATAAGGGCGACACG
GAAATGTTGAATACTCATACTCTTCCTTTTTCAATATTATTGAAGCATTTATCAGGGTTATTGTCTCATGAGCGGATACATATTTGAATGTATTTAGAAAAATAAACAAATAGGG
GTTCCGCGCACATTTCCCCGAAAAGTGCCACCTGACGTCTAAGAAACCATTATTATCATGACATTAACCTATAAAAATAGGCGTATCACGAGGCCCTTTCGTCTCGCGCGTTT
CGGTGATGACGGTGAAAACCTCTGACACATGCAGCTCCCGGAGACGGTCACAGCTTGTCTGTAAGCGGATGCCGGGAGCAGACAAGCCCGTCAGGGCGCGTCAGCGGGT
GTTGGCGGGTGTCGGGGCTGGCTTAACTATGCGGCATCAGAGCAGATTGTACTGAGAGTGCACCATATGGACATATTGTCGTTAGAACGCGGCTACAATTAATACATAACCT
TATGTATCATACACATACGATTTAGGTGACACTATAGAACGCGGCCGCCAGCTGAAGCTTCGTACGCTGCAGGTCGACGGATCCACATTGTTAAAAGTCGATACTTCAGTTG
CTTTTTTTTATTATTTTCTATCCAGCAAAAAACACATTGTCATTGTTTGTGTATCATTTTTTGTCCAGATAACAAACACCAAATTGAGGTCAACTAAACAACAACTTGTATAGTTCT
ACCACACTATTTTGAAGAGTCAGCTACAGTTCCTAGTTTCTCAACATAATATATCAACCTATCAATACTCGGATTGCAGGTCAATTTAGTTTACAACCACCAATTGCCAAAGTAT
TGGATTCTACTTTTTCCGACTTGTAAGTGAACACAAAGAAGATTAGACAAACAATCACCCACTCACACCTACTACTCACCCAATTTCCATTCCACCTTTTTTTTTCGAAAAAAAT
AACCTTCCCCCTTTTTGATGGTGAATCTTTTAAAGAAACTTCACATCACCAAGCTGCATCAACTGAATCAATCCAATTCGGACAGTAATTCCTTAAACTCAAACACATCAATGTC
TAAAGGTGAAGAATTATTCACTGGTGTTGTCCCAATTTTGGTTGAATTAGATGGTGATGTTAATGGTCACAAATTTTCTGTCTCCGGTGAAGGTGAAGGTGATGCTACTTACGG
TAAATTGACCTTAAAATTTATTTGTACTACTGGTAAATTGCCAGTTCCATGGCCAACCTTAGTCACTACTTTCGGTTATGGTGTTCAATGTTTTGCGAGATACCCAGATCATATG
AAACAACATGACTTTTTCAAGTCTGCCATGCCAGAAGGTTATGTTCAAGAAAGAACTATTTTTTTCAAAGATGACGGTAACTACAAGACCAGAGCTGAAGTCAAGTTTGAAGGT
GATACCTTAGTTAATAGAATCGAATTAAAAGGTATTGATTTTAAAGAAGATGGTAACATTTTAGGTCACAAATTGGAATACAACTATAACTCTCACAATGTTTACATCATGGCTG
ACAAACAAAAGAATGGTATCAAAGTTAACTTCAAAATTAGACACAACATTGAAGATGGTTCTGTTCAATTAGCTGACCATTATCAACAAAATACTCCAATTGGTGATGGTCCAG
TCTTGTTACCAGACAACCATTACTTATCCACTCAATCTGCCTTATCCAAAGATCCAAACGAAAAGAGAGACCACATGGTCTTGTTAGAATTTGTTACTGCTGCTGGTATTACCC
ATGGTATGGATGAATTGTACAAAGGTGGTGGTGGTCAACTTTATCCAGGCGAACAAATAGTTCACCCTGCTGCAGCCCAAACTGGACAAAATACTACAAATGTAACAGCAGT
GTCGAGCTCCAACATTACCCAATCTGCTACCTCATCACTCCATTCACAGCAATTGCAGCATGTAGATGTTTCCAAATCGGCTGCTGAAGAAGCCATCAGAAGATCGTTGTTGC
CTGAACGTTCTACTGTTTCATAAGGCGCGCCATTATAAGTAAATGCATGTATACTAAACTCACAAATTAGAGCTTCAATTTAATTATATCAGTTATTACCCGGGAATCTCGGTC
GTAATGATTTCTATAATGACGAAAAAAAAAAAAATTGGAAAGAAAAAGCTTCATGGCCTTTATAAAAAGGAACTATCCAATACCTCGCCAGAACCAAGTAACAGTATTTTACGGG
GCACAAATCAAGAACAATAAGACAGGACTGTAAAGATGGACGCATAGATCCTGGAGGATGAGGAGACAGAAGTTAGTAGTAACAATTTGGGGACAGAAGAGGATACAGTGA
CATCAGAGGAAGAAGACAGCGAAGGTATAGTCAAAAAGCAATAGGACAGCAAGACAATTTATTTATTTAATAGTTTTTTATAAATGTAAAATCTATCACAACATGTGTACTTAAT
TTTCTGTGGTAGGGGAGGCCATTTCGTTGAAACGTGTCGCGAGCGGTTGTGAGAATTGACTCATTTTGATTATTACGCAACCATTGAAATTTTTTATTTTTTTTTGGTGAAGAT
TTTTCCCACACAACTTCTTCTTTTACTTAACCACCAACTACCGATAATGGATTTAGTCAATCATTTACCAGACCGTTTGTTATTTGCTGTTCCTAAAAAGGGCAGATTATACGAA
AAATGCTGTAACTTATTGAGTGGTGCCGATATACAGTTTAGAAGATCTAATAGATTAGATATAGCACTTTCTACAAACTTGCCAATTGCATTAATCTTCTTGCCTGCAGCTGATA
TCCCAGTTTTCGTTGGAGAAGGTAATTGTGACTTGGGTATAACTGGGTTAGACCAAATCAAAGAAGCTGAACAATTCGACAACATCGAGGACTTGTTGGATTTGAAATTTGGT
TCATGCAAATTGCAGATCCAAGTTCCAGCAGATGGCGAGTACGAAAAGCCAGAACAGCTTGTTGGAAAGAAAATTGTGTCTTCATTTACAAAATTGAGTACCGACTATTTCAA
ACAATTGTCAGACAAACCTACTAATATCAGATATGTCGGTGGTTCCGTTGAGGCTTCTTGTGCCTTGGGTGTTGCTGATGCTATTGTCGATTTGGTTGAAAGTGGTGAAACTA
TGAAAGCAGCTGGGTTAAAGGCCATAGAAACCATATTGGAGACTTCGGCTCATTTGATTTCGTCAAAAAAAAGTAAATTCCCAGAAATGGTCAATATAATCGTCCAAAGACTT
CAAGGTGTTTTAGCTGCCCAGGAATATGTCTTGTGTAACTACAATGCCCCAAAATCCATCCAAGCAAAATGTTTAACCATTACTCCAGGCAGAAGGGCCGCTACTGTCTCTAC
TTTGGACAAACACAGCGACGACGAAGAGGACTGGGTTGCCATCTCATCCATGGTTAATAGAAAGGAAATCGGTAATGTAATGGACGAATTGAAGAAAGCTGGTGCAACCGAT
ATATTGGTACTCGAGATATCAAATTGTAGAGTTTAAAAGAAGTGATAGTTTCTCATAAATATTATACATATATACTTATAGATCTACTTCAATTTGACTCAATCTGTCTTATACCAT
CTTTATCTACCACCTTGATGTACACACCTTTAAAGTCAATAGGCATTCTCGTTTAAACGAGCTCAACATGTTGACTCTTCCTTGTCTGCTTTGTTTTTCGGTATTTTTTTTTATT
TCTTTGCCTTTATGTATCTATATTAAATGTACAAAATATATTACATGATATTCCAAATATCTTGCTTTCTTAAAAGTCTTTGAAAACTCAAACTAGCTATGGCCCTGTTGTATTCAA
TAAAAATTACAATGCATGATAGTAAACACACTAACAATACCCTTGACATGAGTCAAAATCAACCGCGGATCTGCCGGTCTCCCTATAGTGAGTCGTATTAATTTCGATAAGCCA
GGTTAACCTGCATTAATGAATCGGCCAACGCGCGGGGAGAGGCGGTTTGCGTATTGGGCGCTCTTCCGCTTCCTCGCTCACTGACTCGCTGCGCTCGGTCGTTCGGCTGC
GGCGAGCGGTATCAGCTCACTCAAAGGCGGTAATACGGTTATCCACAGAATCAGGGGATAACGCAGGAAAGAACATGTGAGCAAAAGGCCAGCAAAAGGCCAGGAACCGT
AAAAAGGCCGCGTTGCTGGCGTTTTTCCATAGGCTCCGCCCCCCTGACGAGCATCACAAAAATCGACGCTCAAGTCAGAGGTGGCGAAACCCGACAGGACTATAAAGATAC
CAGGCGTTTCCCCCTGGAAGCTCCCTCGTGCGCTCTCCTGTTCCGACCCTGCCGCTTACCGGATACCTGTCCGCCTTTCTCCCTTCGGGAAGCGTGGCGCTTTCTCATAGC
TCACGCTGTAGGTATCTCAGTTCGGTGTAGGTCGTTCGCTCCAAGCTGGGCTGTGTGCACGAACCCCCCGTTCAGCCCGACCGCTGCGCCTTATCCGGTAACTATCGTCTT
GAGTCCAACCCGGTAAGACACGACTTATCGCCACTGGCAGCAGCCACTGGTAACAGGATTAGCAGAGCGAGGTATGTAGGCGGTGCTACAGAGTTCTTGAAGTGGTGGCC
TAACTACGGCTACACTAGAAGAACAGTATTTGGTATCTGCGCTCTGCTGAAGCCAGTTACCTTCGGAAAAAGAGTTGGTAGCTCTTGATCCGGCAAACAAACCACCGCTGGT
AGCGGTGGTTTTTTTGTTTGCAAGCAGCAGATTACGCGCAGAAAAAAAGGATCTCAA

*TPK2* promoter; GFP+GGGGlinker; *TPK2N*$^{\Delta Q}$ coding sequence; *HIS1* marker; *TPK2* 3’ HR

>pLC1699_pFA-mNG-TPK1-HIS

GAAGATCCTTTGATCTTTTCTACGGGGTCTGACGCTCAGTGGAACGAAAACTCACGTTAAGGGATTTTGGTCATGAGATTATCAAAAAGGATCTTCACCTAGATCCTTTTAAAT
TAAAAATGAAGTTTTAAATCAATCTAAAGTATATATGAGTAAACTTGGTCTGACAGTTACCAATGCTTAATCAGTGAGGCACCTATCTCAGCGATCTGTCTATTTCGTTCATCCA
TAGTTGCCTGACTCCCCGTCGTGTAGATAACTACGATACGGGAGGGCTTACCATCTGGCCCCAGTGCTGCAATGATACCGCGAGACCCACGCTCACCGGCTCCAGATTTAT
CAGCAATAAACCAGCCAGCCGGAAGGGCCGAGCGCAGAAGTGGTCCTGCAACTTTATCCGCCTCCATCCAGTCTATTAATTGTTGCCGGGAAGCTAGAGTAAGTAGTTCGC
CAGTTAATAGTTTGCGCAACGTTGTTGCCATTGCTACAGGCATCGTGGTGTCACGCTCGTCGTTTGGTATGGCTTCATTCAGCTCCGGTTCCCAACGATCAAGGCGAGTTAC
ATGATCCCCCATGTTGTGCAAAAAAGCGGTTAGCTCCTTCGGTCCTCCGATCGTTGTCAGAAGTAAGTTGGCCGCAGTGTTATCACTCATGGTTATGGCAGCACTGCATAATT
CTCTTACTGTCATGCCATCCGTAAGATGCTTTTCTGTGACTGGTGAGTACTCAACCAAGTCATTCTGAGAATAGTGTATGCGGCGACCGAGTTGCTCTTGCCCGGCGTCAATA
CGGGATAATACCGCGCCACATAGCAGAACTTTAAAAGTGCTCATCATTGGAAAACGTTCTTCGGGGCGAAAACTCTCAAGGATCTTACCGCTGTTGAGATCCAGTTCGATGT
AACCCACTCGTGCACCCAACTGATCTTCAGCATCTTTTACTTTCACCAGCGTTTCTGGGTGAGCAAAAACAGGAAGGCAAAATGCCGCAAAAAAGGGAATAAGGGCGACACG
GAAATGTTGAATACTCATACTCTTCCTTTTTCAATATTATTGAAGCATTTATCAGGGTTATTGTCTCATGAGCGGATACATATTTGAATGTATTTAGAAAAATAAACAAATAGGG
GTTCCGCGCACATTTCCCCGAAAAGTGCCACCTGACGTCTAAGAAACCATTATTATCATGACATTAACCTATAAAAATAGGCGTATCACGAGGCCCTTTCGTCTCGCGCGTTT
CGGTGATGACGGTGAAAACCTCTGACACATGCAGCTCCCGGAGACGGTCACAGCTTGTCTGTAAGCGGATGCCGGGAGCAGACAAGCCCGTCAGGGCGCGTCAGCGGGT
GTTGGCGGGTGTCGGGGCTGGCTTAACTATGCGGCATCAGAGCAGATTGTACTGAGAGTGCACCATATGGACATATTGTCGTTAGAACGCGGCTACAATTAATACATAACCT
TATGTATCATACACATACGATTTAGGTGACACTATAGAACGCGGCCGCCAGCTGAAGCTTCGTACGCTGCAGGTCGACGGATCCAGACAGAAGATAATATTTTTTCTTAACTG
TCACGCAAAAGTTTTAACTAACTAACTAACTAACTAATTAGACCAATTCCCCTAAAGGACTTAAGGACTAAAATAAGCTTATTTCAATTAATATTATCATTGAATAATAGATACTT
ATAGCAGTTATAGTAGAATTTAATTTCTTTTTTTTTTTCTTTCTTTCTTTTGTCACACATTTATCCGTTTTGACAGACTAAATTAAACAAAAAATTTTTGAACATCAGGGAACGACC
ATATACAAATAAAAGCAGAAGAAAAAAACGGAAGAAGAAGAAGAAGAAAAAAAAGTCAACCAAAAAATTGAACAAGAGGTCTAAAGAACCACAACATTATACAAAAGAAAAGA
AATTAAATTAAATTACATTAAAAGAGAATTGAAACATAACACTTGAAGAAGAAAATAAAAGTATTTTTATATTATATATTCCGACTAGTATTATCTGAGATCAATATATCCTATCGT
TATCCTCCTCCTTCTCCTTTCAACTTTTGAAAAAGGTGATATTATTCAACACTGTTTTTGTTTTATCAACCAAAACCAGGATGGTTTCTAAAGGTGAAGAAGATAATATGGCTAG
TTTGCCTGCAACACATGAATTACATATTTTTGGTAGTATTAATGGTGTTGATTTTGATATGGTTGGTCAAGGTACTGGTAATCCAAATGATGGTTATGAAGAATTGAATTTGAAA
TCAACTAAAGGAGATTTGCAATTTTCTCCATGGATTTTGGTTCCACATATTGGTTATGGTTTCCATCAATATTTGCCATATCCAGATGGAATGTCACCATTTCAAGCTGCTATGG
TTGATGGTTCTGGTTATCAAGTTCATAGAACTATGCAATTCGAAGATGGTGCTTCATTGACTGTTAATTATAGATATACTTATGAAGGTTCTCATATTAAAGGTGAAGCTCAAGT
TAAAGGTACTGGTTTTCCAGCTGATGGTCCAGTTATGACTAATTCATTAACTGCTGCTGATTGGTGTAGATCAAAGAAAACTTATCCAAATGATAAAACTATTATTTCAACTTTT
AAATGGTCTTATACTACTGGTAATGGTAAAAGATATAGATCAACTGCTAGAACTACTTATACTTTCGCTAAACCAATGGCTGCTAATTATTTGAAAAATCAACCAATGTATGTTT
TTAGAAAAACTGAATTAAAACATTCTAAAACTGAATTGAATTTCAAAGAATGGCAAAAAGCATTCACTGATGTTATGGGTATGGATGAATTGTATAAAGGTGGTGGTGGTACAT
CCATGGAACCAGCAGACACAAGCATCAGGTCATTAAACGACATCAACTTACAAGAACTTGCCAACAAACAACATCAACGAAATATATATGCTCAAGGATCACCTACACTAGAA
GATTCAATAACATCAAATGGTAATGATATTAATAATCCCAACAATAATATAAATAAAACCAATAATAACAGTAATTCAAGTCTTTCCTTGACCAAGAAAACATCAAGACATATCAA
TAAAGACACTACAACTAAAGGCAAATATACATTAAATGATTTTCAAATATTACGAACATTAGGAACAGGTTCCTTTGGTAGAGTTCATTTAACTCGATCGATTCATAATGGTAGA
TTTTATGCTATGAAAGTATTAAAGAAACAACGAGTAGTACAAATGAAACAAATCGAACATACTAATGATGAAAGAAGAATGTTAAAATTAGCTCAACATCCATTTATAATTCGAA
TGTGGGGGACATTCCAAGATTGTCATAATTTATTTATGATTATGGATTATATAGAAGGAGGTGAATTATTTTCATTATTAAGAAAATCCCAACGATTCCCTACTCCAGTAGCTAA
ATTTTATGCTGCCGAAGTATTTTTGGCTATTGAATATTTACACAGTTTAGACATTATTTATCGAGATTTAAAACCAGAAAATATATTATTGGATAAAAATGGTCATATCAAATTAA
CTGATTTTGGATTTGCTAAAGAAGTTCAAGATGTGACTTATACTTTATGTGGGACTCCAGATTATATTGCTCCTGAAGTGGTGGCAACAAAACCTTATAATAAATCAGTTGATT
GGTGGTCATTTGGTATATTAATATTTGAAATGTTAACTGGTTATACTCCATTCTATGATCCTACTCCAATGAAAACTTATGAAAACATTTTAAATGGATCAATTACTTATCCTGAT
TATTTACCTCCAGATATATTGGATCTTCTACAAAAATTAATTGTCAAAGATTTAACTCAAAGATTAGGTAATTTACAAGGTGGTTCTGATGATGTGAAAAATCATCCGTGGTTTA
AAGAAGTTATTTGGGAAAGATTGTTGTCACGTGATATAGAAACTCCATATGAACCACCTATAACTTCTGGTGTTGGTGATACTTCACAATTTGATCGATACCCAGAAGATAAAG
ATTTGGATTATGGTATAAGTGGAGTTGAAGACCCATATCGTGATCAATTCCAGGACTTTTAAATGTAAAAATAAAATTGTATTTATTGGCACAACAATGTTAATTATGATAACTA
GTTTTATAGATGTATAATTAATTTGCAAGAAAGAAGAAGACGAAGATTTGATTAGTGAATTTTTTAAAGCTAAAAAAACACTTCTTGGTGTATCAAAAGTAATTTTGGCAAATCTT
ATAGACAGCTAAAATATAAGCAGTGTCTTATTTTTAAACCAATGATCACTATATGAATTGGCTTCCGGCGCGCCATTATAAGTAAATGCATGTATACTAAACTCACAAATTAGAG
CTTCAATTTAATTATATCAGTTATTACCCGGGAATCTCGGTCGTAATGATTTCTATAATGACGAAAAAAAAAAAAATTGGAAAGAAAAAGCTTCATGGCCTTTATAAAAAGGAACT
ATCCAATACCTCGCCAGAACCAAGTAACAGTATTTTACGGGGCACAAATCAAGAACAATAAGACAGGACTGTAAAGATGGACGCATAGATCCTGGAGGATGAGGAGACAGAA
GTTAGTAGTAACAATTTGGGGACAGAAGAGGATACAGTGACATCAGAGGAAGAAGACAGCGAAGGTATAGTCAAAAAGCAATAGGACAGCAAGACAATTTATTTATTTAATAG
TTTTTTATAAATGTAAAATCTATCACAACATGTGTACTTAATTTTCTGTGGTAGGGGAGGCCATTTCGTTGAAACGTGTCGCGAGCGGTTGTGAGAATTGACTCATTTTGATTAT
TACGCAACCATTGAAATTTTTTATTTTTTTTTGGTGAAGATTTTTCCCACACAACTTCTTCTTTTACTTAACCACCAACTACCGATAATGGATTTAGTCAATCATTTACCAGACCG
TTTGTTATTTGCTGTTCCTAAAAAGGGCAGATTATACGAAAAATGCTGTAACTTATTGAGTGGTGCCGATATACAGTTTAGAAGATCTAATAGATTAGATATAGCACTTTCTACA
AACTTGCCAATTGCATTAATCTTCTTGCCTGCAGCTGATATCCCAGTTTTCGTTGGAGAAGGTAATTGTGACTTGGGTATAACTGGGTTAGACCAAATCAAAGAAGCTGAACA
ATTCGACAACATCGAGGACTTGTTGGATTTGAAATTTGGTTCATGCAAATTGCAGATCCAAGTTCCAGCAGATGGCGAGTACGAAAAGCCAGAACAGCTTGTTGGAAAGAAA
ATTGTGTCTTCATTTACAAAATTGAGTACCGACTATTTCAAACAATTGTCAGACAAACCTACTAATATCAGATATGTCGGTGGTTCCGTTGAGGCTTCTTGTGCCTTGGGTGTT
GCTGATGCTATTGTCGATTTGGTTGAAAGTGGTGAAACTATGAAAGCAGCTGGGTTAAAGGCCATAGAAACCATATTGGAGACTTCGGCTCATTTGATTTCGTCAAAAAAAAG
TAAATTCCCAGAAATGGTCAATATAATCGTCCAAAGACTTCAAGGTGTTTTAGCTGCCCAGGAATATGTCTTGTGTAACTACAATGCCCCAAAATCCATCCAAGCAAAATGTTT
AACCATTACTCCAGGCAGAAGGGCCGCTACTGTCTCTACTTTGGACAAACACAGCGACGACGAAGAGGACTGGGTTGCCATCTCATCCATGGTTAATAGAAAGGAAATCGG
TAATGTAATGGACGAATTGAAGAAAGCTGGTGCAACCGATATATTGGTACTCGAGATATCAAATTGTAGAGTTTAAAAGAAGTGATAGTTTCTCATAAATATTATACATATATAC
TTATAGATCTACTTCAATTTGACTCAATCTGTCTTATACCATCTTTATCTACCACCTTGATGTACACACCTTTAAAGTCAATAGGCATTCTCGTTTAAACGAGCTCCACAATAGTT
CAATATTGGAATCAAACATAATAGCGAGAATTGGTTTTTATCTGAGTACCACATTGAGAATGGTATTGGATGTTATTAAGTTGCCATTTTTGAATGAACTTCTCACTGATGATTC
TTTTTATTAAATCCAGGGTCCTTTTTTTAGGGTATTCTACTAAAACTTGATATTTGGGATGATTTGGTAGTTCTATTTTGATTTCCACTAATGAAAAGCATTTCTAACAAGTTTTG
GAAATTTGACTGAGATGTGCATTACTAAGAGCTTCGTTGTTACGGTGATACTAAAGTTTGTCAAGTCTCTTAAACATTACATTGAATACTATTTGATAGTTGGTGATAAAAAAAA
CTCCGTAAACAATAATTTCGTGTTTAATTCACATTTTATTGGAGCATGAAAATTATTTAACCGAACGCGTTCTTGAATGTGTATAAAAATAGAAATCCCATATTATCGAATTAGAG
GTCTCCGCGGATCTGCCGGTCTCCCTATAGTGAGTCGTATTAATTTCGATAAGCCAGGTTAACCTGCATTAATGAATCGGCCAACGCGCGGGGAGAGGCGGTTTGCGTATT
GGGCGCTCTTCCGCTTCCTCGCTCACTGACTCGCTGCGCTCGGTCGTTCGGCTGCGGCGAGCGGTATCAGCTCACTCAAAGGCGGTAATACGGTTATCCACAGAATCAGG
GGATAACGCAGGAAAGAACATGTGAGCAAAAGGCCAGCAAAAGGCCAGGAACCGTAAAAAGGCCGCGTTGCTGGCGTTTTTCCATAGGCTCCGCCCCCCTGACGAGCATC
ACAAAAATCGACGCTCAAGTCAGAGGTGGCGAAACCCGACAGGACTATAAAGATACCAGGCGTTTCCCCCTGGAAGCTCCCTCGTGCGCTCTCCTGTTCCGACCCTGCCGC
TTACCGGATACCTGTCCGCCTTTCTCCCTTCGGGAAGCGTGGCGCTTTCTCATAGCTCACGCTGTAGGTATCTCAGTTCGGTGTAGGTCGTTCGCTCCAAGCTGGGCTGTGT
GCACGAACCCCCCGTTCAGCCCGACCGCTGCGCCTTATCCGGTAACTATCGTCTTGAGTCCAACCCGGTAAGACACGACTTATCGCCACTGGCAGCAGCCACTGGTAACAG
GATTAGCAGAGCGAGGTATGTAGGCGGTGCTACAGAGTTCTTGAAGTGGTGGCCTAACTACGGCTACACTAGAAGAACAGTATTTGGTATCTGCGCTCTGCTGAAGCCAGT
TACCTTCGGAAAAAGAGTTGGTAGCTCTTGATCCGGCAAACAAACCACCGCTGGTAGCGGTGGTTTTTTTGTTTGCAAGCAGCAGATTACGCGCAGAAAAAAAGGATCTCAA

*TPK1* promoter; mNeonGreen+GGGGlinker; *TPK1* ORF; *HIS1* marker (replaced with a *SAT1* marker in pLC1708); *TPK1* 3' HR

>pLC1700_pFA-mNG-TPK2-HIS

GAAGATCCTTTGATCTTTTCTACGGGGTCTGACGCTCAGTGGAACGAAAACTCACGTTAAGGGATTTTGGTCATGAGATTATCAAAAAGGATCTTCACCTAGATCCTTTTAAAT
TAAAAATGAAGTTTTAAATCAATCTAAAGTATATATGAGTAAACTTGGTCTGACAGTTACCAATGCTTAATCAGTGAGGCACCTATCTCAGCGATCTGTCTATTTCGTTCATCCA
TAGTTGCCTGACTCCCCGTCGTGTAGATAACTACGATACGGGAGGGCTTACCATCTGGCCCCAGTGCTGCAATGATACCGCGAGACCCACGCTCACCGGCTCCAGATTTAT
CAGCAATAAACCAGCCAGCCGGAAGGGCCGAGCGCAGAAGTGGTCCTGCAACTTTATCCGCCTCCATCCAGTCTATTAATTGTTGCCGGGAAGCTAGAGTAAGTAGTTCGC
CAGTTAATAGTTTGCGCAACGTTGTTGCCATTGCTACAGGCATCGTGGTGTCACGCTCGTCGTTTGGTATGGCTTCATTCAGCTCCGGTTCCCAACGATCAAGGCGAGTTAC
ATGATCCCCCATGTTGTGCAAAAAAGCGGTTAGCTCCTTCGGTCCTCCGATCGTTGTCAGAAGTAAGTTGGCCGCAGTGTTATCACTCATGGTTATGGCAGCACTGCATAATT
CTCTTACTGTCATGCCATCCGTAAGATGCTTTTCTGTGACTGGTGAGTACTCAACCAAGTCATTCTGAGAATAGTGTATGCGGCGACCGAGTTGCTCTTGCCCGGCGTCAATA
CGGGATAATACCGCGCCACATAGCAGAACTTTAAAAGTGCTCATCATTGGAAAACGTTCTTCGGGGCGAAAACTCTCAAGGATCTTACCGCTGTTGAGATCCAGTTCGATGT
AACCCACTCGTGCACCCAACTGATCTTCAGCATCTTTTACTTTCACCAGCGTTTCTGGGTGAGCAAAAACAGGAAGGCAAAATGCCGCAAAAAAGGGAATAAGGGCGACACG
GAAATGTTGAATACTCATACTCTTCCTTTTTCAATATTATTGAAGCATTTATCAGGGTTATTGTCTCATGAGCGGATACATATTTGAATGTATTTAGAAAAATAAACAAATAGGG
GTTCCGCGCACATTTCCCCGAAAAGTGCCACCTGACGTCTAAGAAACCATTATTATCATGACATTAACCTATAAAAATAGGCGTATCACGAGGCCCTTTCGTCTCGCGCGTTT
CGGTGATGACGGTGAAAACCTCTGACACATGCAGCTCCCGGAGACGGTCACAGCTTGTCTGTAAGCGGATGCCGGGAGCAGACAAGCCCGTCAGGGCGCGTCAGCGGGT
GTTGGCGGGTGTCGGGGCTGGCTTAACTATGCGGCATCAGAGCAGATTGTACTGAGAGTGCACCATATGGACATATTGTCGTTAGAACGCGGCTACAATTAATACATAACCT
TATGTATCATACACATACGATTTAGGTGACACTATAGAACGCGGCCGCCAGCTGAAGCTTCGTACGCTGCAGGTCGACGGATCCACATTGTTAAAAGTCGATACTTCAGTTG
CTTTTTTTTATTATTTTCTATCCAGCAAAAAACACATTGTCATTGTTTGTGTATCATTTTTTGTCCAGATAACAAACACCAAATTGAGGTCAACTAAACAACAACTTGTATAGTTCT
ACCACACTATTTTGAAGAGTCAGCTACAGTTCCTAGTTTCTCAACATAATATATCAACCTATCAATACTCGGATTGCAGGTCAATTTAGTTTACAACCACCAATTGCCAAAGTAT
TGGATTCTACTTTTTCCGACTTGTAAGTGAACACAAAGAAGATTAGACAAACAATCACCCACTCACACCTACTACTCACCCAATTTCCATTCCACCTTTTTTTTTCGAAAAAAAT
AACCTTCCCCCTTTTTGATGGTGAATCTTTTAAAGAAACTTCACATCACCAAGCTGCATCAACTGAATCAATCCAATTCGGACAGTAATTCCTTAAACTCAAACACATCAATGGT
TTCTAAAGGTGAAGAAGATAATATGGCTAGTTTGCCTGCAACACATGAATTACATATTTTTGGTAGTATTAATGGTGTTGATTTTGATATGGTTGGTCAAGGTACTGGTAATCC
AAATGATGGTTATGAAGAATTGAATTTGAAATCAACTAAAGGAGATTTGCAATTTTCTCCATGGATTTTGGTTCCACATATTGGTTATGGTTTCCATCAATATTTGCCATATCCA
GATGGAATGTCACCATTTCAAGCTGCTATGGTTGATGGTTCTGGTTATCAAGTTCATAGAACTATGCAATTCGAAGATGGTGCTTCATTGACTGTTAATTATAGATATACTTATG
AAGGTTCTCATATTAAAGGTGAAGCTCAAGTTAAAGGTACTGGTTTTCCAGCTGATGGTCCAGTTATGACTAATTCATTAACTGCTGCTGATTGGTGTAGATCAAAGAAAACTT
ATCCAAATGATAAAACTATTATTTCAACTTTTAAATGGTCTTATACTACTGGTAATGGTAAAAGATATAGATCAACTGCTAGAACTACTTATACTTTCGCTAAACCAATGGCTGCT
AATTATTTGAAAAATCAACCAATGTATGTTTTTAGAAAAACTGAATTAAAACATTCTAAAACTGAATTGAATTTCAAAGAATGGCAAAAAGCATTCACTGATGTTATGGGTATGG
ATGAATTGTATAAAGGTGGTGGTGGTGACAATCATCAACAACAACAGCAGCTTCAGCATCAGCAATATCAGCAACAATTTCAACAACCGCAGCAACAACTTTATCCAGGCGAA
CAAATAGTTCACCCTGCTGCAGCCCAAACTGGACAAAATACTACAAATGTAACAGCAGTGTCGAGCTCCAACATTACCCAATCTGCTACCTCATCACTCCATTCACAGCAATT
GCAGCATGTAGATGTTTCCAAATCGGCTGCTGAAGAAGCCATCAGAAGATCGTTGTTGCCTGAACGTTCTACTGTTTCAAAGGGGAAATACTCTTTGACTGATTTCTCAATTA
TGAGAACTTTGGGAACAGGTTCCTTTGGTAGAGTACATTTAGTGAGATCAGTTCACAATGGTAGATATTATGCTATCAAAGTTTTGAAGAAGCATCAAGTTGTGAAAATGAAGC
AAGTTGAACACACAAATGATGAAAGAAGAATGTTGAAGTTGGTTGAACATCCATTTTTGATTAGAATGTGGGGGACTTTCCAAGATTCCAAAAATTTATTTATGGTTATGGACT
ATATTGAAGGGGGAGAGTTATTTTCATTGTTAAGAAAGTCTCAGAGATTTCCCAATCCAGTAGCCAAGTTTTATGCTGCAGAGGTTACCTTGGCTTTGGAGTATTTGCACAGTC
ATGATATTATCTATCGTGATTTGAAACCAGAAAATATATTATTGGATAGAAATGGTCATATAAAGATTACAGATTTTGGATTTGCCAAAGAAGTTAGTACCGTTACATGGACGTT
ATGTGGTACCCCCGATTATATTGCCCCAGAAGTCATAACAACCAAACCCTATAACAAGTCGGTGGACTGGTGGTCGTTGGGGGTATTGATTTTTGAAATGTTGGCAGGTTAC
ACTCCATTTTATGATTCGACTCCAATGAAAACTTATGAAAAAATTTTGGCAGGCAAAATACATTACCCAAGTTTTTTTCAACCTGATGTGATTGATTTGTTGACTAAATTAATAAC
TGCTGATTTGACAAGAAGATTGGGTAATTTGATCAACGGACCAGCAGATATCAGAAATCATCCCTGGTTTCTGGAAGTGGTATGGGAGAAATTGTTGGCAAAGGATATTGAAA
CTCCTTATGAACCACCAATCACAGCTGGGGTTGGTGATTCGTCATTGTTTGACCATTATCCAGAAGAACAATTAGACTACGGAAGCCAAGGAGAAGATCCTTATGCTCTGTAT
TTCCTTGACTTTTGAATTGAGAGGAGATGAACTTTCATAAATGAATAAATGAGGCGCGCCATTATAAGTAAATGCATGTATACTAAACTCACAAATTAGAGCTTCAATTTAATTA
TATCAGTTATTACCCGGGAATCTCGGTCGTAATGATTTCTATAATGACGAAAAAAAAAAAAATTGGAAAGAAAAAGCTTCATGGCCTTTATAAAAAGGAACTATCCAATACCTCG
CCAGAACCAAGTAACAGTATTTTACGGGGCACAAATCAAGAACAATAAGACAGGACTGTAAAGATGGACGCATAGATCCTGGAGGATGAGGAGACAGAAGTTAGTAGTAACA
ATTTGGGGACAGAAGAGGATACAGTGACATCAGAGGAAGAAGACAGCGAAGGTATAGTCAAAAAGCAATAGGACAGCAAGACAATTTATTTATTTAATAGTTTTTTATAAATG
TAAAATCTATCACAACATGTGTACTTAATTTTCTGTGGTAGGGGAGGCCATTTCGTTGAAACGTGTCGCGAGCGGTTGTGAGAATTGACTCATTTTGATTATTACGCAACCATT
GAAATTTTTTATTTTTTTTTGGTGAAGATTTTTCCCACACAACTTCTTCTTTTACTTAACCACCAACTACCGATAATGGATTTAGTCAATCATTTACCAGACCGTTTGTTATTTGCT
GTTCCTAAAAAGGGCAGATTATACGAAAAATGCTGTAACTTATTGAGTGGTGCCGATATACAGTTTAGAAGATCTAATAGATTAGATATAGCACTTTCTACAAACTTGCCAATT
GCATTAATCTTCTTGCCTGCAGCTGATATCCCAGTTTTCGTTGGAGAAGGTAATTGTGACTTGGGTATAACTGGGTTAGACCAAATCAAAGAAGCTGAACAATTCGACAACAT
CGAGGACTTGTTGGATTTGAAATTTGGTTCATGCAAATTGCAGATCCAAGTTCCAGCAGATGGCGAGTACGAAAAGCCAGAACAGCTTGTTGGAAAGAAAATTGTGTCTTCAT
TTACAAAATTGAGTACCGACTATTTCAAACAATTGTCAGACAAACCTACTAATATCAGATATGTCGGTGGTTCCGTTGAGGCTTCTTGTGCCTTGGGTGTTGCTGATGCTATTG
TCGATTTGGTTGAAAGTGGTGAAACTATGAAAGCAGCTGGGTTAAAGGCCATAGAAACCATATTGGAGACTTCGGCTCATTTGATTTCGTCAAAAAAAAGTAAATTCCCAGAA
ATGGTCAATATAATCGTCCAAAGACTTCAAGGTGTTTTAGCTGCCCAGGAATATGTCTTGTGTAACTACAATGCCCCAAAATCCATCCAAGCAAAATGTTTAACCATTACTCCA
GGCAGAAGGGCCGCTACTGTCTCTACTTTGGACAAACACAGCGACGACGAAGAGGACTGGGTTGCCATCTCATCCATGGTTAATAGAAAGGAAATCGGTAATGTAATGGAC
GAATTGAAGAAAGCTGGTGCAACCGATATATTGGTACTCGAGATATCAAATTGTAGAGTTTAAAAGAAGTGATAGTTTCTCATAAATATTATACATATATACTTATAGATCTACT
TCAATTTGACTCAATCTGTCTTATACCATCTTTATCTACCACCTTGATGTACACACCTTTAAAGTCAATAGGCATTCTCGTTTAAACGAGCTCAACATGTTGACTCTTCCTTGTC
TGCTTTGTTTTTCGGTATTTTTTTTTTATTTCTTTGCCTTTATGTATCTATATTAAATGTACAAAATATATTACATGATATTCCAAATATCTTGCTTTCTTAAAAGTCTTTGAAAACT
CAAACTAGCTATGGCCCTGTTGTATTCAATAAAAATTACAATGCATGATAGTAAACACACTAACAATACCCTTGACATGAGTCAAAATCAACCGCGGATCTGCCGGTCTCCCTA
TAGTGAGTCGTATTAATTTCGATAAGCCAGGTTAACCTGCATTAATGAATCGGCCAACGCGCGGGGAGAGGCGGTTTGCGTATTGGGCGCTCTTCCGCTTCCTCGCTCACTG
ACTCGCTGCGCTCGGTCGTTCGGCTGCGGCGAGCGGTATCAGCTCACTCAAAGGCGGTAATACGGTTATCCACAGAATCAGGGGATAACGCAGGAAAGAACATGTGAGCA
AAAGGCCAGCAAAAGGCCAGGAACCGTAAAAAGGCCGCGTTGCTGGCGTTTTTCCATAGGCTCCGCCCCCCTGACGAGCATCACAAAAATCGACGCTCAAGTCAGAGGTG
GCGAAACCCGACAGGACTATAAAGATACCAGGCGTTTCCCCCTGGAAGCTCCCTCGTGCGCTCTCCTGTTCCGACCCTGCCGCTTACCGGATACCTGTCCGCCTTTCTCCC
TTCGGGAAGCGTGGCGCTTTCTCATAGCTCACGCTGTAGGTATCTCAGTTCGGTGTAGGTCGTTCGCTCCAAGCTGGGCTGTGTGCACGAACCCCCCGTTCAGCCCGACCG
CTGCGCCTTATCCGGTAACTATCGTCTTGAGTCCAACCCGGTAAGACACGACTTATCGCCACTGGCAGCAGCCACTGGTAACAGGATTAGCAGAGCGAGGTATGTAGGCGG
TGCTACAGAGTTCTTGAAGTGGTGGCCTAACTACGGCTACACTAGAAGAACAGTATTTGGTATCTGCGCTCTGCTGAAGCCAGTTACCTTCGGAAAAAGAGTTGGTAGCTCT
TGATCCGGCAAACAAACCACCGCTGGTAGCGGTGGTTTTTTTGTTTGCAAGCAGCAGATTACGCGCAGAAAAAAAGGATCTCAA

*TPK2* promoter; mNeonGreen+GGGGlinker; *TPK2* ORF; *HIS1* marker (replaced with a *SAT1* marker in pLC1709); *TPK2* 3’ HR

>pLC1214_pFA-GFP-HYGB

GAACGCGGCCGCCAGCTGAAGCTTCGTACGCTGCAGGTCGACGGATCCCCggtggtggtTCTAAAGGTGAAGAATTATTCACTGGTGTTGTCCCAATTTTGGTTGAATTAGATGG
TGATGTTAATGGTCACAAATTTTCTGTCTCCGGTGAAGGTGAAGGTGATGCTACTTACGGTAAATTGACCTTAAAATTTATTTGTACTACTGGTAAATTGCCAGTTCCATGGCC
AACCTTAGTCACTACTTTCGGTTATGGTGTTCAATGTTTTGCGAGATACCCAGATCATATGAAACAACATGACTTTTTCAAGTCTGCCATGCCAGAAGGTTATGTTCAAGAAAG
AACTATTTTTTTCAAAGATGACGGTAACTACAAGACCAGAGCTGAAGTCAAGTTTGAAGGTGATACCTTAGTTAATAGAATCGAATTAAAAGGTATTGATTTTAAAGAAGATGG
TAACATTTTAGGTCACAAATTGGAATACAACTATAACTCTCACAATGTTTACATCATGGCTGACAAACAAAAGAATGGTATCAAAGTTAACTTCAAAATTAGACACAACATTGAA
GATGGTTCTGTTCAATTAGCTGACCATTATCAACAAAATACTCCAATTGGTGATGGTCCAGTCTTGTTACCAGACAACCATTACTTATCCACTCAATCTGCCTTATCCAAAGAT
CCAAACGAAAAGAGAGACCACATGGTCTTGTTAGAATTTGTTACTGCTGCTGGTATTACCCATGGTATGGATGAATTGTACAAAtaaGGCGCGCCcttgctgttcgatattgctagaggcaaa
gaaaaaggattacctcacgcctaaaatggaatttgaaaattgaggagatgagttgagcttttgtcttttacactttccctattcaattgtgccgcaaaaacttcaatatctaacacagtgtgaaatatttgttatgtataccgaaagccaaataccatttaaactggtat
ttcaagatttcataaatcaagtttctggttaaattaattgctgttaaacagaacaagagcttcacattgatgttggaataactactatagaattataaaatacacaaaaccaggtcgtgtacgcaataacttttcaatgtatgaaaaaatttttttttttttgcttcttctctt
cgcttctttttctagttatcaatttggcaatgaattgatttgattgccctttgtttagtttactatgaaaaaaaattttttttgtctctgtcagatttctctcatttttttttgcctcataatgaacaactcaaaaaagaatataaatttctcatcaaagacctttccaattgaaaat
tttttctttccttcttttcctttttttttctttctttcttcttttctaagtatacattttatataatcaatcctcgagatgaaaaaaccagaattgactgctacttctgttgaaaaatttttgattgaaaaatttgattctgtttctgatttgatgcaattgtctgaaggtgaagaatcaagag
ctttttcttttgatgttggtggtagaggttatgttttgagagttaattcttgtgctgatggtttttataaagatagatatgtttatagacattttgcttctgctgctttgccaattccagaagttttggatattggtgaattttctgaatctttgacttattgtatttcaagaagagctcaag
gtgttactttgcaagatttgccagaaactgaattgccagctgttttgcaaccagttgctgaagctatggatgctattgctgctgctgatttgtctcaaacttctggttttggtccatttggtccacaaggtattggtcaatatactacttggagagattttattgtgctattgct
gatccacatgtttatcattggcaaactgttatggatgatactgtttctgcttctgttgctcaagcattggatgaattgatgttgtgggctgaagattgtccagaagttagacatttggttcatgctgattttggttctaataatgttttgactgataatggtagaattactgctgtt
attgattggtctgaagctatgtttggtgattctcaatatgaagttgctaatattttttttggagaccatggttggcttgtatggaacaacaaactagatatttgaaagaagacatccagaattggctggttctccaagattgagagcttatatgttgagaattggtttggat
caattgtatcaatctttggttgatggtaattttgatgatgctgcttgggctcaaggtagatgtgatgctattgttagatcaggtgctggtactgttggtagaactcaaattgctagaagatcagctgctgtttggactgatggttgtgttgaagttttggctgattctggtaata
gaagaccatctactagaccaagagctaaagaataaggatcGTTTAAACGAGCTCGAATTCATCGATGATATCAGATCCACTAGTGGCCTATGCGGCCGCGGATCTGCCGGTCTCCCTATAGTG
AGTCGTATTAATTTCGATAAGCCAGGTTAACCTGCATTAATGAATCGGCCAACGCGCGGGGAGAGGCGGTTTGCGTATTGGGCGCTCTTCCGCTTCCTCGCTCACTGACTC
GCTGCGCTCGGTCGTTCGGCTGCGGCGAGCGGTATCAGCTCACTCAAAGGCGGTAATACGGTTATCCACAGAATCAGGGGATAACGCAGGAAAGAACATGTGAGCAAAAG
GCCAGCAAAAGGCCAGGAACCGTAAAAAGGCCGCGTTGCTGGCGTTTTTCCATAGGCTCCGCCCCCCTGACGAGCATCACAAAAATCGACGCTCAAGTCAGAGGTGGCGA
AACCCGACAGGACTATAAAGATACCAGGCGTTTCCCCCTGGAAGCTCCCTCGTGCGCTCTCCTGTTCCGACCCTGCCGCTTACCGGATACCTGTCCGCCTTTCTCCCTTCG
GGAAGCGTGGCGCTTTCTCAATGCTCACGCTGTAGGTATCTCAGTTCGGTGTAGGTCGTTCGCTCCAAGCTGGGCTGTGTGCACGAACCCCCCGTTCAGCCCGACCGCTG
CGCCTTATCCGGTAACTATCGTCTTGAGTCCAACCCGGTAAGACACGACTTATCGCCACTGGCAGCAGCCACTGGTAACAGGATTAGCAGAGCGAGGTATGTAGGCGGTGC
TACAGAGTTCTTGAAGTGGTGGCCTAACTACGGCTACACTAGAAGGACAGTATTTGGTATCTGCGCTCTGCTGAAGCCAGTTACCTTCGGAAAAAGAGTTGGTAGCTCTTGA
TCCGGCAAACAAACCACCGCTGGTAGCGGTGGTTTTTTTGTTTGCAAGCAGCAGATTACGCGCAGAAAAAAAGGATCTCAAGAAGATCCTTTGATCTTTTCTACGGGGTCTG
ACGCTCAGTGGAACGAAAACTCACGTTAAGGGATTTTGGTCATGAGATTATCAAAAAGGATCTTCACCTAGATCCTTTTAAATTAAAAATGAAGTTTTAAATCAATCTAAAGTAT
ATATGAGTAAACTTGGTCTGACAGTTACCAATGCTTAATCAGTGAGGCACCTATCTCAGCGATCTGTCTATTTCGTTCATCCATAGTTGCCTGACTCCCCGTCGTGTAGATAAC
TACGATACGGGAGGGCTTACCATCTGGCCCCAGTGCTGCAATGATACCGCGAGACCCACGCTCACCGGCTCCAGATTTATCAGCAATAAACCAGCCAGCCGGAAGGGCCG
AGCGCAGAAGTGGTCCTGCAACTTTATCCGCCTCCATCCAGTCTATTAATTGTTGCCGGGAAGCTAGAGTAAGTAGTTCGCCAGTTAATAGTTTGCGCAACGTTGTTGCCATT
GCTACAGGCATCGTGGTGTCACGCTCGTCGTTTGGTATGGCTTCATTCAGCTCCGGTTCCCAACGATCAAGGCGAGTTACATGATCCCCCATGTTGTGCAAAAAAGCGGTTA
GCTCCTTCGGTCCTCCGATCGTTGTCAGAAGTAAGTTGGCCGCAGTGTTATCACTCATGGTTATGGCAGCACTGCATAATTCTCTTACTGTCATGCCATCCGTAAGATGCTTT
TCTGTGACTGGTGAGTACTCAACCAAGTCATTCTGAGAATAGTGTATGCGGCGACCGAGTTGCTCTTGCCCGGCGTCAATACGGGATAATACCGCGCCACATAGCAGAACTT
TAAAAGTGCTCATCATTGGAAAACGTTCTTCGGGGCGAAAACTCTCAAGGATCTTACCGCTGTTGAGATCCAGTTCGATGTAACCCACTCGTGCACCCAACTGATCTTCAGCA
TCTTTTACTTTCACCAGCGTTTCTGGGTGAGCAAAAACAGGAAGGCAAAATGCCGCAAAAAAGGGAATAAGGGCGACACGGAAATGTTGAATACTCATACTCTTCCTTTTTCA
ATATTATTGAAGCATTTATCAGGGTTATTGTCTCATGAGCGGATACATATTTGAATGTATTTAGAAAAATAAACAAATAGGGGTTCCGCGCACATTTCCCCGAAAAGTGCCACC
TGACGTCTAAGAAACCATTATTATCATGACATTAACCTATAAAAATAGGCGTATCACGAGGCCCTTTCGTCTCGCGCGTTTCGGTGATGACGGTGAAAACCTCTGACACATGC
AGCTCCCGGAGACGGTCACAGCTTGTCTGTAAGCGGATGCCGGGAGCAGACAAGCCCGTCAGGGCGCGTCAGCGGGTGTTGGCGGGTGTCGGGGCTGGCTTAACTATGC
GGCATCAGAGCAGATTGTACTGAGAGTGCACCATATGGACATATTGTCGTTAGAACGCGGCTACAATTAATACATAACCTTATGTATCATACACATACGATTTAGGTGACACT
ATA

Linker+GFP; *HygB$^R$* marker (replaced with a *HIS1* marker in pLC1206)

>pLC1211_pFA-HA-HYGB

GAACGCGGCCGCCAGCTGAAGCTTCGTACGCTGCAGGTCGACGGATCCCCGGGTACCCATACGATGTTCCTGACTATGCGGGCTATCCCTATGACGTCCCGGACTATGCA
GGATATCCATATGACGTTCCAGATTACGCTTAGGGCGCGCCcttgctgttcgatattgctagaggcaaagaaaaaggattacctcacgcctaaaatggaatttgaaaattgaggagatgagttgagcttttgtcttttacactttccct
attcaattgtgccgcaaaaacttcaatatctaacacagtgtgaaatatttgttatgtataccgaaagccaaataccatttaaactggtatttcaagatttcataaatcaagtttctggttaaattaattgctgttaaacagaacaagagcttcacattgatgttggaata
actactatagaattataaaatacacaaaaccaggtcgtgtacgcaataacttttcaatgtatgaaaaaattttttttttttttgcttcttctcttcgcttctttttctagttatcaatttggcaatgaattgatttgattgccctttgtttagtttactatgaaaaaaaatttttttttgt
ctctgtcagattttctctcatttttttttgcctcataatgaacaactcaaaaaagaatataaatttctcatcaaagacctttccaattgaaaatttttctttccttcttttcctttttttttctttctttcttctttttctaagtatacattttatataatcaatcctcgagatgaaaaaacc
agaattgactgctacttctgttgaaaaatttttgattgaaaaatttgattctgtttctgatttgatgcaattgtctgaaggtgaagaatcaagagctttttcttttgatgttggtggtagaggttatgttttgagagttaattcttgtgctgatggtttttataaagatagatatgtttat
agacattttgcttctgctgctttgccaattccagaagttttggatattggtgaattttctgaatctttgacttattgtatttcaagaagagctcaaggtgttactttgcaagatttgccagaaactgaattgccagctgttttgcaaccagttgctgaagctatggatgctattg
ctgctgctgatttgtctcaaacttctggttttggtccattggtccacaaggtattggtcaatatactacttggagagattttatttgtgctattgctgatccacatgtttatcattggcaaactgttatggatgatactgtttctgcttctgttgctcaagcattggatgaattgatg
ttgtgggctgaagattgtccagaagttagacatttggttcatgctgattttggttctaataatgttttgactgataatggtagaattactgctgttattgattggtctgaagctatgtttggtgattctcaatatgaagttgctaatatttttttttggagaccatggttggcttgtatg
gaacaacaaactagatattttgaaagaagacatccagaattggctggttctccaagattgagagcttatatgttgagaattggtttggatcaattgtatcaatctttggttgatggtaattttgatgatgctgcttgggctcaaggtagatgtgatgctattgttagatca
ggtgctggtactgttggtagaactcaaattgctagaagatcagctgctgtttggactgatggttgtgttgaagttttggctgattctggtaatagaagaccatctactagaccaagagctaaagaataaggatcGTTTAAACGAGCTCGAATTCATC
GATGATATCAGATCCACTAGTGGCCTATGCGGCCGCGGATCTGCCGGTCTCCCTATAGTGAGTCGTATTAATTTCGATAAGCCAGGTTAACCTGCATTAATGAATCGGCCAA
CGCGCGGGGAGAGGCGGTTTGCGTATTGGGCGCTCTTCCGCTTCCTCGCTCACTGACTCGCTGCGCTCGGTCGTTCGGCTGCGGCGAGCGGTATCAGCTCACTCAAAGG
CGGTAATACGGTTATCCACAGAATCAGGGGATAACGCAGGAAAGAACATGTGAGCAAAAGGCCAGCAAAAGGCCAGGAACCGTAAAAAGGCCGCGTTGCTGGCGTTTTTCC
ATAGGCTCCGCCCCCCTGACGAGCATCACAAAAATCGACGCTCAAGTCAGAGGTGGCGAAACCCGACAGGACTATAAAGATACCAGGCGTTTCCCCCTGGAAGCTCCCTC
GTGCGCTCTCCTGTTCCGACCCTGCCGCTTACCGGATACCTGTCCGCCTTTCTCCCTTCGGGAAGCGTGGCGCTTTCTCAATGCTCACGCTGTAGGTATCTCAGTTCGGTGT
AGGTCGTTCGCTCCAAGCTGGGCTGTGTGCACGAACCCCCCGTTCAGCCCGACCGCTGCGCCTTATCCGGTAACTATCGTCTTGAGTCCAACCCGGTAAGACACGACTTAT
CGCCACTGGCAGCAGCCACTGGTAACAGGATTAGCAGAGCGAGGTATGTAGGCGGTGCTACAGAGTTCTTGAAGTGGTGGCCTAACTACGGCTACACTAGAAGGACAGTA
TTTGGTATCTGCGCTCTGCTGAAGCCAGTTACCTTCGGAAAAAGAGTTGGTAGCTCTTGATCCGGCAAACAAACCACCGCTGGTAGCGGTGGTTTTTTTGTTTGCAAGCAGC
AGATTACGCGCAGAAAAAAAGGATCTCAAGAAGATCCTTTGATCTTTTCTACGGGGTCTGACGCTCAGTGGAACGAAAACTCACGTTAAGGGATTTTGGTCATGAGATTATCA
AAAAGGATCTTCACCTAGATCCTTTTAAATTAAAAATGAAGTTTTAAATCAATCTAAAGTATATATGAGTAAACTTGGTCTGACAGTTACCAATGCTTAATCAGTGAGGCACCTA
TCTCAGCGATCTGTCTATTTCGTTCATCCATAGTTGCCTGACTCCCCGTCGTGTAGATAACTACGATACGGGAGGGCTTACCATCTGGCCCCAGTGCTGCAATGATACCGCG
AGACCCACGCTCACCGGCTCCAGATTTATCAGCAATAAACCAGCCAGCCGGAAGGGCCGAGCGCAGAAGTGGTCCTGCAACTTTATCCGCCTCCATCCAGTCTATTAATTG
TTGCCGGGAAGCTAGAGTAAGTAGTTCGCCAGTTAATAGTTTGCGCAACGTTGTTGCCATTGCTACAGGCATCGTGGTGTCACGCTCGTCGTTTGGTATGGCTTCATTCAGC
TCCGGTTCCCAACGATCAAGGCGAGTTACATGATCCCCCATGTTGTGCAAAAAAGCGGTTAGCTCCTTCGGTCCTCCGATCGTTGTCAGAAGTAAGTTGGCCGCAGTGTTAT
CACTCATGGTTATGGCAGCACTGCATAATTCTCTTACTGTCATGCCATCCGTAAGATGCTTTTCTGTGACTGGTGAGTACTCAACCAAGTCATTCTGAGAATAGTGTATGCGG
CGACCGAGTTGCTCTTGCCCGGCGTCAATACGGGATAATACCGCGCCACATAGCAGAACTTTAAAAGTGCTCATCATTGGAAAACGTTCTTCGGGGCGAAAACTCTCAAGGA
TCTTACCGCTGTTGAGATCCAGTTCGATGTAACCCACTCGTGCACCCAACTGATCTTCAGCATCTTTTACTTTCACCAGCGTTTCTGGGTGAGCAAAAACAGGAAGGCAAAAT
GCCGCAAAAAAGGGAATAAGGGCGACACGGAAATGTTGAATACTCATACTCTTCCTTTTTCAATATTATTGAAGCATTTATCAGGGTTATTGTCTCATGAGCGGATACATATTT
GAATGTATTTAGAAAAATAAACAAATAGGGGTTCCGCGCACATTTCCCCGAAAAGTGCCACCTGACGTCTAAGAAACCATTATTATCATGACATTAACCTATAAAAATAGGCGT
ATCACGAGGCCCTTTCGTCTCGCGCGTTTCGGTGATGACGGTGAAAACCTCTGACACATGCAGCTCCCGGAGACGGTCACAGCTTGTCTGTAAGCGGATGCCGGGAGCAG
ACAAGCCCGTCAGGGCGCGTCAGCGGGTGTTGGCGGGTGTCGGGGCTGGCTTAACTATGCGGCATCAGAGCAGATTGTACTGAGAGTGCACCATATGGACATATTGTCGT
TAGAACGCGGCTACAATTAATACATAACCTTATGTATCATACACATACGATTTAGGTGACACTATA

Linker+3xHA; *HygB*$^{R}$ marker

>pLC1215_pFA-RFP-HYGB

GAACGCGGCCGCCAGCTGAAGCTTCGTACGCTGCAGGTCGACGGATCCCCGGTGGTGGTGGTGTTTCAAAAGGTGAAGATGATAATATGGCTATTATTAAAGAATTTATGA
GATTTAAAGTTCATATGGAAGGTTCAGTTAATGGTCATGAATTTGAAATTGAAGGTGAAGGTGAAGGTAGACCATATGAAGGTACTCAAACTGCTAAATTGAAAGTTACTAAAG
GTGGTCCATTACCATTTGCTTGGGATATTTTGTCACCACAATTTATGTATGGTTCAAAAGCTTATGTTAAACATCCAGCTGATATTCCAGATTATTTAAAATTGTCATTTCCAGAA
GGTTTTAAATGGGAAAGAGTTATGAATTTTGAAGATGGTGGTGTTGTTACTGTTACTCAAGATTCATCATTACAAGATGGTGAATTTATTTATAAAGTTAAATTGAGAGGTACTA
ATTTTCCATCAGATGGTCCAGTTATGCAAAAAAAAACTATGGGTTGGGAAGCTTCATCAGAAAGAATGTATCCAGAAGATGGTGCTTTAAAAGGTGAAATTAAACAAAGATTGA
AATTAAAAGATGGTGGTCATTATGATGCTGAAGTTAAAACTACTTATAAAGCTAAAAAACCAGTTCAATTACCAGGTGCTTATAATGTTAATATTAAATTGGATATTACTTCACAT
AATGAAGATTATACTATTGTTGAACAATATGAAAGAGCTGAAGGTAGACATTCAACTGGTGGTATGGATGAATTATATAAATAAGGCGCGCCcttgctgttcgatattgctagaggcaaagaaa
aaggattacctcacgcctaaaatggaatttgaaaattgaggagatgagttgagcttttgtcttttacactttccctattcaattgtgccgcaaaaacttcaatatctaacacagtgtgaaatatttgttatgtataccgaaagccaaataccatttaaactggtatttcaa
gatttcataaatcaagtttctggttaaattaattgctgttaaacagaacaagagcttcacattgatgttggaataactactatagaattataaaatacacaaaaccaggtcgtgtacgcaataacttttcaatgtatgaaaaaattttttttttttttgcttcttctcttcgctt
ctttttctagttatcaatttggcaatgaattgatttgattgccctttgtttagtttactatgaaaaaaaattttttttttgtctctgtcagattttctctcattttttttttgcctcataatgaacaactcaaaaaagaatataaatttctcatcaaagacctttccaattgaaaatttttct
ttccttcttttcctttttttttctttctttcttcttttctaagtatacattttatataatcaatcctcgagatgaaaaaaccagaattgactgctacttctgttgaaaaatttttgattgaaaaatttgattctgtttctgatttgatgcaattgtctgaaggtgaagaatcaagagcttttt
cttttgatgttggtggtagaggttatgttttgagagttaattcttgtgctgatggtttttataaagatagatatgtttatagacattttgcttctgctgctttgccaattccagaagttttggatattggtgaattttctgaatctttgacttattgtatttcaagaagagctcaaggtgtt
actttgcaagatttgccagaaactgaattgccagctgttttgcaaccagttgctgaagctatggatgctattgctgctgctgatttgtctcaaacttctggttttggtccatttggtccacaaggtattggtcaatatactacttggagagattttatttgtgctattgctgatcc
acatgtttatcattggcaaactgttatggatgatactgtttctgcttctgttgctcaagcattggatgaattgatgttgtgggctgaagattgtccagaagttagacatttggttcatgctgattttggttctaataatgttttgactgataatggtagaattactgctgttattgat
tggtctgaagctatgtttggtgattctcaatatgaagttgctaatatttttttttggagaccatggttggcttgtatggaacaacaaactagatattttgaaagaagacatccagaattggctggttctccaagattgagagcttatatgttgagaattggtttggatcaatt
gtatcaatctttggttgatggtaattttgatgatgctgcttgggctcaaggtagatgtgatgctattgttagatcaggtgctggtactgttggtagaactcaaattgctagaagatcagctgctgtttggactgatggttgtgttgaagttttggctgattctggtaatagaag
accatctactagaccaagagctaaagaataaggatcGTTTAAACGAGCTCGAATTCATCGATGATATCAGATCCACTAGTGGCCTATGCGGCCGCGGATCTGCCGGTCTCCCTATAGTGAGT
CGTATTAATTTCGATAAGCCAGGTTAACCTGCATTAATGAATCGGCCAACGCGCGGGGAGAGGCGGTTTGCGTATTGGGCGCTCTTCCGCTTCCTCGCTCACTGACTCGCT
GCGCTCGGTCGTTCGGCTGCGGCGAGCGGTATCAGCTCACTCAAAGGCGGTAATACGGTTATCCACAGAATCAGGGGATAACGCAGGAAAGAACATGTGAGCAAAAGGCC
AGCAAAAGGCCAGGAACCGTAAAAAGGCCGCGTTGCTGGCGTTTTTCCATAGGCTCCGCCCCCCTGACGAGCATCACAAAAATCGACGCTCAAGTCAGAGGTGGCGAAAC
CCGACAGGACTATAAAGATACCAGGCGTTTCCCCCTGGAAGCTCCCTCGTGCGCTCTCCTGTTCCGACCCTGCCGCTTACCGGATACCTGTCCGCCTTTCTCCCTTCGGGA
AGCGTGGCGCTTTCTCAATGCTCACGCTGTAGGTATCTCAGTTCGGTGTAGGTCGTTCGCTCCAAGCTGGGCTGTGTGCACGAACCCCCCGTTCAGCCCGACCGCTGCGC
CTTATCCGGTAACTATCGTCTTGAGTCCAACCCGGTAAGACACGACTTATCGCCACTGGCAGCAGCCACTGGTAACAGGATTAGCAGAGCGAGGTATGTAGGCGGTGCTAC
AGAGTTCTTGAAGTGGTGGCCTAACTACGGCTACACTAGAAGGACAGTATTTGGTATCTGCGCTCTGCTGAAGCCAGTTACCTTCGGAAAAAGAGTTGGTAGCTCTTGATCC
GGCAAACAAACCACCGCTGGTAGCGGTGGTTTTTTTGTTTGCAAGCAGCAGATTACGCGCAGAAAAAAAGGATCTCAAGAAGATCCTTTGATCTTTTCTACGGGGTCTGACG
CTCAGTGGAACGAAAACTCACGTTAAGGGATTTTGGTCATGAGATTATCAAAAAGGATCTTCACCTAGATCCTTTTAAATTAAAAATGAAGTTTTAAATCAATCTAAAGTATATA
TGAGTAAACTTGGTCTGACAGTTACCAATGCTTAATCAGTGAGGCACCTATCTCAGCGATCTGTCTATTTCGTTCATCCATAGTTGCCTGACTCCCCGTCGTGTAGATAACTA
CGATACGGGAGGGCTTACCATCTGGCCCCAGTGCTGCAATGATACCGCGAGACCCACGCTCACCGGCTCCAGATTTATCAGCAATAAACCAGCCAGCCGGAAGGGCCGAG
CGCAGAAGTGGTCCTGCAACTTTATCCGCCTCCATCCAGTCTATTAATTGTTGCCGGGAAGCTAGAGTAAGTAGTTCGCCAGTTAATAGTTTGCGCAACGTTGTTGCCATTGC
TACAGGCATCGTGGTGTCACGCTCGTCGTTTGGTATGGCTTCATTCAGCTCCGGTTCCCAACGATCAAGGCGAGTTACATGATCCCCCATGTTGTGCAAAAAAGCGGTTAGC
TCCTTCGGTCCTCCGATCGTTGTCAGAAGTAAGTTGGCCGCAGTGTTATCACTCATGGTTATGGCAGCACTGCATAATTCTCTTACTGTCATGCCATCCGTAAGATGCTTTTC
TGTGACTGGTGAGTACTCAACCAAGTCATTCTGAGAATAGTGTATGCGGCGACCGAGTTGCTCTTGCCCGGCGTCAATACGGGATAATACCGCGCCACATAGCAGAACTTTA
AAAGTGCTCATCATTGGAAAACGTTCTTCGGGGCGAAAACTCTCAAGGATCTTACCGCTGTTGAGATCCAGTTCGATGTAACCCACTCGTGCACCCAACTGATCTTCAGCAT
CTTTTACTTTCACCAGCGTTTCTGGGTGAGCAAAAACAGGAAGGCAAAATGCCGCAAAAAAGGGAATAAGGGCGACACGGAAATGTTGAATACTCATACTCTTCCTTTTTCAA
TATTATTGAAGCATTTATCAGGGTTATTGTCTCATGAGCGGATACATATTTGAATGTATTTAGAAAAATAAACAAATAGGGGTTCCGCGCACATTTCCCCGAAAAGTGCCACCT
GACGTCTAAGAAACCATTATTATCATGACATTAACCTATAAAAATAGGCGTATCACGAGGCCCTTTCGTCTCGCGCGTTTCGGTGATGACGGTGAAAACCTCTGACACATGCA
GCTCCCGGAGACGGTCACAGCTTGTCTGTAAGCGGATGCCGGGAGCAGACAAGCCCGTCAGGGCGCGTCAGCGGGTGTTGGCGGGTGTCGGGGCTGGCTTAACTATGC
GGCATCAGAGCAGATTGTACTGAGAGTGCACCATATGGACATATTGTCGTTAGAACGCGGCTACAATTAATACATAACCTTATGTATCATACACATACGATTTAGGTGACACT
ATA

Linker+RFP; *HygB*$^{R}$ marker

>pLC1696_pFA-NeonGreen-HYGB

GAACGCGGCCGCCAGCTGAAGCTTCGTACGCTGCAGGTCGACGGATCCCCggtggtagtggtatggtttctaaaggtgaagaagataatatggctagtttgcctgcaacacatgaattacatattttggtagtattaatggt
gttgattttgatatggttggtcaaggtactggtaatccaaatgatggttatgaagaattgaatttgaaatcaactaaaggagatttgcaattttctccatggattttggttccacatattggttatggtttccatcaatatttgccatatccagatggaatgtcaccatttcaa
gctgctatggttgatggttctggttatcaagttcatagaactatgcaattcgaagatggtgcttcattgactgttaattatagatatacttatgaaggttctcatattaaaggtgaagctcaagttaaaggtactggttttccagctgatggtccagttatgactaattcatta
actgctgctgattggtgtagatcaaagaaaacttatccaaatgataaaactattatttcaacttttaaatggtcttatactactggtaatggtaaaagatatagatcaactgctagaactacttatactttcgctaaaccaatggctgctaattatttgaaaaatcaacc
aatgtatgtttttagaaaaactgaattaaaacattctaaaactgaattgaatttcaaagaatggcaaaaagcattcactgatgttatgggtatggatgaattgtataaatagctctagGGCGCGCCcttgctgttcgatattgctagaggcaaagaaaaagg
attacctcacgcctaaaatggaatttgaaaattgaggagatgagttgagcttttgtcttttacactttccctattcaattgtgccgcaaaaacttcaatatctaacacagtgtgaaatatttgttatgtataccgaaagccaaataccatttaaactggtatttcaagattt
cataaatcaagtttctggttaaattaattgctgttaaacagaacaagagcttcacattgatgttggaataactactatagaattataaaatacacaaaaccaggtcgtgtacgcaataacttttcaatgtatgaaaaaatttttttttttttgcttcttctcttcgcttcttttt
ctagttatcaatttggcaatgaattgatttgattgccctttgtttagtttactatgaaaaaaaaatttttttttgtctctgtcagattttctctcatttttttttgcctcataatgaacaactcaaaaaagaatataaatttctcatcaaagacctttccaattgaaaatttttctttcctt
cttttcctttttttttttctttctttcttcttttctaagtatacattttatataatcaatcctcgagatgaaaaaaccagaattgactgctacttctgttgaaaaatttttgattgaaaaatttgattctgtttctgatttgatgcaattgtctgaaggtgaagaatcaagagcttttttctttg
atgttggtggtagaggttatgttttgagagttaattcttgtgctgatggtttttataaagatagatatgtttatagacattttgcttctgctgctttgccaattccagaagttttggatattggtgaattttctgaatctttgacttattgtatttcaagaagagctcaaggtgttacttt
gcaagatttgccagaaactgaattgccagctgttttgcaaccagttgctgaagctatggatgctattgctgctgctgatttgtctcaaacttctggttttggtccatttggtccacaaggtattggtcaatatactacttggagagattttatttgtgctattgctgatccacat
gtttatcattggcaaactgttatggatgatactgtttctgcttctgttgctcaagcattggatgaattgatgttgtgggctgaagattgtccagaagttagacatttggttcatgctgattttggttctaataatgttttgactgataatggtagaattactgctgttattgattggt
ctgaagctatgtttggtgattctcaatatgaagttgctaatattttttttggagaccatggttggcttgtatggaacaacaaactagatattttgaaagaagacatccagaattggctggttctccaagattgagagcttatatgttgagaattggtttggatcaattgtat
caatctttggttgatggtaattttgatgatgctgcttgggctcaaggtagatgtgatgctattgttagatcaggtgctggtactgttggtagaactcaaattgctagaagatcagctgctgtttggactgatggttgtgttgaagttttggctgattctggtaatagaagacc
atctactagaccaagagctaaagaataaggatcGTTTAAACGAGCTCGAATTCATCGATGATATCAGATCCACTAGTGGCCTATGCGGCCGCGGATCTGCCGGTCTCCCTATAGTGAGTCG
TATTAATTTCGATAAGCCAGGTTAACCTGCATTAATGAATCGGCCAACGCGCGGGGAGAGGCGGTTTGCGTATTGGGCGCTCTTCCGCTTCCTCGCTCACTGACTCGCTGC
GCTCGGTCGTTCGGCTGCGGCGAGCGGTATCAGCTCACTCAAAGGCGGTAATACGGTTATCCACAGAATCAGGGGATAACGCAGGAAAGAACATGTGAGCAAAAGGCCAG
CAAAAGGCCAGGAACCGTAAAAAGGCCGCGTTGCTGGCGTTTTTCCATAGGCTCCGCCCCCCTGACGAGCATCACAAAAATCGACGCTCAAGTCAGAGGTGGCGAAACCC
GACAGGACTATAAAGATACCAGGCGTTTCCCCCTGGAAGCTCCCTCGTGCGCTCTCCTGTTCCGACCCTGCCGCTTACCGGATACCTGTCCGCCTTTCTCCCTTCGGGAAG
CGTGGCGCTTTCTCAATGCTCACGCTGTAGGTATCTCAGTTCGGTGTAGGTCGTTCGCTCCAAGCTGGGCTGTGTGCACGAACCCCCCGTTCAGCCCGACCGCTGCGCCTT
ATCCGGTAACTATCGTCTTGAGTCCAACCCGGTAAGACACGACTTATCGCCACTGGCAGCAGCCACTGGTAACAGGATTAGCAGAGCGAGGTATGTAGGCGGTGCTACAGA
GTTCTTGAAGTGGTGGCCTAACTACGGCTACACTAGAAGGACAGTATTTGGTATCTGCGCTCTGCTGAAGCCAGTTACCTTCGGAAAAAGAGTTGGTAGCTCTTGATCCGGC
AAACAAACCACCGCTGGTAGCGGTGGTTTTTTTGTTTGCAAGCAGCAGATTACGCGCAGAAAAAAAGGATCTCAAGAAGATCCTTTGATCTTTTCTACGGGGTCTGACGCTC
AGTGGAACGAAAACTCACGTTAAGGGATTTTGGTCATGAGATTATCAAAAAGGATCTTCACCTAGATCCTTTTAAATTAAAAATGAAGTTTTAAATCAATCTAAAGTATATATGA
GTAAACTTGGTCTGACAGTTACCAATGCTTAATCAGTGAGGCACCTATCTCAGCGATCTGTCTATTTCGTTCATCCATAGTTGCCTGACTCCCCGTCGTGTAGATAACTACGA
TACGGGAGGGCTTACCATCTGGCCCCAGTGCTGCAATGATACCGCGAGACCCACGCTCACCGGCTCCAGATTTATCAGCAATAAACCAGCCAGCCGGAAGGGCCGAGCGC
AGAAGTGGTCCTGCAACTTTATCCGCCTCCATCCAGTCTATTAATTGTTGCCGGGAAGCTAGAGTAAGTAGTTCGCCAGTTAATAGTTTGCGCAACGTTGTTGCCATTGCTAC
AGGCATCGTGGTGTCACGCTCGTCGTTTGGTATGGCTTCATTCAGCTCCGGTTCCCAACGATCAAGGCGAGTTACATGATCCCCCATGTTGTGCAAAAAAGCGGTTAGCTCC
TTCGGTCCTCCGATCGTTGTCAGAAGTAAGTTGGCCGCAGTGTTATCACTCATGGTTATGGCAGCACTGCATAATTCTCTTACTGTCATGCCATCCGTAAGATGCTTTTCTGT
GACTGGTGAGTACTCAACCAAGTCATTCTGAGAATAGTGTATGCGGCGACCGAGTTGCTCTTGCCCGGCGTCAATACGGGATAATACCGCGCCACATAGCAGAACTTTAAAA
GTGCTCATCATTGGAAAACGTTCTTCGGGGCGAAAACTCTCAAGGATCTTACCGCTGTTGAGATCCAGTTCGATGTAACCCACTCGTGCACCCAACTGATCTTCAGCATCTTT
TACTTTCACCAGCGTTTCTGGGTGAGCAAAAACAGGAAGGCAAAATGCCGCAAAAAAGGGAATAAGGGCGACACGGAAATGTTGAATACTCATACTCTTCCTTTTTCAATATT
ATTGAAGCATTTATCAGGGTTATTGTCTCATGAGCGGATACATATTTGAATGTATTTAGAAAAATAAACAAATAGGGGTTCCGCGCACATTTCCCCGAAAAGTGCCACCTGAC
GTCTAAGAAACCATTATTATCATGACATTAACCTATAAAAATAGGCGTATCACGAGGCCCTTTCGTCTCGCGCGTTTCGGTGATGACGGTGAAAACCTCTGACACATGCAGCT
CCCGGAGACGGTCACAGCTTGTCTGTAAGCGGATGCCGGGAGCAGACAAGCCCGTCAGGGCGCGTCAGCGGGTGTTGGCGGGTGTCGGGGCTGGCTTAACTATGCGGC
ATCAGAGCAGATTGTACTGAGAGTGCACCATATGGACATATTGTCGTTAGAACGCGGCTACAATTAATACATAACCTTATGTATCATACACATACGATTTAGGTGACACTATA

Linker+mNeonGreen; *HygB*$^{R}$ marker

>pLC1697_pFA-mScarlet-HYGB

GAACGCGGCCGCCAGCTGAAGCTTCGTACGCTGCAGGTCGACGGATCCCCggtggtagtggtatggtttctaaaggtgaagcagttattaaagaattcatgagattcaaagttcatatggaaggtagtatgaatggtcatg
aatttgaaattgaaggtgaaggtgaaggtagaccatatgaaggtactcaaactgctaaattgaaagttactaaaggtggtccattaccattttcatgggatattttgtcaccacaattcatgtatggttcaagagcttttattaaacatccagctgatattccagattat
tataaacaatcttttccagaaggttttaaatgggaaagagttatgaatttcgaagatggtggtgctgttactgttactcaagatacttcattagaagatggtactttgatctataaagttaaattgagaggtactaattttccaccagatggtccagttatgcaaaagaa
aactatgggttgggaagcttctactgaaagattatatccagaagatggtgttttgaaaggagatattaaaatggctttgagattgaaagatggtggtagatatttggctgatttcaaaactacttataaagctaagaaaccagttcaaatgccaggtgcttataatgt
tgatagaaaattggatattacttcacataatgaagattatactgttgttgaacaatatgaaagaagtgaaggtagacatagtacaggtggtatggatgaattatattagctctagGGCGCGCCcttgctgttcgatattgctagaggcaaagaaaaaggatt
acctcacgcctaaaatggaatttgaaaattgaggagatgagttgagcttttgtcttttacactttccctattcaattgtgccgcaaaaacttcaatatctaacacagtgtgaaatatttgttatgtataccgaaagccaaataccatttaaactggtatttcaagatttcat
aaatcaagtttctggttaaattaattgctgttaaacagaacaagagcttcacattgatgttggaataactactatagaattataaaatacacaaaaccaggtcgtgtacgcaataacttttcaatgtatgaaaaaattttttttttttttgcttcttctcttcgcttctttttcta
gttatcaatttggcaatgaattgatttgattgccctttgtttagtttactatgaaaaaaaattttttttgtctctgtcagattttctctcatttttttttttgcctcataatgaacaactcaaaaaagaatataaatttctcatcaaagacctttccaattgaaaatttttctttccttcttt
tcctttttttttttcttctttcttcttttctaagtatacattttatataatcaatcctcgagatgaaaaaaccagaattgactgctacttctgttgaaaaatttttgattgaaaaatttgattctgtttctgatttgatgcaattgtctgaaggtgaagaatcaagagctttttcttttgatg
ttggtggtagaggttatgttttgagagttaattcttgtgctgatggtttttataaagatagatatgtttatagacattttgcttctgctgctttgccaattccagaagttttggatattggtgaattttctgaatctttgacttattgtatttcaagaagagctcaaggtgttactttgca
agatttgccagaaactgaattgccagctgttttgcaaccagttgctgaagctatggatgctattgctgctgctgatttgtctcaaacttctggttttggtccatttggtccacaaggtattggtcaatatactacttggagagattttatttgtgctattgctgatccacatgttt
atcattggcaaactgttatggatgatactgtttctgcttctgttgctcaagcattggatgaattgatgttgtgggctgaagattgtccagaagttagacatttggttcatgctgattttggttctaataatgttttgactgataatggtagaattactgctgttattgattggtctg
aagctatgtttggtgattctcaatatgaagttgctaatatttttttttggagaccatggttggcttgtatggaacaacaaactagatattttgaaagaagacatccagaattggctggttctccaagattgagagcttatatgttgagaattggtttggatcaattgtatcaa
tctttggttgatggtaattttgatgatgctgcttgggctcaaggtagatgtgatgctattgttagatcaggtgctggtactgttggtagaactcaaattgctagaagatcagctgctgtttggactgatggttgtgttgaagttttggctgattctggtaatagaagaccatct
actagaccaagagctaaagaataaggatcGTTTAAACGAGCTCGAATTCATCGATGATATCAGATCCACTAGTGGCCTATGCGGCCGCGGATCTGCCGGTCTCCCTATAGTGAGTCGTAT
TAATTTCGATAAGCCAGGTTAACCTGCATTAATGAATCGGCCAACGCGCGGGGAGAGGCGGTTTGCGTATTGGGCGCTCTTCCGCTTCCTCGCTCACTGACTCGCTGCGCT
CGGTCGTTCGGCTGCGGCGAGCGGTATCAGCTCACTCAAAGGCGGTAATACGGTTATCCACAGAATCAGGGGATAACGCAGGAAAGAACATGTGAGCAAAAGGCCAGCAA
AAGGCCAGGAACCGTAAAAAGGCCGCGTTGCTGGCGTTTTTCCATAGGCTCCGCCCCCCTGACGAGCATCACAAAAATCGACGCTCAAGTCAGAGGTGGCGAAACCCGAC
AGGACTATAAAGATACCAGGCGTTTCCCCCTGGAAGCTCCCTCGTGCGCTCTCCTGTTCCGACCCTGCCGCTTACCGGATACCTGTCCGCCTTTCTCCCTTCGGGAAGCGT
GGCGCTTTCTCAATGCTCACGCTGTAGGTATCTCAGTTCGGTGTAGGTCGTTCGCTCCAAGCTGGGCTGTGTGCACGAACCCCCCGTTCAGCCCGACCGCTGCGCCTTATC
CGGTAACTATCGTCTTGAGTCCAACCCGGTAAGACACGACTTATCGCCACTGGCAGCAGCCACTGGTAACAGGATTAGCAGAGCGAGGTATGTAGGCGGTGCTACAGAGTT
CTTGAAGTGGTGGCCTAACTACGGCTACACTAGAAGGACAGTATTTGGTATCTGCGCTCTGCTGAAGCCAGTTACCTTCGGAAAAAGAGTTGGTAGCTCTTGATCCGGCAAA
CAAACCACCGCTGGTAGCGGTGGTTTTTTTGTTTGCAAGCAGCAGATTACGCGCAGAAAAAAAGGATCTCAAGAAGATCCTTTGATCTTTTCTACGGGGTCTGACGCTCAGT
GGAACGAAAACTCACGTTAAGGGATTTTGGTCATGAGATTATCAAAAAGGATCTTCACCTAGATCCTTTTAAATTAAAAATGAAGTTTTAAATCAATCTAAAGTATATATGAGTA
AACTTGGTCTGACAGTTACCAATGCTTAATCAGTGAGGCACCTATCTCAGCGATCTGTCTATTTCGTTCATCCATAGTTGCCTGACTCCCCGTCGTGTAGATAACTACGATAC
GGGAGGGCTTACCATCTGGCCCCAGTGCTGCAATGATACCGCGAGACCCACGCTCACCGGCTCCAGATTTATCAGCAATAAACCAGCCAGCCGGAAGGGCCGAGCGCAGA
AGTGGTCCTGCAACTTTATCCGCCTCCATCCAGTCTATTAATTGTTGCCGGGAAGCTAGAGTAAGTAGTTCGCCAGTTAATAGTTTGCGCAACGTTGTTGCCATTGCTACAGG
CATCGTGGTGTCACGCTCGTCGTTTGGTATGGCTTCATTCAGCTCCGGTTCCCAACGATCAAGGCGAGTTACATGATCCCCCATGTTGTGCAAAAAAGCGGTTAGCTCCTTC
GGTCCTCCGATCGTTGTCAGAAGTAAGTTGGCCGCAGTGTTATCACTCATGGTTATGGCAGCACTGCATAATTCTCTTACTGTCATGCCATCCGTAAGATGCTTTTCTGTGAC
TGGTGAGTACTCAACCAAGTCATTCTGAGAATAGTGTATGCGGCGACCGAGTTGCTCTTGCCCGGCGTCAATACGGGATAATACCGCGCCACATAGCAGAACTTTAAAAGTG
CTCATCATTGGAAAACGTTCTTCGGGGCGAAAACTCTCAAGGATCTTACCGCTGTTGAGATCCAGTTCGATGTAACCCACTCGTGCACCCAACTGATCTTCAGCATCTTTTAC
TTTCACCAGCGTTTCTGGGTGAGCAAAAACAGGAAGGCAAAATGCCGCAAAAAAGGGAATAAGGGCGACACGGAAATGTTGAATACTCATACTCTTCCTTTTTCAATATTATT
GAAGCATTTATCAGGGTTATTGTCTCATGAGCGGATACATATTTGAATGTATTTAGAAAAATAAACAAATAGGGGTTCCGCGCACATTTCCCCGAAAAGTGCCACCTGACGTC
TAAGAAACCATTATTATCATGACATTAACCTATAAAAATAGGCGTATCACGAGGCCCTTTCGTCTCGCGCGTTTCGGTGATGACGGTGAAAACCTCTGACACATGCAGCTCCC
GGAGACGGTCACAGCTTGTCTGTAAGCGGATGCCGGGAGCAGACAAGCCCGTCAGGGCGCGTCAGCGGGTGTTGGCGGGTGTCGGGGCTGGCTTAACTATGCGGCATCA
GAGCAGATTGTACTGAGAGTGCACCATATGGACATATTGTCGTTAGAACGCGGCTACAATTAATACATAACCTTATGTATCATACACATACGATTTAGGTGACACTATA

Linker+mScarlet; *HygB*$^R$ marker (replaced with an *ARG4* marker in pLC1693)

>pLC2041_pFA-NLS-TPK1-HIS

GAAGATCCTTTGATCTTTTCTACGGGGTCTGACGCTCAGTGGAACGAAAACTCACGTTAAGGGATTTTGGTCATGAGATTATCAAAAAGGATCTTCACCTAGATCCTTTTAAAT
TAAAAATGAAGTTTTAAATCAATCTAAAGTATATATGAGTAAACTTGGTCTGACAGTTACCAATGCTTAATCAGTGAGGCACCTATCTCAGCGATCTGTCTATTTCGTTCATCCA
TAGTTGCCTGACTCCCCGTCGTGTAGATAACTACGATACGGGAGGGCTTACCATCTGGCCCCAGTGCTGCAATGATACCGCGAGACCCACGCTCACCGGCTCCAGATTTAT
CAGCAATAAACCAGCCAGCCGGAAGGGCCGAGCGCAGAAGTGGTCCTGCAACTTTATCCGCCTCCATCCAGTCTATTAATTGTTGCCGGGAAGCTAGAGTAAGTAGTTCGC
CAGTTAATAGTTTGCGCAACGTTGTTGCCATTGCTACAGGCATCGTGGTGTCACGCTCGTCGTTTGGTATGGCTTCATTCAGCTCCGGTTCCCAACGATCAAGGCGAGTTAC
ATGATCCCCCATGTTGTGCAAAAAAGCGGTTAGCTCCTTCGGTCCTCCGATCGTTGTCAGAAGTAAGTTGGCCGCAGTGTTATCACTCATGGTTATGGCAGCACTGCATAATT
CTCTTACTGTCATGCCATCCGTAAGATGCTTTTCTGTGACTGGTGAGTACTCAACCAAGTCATTCTGAGAATAGTGTATGCGGCGACCGAGTTGCTCTTGCCCGGCGTCAATA
CGGGATAATACCGCGCCACATAGCAGAACTTTAAAAGTGCTCATCATTGGAAAACGTTCTTCGGGGCGAAAACTCTCAAGGATCTTACCGCTGTTGAGATCCAGTTCGATGT
AACCCACTCGTGCACCCAACTGATCTTCAGCATCTTTTACTTTCACCAGCGTTTCTGGGTGAGCAAAAACAGGAAGGCAAAATGCCGCAAAAAAGGGAATAAGGGCGACACG
GAAATGTTGAATACTCATACTCTTCCTTTTTCAATATTATTGAAGCATTTATCAGGGTTATTGTCTCATGAGCGGATACATATTTGAATGTATTTAGAAAAATAAACAAATAGGG
GTTCCGCGCACATTTCCCCGAAAAGTGCCACCTGACGTCTAAGAAACCATTATTATCATGACATTAACCTATAAAAATAGGCGTATCACGAGGCCCTTTCGTCTCGCGCGTTT
CGGTGATGACGGTGAAAACCTCTGACACATGCAGCTCCCGGAGACGGTCACAGCTTGTCTGTAAGCGGATGCCGGGAGCAGACAAGCCCGTCAGGGCGCGTCAGCGGGT
GTTGGCGGGTGTCGGGGCTGGCTTAACTATGCGGCATCAGAGCAGATTGTACTGAGAGTGCACCATATGGACATATTGTCGTTAGAACGCGGCTACAATTAATACATAACCT
TATGTATCATACACATACGATTTAGGTGACACTATAGAACGCGGCCGCCAGCTGAAGCTTCGTACGCTGCAGGTCGACGGATCCAGACAGAAGATAATATTTTTTCTTAACTG
TCACGCAAAAGTTTTAACTAACTAACTAACTAACTAATTAGACCAATTCCCCTAAAGGACTTAAGGACTAAAATAAGCTTATTTCAATTAATATTATCATTGAATAATAGATACTT
ATAGCAGTTATAGTAGAATTTAATTTCTTTTTTTTTTCTTTCTTTCTTTTGTCACACATTTATCCGTTTTGACAGACTAAATTAAACAAAAAATTTTTGAACATCAGGGAACGACCA
TATACAAATAAAAGCAGAAGAAAAAAACGGAAGAAGAAGAAGAAGAAAAAAAAGTCAACCAAAAAATTGAACAAGAGGTCTAAAGAACCACAACATTATACAAAAGAAAAGAA
ATTAAATTAAATTACATTAAAAGAGAATTGAAACATAACACTTGAAGAAGAAAATAAAAGTATTTTTATATTATATATTCCGACTAGTATTATCTGAGATCAATATATCCTATCGTT
ATCCTCCTCCTTCTCCTTTCAACTTTTGAAAAAGGTGATATTATTCAACACTGTTTTTGTTTTATCAACCAAAACCAGGATGGGTGCTCCTCCAAAAAAGAAGAGAAAGGTAGG
TACTGGTGGTGGTACATCCATGGAACCAGCAGACACAAGCATCAGGTCATTAAACGACATCAACTTACAAGAACTTGCCAACAAACAACATCAACGAAATATATATGCTCAAG
GATCACCTACACTAGAAGATTCAATAACATCAAATGGTAATGATATTAATAATCCCAACAATAATATAAATAAAACCAATAATAACAGTAATTCAAGTCTTTCCTTGACCAAGAAA
ACATCAAGACATATCAATAAAGACACTACAACTAAAGGCAAATATACATTAAATGATTTTCAAATATTACGAACATTAGGAACAGGTTCCTTTGGTAGAGTTCATTTAACTCGAT
CGATTCATAATGGTAGATTTTATGCTATGAAAGTATTAAAGAAACAACGAGTAGTACAAATGAAACAAATCGAACATACTAATGATGAAAGAAGAATGTTAAAATTAGCTCAACA
TCCATTTATAATTCGAATGTGGGGGACATTCCAAGATTGTCATAATTTATTTATGATTATGGATTATATAGAAGGAGGTGAATTATTTTCATTATTAAGAAAATCCCAACGATTCC
CTACTCCAGTAGCTAAATTTTATGCTGCCGAAGTATTTTTGGCTATTGAATATTTACACAGTTTAGACATTATTTATCGAGATTTAAAACCAGAAAATATATTATTGGATAAAAAT
GGTCATATCAAATTAACTGATTTTGGATTTGCTAAAGAAGTTCAAGATGTGACTTATACTTTATGTGGGACTCCAGATTATATTGCTCCTGAAGTGGTGGCAACAAAACCTTAT
AATAAATCAGTTGATTGGTGGTCATTTGGTATATTAATATTTGAAATGTTAACTGGTTATACTCCATTCTATGATCCTACTCCAATGAAAACTTATGAAAACATTTTAAATGGATC
AATTACTTATCCTGATTATTTACCTCCAGATATATTGGATCTTCTACAAAAATTAATTGTCAAAGATTTAACTCAAAGATTAGGTAATTTACAAGGTGGTTCTGATGATGTGAAAA
ATCATCCGTGGTTTAAAGAAGTTATTTGGGAAAGATTGTTGTCACGTGATATAGAAACTCCATATGAACCACCTATAACTTCTGGTGTTGGTGATACTTCACAATTTGATCGAT
ACCCAGAAGATAAAGATTTGGATTATGGTATAAGTGGAGTTGAAGACCCATATCGTGATCAATTCCAGGACTTTTAAATGTAAAAATAAAATTGTATTTATTGGCACAACAATGT
TAATTATGATAACTAGTTTTATAGATGTATAATTAATTTGCAAGAAAGAAGAAGACGAAGATTTGATTAGTGAATTTTTTAAAGCTAAAAAAACACTTCTTGGTGTATCAAAAGTA
ATTTTGGCAAATCTTATAGACAGCTAAAATATAAGCAGTGTCTTATTTTTAAACCAATGATCACTATATGAATTGGCTTCCGGCGCGCCATTATAAGTAAATGCATGTATACTAA
ACTCACAAATTAGAGCTTCAATTTAATTATATCAGTTATTACCCGGGAATCTCGGTCGTAATGATTTCTATAATGACGAAAAAAAAAAAATTGGAAAGAAAAAGCTTCATGGCCTT
TATAAAAAGGAACTATCCAATACCTCGCCAGAACCAAGTAACAGTATTTTACGGGGCACAAATCAAGAACAATAAGACAGGACTGTAAAGATGGACGCATAGATCCTGGAGG
ATGAGGAGACAGAAGTTAGTAGTAACAATTTGGGGACAGAAGAGGATACAGTGACATCAGAGGAAGAAGACAGCGAAGGTATAGTCAAAAAGCAATAGGACAGCAAGACAA
TTTATTTATTTAATAGTTTTTATAAATGTAAAATCTATCACAACATGTGTACTTAATTTTCTGTGGTAGGGGAGGCCATTTCGTTGAAACGTGTCGCGAGCGGTTGTGAGAATT
GACTCATTTTGATTATTACGCAACCATTGAAATTTTTTATTTTTTTTTGGTGAAGATTTTTCCCACACAACTTCTTCTTTTACTTAACCACCAACTACCGATAATGGATTTAGTCAA
TCATTTACCAGACCGTTTGTTATTTGCTGTTCCTAAAAAGGGCAGATTATACGAAAAATGCTGTAACTTATTGAGTGGTGCCGATATACAGTTTAGAAGATCTAATAGATTAGAT
ATAGCACTTTCTACAAACTTGCCAATTGCATTAATCTTCTTGCCTGCAGCTGATATCCCAGTTTTCGTTGGAGAAGGTAATTGTGACTTGGGTATAACTGGGTTAGACCAAATC
AAAGAAGCTGAACAATTCGACAACATCGAGGACTTGTTGGATTTGAAATTTGGTTCATGCAAATTGCAGATCCAAGTTCCAGCAGATGGCGAGTACGAAAAGCCAGAACAGC
TTGTTGGAAAGAAAATTGTGTCTTCATTTACAAAATTGAGTACCGACTATTTCAAACAATTGTCAGACAAACCTACTAATATCAGATATGTCGGTGGTTCCGTTGAGGCTTCTT
GTGCCTTGGGTGTTGCTGATGCTATTGTCGATTTGGTTGAAAGTGGTGAAACTATGAAAGCAGCTGGGTTAAAGGCCATAGAAACCATATTGGAGACTTCGGCTCATTTGATT
TCGTCAAAAAAAAGTAAATTCCCAGAAATGGTCAATATAATCGTCCAAAGACTTCAAGGTGTTTTAGCTGCCCAGGAATATGTCTTGTGTAACTACAATGCCCCAAAATCCATC
CAAGCAAAATGTTTAACCATTACTCCAGGCAGAAGGGCCGCTACTGTCTCTACTTTGGACAAACACAGCGACGACGAAGAGGACTGGGTTGCCATCTCATCCATGGTTAATA
GAAAGGAAATCGGTAATGTAATGGACGAATTGAAGAAAGCTGGTGCAACCGATATATTGGTACTCGAGATATCAAATTGTAGAGTTTAAAAGAAGTGATAGTTTCTCATAAATA
TTATACATATATACTTATAGATCTACTTCAATTTGACTCAATCTGTCTTATACCATCTTTATCTACCACCTTGATGTACACACCTTTAAAGTCAATAGGCATTCTCGTTTAAACGA
GCTCCACAATAGTTCAATATTGGAATCAAACATAATAGCGAGAATTGGTTTTTATCTGAGTACCACATTGAGAATGGTATTGGATGTTATTAAGTTGCCATTTTTGAATGAACTT
CTCACTGATGATTCTTTTTATTAAATCCAGGGTCCTTTTTTTAGGGTATTCTACTAAAACTTGATATTTGGGATGATTTGGTAGTTCTATTTTGATTTCCACTAATGAAAAGCATT
TCTAACAAGTTTTGGAAATTTGACTGAGATGTGCATTACTAAGAGCTTCGTTGTTACGGTGATACTAAAGTTTGTCAAGTCTCTTAAACATTACATTGAATACTATTTGATAGTT
GGTGATAAAAAAAACTCCGTAAACAATAATTTCGTGTTTAATTCACATTTTATTGGAGCATGAAAATTATTTAACCGAACGCGTTCTTGAATGTGTATAAAAATAGAAATCCCAT
ATTATCGAATTAGAGGTCTCCGCGGATCTGCCGGTCTCCCTATAGTGAGTCGTATTAATTTCGATAAGCCAGGTTAACCTGCATTAATGAATCGGCCAACGCGCGGGGAGAG
GCGGTTTGCGTATTGGGCGCTCTTCCGCTTCCTCGCTCACTGACTCGCTGCGCTCGGTCGTTCGGCTGCGGCGAGCGGTATCAGCTCACTCAAAGGCGGTAATACGGTTAT
CCACAGAATCAGGGGATAACGCAGGAAAGAACATGTGAGCAAAAGGCCAGCAAAAGGCCAGGAACCGTAAAAAGGCCGCGTTGCTGGCGTTTTTCCATAGGCTCCGCCCC
CCTGACGAGCATCACAAAAATCGACGCTCAAGTCAGAGGTGGCGAAACCCGACAGGACTATAAAGATACCAGGCGTTTCCCCCTGGAAGCTCCCTCGTGCGCTCTCCTGTT
CCGACCCTGCCGCTTACCGGATACCTGTCCGCCTTTCTCCCTTCGGGAAGCGTGGCGCTTTCTCATAGCTCACGCTGTAGGTATCTCAGTTCGGTGTAGGTCGTTCGCTCC
AAGCTGGGCTGTGTGCACGAACCCCCCGTTCAGCCCGACCGCTGCGCCTTATCCGGTAACTATCGTCTTGAGTCCAACCCGGTAAGACACGACTTATCGCCACTGGCAGCA
GCCACTGGTAACAGGATTAGCAGAGCGAGGTATGTAGGCGGTGCTACAGAGTTCTTGAAGTGGTGGCCTAACTACGGCTACACTAGAAGAACAGTATTTGGTATCTGCGCT
CTGCTGAAGCCAGTTACCTTCGGAAAAAGAGTTGGTAGCTCTTGATCCGGCAAACAAACCACCGCTGGTAGCGGTGGTTTTTTTGTTTGCAAGCAGCAGATTACGCGCAGAA
AAAAAGGATCTCAA

*TPK1* promoter; SV40 NLS+linker; *TPK1* ORF; *HIS1* marker; *TPK1* 3’ HR

>pLC2042_pFA-NLS-TPK2-HIS

GAAGATCCTTTGATCTTTTCTACGGGGTCTGACGCTCAGTGGAACGAAAACTCACGTTAAGGGATTTTGGTCATGAGATTATCAAAAAGGATCTTCACCTAGATCCTTTTAAAT
TAAAAATGAAGTTTTAAATCAATCTAAAGTATATATGAGTAAACTTGGTCTGACAGTTACCAATGCTTAATCAGTGAGGCACCTATCTCAGCGATCTGTCTATTTCGTTCATCCA
TAGTTGCCTGACTCCCCGTCGTGTAGATAACTACGATACGGGAGGGCTTACCATCTGGCCCCAGTGCTGCAATGATACCGCGAGACCCACGCTCACCGGCTCCAGATTTAT
CAGCAATAAACCAGCCAGCCGGAAGGGCCGAGCGCAGAAGTGGTCCTGCAACTTTATCCGCCTCCATCCAGTCTATTAATTGTTGCCGGGAAGCTAGAGTAAGTAGTTCGC
CAGTTAATAGTTTGCGCAACGTTGTTGCCATTGCTACAGGCATCGTGGTGTCACGCTCGTCGTTTGGTATGGCTTCATTCAGCTCCGGTTCCCAACGATCAAGGCGAGTTAC
ATGATCCCCCATGTTGTGCAAAAAAGCGGTTAGCTCCTTCGGTCCTCCGATCGTTGTCAGAAGTAAGTTGGCCGCAGTGTTATCACTCATGGTTATGGCAGCACTGCATAATT
CTCTTACTGTCATGCCATCCGTAAGATGCTTTTCTGTGACTGGTGAGTACTCAACCAAGTCATTCTGAGAATAGTGTATGCGGCGACCGAGTTGCTCTTGCCCGGCGTCAATA
CGGGATAATACCGCGCCACATAGCAGAACTTTAAAAGTGCTCATCATTGGAAAACGTTCTTCGGGGCGAAAACTCTCAAGGATCTTACCGCTGTTGAGATCCAGTTCGATGT
AACCCACTCGTGCACCCAACTGATCTTCAGCATCTTTTACTTTCACCAGCGTTTCTGGGTGAGCAAAAACAGGAAGGCAAAATGCCGCAAAAAAGGGAATAAGGGCGACACG
GAAATGTTGAATACTCATACTCTTCCTTTTTCAATATTATTGAAGCATTTATCAGGGTTATTGTCTCATGAGCGGATACATATTTGAATGTATTTAGAAAAATAAACAAATAGGG
GTTCCGCGCACATTTCCCCGAAAAGTGCCACCTGACGTCTAAGAAACCATTATTATCATGACATTAACCTATAAAAATAGGCGTATCACGAGGCCCTTTCGTCTCGCGCGTTT
CGGTGATGACGGTGAAAACCTCTGACACATGCAGCTCCCGGAGACGGTCACAGCTTGTCTGTAAGCGGATGCCGGGAGCAGACAAGCCCGTCAGGGCGCGTCAGCGGGT
GTTGGCGGGTGTCGGGGCTGGCTTAACTATGCGGCATCAGAGCAGATTGTACTGAGAGTGCACCATATGGACATATTGTCGTTAGAACGCGGCTACAATTAATACATAACCT
TATGTATCATACACATACGATTTAGGTGACACTATAGAACGCGGCCGCCAGCTGAAGCTTCGTACGCTGCAGGTCGACGGATCCACATTGTTAAAAGTCGATACTTCAGTTG
CTTTTTTTTATTATTTTCTATCCAGCAAAAAACACATTGTCATTGTTTGTGTATCATTTTTTGTCCAGATAACAAACACCAAATTGAGGTCAACTAAACAACAACTTGTATAGTTCT
ACCACACTATTTGAAGAGTCAGCTACAGTTCCTAGTTTCTCAACATAATATATCAACCTATCAATACTCGGATTGCAGGTCAATTTAGTTTACAACCACCAATTGCCAAAGTAT
TGGATTCTACTTTTTCCGACTTGTAAGTGAACACAAAGAAGATTAGACAAACAATCACCCACTCACACCTACTACTCACCCAATTTCCATTCCACCTTTTTTTTTCGAAAAAAAT
AACCTTCCCCCTTTTTGATGGTGAATCTTTTAAAGAAACTTCACATCACCAAGCTGCATCAACTGAATCAATCCAATTCGGACAGTAATTCCTTAAACTCAAACACATCAATGG
GTGCTCCTCCAAAAAAGAAGAGAAAGGTAGGTACTGGTGGTGGTGACAATCATCAACAACAACAGCAGCTTCAGCATCAGCAATATCAGCAACAATTTCAACAACCGCAGCA
ACAACTTTATCCAGGCGAACAAATAGTTCACCCTGCTGCAGCCCAAACTGGACAAAATACTACAAATGTAACAGCAGTGTCGAGCTCCAACATTACCCAATCTGCTACCTCAT
CACTCCATTCACAGCAATTGCAGCATGTAGATGTTTCCAAATCGGCTGCTGAAGAAGCCATCAGAAGATCGTTGTTGCCTGAACGTTCTACTGTTTCAAAGGGGAAATACTCT
TTGACTGATTTCTCAATTATGAGAACTTTGGGAACAGGTTCCTTTGGTAGAGTACATTTAGTGAGATCAGTTCACAATGGTAGATATTATGCTATCAAAGTTTTGAAGAAGCAT
CAAGTTGTGAAAATGAAGCAAGTTGAACACACAAATGATGAAAGAAGAATGTTGAAGTTGGTTGAACATCCATTTTTGATTAGAATGTGGGGGACTTTCCAAGATTCCAAAAAT
TTATTTATGGTTATGGACTATATTGAAGGGGGAGAGTTATTTTCATTGTTAAGAAAGTCTCAGAGATTTCCCAATCCAGTAGCCAAGTTTTATGCTGCAGAGGTTACCTTGGCT
TTGGAGTATTTGCACAGTCATGATATTATCTATCGTGATTTGAAACCAGAAAATATATTATTGGATAGAAATGGTCATATAAAGATTACAGATTTTGGATTTGCCAAAGAAGTTA
GTACCGTTACATGGACGTTATGTGGTACCCCCGATTATATTGCCCCAGAAGTCATAACAACCAAACCCTATAACAAGTCGGTGGACTGGTGGTCGTTGGGGGTATTGATTTTT
GAAATGTTGGCAGGTTACACTCCATTTTATGATTCGACTCCAATGAAAACTTATGAAAAAATTTTGGCAGGCAAAATACATTACCCAAGTTTTTTTCAACCTGATGTGATTGATT
TGTTGACTAAATTAATAACTGCTGATTTGACAAGAAGATTGGGTAATTTGATCAACGGACCAGCAGATATCAGAAATCATCCCTGGTTTCTGGAAGTGGTATGGGAGAAATTG
TTGGCAAAGGATATTGAAACTCCTTATGAACCACCAATCACAGCTGGGGTTGGTGATTCGTCATTGTTTGACCATTATCCAGAAGAACAATTAGACTACGGAAGCCAAGGAGA
AGATCCTTATGCTCTGTATTTCCTTGACTTTTGAATTGAGAGGAGATGAACTTTCATAAATGAATAAATGAGGCGCGCCATTATAAGTAAATGCATGTATACTAAACTCACAAAT
TAGAGCTTCAATTTAATTATATCAGTTATTACCCGGGAATCTCGGTCGTAATGATTTCTATAATGACGAAAAAAAAAAAATTGGAAAGAAAAAGCTTCATGGCCTTTATAAAAAG
GAACTATCCAATACCTCGCCAGAACCAAGTAACAGTATTTTACGGGGCACAAATCAAGAACAATAAGACAGGACTGTAAAGATGGACGCATAGATCCTGGAGGATGAGGAGA
CAGAAGTTAGTAGTAACAATTTGGGGACAGAAGAGGATACAGTGACATCAGAGGAAGAAGACAGCGAAGGTATAGTCAAAAAGCAATAGGACAGCAAGACAATTTATTTATT
TAATAGTTTTTTATAAATGTAAAATCTATCACAACATGTGTACTTAATTTTCTGTGGTAGGGGAGGCCATTTCGTTGAAACGTGTCGCGAGCGGTTGTGAGAATTGACTCATTTT
GATTATTACGCAACCATTGAAATTTTTTATTTTTTTTTGGTGAAGATTTTTCCCACACAACTTCTTCTTTTACTTAACCACCAACTACCGATAATGGATTTAGTCAATCATTTACCA
GACCGTTTGTTATTTGCTGTTCCTAAAAAGGGCAGATTATACGAAAAATGCTGTAACTTATTGAGTGGTGCCGATATACAGTTTAGAAGATCTAATAGATTAGATATAGCACTT
TCTACAAACTTGCCAATTGCATTAATCTTCTTGCCTGCAGCTGATATCCCAGTTTTCGTTGGAGAAGGTAATTGTGACTTGGGTATAACTGGGTTAGACCAAATCAAAGAAGCT
GAACAATTCGACAACATCGAGGACTTGTTGGATTTGAAATTTGGTTCATGCAAATTGCAGATCCAAGTTCCAGCAGATGGCGAGTACGAAAAGCCAGAACAGCTGTTGGAA
AGAAAATTGTGTCTTCATTTACAAAATTGAGTACCGACTATTTCAAACAATTGTCAGACAAACCTACTAATATCAGATATGTCGGTGGTTCCGTTGAGGCTTCTTGTGCCTTGG
GTGTTGCTGATGCTATTGTCGATTTGGTTGAAAGTGGTGAAACTATGAAAGCAGCTGGGTTAAAGGCCATAGAAACCATATTGGAGACTTCGGCTCATTTGATTTCGTCAAAA
AAAAGTAAATTCCCAGAAATGGTCAATATAATCGTCCAAAGACTTCAAGGTGTTTTAGCTGCCCAGGAATATGTCTTGTGTAACTACAATGCCCCAAAATCCATCCAAGCAAAA
TGTTTAACCATTACTCCAGGCAGAAGGGCCGCTACTGTCTCTACTTTGGACAAACACAGCGACGACGAAGAGGACTGGGTTGCCATCTCATCCATGGTTAATAGAAAGGAAA
TCGGTAATGTAATGGACGAATTGAAGAAAGCTGGTGCAACCGATATATTGGTACTCGAGATATCAAATTGTAGAGTTTAAAAGAAGTGATAGTTTCTCATAAATATTATACATA
TATACTTATAGATCTACTTCAATTTGACTCAATCTGTCTTATACCATCTTTATCTACCACCTTGATGTACACACCTTTAAAGTCAATAGGCATTCTCGTTTAAACGAGCTCAACAT
GTTGACTCTTCCTTGTCTGCTTTGTTTTTCGGTATTTTTTTTTATTTCTTTGCCTTTATGTATCTATATTAAATGTACAAAATATATTACATGATATTCCAAATATCTTGCTTTCTT
AAAAGTCTTTGAAAACTCAAACTAGCTATGGCCCTGTTGTATTCAATAAAAATTACAATGCATGATAGTAAACACACTAACAATACCCTTGACATGAGTCAAAATCAACCGCGG
ATCTGCCGGTCTCCCTATAGTGAGTCGTATTAATTTCGATAAGCCAGGTTAACCTGCATTAATGAATCGGCCAACGCGCGGGGAGAGGCGGTTTGCGTATTGGGCGCTCTTC
CGCTTCCTCGCTCACTGACTCGCTGCGCTCGGTCGTTCGGCTGCGGCGAGCGGTATCAGCTCACTCAAAGGCGGTAATACGGTTATCCACAGAATCAGGGGATAACGCAG
GAAAGAACATGTGAGCAAAAGGCCAGCAAAAGGCCAGGAACCGTAAAAAGGCCGCGTTGCTGGCGTTTTTCCATAGGCTCCGCCCCCCTGACGAGCATCACAAAAATCGAC
GCTCAAGTCAGAGGTGGCGAAACCCGACAGGACTATAAAGATACCAGGCGTTTCCCCCTGGAAGCTCCCTCGTGCGCTCTCCTGTTCCGACCCTGCCGCTTACCGGATACC
TGTCCGCCTTTCTCCCTTCGGGAAGCGTGGCGCTTTCTCATAGCTCACGCTGTAGGTATCTCAGTTCGGTGTAGGTCGTTCGCTCCAAGCTGGGCTGTGTGCACGAACCCC
CCGTTCAGCCCGACCGCTGCGCCTTATCCGGTAACTATCGTCTTGAGTCCAACCCGGTAAGACACGACTTATCGCCACTGGCAGCAGCCACTGGTAACAGGATTAGCAGAG
CGAGGTATGTAGGCGGTGCTACAGAGTTCTTGAAGTGGTGGCCTAACTACGGCTACACTAGAAGAACAGTATTTGGTATCTGCGCTCTGCTGAAGCCAGTTACCTTCGGAAA
AAGAGTTGGTAGCTCTTGATCCGGCAAACAAACCACCGCTGGTAGCGGTGGTTTTTTTGTTTGCAAGCAGCAGATTACGCGCAGAAAAAAAGGATCTCAA

*TPK2* promoter; SV40 NLS+linker; *TPK2* ORF; *HIS1* marker; *TPK2* 3’ HR

**References:**


1. Fonzi WA, Irwin MY. 1993. Isogenic strain construction and gene mapping in *Candida albicans*. Genetics 134:717–728.

2. Noble SM, Johnson AD. 2005. Strains and strategies for large-scale gene deletion studies of the diploid human fungal pathogen *Candida albicans*. Eukaryotic Cell 4:298–309.

3. Veri AO, Miao Z, Shapiro RS, Tebbji F, O'Meara TR, Kim SH, Colazo J, Tan K, Vyas VK, Whiteway M, Robbins N, Wong KH, Cowen LE. 2018. Tuning Hsf1 levels drives distinct fungal morphogenetic programs with depletion impairing Hsp90 function and overexpression expanding the target space. PLoS Genet 14:e1007270.

4. Min K, Ichikawa Y, Woolford CA, Mitchell AP. 2016. *Candida albicans* gene deletion with a transient CRISPR-Cas9 system. mSphere 1:e00130-16.

5. Liu Z, Myers LC. 2017. Mediator tail module is required for Tac1-activated *CDR1* expression and azole resistance in *Candida albicans*. Antimicrob Agents Chemother 61:e01342-17.

6. Zhang A, Petrov KO, Hyun ER, Liu Z, Gerber SA, Myers LC. 2012. The Tlo Proteins are stoichiometric components of *Candida albicans* mediator anchored via the Med3 subunit. Eukaryot Cell 11:874–884.

7. Tournu H, Luna-Tapia A, Peters BM, Palmer GE. 2017. In Vivo Indicators of cytoplasmic, vacuolar, and extracellular pH Using pHluorin2 in *Candida albicans*. mSphere 2:e00276-17.

8. Gerami-Nejad M, Zacchi LF, McClellan M, Matter K, Berman J. 2013. Shuttle vectors for facile gap repair cloning and integration into a neutral locus in Candida albicans. Microbiology 159:565–579.
